# General acid-mediated aminolactone formation using unactivated alkenes†

**DOI:** 10.1039/d3sc04073a

**Published:** 2023-09-28

**Authors:** David Just, Carlos R. Gonçalves, Uroš Vezonik, Daniel Kaiser, Nuno Maulide

**Affiliations:** a Institute of Organic Chemistry, University of Vienna Währinger Straße 38 1090 Vienna Austria nuno.maulide@univie.ac.at

## Abstract

Spirocyclic butyrolactones and butenolides are widespread structural motifs in bioactive substances. Despite their prevalence, a simple method ensuring their direct preparation from exocyclic alkenes, ideally in a late-stage context, remains elusive. Herein, we report direct aminolactone formation using unactivated alkenes which addresses this gap, employing cheap and readily available reactants. The method relies on the hijacking of a cationic aminoalkylation pathway and affords (spiro)aminolactones with excellent functional group tolerance and chemoselectivity. The synthetic versatility of the products is demonstrated through a range of transformations, notably exploiting stereospecific rearrangement chemistry to produce sterically congested scaffolds.

## Abstract 2

A transformation that delivers amino-butyrolactones from unactivated alkenes, characterized by its simplicity, ready availability of reagents and broad functional group tolerance, is reported.

## Graphical abstract

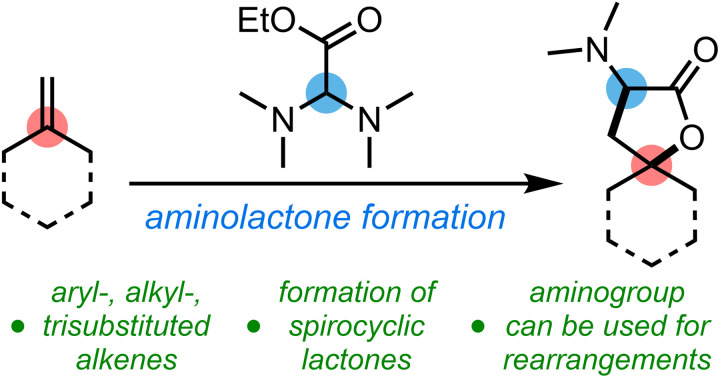

## Introduction

**Fig. 1:**
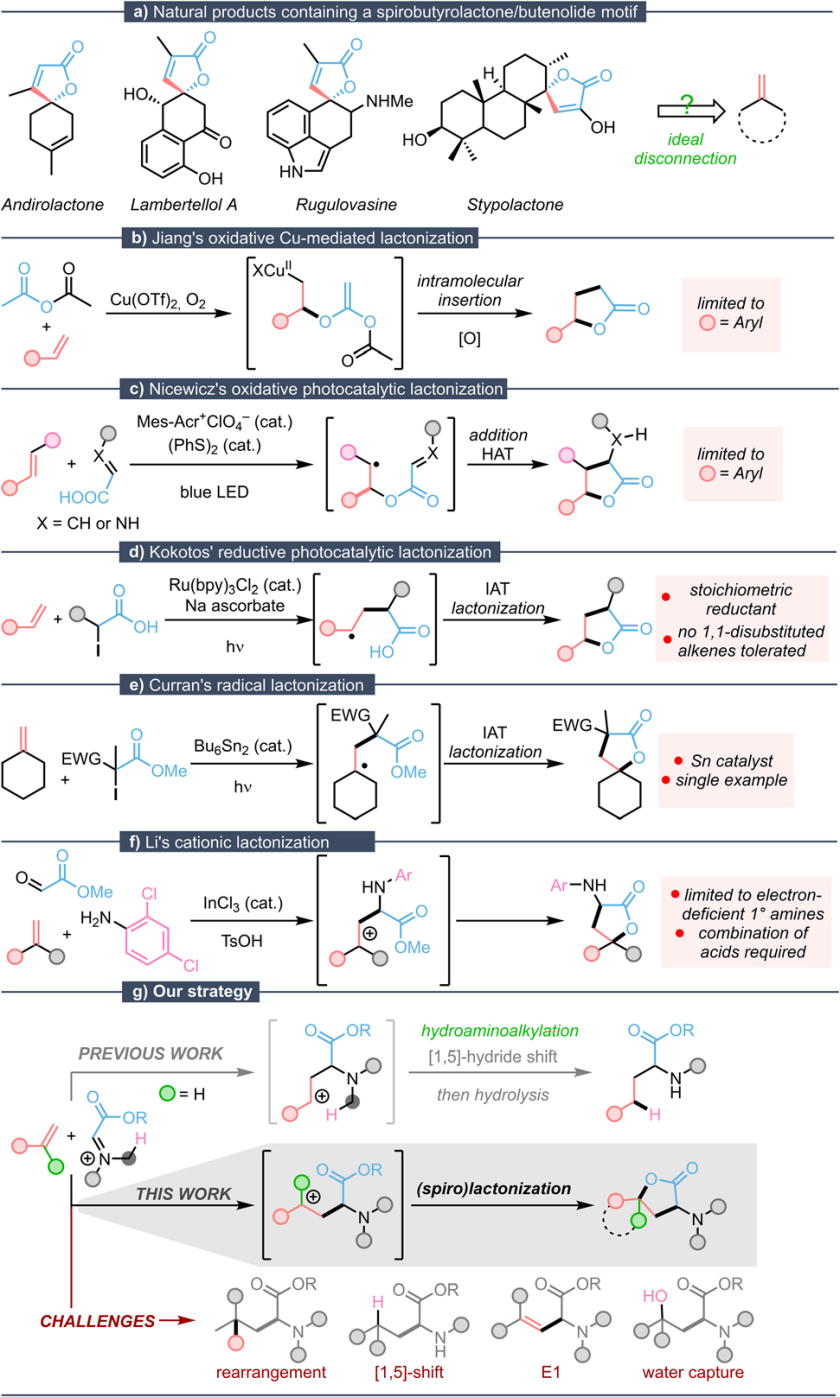
(a) Selected examples of natural products containing spiro-butyrolactone motives and their conceptual tracing back to an exocyclic methylene precursor. (b–f) State-of-the-art methods for the transformation of double bonds to butyrolactone cores. (g) Our novel, metal-free strategy for butyrolactone synthesis from alkenes.

Butyrolactones and butenolides are ubiquitous structural motifs.^1^ Spirocyclic butyrolactones and butenolides, such as those shown in Fig. 1a, are of particular importance: as a result of the spirocenter, they possess interesting conformational features that directly impact their biodynamic properties.^2^ For instance, the terpenoid Andirolactone^3^ has shown antibiotic and cytotoxic activity; the polyketide Lambertellol A^4^ possesses antifungal activity; the indole alkaloid Rugulovasine^5^ shows excellent affinity to 5-HT_1A_ receptors; and the diterpenoid Stypolactone^6^ exhibits activity towards human lung and colon carcinoma cell lines (Fig. 1a). There is a demand for facile and convenient methods to access these architectures, especially in late-stage contexts. So far, the most common synthetic approaches towards similar spirolactone cores either build on multistep transformations of cyclic ketones,^7^ or rely on coupling of the preassembled lactone core.^8^ Both directions are, however, associated with tedious multistep sequences. A straightforward strategy for the direct preparation of such constructs from exocyclic alkene precursors remains a sought-after approach.

A number of approaches to convert alkenes into butyrolactones are known. In 2010, Jiang described a Cu-catalyzed coupling of alkenes and anhydrides using oxygen as an oxidant (Fig. 1b).^9^ The method is limited to styrene derivatives, precluding access to important families of lactones. Later, the Nicewicz group developed an oxidative approach employing a Fukuzumi-type acridinium photocatalyst and substoichiometric quantities of a redox-active hydrogen atom donor (Fig. 1c).^10^ As this method relies on oxidation of the alkene to a radical cation, which is annulated with the unsaturated acid partner through polar-radical crossover, this method is limited to easily oxidizable alkenes such as styrenes.

More recently, the Kokotos group developed a reductive photocatalytic manifold utilizing Ru(bpy)_3_Cl_2_,^11^ improving on earlier, conceptually related protocols that required stoichiometric amounts of initiators^12^ or copper.^13^ This relies on the addition of a radical, stemming from an α-halocarboxylic acid, to the alkene followed by iodine atom transfer (IAT), after which the lactone is formed through intramolecular substitution (Fig. 1d). While amenable to a wider range of alkene partners, this protocol still necessitates a stoichiometric amount of ascorbate as the reducing agent. Moreover, 1,1-disubstituted alkenes—such as those that might result in spirocyclic products—were found to suffer competitive elimination processes, a notable limitation.

To the best of our knowledge, hitherto only few reports for the preparation of spirobutyrolactones from exocyclic alkene precursors exist. One such transformation was reported by Curran in 1989, where a radical one-pot atom-transfer addition/annulation of iodomalonate with methylenecyclohexane, catalyzed by hexabutylditin under visible-light irradiation, delivered a spirocyclic lactone carrying an electron-withdrawing substituent (Fig. 1).^14^ Intriguingly, a common feature of all methods presented above is the deployment of a metal-based catalyst in conjunction with a stoichiometric redox-active reagent or iodine-tailored reactants. Moreover, they are often limited to specific substrates, preventing general applicability. Another seminal example reported by Li relied on the use of primary anilines and aldehydes, as well as a combination of both a Brønsted and a Lewis acid (Fig. 1f).^15^ This transformation proceeds *via* the addition of the alkene to an iminium ion formed *in situ*, followed by cyclization with the adjacent ester. Importantly, however, this protocol is limited to polychlorinated—*i.e.* highly electron-deficient—primary anilines, underlining the importance of the electronic nature of the amines (as revealed by further studies in our laboratory).^16^

We have previously reported an approach to hydroaminoalkylation relying on rapid intermolecular aza-Prins-type reaction of electron-deficient iminium ions with alkenes, followed by an internal and redox-neutral reduction event (1,5-hydride transfer) (Fig. 1g, top).^17^ In contrast, the work we shall present in this communication details an alternative reaction outcome, observed when stabilized carbocationic intermediates reroute the reaction pathway (Fig. 1g, middle). Herein, we report the development of the first general, one-step cationic formation of (spiro)aminolactones from alkenes, including late-stage functionalization reactions and further product manipulations showcasing the power of this deceitfully simple transformation.

## Results and discussion

Spiroaminolactone formation was first observed when methylenecyclohexene (1a) was employed as the substrate for an attempted hydroaminoalkylation with an aminal derived from ethyl glyoxylate.^17*b*^ While we were intrigued by the unforeseen obtention of product 2a, the accompanying by-products in this initial experiment also made us keenly aware of the potential competing pathways (Fig. 1g, bottom). These potential pitfalls include not only the originally reported internal redox event by [1,5]-hydride transfer, but also processes of elimination and water capture of the intermediate, which were initially observed in varying amounts.

Following the finding that, when employing methylenecyclohexane (1a) as the substrate, the spirocyclic lactone 2a was formed in 57% yield (Table 1, entry 1), we engaged in further optimization of the process, pursuing a method which could deliver such products selectively. As we hypothesized that the observed reaction might benefit from increased stabilization of a (longer-lived) carbocationic intermediate, enabling capture by the proximal carboxylic ester moiety (see Fig. 1f, middle), we considered adding a polar co-solvent. While the addition of NMP (*N*-methyl-2-pyrrolidone) did lead to an increased yield (entry 2), it rendered isolation challenging, leading us to pursue alternatives. Ethyl acetate provided a slightly lower yield at the same temperature (50 °C, entry 3), but a decrease in temperature to 20 °C surprisingly led to an improved result (entry 4). An ethereal solvent, DME (1,2-dimethoxyethane), employed at the same temperature gave a satisfactory yield (entry 5), which was enhanced further by raising the reaction temperature to 70 °C (entry 6). However, concerns regarding the stability of the aminal reactant led us to select 50 °C as the optimal temperature, affording 2a in 82% isolated yield (entry 7).

**Table 1:** Optimization of the reaction conditions. Reactions were conducted on 0.2 mmol scale. Yields were determined by ^1^H-NMR using mesitylene as an internal standard, unless stated otherwise. Optimal reaction conditions: 1a (1 eq.), aminal (4 eq.), TFA/DME (1 : 1, 0.3 M), 50 °C, 20 h

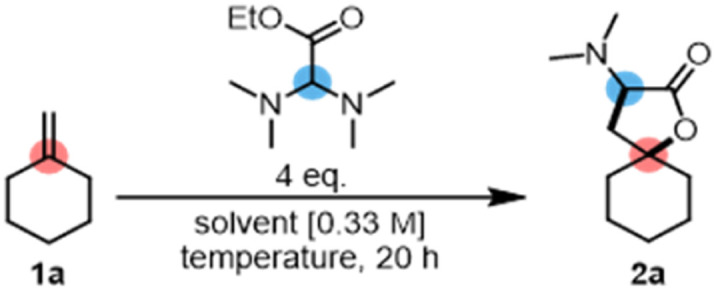			
Entry	Solvent	T (°C)	Yield (%)
1	TFA	50	57
2	TFA/NMP	50	75
3	TFA/EtOAc	50	63
4	TFA/EtOAc	20	80
5	TFA/DME	20	63
6	TFA/DME	70	78
**7**	**TFA/DME**	**50**	**85 (82)** a

a Isolated yield. For further details see the ESI.

**Scheme 1:**
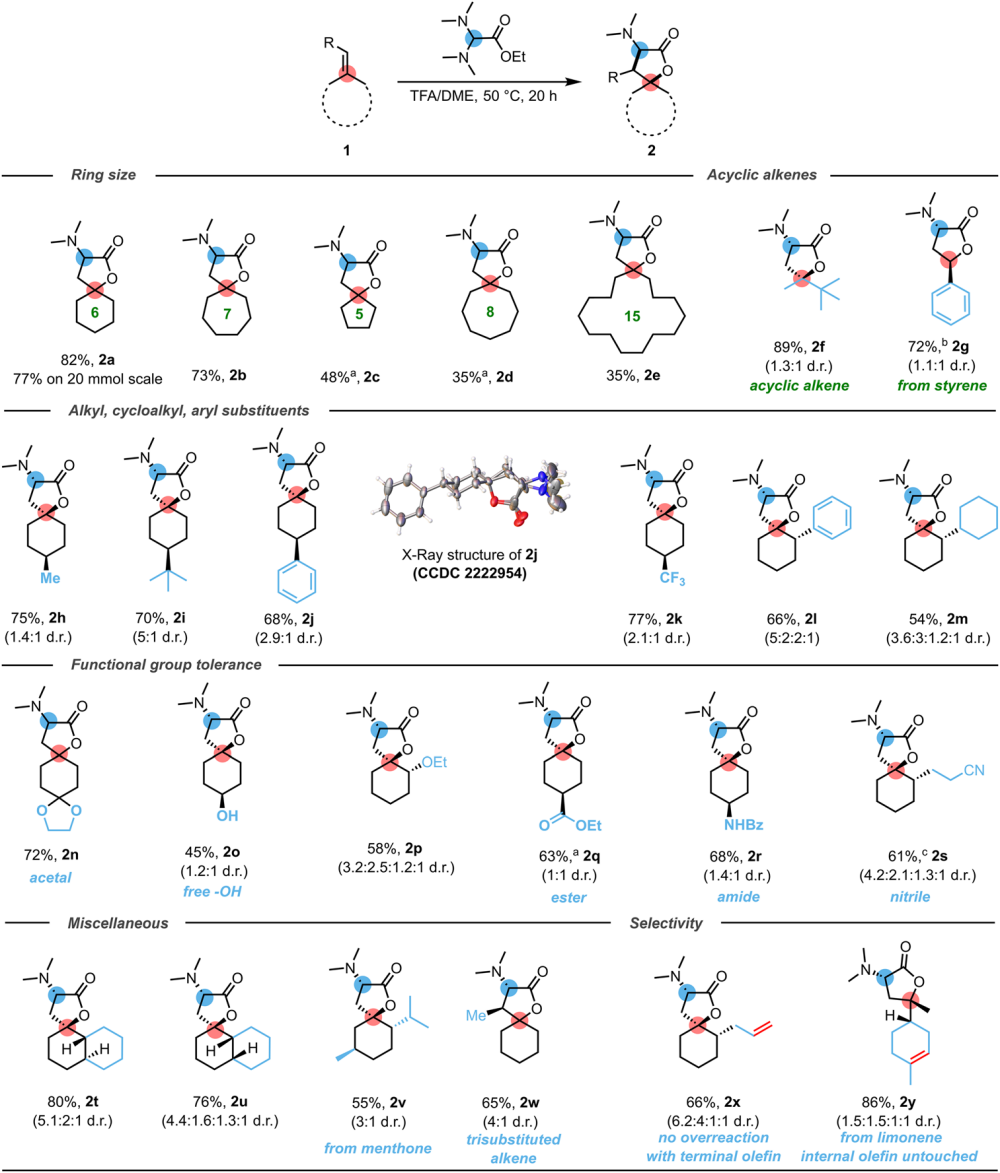
Substrate scope for the synthesis of α-amino butyrolactones. Reactions conducted on 0.2 mmol scale using 4 eq. aminal in a TFA/DME mixture 1 : 1 (0.3 M) at 50 °C for 20 h. ^*a*^Reaction performed using a TFA/EtOAc solvent mixture at 25 °C. ^*b*^Addition of alkene at 50 °C and heating the solvent mixture to 100 °C. ^*c*^Reaction performed using a TFA/DME solvent mixture at 75 °C. See the ESI† for details and for additional examples illustrating the reactivity patterns of other classes of alkenes.

To evaluate the scope of this method, an array of alkenes adorned with various functional groups was subjected to the optimized reaction conditions. The results are compiled in Scheme 1. In the exocyclic alkene series, the best results were obtained for six- and seven-membered ring substrates (1a and 1b), providing high yields for the corresponding (spiro)aminolactones 2a and 2b (Scheme 1), while methylenecyclopentane (1c) and -cyclooctane (1d) were, by comparison, less efficient. Interestingly, methylenecyclopentadecane (1e), featuring a 15-membered ring, was a competent substrate, yielding the spirolactone 2e and emphasizing the simplicity with which this process enables the opening of chemical space around the spirolactone motif. To further inquire whether the reaction is limited to cyclic systems, we investigated 2,3,3-trimethylbut-1-ene and styrene as potential substrates. The monocyclic lactone 2f was obtained in excellent 89% yield, while styrene-derived 2g was produced in good isolated yield, showing that the method presented herein is, to the best of our knowledge, the only lactone synthesis that can address a range of olefinic precursors (aryl-, alkyl, 1,1-disubstituted, and—as shown later—trisubstituted).

A range of substituents on the carbocycle (including methyl, *tert*-butyl, trifluoromethyl, phenyl and cyclohexyl) were tolerated, leading to products 2h–m. Interestingly, substitution at position 4 of the cyclohexane framework resulted only in two diastereomers (2h–2k)—the reason being the pseudochiral nature of the spiro-stereocenter. This pseudochirality^18^ imparts peculiar symmetry to the products, creating diastereomers only with respect to the relationship between the spiro-center and the substituent on the six-membered ring. Inverting the orientation of the C–N bond, on the other hand, only converts one enantiomer into the other (see ESI† for details). The structure of the major diastereomer (shown) was unambiguously confirmed by X-ray analysis (see crystal structure of 2j and ESI† for details).

When evaluating functional group tolerance, we found that acetals (2n),^19^ alcohols (2o),^20^ and ethers (2p), as well as other carboxylates and derivatives thereof, such as an ester (2q), amide (2r) or nitrile (2s), all were compatible with this acid-mediated protocol.

Moreover, both *trans*- and *cis*-decalin provided high yields for lactones 2t and 2u, and the alkene formed by methylenation of menthone also afforded 2v in good yield. Similarly, a trisubstituted alkene (1w) also provided the highly decorated lactone 2w in good yield, with an *anti* : *syn* ratio of 4 : 1.

To our delight, a terminal olefin (2x) was also well tolerated. Notably, despite having the potential to undergo hydroaminoalkylation, the allyl appendage of substrate 1t neither competed with the exocyclic double bond, nor did the formed product engage in overreaction. Further exquisite selectivity was observed for limonene (1y), which underwent exclusive reaction at the 1,1-disubstituted olefin, to form 2y (see the ESI† for additional examples illustrating the reactivity patterns of other classes of alkenes).

**Scheme 2:**
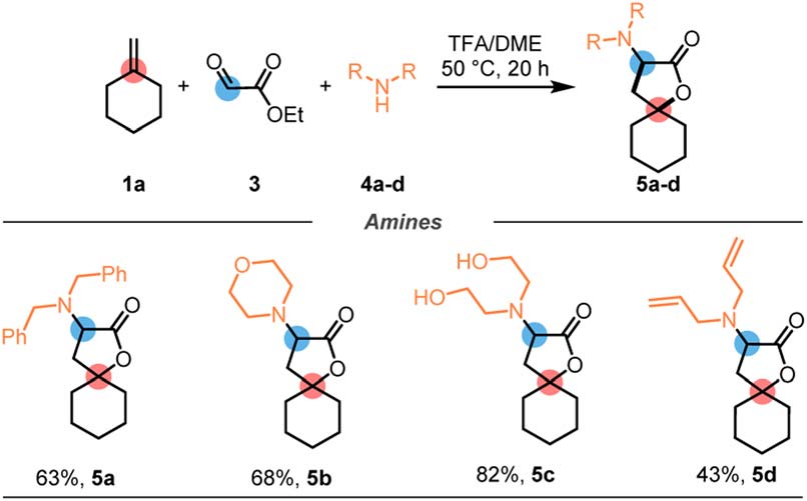
Substrate scope relying on *in situ* condensation of ethyl glyoxylate (3) and amine (4a–d). 4 eq. of 3 and 4a–d were employed. See ESI† for details.

Notably, the reaction is not limited to aminals bearing methyl groups on nitrogen, as highlighted by the diverse alkylamines shown in Scheme 2.

Importantly, we found that *in situ* condensation of ethyl glyoxalate with the desired amine^21^ is possible, ultimately rendering the reaction a three-component coupling of an alkene (1), glyoxylate (3), and an amine (4), to deliver spirolactones 5 with high levels of efficiency (Scheme 2). Several secondary amines (dibenzylamine 5a, morpholine 5b, diethanolamine 5c, and diallylamine 5d) were employed and gave the desired lactone products in good yields throughout, highlighting further the synthetic versatility of this method compared to previously established procedures.^16^

**Scheme 3:**
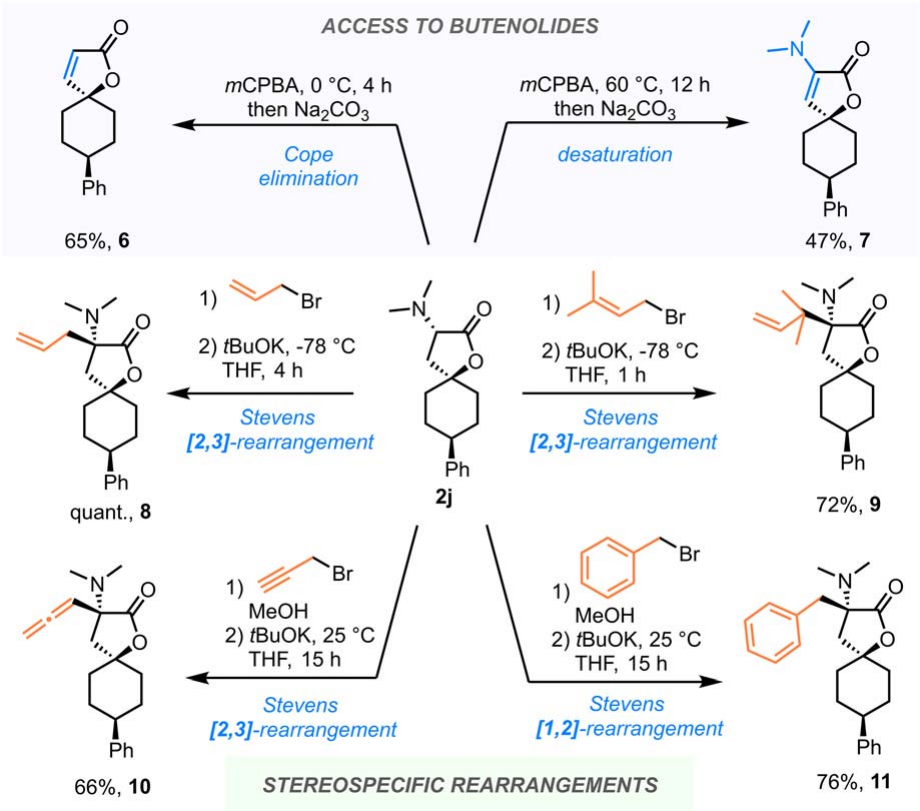
Post-synthetic modifications utilizing the amine as a functional handle. See ESI† for details.

We soon became intrigued by the synthetic possibilities made available by the amino functionality, which is a pervasive feature of the products of this reaction. To showcase its utility as a synthetic handle, several reactions of spirobutyrolactone 2h were studied in more detail, resulting in its successful transformation into a range of structurally distinct products (Scheme 3). Indeed, conversion to the respective amine *N*-oxide (using *m*CPBA at 0 °C) triggered Cope elimination, unmasking the spirocyclic butenolide 6 in 65% yield. Interestingly, deploying the same conditions, but at 60 °C, provided spiroaminobutenolide 7.^22^

Most interestingly, the amino group can serve as a handle for stereospecific functionalization at the α-position through rearrangement chemistry. As shown, facile alkylation of the amine moiety (to yield an intermediate ammonium salt) enables the deployment of a range of C–C bond forming processes. While allylation of 2h, followed by treatment with a base, delivers α-allyl-spirolactone 8, the use of prenyl- or propargyl bromide results in the products of reverse prenylation (9) or allenylation (10), which would arguably be very challenging to access otherwise. These appear to result from a [2,3]-Stevens rearrangement reaction manifold.^23^ In contrast, when benzyl bromide is used as the alkylating agent, the benzyl group migrates through a [1,2]-Stevens rearrangement pathway (*cf.*11),^24^ requiring higher temperature (25 °C) and longer reaction time. It is noteworthy that all these rearrangement products were obtained in high yields in a stereospecific manner.

## Conclusions

In summary, we have developed a transformation that delivers amino-butyrolactones from unactivated alkenes. The method distinguishes itself from the state-of-the-art in this area by its simplicity, ready availability of reagents and broad functional group tolerance. Importantly, this transformation can also be conducted as a three-component coupling, further enhancing its synthetic value. The products carry a versatile amino group which can be cleaved, retained, or harnessed as a handle for further stereospecific C–C bond formation, capitalizing on the elegance of sigmatropic rearrangements. We believe that this transformation is likely to find broad applicability in the field of bioactive-molecule synthesis.

## Data availability

All experimental data, and detailed experimental procedures are available in the published article and ESI.†

## Author contributions

The work was conceptualized by N. M. The experiments were performed by D. J., C. R. G., and U. V. The manuscript was written through contributions of all authors. D. K. and N. M. were involved in manuscript editing, finalizing and overall supervision of the project. N. M. secured funding and supervised the entire work.

## Conflicts of interest

There are no conflicts to declare.

## Supplementary Material

SC-014-D3SC04073A-s001

SC-014-D3SC04073A-s002
