# Supplementary material for: General acid-mediated aminolactone formation using unactivated alkenes
Source: Chem Sci. 2023 Sep 28;14(39):10806–11. doi: 10.1039/d3sc04073a (PMC10566462; doi:10.1039/d3sc04073a)
Supplement: SC-014-D3SC04073A-s001 [file SC-014-D3SC04073A-s001.pdf]

Electronic Supplementary Material (ESI) for Chemical Science.
This journal is © The Royal Society of Chemistry 2023

Supporting Information

A general acid-mediated aminolactone formation using unactivated alkenes

David Just[+], Carlos R. Gonçalves[+], Uroš Vezonik, Daniel Kaiser and Nuno Maulide*

Table of Contents

1. General information 3
2. Preparation of the starting materials. General procedure A 4
   2.1 Characterization of the starting materials 5
   2.2 Spectral data of the starting materials 9
3. General procedure B for the formation of aminolactones from alkenes 11
   3.1 Characterization of the aminolactones 12
4. General procedure C for the three-component formation of aminolactones from alkenes 28
   4.1 Characterization of the aminolactones formed using procedure C 29
5. Procedures and characterization: Derivatization of the spiroaminolactones 31
6. Assessment of the reactivity of different classes of alkenes 36
7. Comparison with the Huang & Li procedure[14] 38
8. Stereochemical assignments 39
9. X-Ray crystallographic data of 2j 42
10. Spectral data 47
11. References 90

1. General information

All glassware was oven dried at 100 °C before use. All solvents were distilled from appropriate drying agents prior to use or directly taken from commercial sealed bottles under an atmosphere of argon. All reagents were used as received from commercial suppliers unless otherwise stated. Neat infrared spectra were recorded using a Perkin-Elmer Spectrum 100 FT-IR spectrometer. Wavenumbers (v) are reported in cm^{-1}. Mass spectra were obtained using a Finnigan MAT 8200 or (70 eV) or an Agilent 5973 (70 eV) spectrometer, using electrospray ionization (ESI). All ^{1}H NMR, ^{13}C NMR, and ^{19}F NMR experiments were recorded using Bruker AV-400, AV-600 and AV-700 spectrometers at 300 K. Chemical shifts (δ) are quoted in ppm and coupling constants (*J*) are quoted in Hz. The 7.26 ppm resonance of residual $CHCl_3$ for ^{1}H NMR spectra and 77.16 ppm resonance for ^{13}C NMR spectra were used as internal references. ^{1}H-NMR splitting patterns were designated as singlet (s), doublet (d), triplet (t), quartet (q), quintet (quint) or combinations thereof, as well as broad signal (br). Splitting patterns that could not be interpreted were designated as multiplet (m). Spatial long-range contacts were determined by $^{1}H,^{1}H$ NOESY experiments. $CDCl_3$ was dried over 3Å molecular sieves prior to the measurements. Reaction progress was monitored by thin layer chromatography (TLC) performed on aluminum plates coated with kieselgel F254 with 0.2 mm thickness. Visualization was achieved by a combination of ultraviolet light (254 nm) and acidic potassium permanganate. Flash column chromatography was performed using silica gel 60 (230–400 mesh, Merck and co.). Aminal **A** - ethyl 2,2-bis(dimethylamino)acetate – was prepared according to the literature.[1]

2. Preparation of the starting materials. General procedure A

Unless mentioned explicitly below, the starting materials were obtained from commercial sources and used as received.

Those alkenes that were not commercially available were synthesized using the following general procedure.

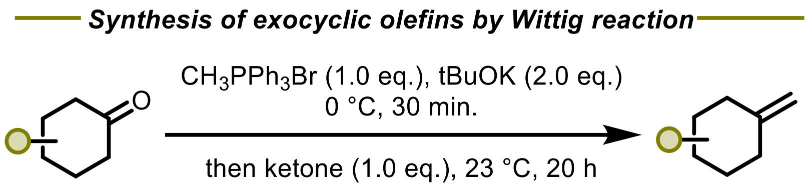

1

A suspension of methyltriphenylphosphonium bromide (1.0 eq., 5.0 mmol) in anhydrous Et_2O (50 mL, 0.1 M) was cooled to 0 °C, after which *t*BuOK (2.0 eq., 10 mmol) was added. The resulting mixture was stirred at the same temperature for 30 min, with a noticeable color change. After that time, a solution of the ketone (1.0 eq., 5 mmol) in anhydrous Et_2O (10 mL, precooled to 0 °C), was added to the reaction mixture, after which it was allowed to warm to 23 °C over the course of 14 h. The reaction was monitored by TLC and gently heated if little conversion was observed. After completion, excess base was quenched by the addition of a saturated aqueous solution of NH_4Cl, and extracted with Et_2O (3 × 20 mL). The pooled organic layers were dried over $MgSO_4$ and concentrated under reduced pressure. The crude product was purified by flash column chromatography (silica gel, pentane) to obtain alkenes **1**.

2.1 Characterization of the starting materials

Methylenecycloheptane (1b)

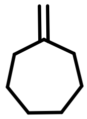

The title compound was prepared using general procedure **A** for the synthesis of exocyclic alkenes. After flash chromatography, the compound was isolated in 72% yield as a colorless oil. All spectral data was found to be in accordance with the literature.[2]

Methylenecyclooctane (1d)

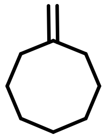

The title compound was prepared using general procedure **A** for the synthesis of exocyclic alkenes. After flash chromatography, the compound was isolated in 53% yield as a colorless oil. All spectral data was found to be in accordance with the literature.[3]

1-Methyl-4-methylenecyclohexane (1h)

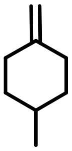

The title compound was prepared using general procedure **A** for the synthesis of exocyclic alkenes. After flash chromatography, the compound was isolated in 15% yield as a volatile colorless oil. All spectral data was found to be in accordance with the literature.[4]

1-(*tert*-Butyl)-4-methylenecyclohexane (1i)

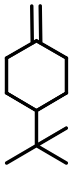

The title compound was prepared using general procedure **A** for the synthesis of exocyclic alkenes. After flash chromatography, the compound was isolated in 90% yield as a colorless oil. All spectral data was found to be in accordance with the literature.[4-5]

(4-Methylenecyclohexyl)benzene (1j)

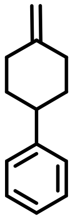

The title compound was prepared using general procedure **A** for the synthesis of exocyclic alkenes. After flash chromatography, the compound was isolated in 75% yield as a colorless oil. All spectral data was found to be in accordance with the literature.[5]

1-Methylene-4-(trifluoromethyl)cyclohexane (1k)

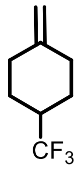

The title compound was prepared using general procedure **A** for the synthesis of exocyclic alkenes. After flash chromatography, the compound was isolated in 39% yield as a volatile colorless oil. All spectral data was found to be in accordance with the literature.[6]

(2-Methylenecyclohexyl)benzene (1l)

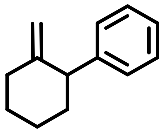

The title compound was prepared using general procedure **A** for the synthesis of exocyclic alkenes. After flash chromatography, the compound was isolated in 32% yield as a colorless oil. All spectral data was found to be in accordance with the literature.[7]

2-Methylene-1,1'-bi(cyclohexane) (1m)

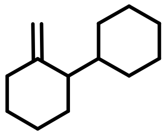

The title compound was prepared using general procedure **A** for the synthesis of exocyclic alkenes. After flash chromatography, the compound was isolated in 67% yield as a colorless oil.

^{1}H NMR (400 MHz, $CDCl_3$) **δ** 4.65 – 4.62 (m, 1H), 4.54 (d, J = 2.6 Hz, 1H), 2.11 – 2.03 (m, 2H), 1.92 – 1.61 (m, 8H), 1.55 – 1.36 (m, 5H), 1.29 – 1.09 (m, 3H), 0.87 – 0.72 (m, 2H); **^{13}C NMR** (101 MHz, $CDCl_3$)**: δ** 152.2, 107.3, 49.7, 35.8, 33.2, 32.1, 30.7, 29.4, 28.9, 26.8, 26.8, 22.2; **IR** (neat, cm^{-1}): 2921, 2894, 2850, 1646, 1147, 889.

8-Methylene-1,4-dioxaspiro[4.5]decane (1n)

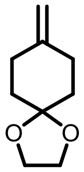

The title compound was prepared using general procedure **A** for the synthesis of exocyclic alkenes. After flash chromatography, the compound was isolated in 65% yield as a colorless oil. All spectral data was found to be in accordance with the literature.[8]

***tert*-Butyldimethyl((4-methylenecyclohexyl)oxy)silane (1o)**

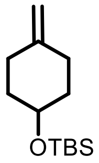

The title compound was prepared using general procedure **A** for the synthesis of exocyclic alkenes. After flash chromatography, the compound was isolated in 86% yield as a volatile colorless oil. All spectral data was found to be in accordance with the literature.[4]

1-Ethoxy-2-methylenecyclohexane (1p)

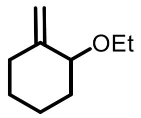

The title compound was prepared using general procedure **A** for the synthesis of exocyclic alkenes. After flash chromatography, the compound was isolated in 23% yield as a volatile colorless oil. All spectral data was found to be in accordance with the literature.[9]

Ethyl 4-methylenecyclohexane-1-carboxylate (1q)

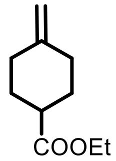

The title compound was prepared using general procedure **A** for the synthesis of exocyclic alkenes. After flash chromatography, the compound was isolated in 83 % yield as a volatile colorless oil. All spectral data was found to be in accordance with the literature.[5]

***N*-(4-Methylenecyclohexyl)benzamide (1r)**

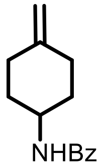

The title compound was prepared using general procedure **A** for the synthesis of exocyclic alkenes. After flash chromatography, the compound was isolated in 37% yield as an off-white solid.

^{1}H NMR (700 MHz, $CDCl_3$)**: δ** 7.76 – 7.73 (m, 2H), 7.47 – 7.43 (m, 1H), 7.38 (t, *J* = 7.7 Hz, 2H), 6.23 (s, 1H), 4.66 (s, 2H), 4.15 – 4.09 (m, 1H), 2.32 (dt, *J* = 13.8, 3.3 Hz, 2H), 2.17 (td, *J* = 13.5, 4.0 Hz, 2H), 2.11 – 2.06 (m, 2H), 1.40 – 1.34 (m, 2H); **^{13}C NMR** (176 MHz, $CDCl_3$)**: δ** 166.9, 147.3, 135.0, 131.4, 128.6, 127.0, 108.4, 48.3, 33.9, 33.1; **HRMS** (ESI^+): calculated for $C_{14}H_{18}ON$ $[M+H]^+$ *m/z*: 216.1388, found: 216.1381; **IR** (neat, cm^{-1}): 3306 2937, 2857, 1648, 1578, 1209, 904.

3-(2-Methylenecyclohexyl)propanenitrile (1s)

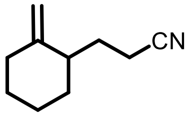

The title compound was prepared using general procedure **A** for the synthesis of exocyclic alkenes. After flash chromatography, the compound was isolated in 89% yield as a volatile colorless oil. All spectral data was found to be in accordance with the literature.[10]

(4a*S,8a*R**)-1-Methylenedecahydronaphthalene (1t)**

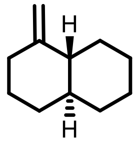

The title compound was prepared using general procedure **A** for the synthesis of exocyclic alkenes. After flash chromatography, the compound was isolated in 78% yield as a colorless oil.[11]

(4a*S,8a*S**)-1-Methylenedecahydronaphthalene (1u)**

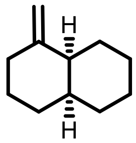

The title compound was prepared using general procedure **A** for the synthesis of exocyclic alkenes. After flash chromatography, the compound was isolated in 75% yield as a colorless oil.[11]

(1*S*,4*R*)-1-Isopropyl-4-methyl-2-methylenecyclohexane (1v)

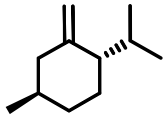

The title compound was prepared using general procedure for the synthesis of exocyclic alkenes. After flash chromatography, the compound was isolated in 85% yield as a volatile colorless oil. All spectral data was found to be in accordance with the literature.[12]

1-Allyl-2-methylenecyclohexane (1x)

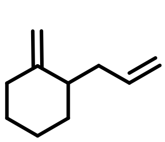

The title compound was prepared using general procedure **A** for the synthesis of exocyclic alkenes. After flash chromatography, the compound was isolated in 45% yield as a volatile colorless oil. All spectral data was found to be in accordance with the literature.[13]

2.2 Spectral data of the starting materials

2-Methylene-1,1'-bi(cyclohexane) (1m)

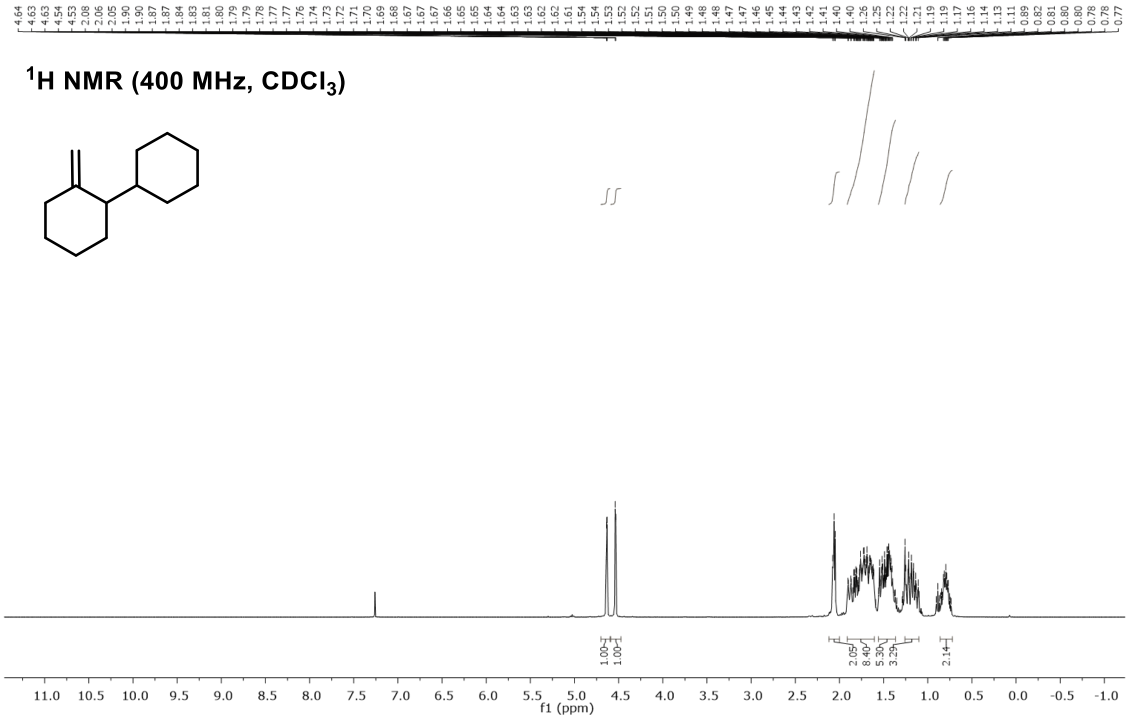

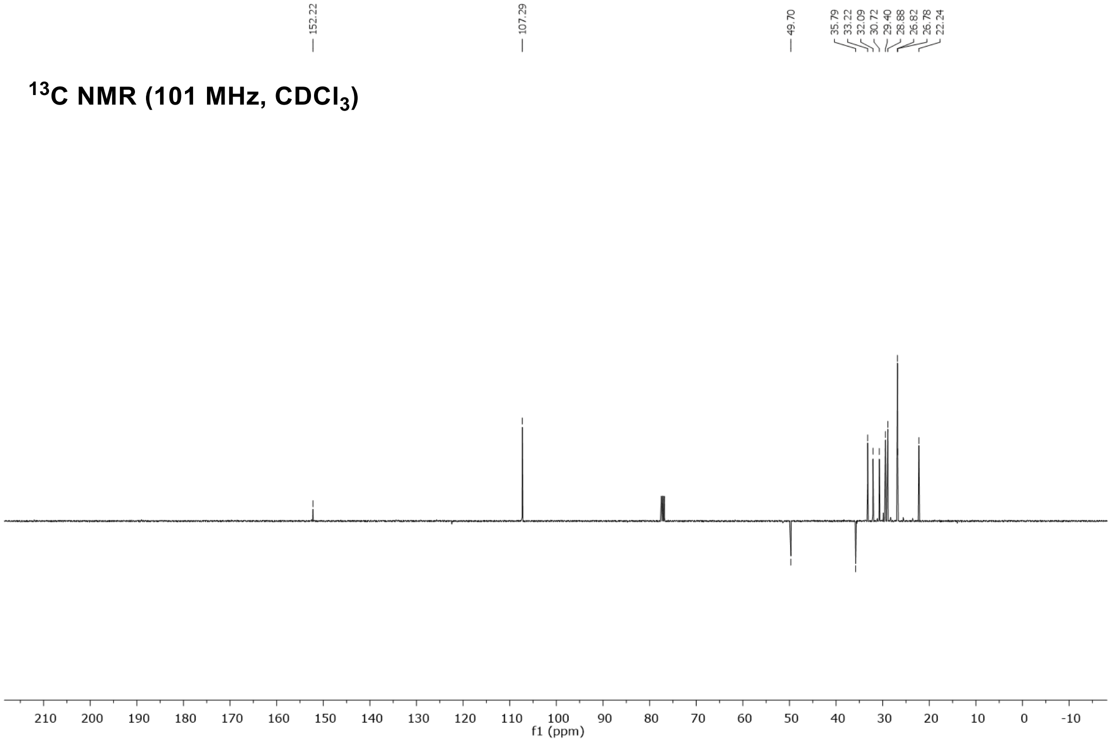

N-(4-Methylenecyclohexyl)benzamide (1r)

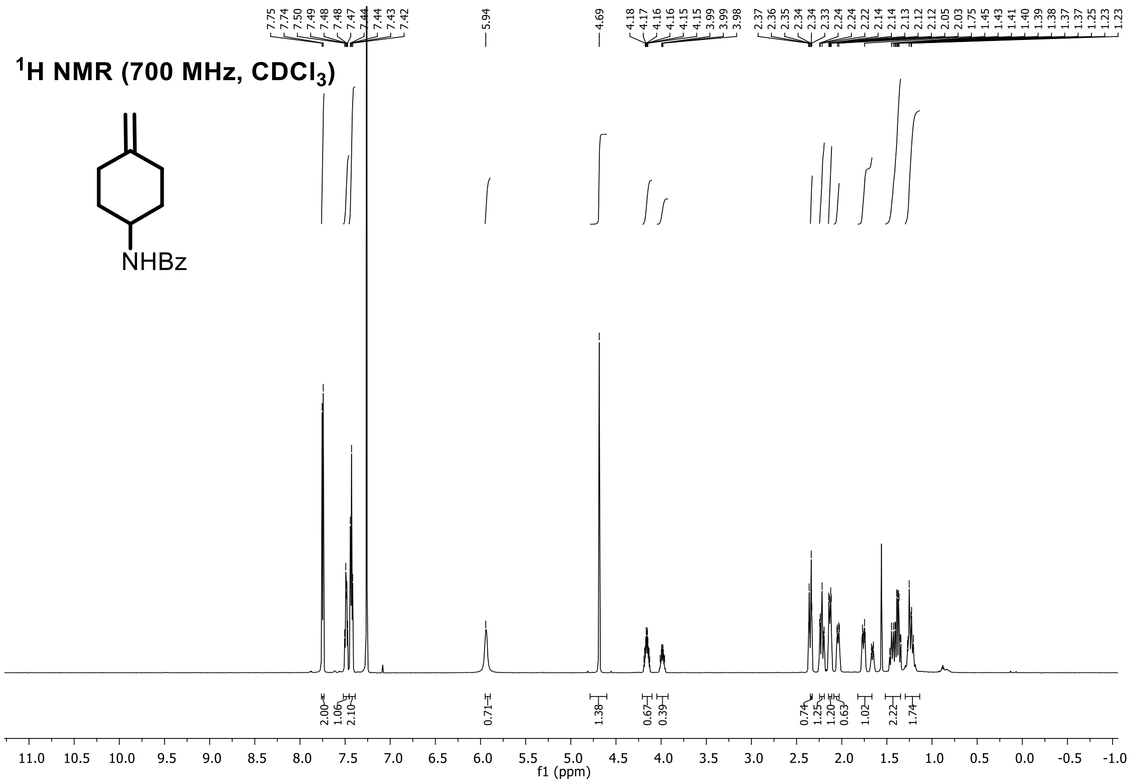

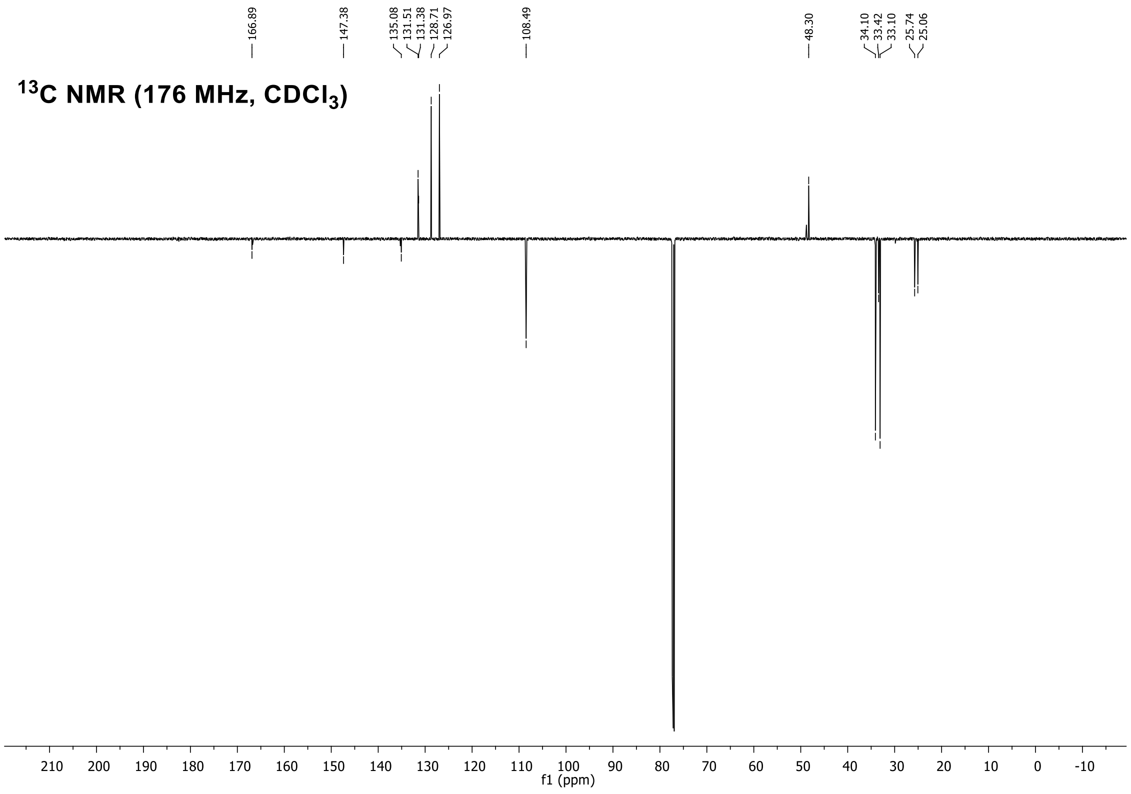

3. General procedure B for the formation of aminolactones from alkenes

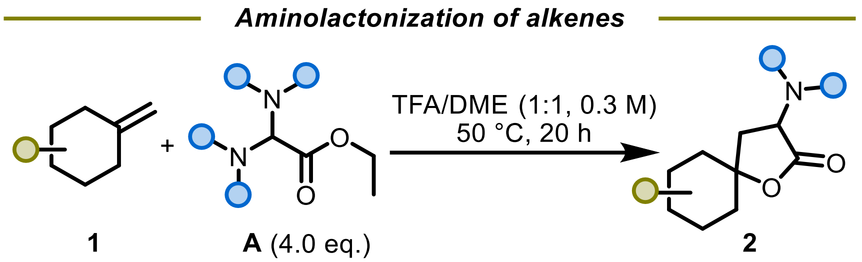

All reactions were performed on a 0.2 mmol scale.

To a flame-dried Schlenk flask charged with the aminal **A** (synthesized following the procedure reported in reference [1], 4.0 eq., 0.8 mmol) was added trifluoroacetic acid (0.33 mL, 0.6 M), then the mixture was cooled to 0 °C. After 5 min, the corresponding alkene **1** (1.0 eq., 0.2 mmol) was added as a solution in anhydrous 1,2-dimethoxyethane (0.33 mL). The reaction was heated to 50 °C and vigorously stirred for 20 h (unless otherwise stated), after which it was cooled to 0 °C. Then, an aqueous solution of NaOH (1.0 M) was added until a pH >7 was reached. The resulting biphasic mixture was separated and the aqueous phase was extracted with DCM (3 × 50 mL/mmol). The combined organic phases were then dried over anhydrous K_2CO_3 and filtered. The filtrate was concentrated under reduced pressure to afford the crude product, which was purified by flash column chromatography on silica gel with DCM-DMA (DMA = solvent mixture of dichloromethane, methanol, aq. NH_4OH in the ratio 9:1:0.15) or heptane-EEA (EEA = solvent mixture of ethyl acetate, ethanol, aq. NH_4OH in the ratio 3:1:0.08) to afford the analytically pure desired products **2**.

3.1 Characterization of the aminolactones

3-(Dimethylamino)-1-oxaspiro[4.5]decan-2-one (2a)

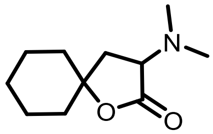

The title compound was prepared using general procedure **B** from alkene **1a**. Purification by column chromatography on silica gel (5% DMA to 10% DMA in DCM) afforded compound **2a** in 82% yield (30.1 mg) as a colorless oil. Reaction on 20 mmol scale provided the product in 77% yield (3.04 g).

^{1}H NMR (600 MHz, $CDCl_3$)**: δ** 3.70 (t, *J* = 10.1 Hz, 1H), 2.40 (s, 6H), 2.23 - 2.16 (m, 1H), 1.89 (t, *J* = 12.0 Hz, 1H), 1.77 - 1.63 (m, 4H), 1.56 - 1.49 (m, 4H), 1.42 - 1.37 (m, 1H), 1.30 - 1.25 (m, 1H); **^{13}C NMR** (151 MHz, $CDCl_3$)**: δ** 174.8, 82.9, 63.7, 41.8 (C2), 38.5, 36.7, 34.4, 25.1, 22.7, 22.6; **HRMS (**ESI$^+$**)**: calculated for $C_{11}H_{19}O_2N_2$ $[M+H]^+$ 198.1494; found 198.1496; **IR (**neat, cm^{-1}**)**: 2930, 2870, 1762, 1410, 1320, 730.

3-(Dimethylamino)-1-oxaspiro[4.6]undecan-2-one (2b)

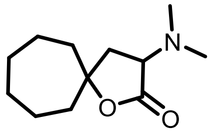

The title compound was prepared using general procedure **B** from alkene **1b**. Purification by column chromatography on silica gel (10% DMA to 30% DMA in DCM) afforded compound **2b** in 73% yield (26.7 mg) as a colorless oil.

^{1}H NMR (600 MHz, $CDCl_3$)**: δ** 3.66 - 3.71 (m, 1H), 2.41 (s, 6H), 2.24 - 2.19 (m, 1H), 2.05 - 2.01 (m, 1H), 1.95 - 1.92 (m, 1H), 1.84 - 1.79 (m, 1H), 1.77 - 1.67 (m, 4H), 1.57 - 1.55 (m, 4H), 1.42 - 1.25 (m, 2H); **^{13}C NMR** (151 MHz, $CDCl_3$)**: δ** 174.8, 82.7, 63.8, 42.3, 41.9, 39.3, 35.7, 29.2, 29.0, 25.1, 22.3 (2C); **HRMS (**ESI$^+$**)**: calculated for $C_{12}H_{22}O_2N$ $[M+H]^+$ *m/z*: 212.1651, found: 212.1648; **IR** (neat, cm^{-1}): 2938, 2860, 1770, 1263, 1152, 1103, 760.

3-(Dimethylamino)-1-oxaspiro[4.4]nonan-2-one (2c)

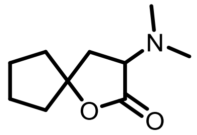

The title compound was prepared using general procedure **B** from alkene **1c** using EtOAc as the cosolvent at 25 °C. Purification by column chromatography on silica gel (10% EEA to 80% EEA in heptane) afforded compound **2c** in 48% yield (17.5 mg) as a colorless oil.

^{1}H NMR (400 MHz, $CDCl_3$)**: δ** 3.67 (dd, *J* = 11.4, 8.6 Hz, 1H), 2.42 (s, 6H), 2.26 (dd, *J* = 12.6, 11.4 Hz, 1H), 2.20 (dd, *J* = 12.6, 8.6 Hz, 1H), 2.08 – 2.00 (m, 1H), 1.95 – 1.78 (m, 3H), 1.78 – 1.62 (m, 4H); **^{13}C NMR** (101 MHz, $CDCl_3$)**: δ** 174.6, 91.1, 64.6, 41.8 (2C), 39.1, 38.6, 33.9, 24.2, 23.3; **HRMS** (ESI^+): calculated for $C_{10}H_{18}O_2N$ $[M+H]^+$ 184.1338; found 184.1330; **IR** (neat, cm^{-1}): 2959, 2936, 2871, 2830, 2786, 1730, 1456, 1370, 1294, 1267, 1246, 1204, 1162, 1152, 1098, 1033, 741.

3-(Dimethylamino)-1-oxaspiro[4.4]dodecan-2-one (2d)

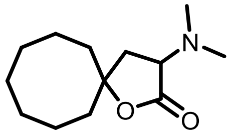

The title compound was prepared using general procedure **B** from alkene **1d** using EtOAc as the cosolvent at 25 °C. Purification by column chromatography on silica gel (10% EEA to 50% EEA in heptane) afforded compound **2d** in 35% yield (15.8 mg) as a colorless oil.

^{1}H NMR (400 MHz, $CDCl_3$)**: δ** 3.69 (dd, *J* = 11.1, 9.2 Hz, 1H), 2.40 (s, 6H), 2.22 (dd, *J* = 12.8, 9.1 Hz, 1H), 2.07 – 1.92 (m, 2H), 1.87 (dd, *J* = 12.7, 11.2 Hz, 1H), 1.79 – 1.56 (m, 8H), 1.56 – 1.38 (m, 4H); **^{13}C NMR** (101 MHz, $CDCl_3$)**: δ** 174.7, 86.7, 64.0, 41.8 (2C), 37.6, 34.8, 34.4, 28.03, 27.97, 24.6, 22.2, 21.8; **HRMS** (ESI^+): calculated for $C_{13}H_{24}O_2N$ $[M+H]^+$ 226.1807; found 226.1804; **IR** (neat, cm^{-1}): 2923, 2862, 2786, 1763, 1474, 1450, 1255, 1201, 1174, 1130, 1097, 1070, 1031, 987, 946.

3-(Dimethylamino)-1-oxaspiro[4.14]nonadecan-2-one (2e)

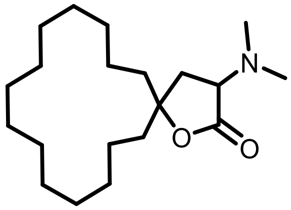

The title compound was prepared using general procedure **B** from alkene **1e**. Purification by column chromatography on silica gel (15% EEA to 60% EEA in heptane) afforded compound **2e** in 35% yield (22.7 mg) as a colorless oil.

^{1}H NMR (400 MHz, $CDCl_3$)**: δ** 3.70 (t, *J* = 10.1 Hz, 1H), 2.40 (s, 6H), 2.20 – 2.14 (m, 1H), 1.90 (t, *J* = 12.0 Hz, 1H), 1.84 – 1.78 (m, 1H), 1.69 – 1.64 (m, 1H), 1.60 – 1.49 (m, 2H), 1.37 – 1.31 (m, 24H**)**; **^{13}C NMR** (101 MHz, $CDCl_3$)**: δ** 174.8, 86.0, 63.9, 41.9 (2C), 38.8, 36.8, 34.2, 27.6 (2C), 27.1 (2C), 26.8, 26.7 (3C), 26.4, 21.7, 22.1, 21.7; **HRMS** (ESI^+): calculated for $C_{20}H_{38}O_2N$ $[M+H]^+$ 324.2903; found 324.2894; **IR** (neat, cm^{-1}): 2927, 2856, 1766, 1458, 1215, 1160, 1144

(3*S,5*S**)-5-(*tert*-Butyl)-3-(dimethylamino)-5-methyldihydrofuran-2(3*H*)-one (2f)**

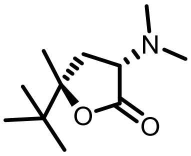

The title compound was prepared using general procedure **B** from alkene **1f**. Purification by column chromatography on silica gel (10% DMA in DCM to 60% DMA) afforded compound **2f** in 89% yield (24.2 mg) in 1.3:1 d.r. as an off-white oil.

^{1}H NMR (600 MHz, $CDCl_3$)**: δ** 3.77 (dd, *J* = 11.4, 9.2 Hz, 1H, d1), 3.67 (dd, *J* = 10.7, 8.8 Hz, 1H, d2), 2.46 – 2.43 (m, 1H, d1), 2.41 (s, 6H, d1), 2.39 (s, 6H, d2), 2.29 (t, *J* = 12.1 Hz, 1H, d2), 1.87 (dd, *J* = 12.7, 9.1 Hz, 1H, d1), 1.83 – 1.78 (m, 1H, d2), 1.40 (s, 3H, d1), 1.32 (s, 3H, d2), 0.98 (s, 9H, d1), 0.95 (s, 9H, d2); **^{13}C NMR** (151 MHz, $CDCl_3$)**: δ** 175.3 (d2), 174.9 (d1), 89.0 (d1), 87.5 (d2), 65.3 (d1), 64.2 (d2), 41.8 (d1, C2), 41.7 (d2, C2), 38.1 (d2), 37.4 (d1), 31.3 (d2), 29.8 (d1), 25.0 (d1, d2), 24.9 (d1), 24.8 (d2); **HRMS** (ESI^+): calculated for $C_{11}H_{22}O_2N$ $[M+H]^+$ *m/z*: 200.1651, found: 200.1647; **IR** (neat, cm^{-1}): 2962, 2878, 2784, 1759, 1398, 1201.

(3*S,5*R**)-3-(Dimethylamino)-5-phenyldihydrofuran-2(3*H*)-one (major diastereomer 2g)**

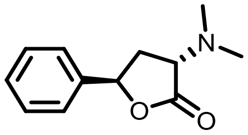

The title compound was prepared using general procedure **B** from alkene **1g**. The alkene was added at 50 °C and the reaction was heated at 100 °C. Purification by column chromatography on silica gel (10% to 25% EEA in heptane) afforded compound **2g** in 72% yield (30.5 mg) as a 1.1:1 mixture of partially separable diastereomers, as a colorless oil (analytical sample contained diastereoisomers).

^{1}H NMR (400 MHz, $CDCl_3$)**: δ** 7.41 – 7.39 (m, 2H), 7.35 – 7.34 (m, 1H), 7.32 – 7.28 (m, 2H), 5.61 (dd, *J* = 8.1, 4.3 Hz, 1H), 3.56 (t, *J* = 8.4 Hz, 1H), 2.71 (dt, *J* = 13.3, 8.2 Hz, 1H), 2.44 (s, 6H), 2.33 (dd, *J* = 8.7, 4.4 Hz, 1H); **^{13}C NMR** (101 MHz, $CDCl_3$)**: δ** 175.1, 139.9, 129.0 (C2), 128.5, 125.1 (C2), 78.3, 62.5, 42.3 (C2), 32.6; **HRMS** (ESI^+): calculated for $C_{12}H_{16}O_2N$ $[M+H]^+$ 206.1181; found 206.1177; **IR** (neat, cm^{-1}): 3034, 2981, 1785, 1774, 1476, 1166, 719.

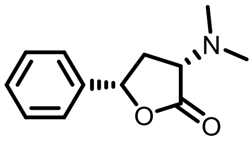

^{1}H NMR (400 MHz, $CDCl_3$)**: δ** 7.43 – 7.38 (m, 2H), 7.38 – 7.31 (m, 3H), 5.35 – 5.28 (m, 1H), 3.81 – 3.75 (m, 1H), 2.67 (ddd, *J* = 12.7, 8.1, 5.7 Hz, 1H), 2.46 (s, 6H), 2.22 – 2.13 (m, 1H); **^{13}C NMR** (101 MHz, $CDCl_3$)**: δ** 174.6, 139.0, 129.0 (2C), 128.8, 125.6 (2C), 77.8, 65.0, 42.0 (C2), 32.9.

(3*S,5*s**,8*R**)-3-(Dimethylamino)-8-methyl-1-oxaspiro[4.5]decan-2-one**

(major diastereomer 2h)

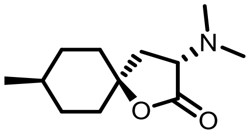

The title compound was prepared using general procedure **B** from alkene **1h** on 0.14 mmol scale. Purification by column chromatography on silica gel (10% EEA to 80% EEA in heptane) afforded compound **2h** in 75% yield (22.1 mg) in 1.4:1 d.r. as a colorless oil. Diastereomers were separable.

^{1}H NMR (400 MHz, $CDCl_3$)**: δ** 3.70 (dd, *J* = 11.2, 9.1 Hz, 1H), 2.40 (s, 6H), 2.08 (dd, *J* = 12.8, 9.1 Hz, 1H), 1.95 (dd, *J* = 12.7, 11.3 Hz, 1H), 1.89 – 1.77 (m, 2H), 1.62 – 1.51 (m, 3H), 1.49 – 1.34 (m, 3H), 1.34 – 1.24 (m, 1H), 0.91 (d, *J* = 5.9 Hz, 3H); **^{13}C NMR** (101 MHz, $CDCl_3$)**: δ** 174.9, 82.0, 63.7, 41.9 (2C), 38.1, 36.9, 35.7, 31.6, 30.7, 30.2, 22.1; **HRMS** (ESI^+): calculated for $C_{12}H_{22}O_2N$ $[M+H]^+$ 212.1651; found 212.1648; **IR** (neat, cm^{-1}): 2946, 2925, 2867, 2858,

2785, 1764, 1456, 1372, 1341, 1304, 1243, 1207, 1158, 1129, 1097, 1077, 1044, 1030, 1010, 982, 952, 929, 811, 798, 754, 700.

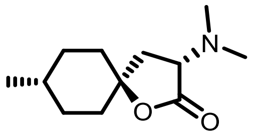

^{1}H NMR (400 MHz, $CDCl_3$)**: δ** 3.69 (dd, *J* = 10.9, 9.2 Hz, 1H), 2.40 (s, 6H), 2.32 (dd, *J* = 12.8, 9.1 Hz, 1H), 1.84 (dd, *J* = 12.8, 11.2 Hz, 1H), 1.80 – 1.65 (m, 5H), 1.54 – 1.40 (m, 1H), 1.34 – 1.21 (m, 1H), 1.14 – 1.00 (m, 2H), 0.92 (d, *J* = 6.6 Hz, 3H); **^{13}C NMR** (101 MHz, $CDCl_3$)**: δ** 174.7, 83.7, 63.8, 41.8 (2C), 38.2, 35.4, 32.0, 32.0, 31.1, 31.1, 21.5.

(3*S,5*s**,8*R**)-8-(*tert*-Butyl)-3-(dimethylamino)-1-oxaspiro[4.5]decan-2-one (2i)**

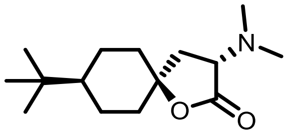

The title compound was prepared using general procedure **B** from alkene **1i**. Purification by column chromatography on silica gel (DCM to 40% DMA) afforded compound **2i** in 70% yield (31.0 mg) in 5:1 d.r. as an off-white solid. Only the major diastereoisomer was characterized.

^{1}H NMR (600 MHz, $CDCl_3$)**: δ** 3.75 – 3.69 (m, 1H), 2.43 (s, 6H), 2.38 – 2.32 (m, 1H), 1.88 – 1.72 (m, 6H), 1.69 – 1.59 (m, 1H), 1.20 – 1.01 (m, 3H), 0.87 (s, 9H); **^{13}C NMR** (151 MHz, $CDCl_3$)**: δ** 174.5, 84.0, 63.7, 46.9, 41.7 (C2), 39.1, 36.0, 31.7, 27.7 (C3), 24.8, 23.8, 20.7; **HRMS** (ESI^{+}): calculated for $C_{15}H_{27}O_2N$ $[M+H]^{+}$ *m/z*: 254.2120, found 254.2113; **IR** (neat, cm^{-1}): 2944, 2864, 2748, 1766, 1470, 1211.

(3*S,5*s**,8*R**)-3-(Dimethylamino)-8-phenyl-1-oxaspiro[4.5]decan-2-one**

(major diastereomer 2j)

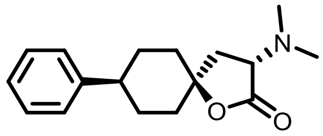

The title compound was prepared using general procedure **B** from alkene **1j**. Purification by column chromatography on silica gel (10% EEA to 80% EEA in heptane) afforded compound **2j** in 68% yield (37.1 mg) in 2.9:1 d.r. as an off-white solid. Diastereomers were separable.

^{1}H NMR (400 MHz, $CDCl_3$)**: δ** 7.33 – 7.27 (m, 2H), 7.24 – 7.17 (m, 3H), 3.75 (dd, *J* = 11.2, 9.1 Hz, 1H), 2.59 – 2.50 (m, 1H), 2.43 (s, 6H), 2.15 (dd, *J* = 12.8, 9.1 Hz, 1H), 2.03 (dd, *J* = 12.8, 11.3 Hz, 1H), 2.02 – 1.91 (m, 3H), 1.92 – 1.83 (m, 1H), 1.83 – 1.70 (m, 3H), 1.63 (td, *J* = 13.4, 4.1 Hz, 1H); **^{13}C NMR** (101 MHz, $CDCl_3$)**: δ** 174.8, 146.4, 128.6, 127.0, 126.4,

81.5, 63.7, 43.4, 41.9 (2C), 38.4, 37.4, 35.8, 29.9, 29.4; **HRMS** (ESI$^+$): calculated for $C_{17}H_{23}O_2N$ [M+H]$^+$ 274.1807; found 274.1801; **IR** (neat, cm^{-1}): 3048, 2979, 2932, 2840, 2749, 1762, 1401, 1277, 1123.

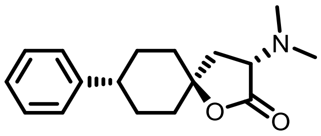

^{1}H NMR (400 MHz, $CDCl_3$)**:** **δ** 7.34 – 7.29 (m, 2H), 7.24 – 7.19 (m, 3H), 3.75 (dd, *J* = 10.8, 9.2 Hz, 1H), 2.60 (tt, *J* = 12.0, 3.5 Hz, 1H), 2.46 (dd, *J* = 12.8, 9.15 Hz, 1H), 2.44 (s, 6H), 2.06 – 1.95 (m, 4H), 1.94 – 1.82 (m, 3H), 1.70 – 1.55 (m, 2H); **^{13}C NMR** (101 MHz, $CDCl_3$)**:** **δ** 174.5, 145.5, 128.7, 126.8, 126.6, 83.2, 63.8, 42.8, 41.9, 38.8, 35.9, 31.8, 31.4, 30.2.

(3*S,5*s**,8*R**)-3-(Dimethylamino)-8-(trifluoromethyl)-1-oxaspiro[4.5]decan-2-one (major diastereomer 2k)**

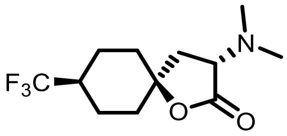

The title compound was prepared using general procedure **B** from alkene **1k**. Purification by column chromatography on silica gel (10% EEA to 80% EEA in heptane) afforded compound **2k** in 77% yield (40.8 mg) in 2.1:1 d.r. as a pale-yellow oil. Diastereomers were separable.

^{1}H NMR (600 MHz, $CDCl_3$)**:** **δ** 3.70 (dd, *J* = 10.8, 9.2 Hz, 1H), 2.41 (s, 6H), 2.33 (dd, *J* = 12.9, 9.1 Hz, 1H), 2.15 – 2.06 (m, 1H), 2.06 – 2.00 (m, 2H), 1.91 (dd, *J* = 12.9, 10.7 Hz, 1H), 1.90 – 1.81 (m, 3H), 1.70 (td, *J* = 12.9, 3.8 Hz, 1H), 1.57 – 1.45 (m, 2H); **^{13}C NMR** (151 MHz, $CDCl_3$)**:** **δ** 174.1, 127.5 (q, *J* = 278.6 Hz), 82.1, 63.7, 41.9 (2C), 40.1 (q, *J* = 27.0 Hz), 36.6, 34.0, 31.9, 22.2 (q, *J* = 2.1 Hz), 21.5 (q, *J* = 2.1 Hz); **^{19}F NMR** (376 MHz, $CDCl_3$)**:** **δ** -72.71 (d, *J* = 7.7 Hz); **HRMS** (ESI$^+$): calculated for $C_{12}H_{19}O_2NF_3$ [M+H]$^+$ 266.1368; found 266.1365; **IR** (neat, cm^{-1}): 2949, 2872, 2834, 2788, 1769, 1456, 1397, 1362, 1344, 1283, 1252, 1200, 1167, 1125, 1095, 1050, 1025, 981, 958, 926, 888, 719, 694.

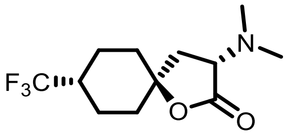

^{1}H NMR (400 MHz, $CDCl_3$)**:** **δ** 3.71 (dd, *J* = 11.0, 9.1 Hz, 1H), 2.41 (s, 6H), 2.12 (dd, *J* = 12.9, 9.1 Hz, 1H), 2.09 – 1.91 (m, 4H), 1.89 – 1.78 (m, 3H), 1.78 – 1.66 (m, 1H), 1.60 (dt, *J* = 13.1, 5.4 Hz, 1H), 1.48 (td, *J* = 13.7, 4.3 Hz, 1H); **^{13}C NMR** (151 MHz, $CDCl_3$)**:** **δ** 174.3, 127.4 (q, *J* = 278.5 Hz), 80.5, 63.6, 41.9 (2C), 41.0 (q, *J* = 27.1 Hz), 36.6 (2C), 35.5, 21.1, 20.7; **^{19}F NMR** (376 MHz, $CDCl_3$)**:** **δ** -73.64 (d, *J* = 8.2 Hz).

(3*S,5*S**,6*S**)-3-(Dimethylamino)-6-phenyl-1-oxaspiro[4.5]decan-2-one (2l)**

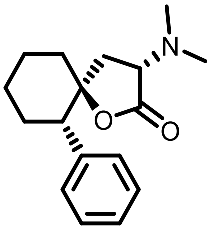

The title compound was prepared using general procedure B from alkene **1l**. The diastereomeric ratio was measured using ^{13}C NMR analysis of the crude sample (5:2:2:1). Purification by column chromatography on silica gel (20% DMA to 80% DMA in DCM) afforded compound **2l** in 66% yield (35.0 mg), in 3:1 d.r. as an opaque oil. Only the major diastereoisomer was characterized.

^{1}H NMR: (600 MHz, $CDCl_3$): **δ** 7.32 - 7.30 (m, 2H), 7.29 - 7.27 (m, 2H), 7.25 - 7.23 (m, 1H), 2.96 (dd, *J* = 12.9, 3.5 Hz, 1H), 2.36 - 2.28 (m, 1H), 2.18 - 2.14 (m, 6H), 2.09 - 2.05 (m, 1H), 2.01 - 1.93 (m, 2H), 1.93 - 1.84 (m, 4H), 1.75 - 1.66 (m, 1H), 1.46 - 1.36 (m, 2H); **^{13}C NMR** (151 MHz, $CDCl_3$): **δ** 174.5, 139.2, 128.9 (2C), 128.5 (2C), 127.4, 85.7, 63.8, 50.3, 41.1 (2C), 40.9, 34.3, 28.7, 28.4, 25.6; **HRMS** (ESI$^+$): calculated for $C_{17}H_{23}O_2N$ $[M+H]^+$ *m/z*: 274.1807, found: 274.1804; **IR** (neat, cm^{-1}): 2934, 2861, 2831, 1767, 1450, 1276, 1167, 922, 703.

(3*S,5S*,6*S**)-6-Cyclohexyl-3-(dimethylamino)-1-oxaspiro[4.5]decan-2-one (2m)**

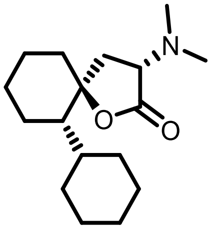

The title compound was prepared using general procedure **B** from alkene **1m**. The diastereomeric ratio was measured using ^{13}C NMR analysis of the crude sample (3.6:2.1:2:1). Purification by column chromatography on silica gel (10% EEA to 80% EEA in heptane) afforded compound **2m** in 54% yield (30.1 mg) in 4.8:3:1.2:1 d.r. as a pale-yellow oil. Diastereomers were separable.

^{1}H NMR (400 MHz, $CDCl_3$)**: δ** 3.73 (dd, *J* = 11.3, 9.2 Hz, 1H), 2.42 (s, 6H), 2.17 (dd, *J* = 12.8, 11.3 Hz, 1H), 2.10 (dd, *J* = 12.9, 9.2 Hz, 1H), 1.80 – 1.65 (m, 8H), 1.65 – 1.47 (m, 4H), 1.47 – 1.30 (m, 1H), 1.27 – 1.00 (m, 7H); **^{13}C NMR** (101 MHz, $CDCl_3$) δ 175.0, 87.2, 63.8, 50.6, 41.7, 37.8, 36.5, 35.0, 30.7, 27.2, 27.0, 26.6, 25.7 (2C), 25.2, 24.1; **HRMS** (ESI$^+$): calculated for $C_{17}H_{30}O_2N$ $[M+H]^+$ 280.2277; found 280.2273; **IR** (neat, cm^{-1}): 2928, 2855, 2784, 1765, 1452, 1275, 1261, 1244, 1215, 1178, 1129, 1040, 1008, 911, 750, 732.

Minor diastereomer*

^{1}**H NMR** (400 MHz, $CDCl_3$)**: δ** 3.64 (t, *J* = 10.1 Hz, 1H), 2.45 (dd, *J* = 13.8, 10.4 Hz, 1H), 2.40 (s, 6H), 1.83 – 1.70 (m, 7H), 1.67 – 1.54 (m, 3H), 1.51 – 1.44 (m, 2H), 1.35 – 1.24 (m, 4H), 1.24 – 1.04 (m, 5H); ^{13}**C NMR** (101 MHz, $CDCl_3$)**: δ** 175.1, 87.4, 64.8, 51.2, 42.3, 41.7, 36.3, 34.5, 29.7, 29.3, 27.08, 27.07, 26.5, 26.0, 25.4, 23.1.

For the other two minor diastereomers only the following signals are unambiguously detectable: ^{1}**H NMR** (400 MHz, $CDCl_3$)**: δ** 3.77 (dd, *J* = 11.1, 9.5 Hz, 1H**), 3.69 (dd, *J* = 10.5, 9.5 Hz, 1H***), 2.42 (s, 6H**), 2.39 (s, 6H***).

3-(Dimethylamino)-1,9,13-trioxadispiro[4.2.5^8.2^5]pentadecan-2-one (2n)

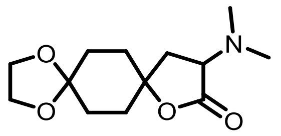

The title compound was prepared using general procedure **B** from alkene **1n**. Purification by column chromatography on silica gel (20% DMA to 40% DMA in DCM) afforded compound **2n** in 72% yield (35.0 mg) as a colorless oil.

^{1}**H NMR** (600 MHz, $CDCl_3$)**: δ** 3.98 - 3.90 (m, 4H), 3.74 - 3.67 (m, 1H), 2.41 (s, 6H), 2.21 - 2.13 (m, 1H), 2.01 - 1.97 (m, 1H), 1.97 - 1.92 (m, 1H), 1.92 - 1.85 (m, 4H), 1.83 - 1.74 (m, 1H), 1.68 - 1.63 (m, 2H); ^{13}**C NMR** (151 MHz, $CDCl_3$)**: δ** 174.6, 107.8, 81.4, 64.6, 64.5, 63.7, 41.9 (C2), 36., 34.4, 34.3, 31.2, 30.9; **HRMS** (ESI^+): calculated for $C_{13}H_{21}O_4N$ $[M+Na]^+$ *m/z*: 278.1368, found 278.1361; **IR** (neat, cm^{-1})**:** 2938, 2876, 2832, 2783, 1763, 1447, 1144.

(3*S,5*s**,8*R**)-3-(Dimethylamino)-8-hydroxy-1-oxaspiro[4.5]decan-2-one (2o)**

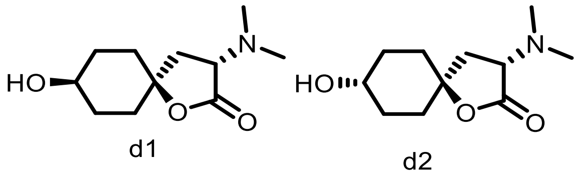

The title compound was prepared using general procedure **B** from alkene **1o** (or commercially available **1o'**). Purification by column chromatography on silica gel (20% EEA in heptane to 100% EEA) afforded compound **2o** in 45% yield (19.1 mg) as a 1.2:1 mixture of inseparable diastereomers, as a colorless oil.

^{1}**H NMR** (600 MHz, $CDCl_3$)**: δ** 3.98 – 3.94 (m, 1H, d1), 3.71 (t, *J* = 9.3 Hz, 1H, d1), 3.69 (t, *J* = 9.4 Hz, 1H, d2), 3.70 – 3.66 (m, 1H, d2), 2.39 (s, 6H, d1), 2.39 (s, 6H, d2), 2.22 (dd, *J* =

12.9, 9.1 Hz, 1H, d1), 2.11 (dd, J = 13.0, 8.9 Hz, 1H, d1), 1.97 – 1.87 (m, 2H, 2H, d2), 1.96 (dd, J = 12.8, 11.1 Hz, 1H, d2), 1.93 (dd, J = 12.8, 11.2 Hz, 1H, d1), 1.86 – 1.79 (m, 2H, 2H, d2), 1.78 – 1.55 (m, 3H, 4H, d2), 1.54 – 1.47 (m, 1H, d1); **^{13}C NMR** (151 MHz, $CDCl_3$)**: δ** 174.7 (d2), 174.5 (d1), 82.4 (d2), 81.2 (d1), 68.7 (d1), 66.3 (d2), 63.7 (d2), 63.7 (d1), 41.8 (C2, d1, d2), 36.0 (d1), 34.5 (d2), 34.4 (d1), 34.4 (d2), 33.4 (d2), 31.8 (d1), 31.2 (d1), 30.9 (2C, d1, d2), 29.9 (d1); **HRMS** (ESI$^+$): calculated for $C_{11}H_{20}O_3N$ $[M+H]^+$ 214.1443; found 214.1438, **IR** (neat, cm^{-1}): 3382, 2937, 2867, 2788, 1760, 1446, 1374, 1203, 1125, 1069, 1015, 991, 924.

(3*S,5S*,6*R**)-3-(Dimethylamino)-6-ethoxy-1-oxaspiro[4.5]decan-2-one (major diastereomer 2p)**

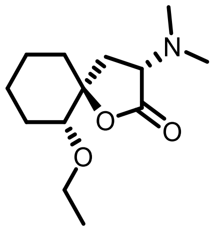

The title compound was prepared using general procedure **B** from alkene **1p**. The diastereomeric ratio was measured using ^{13}C NMR analysis of the crude sample (3.2:2.5:1.2:1). Purification by column chromatography on silica gel (10% EEA in heptane to 100% EEA) afforded compound **2p** in 58% yield (19.1 mg) as a 3.2:2.5:1.2:1 mixture of partially separable diastereomers, as a colorless oil.

^{1}H NMR (400 MHz, $CDCl_3$)**: δ** 3.94 (t, J = 9.8 Hz, 1H), 3.61 (dq, J = 9.2, 7.0 Hz, 1H), 3.40 (dq, J = 9.3, 7.0 Hz, 1H), 3.25 (dd, J = 10.6, 4.7 Hz, 1H), 2.49 (dd, J = 13.0, 9.9 Hz, 1H), 2.38 (s, 6H), 2.08 – 2.00 (m, 1H), 1.79 – 1.66 (m, 4H), 1.70 (dd, J = 13.0, 9.8 Hz, 1H), 1.33 – 1.21 (m, 3H), 1.13 (t, J = 7.0 Hz, 3H); **^{13}C NMR** (101 MHz, $CDCl_3$)**: δ** 175.4, 85.8, 82.1, 65.7, 64.7, 41.4 (2C), 37.8, 29.1, 29.0, 23.7, 22.2, 15.6**; HRMS (**ESI$^+$**)**: calculated for $C_{13}H_{24}O_3N$ $[M+H]^+$ 242.1756; found 242.1752; **IR** (neat, cm^{-1}): 2971, 2937, 2900, 2865, 2786, 1768, 1468, 1454, 1276, 1263, 1244, 1218, 1199, 1170, 1155, 1103, 1074, 1052, 1027, 1013, 979, 969, 942, 913, 751, 733.

Minor diastereomer:

^{1}H NMR (400 MHz, $CDCl_3$)**: δ** 3.73 (dd, J = 10.9, 9.8 Hz, 1H), 3.62 (dq, J = 9.3, 7.0 Hz, 1H), 3.53 (dq, J = 9.3, 7.0 Hz, 1H), 3.31 (dd, J = 10.3, 4.3 Hz, 1H), 2.42 (s, 6H), 1.97 (dd, J = 12.8, 9.8 Hz, 1H), 1.94 – 1.88 (m, 1H), 1.80 – 1.66 (m, 4H), 1.64 – 1.53 (m, 1H), 1.37 – 1.21 (m,

3H), 1.13 (t, *J* = 7.0 Hz, 3H); **^{13}C NMR** (101 MHz, $CDCl_3$)**: δ** 174.8, 85.9, 80.7, 66.2, 63.4, 41.6 (2C), 35.5, 28.8, 25.6, 23.0, 22.9, 15.8.

Ethyl (3*S,5*s**,8*R**)-3-(dimethylamino)-2-oxo-1-oxaspiro[4.5]decane-8-carboxylate (2q)**

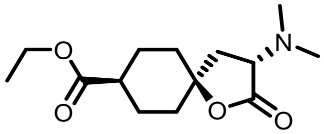

The title compound was prepared using general procedure **B** from alkene **1q** using EtOAc at 23 °C. Purification by column chromatography on silica gel (10% EEA in heptane to 100% EEA) afforded compound **2q** in 63% yield (33.9 mg) as a 1:1 mixture of separable diastereomers, as a colorless oil.

^{1}H NMR (400 MHz, $CDCl_3$)**: δ** 4.12 (q, *J* = 7.1 Hz, 2H), 3.70 (dd, *J* = 10.8, 9.2 Hz, 1H), 2.40 (s, 6H), 2.34 – 2.25 (m, 1H), 2.12 (dd, *J* = 12.7, 9.1 Hz, 1H), 2.01 – 1.80 (m, 7H), 1.65 – 1.55 (m, 1H), 1.53 – 1.44 (m, 1H), 1.24 (t, *J* = 7.1 Hz, 3H); **^{13}C NMR** (101 MHz, $CDCl_3$)**: δ** 174.8, 174.4, 81.1, 63.6, 60.6, 42.0, 41.9, 37.2, 36.0, 35.4, 24.8, 24.4, 14.3; **HRMS** (ESI$^+$): calculated for $C_{14}H_{24}O_4N$ $[M+H]^+$ 270.1705; found 270.1701; **IR** (neat, cm^{-1}): 2940, 2868, 2833, 2786, 1764, 1725, 1455, 1367, 1329, 1295, 1199, 1127, 1019, 980, 958, 924, 863

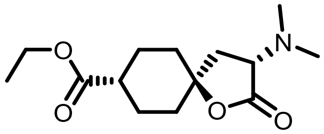

^{1}H NMR (400 MHz, $CDCl_3$)**: δ** 4.13 (q, *J* = 7.1 Hz, 2H), 3.69 (t, *J* = 10.0 Hz, 1H), 2.49 – 2.41 (m, 1H), 2.40 (s, 6H), 2.28 (dd, *J* = 13.1, 3.9 Hz, 1H), 2.05 – 1.97 (m, 2H), 1.96 – 1.84 (m, 1H), 1.84 – 1.67 (m, 6H), 1.25 (t, *J* = 7.1 Hz, 3H); **^{13}C NMR** (101 MHz, $CDCl_3$)**: δ** 174.9, 174.4, 82.4, 63.7, 60.6, 41.9 (2C), 40.4, 36.6, 34.4, 33.1, 25.1, 24.7, 14.4.

***N*-((3*S**,5*s**,8*R**)-3-(Dimethylamino)-2-oxo-1-oxaspiro[4.5]decan-8-yl)benzamide (2r)**

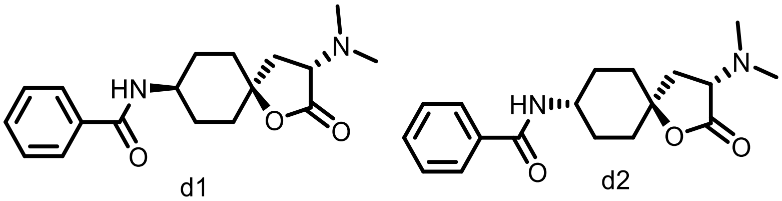

The title compound was prepared using general procedure **B** from alkene **1r**. Purification by column chromatography on silica gel (20% EEA in heptane to 100% EEA) afforded compound **2r** in 68% yield (43.0 mg) as a 1.4:1 mixture of inseparable diastereomers, as a colorless oil.

^{1}H NMR (600 MHz, $CDCl_3$)**: δ** 7.75 – 7.73 (m, 2H, 2H, d1), 7.50 – 7.47 (m, 1H, d2), 7.49 – 7.46 (m, 1H, d1), 7.43 – 7.39 (m, 2H, 2H, d2), 6.17 (d, *J* = 8.2 Hz, 1H, d1), 6.16 (d, *J* = 7.5 Hz, 1H, d2), 4.10 – 3.99 (m, 1H, 1H, d2), 3.71 (dd, *J* = 10.8, 9.2 Hz, 1H, d2), 3.70 (dd, *J* = 11.0, 9.1 Hz, 1H, d1), 2.40 (s, 6H, d2), 2.39 (s, 6H, d1), 2.33 (dd, *J* = 13.0, 9.1 Hz, 1H, d2), 2.16 – 2.10 (m, 1H, d1), 2.13 (dd, *J* = 12.8, 9.0 Hz, 1H, d1), 2.00 (dd, *J* = 12.7, 11.2 Hz, 1H, d1), 1.98 – 1.87 (m, 3H, 3H, d2), 1.86 – 1.79 (m, 2H, d2), 1.79 – 1.70 (m, 1H, 3H, d2), 1.67 – 1.58 (m, 2H, 1H, d2), 1.58 – 1.47 (m, 1H, d1); **^{13}C NMR** (151 MHz, $CDCl_3$)**: δ** 174.5 (d1), 174.2 (d2), 167.2 (d2), 167.0 (d1), 134.7 (d2), 134.7 (d1), 131.6 (d2), 131.5 (d1), 128.7 (C2, d1, d2), 127.0 (C2, d1, d2), 82.1 (d2), 80.9 (d1), 63.7 (d1), 63.6 (d2), 47.5 (c1), 46.9 (c2), 41.9 (2C, d1), 41.8 (2C, d2), 36.9 (d1), 36.2 (d2), 35.7 (cd), 35.0 (d1), 33.8 (d2), 32.8 (d2), 28.9 (d2), 28.7 (d1), 28.3 (d2), 28.2 (d1); **HRMS** (ESI^+): calculated for $C_{18}H_{25}O_3N_2$ $[M+H]^+$ 317.1865; found 317.1856; **IR** (neat, cm^{-1}): 3313, 3059, 2937, 2868, 2833, 2786, 1760, 1635, 1531, 1489, 1447, 1335, 1309, 1273, 1206, 1125, 1095, 1077, 1046, 1021, 954, 929, 802, 713, 696.

3-((3*S,5*S**,6*S**)-3-(Dimethylamino)-2-oxo-1-oxaspiro[4.5]decan-6-yl)propanenitrile (2s)**

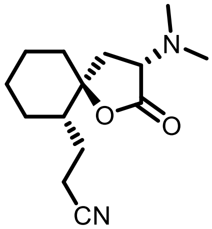

The title compound was prepared using general procedure **B** from alkene **1s** at 75 °C. Purification by column chromatography on silica gel (20% EEA in heptane to 100% EEA) afforded compound **2s** in 61% yield (30.5 mg) as a 4.2:2.1:1.3:1 mixture of inseparable diastereomers, as a colorless oil.

^{1}H NMR (600 MHz, $CDCl_3$)**: δ** 3.71 (dd, *J* = 11.4, 9.1 Hz, 1H, d1), 3.64 (dd, *J* = 10.5, 9.0 Hz, 1H, d2), 2.41 (s, 6H, d1), 2.39 (s, 6H, d2), 2.44 – 2.28 (m, 2H, 2H, d2), 2.23 (dd, *J* = 14.0, 10.6 Hz, 1H, d2), 2.11 (dd, *J* = 12.9, 9.1 Hz, 1H, d1), 1.93 (dd, *J* = 12.8, 11.5 Hz, 1H, d1), 1.91 – 1.85 (m, 1H, 2H, d2), 1.83 (dd, *J* = 14.1, 9.0 Hz, 1H, d2), 1.80 – 1.58 (m, 5H, 5H, d2), 1.49 – 1.39 (m, 2H, 1H, d2), 1.39 – 1.27 (m, 2H, 2H, d2), 1.17 – 1.04 (m, 1H, 1H, d2). For the other two minor diastereomers only the following signals are unambiguously detectable: 3.71 (dd, *J* = 12.6, 9.6 Hz, 1H, d3), 3.69 (dd, *J* = 12.0, 9.5 Hz, 1H, d4), 2.40 (s, 6H, d3), 2.38 (s, 6H, d4), 2.18 (dd, *J* = 12.8, 11.3 Hz, 1H, d3); **^{13}C NMR** (151 MHz, $CDCl_3$)**: δ** 174.2 (d2), 174.0 (d1), 119.3 (d1), 119.1 (d2), 85.8 (d2), 84.9 (d1), 64.4 (d1), 63.2 (d2), 44.6 (d1), 44.5

(d2), 41.6 (2C, d1), 41.5 (2C, d2), 39.6 (d2), 36.0 (d1), 28.7 (d1), 27.4 (d2), 27.1 (d1), 26.7 (d2), 24.8 (d1), 24.7 (d2), 24.5 (d1), 24.1 (d2), 23.1 (d1), 22.4 (d2), 15.6 (d1), 15.2 (d2). For the other two minor diastereomers only the following signals are unambiguously detectable: 174.5, 174.2, 119.3, 119.2, 84.6, 83.7, 63.9, 43.5, 42.9, 41.8, 41.5, 33.8, 31.7, 29.6, 26.9, 26.3, 23.6, 22.0, 21.9, 15.3; **HRMS** (ESI$^+$): calculated for $C_{14}H_{23}O_2N_2$ [M+H]$^+$ 251.1760; found 251.1753; **IR** (neat, cm^{-1}): 2937, 2865, 2832, 2786, 1764, 1453, 1274, 1210, 1149, 1127, 1096, 1073, 1044, 1011, 977, 964, 946, 739.

(2*S,4*S**,4a'*S**,8a'*R**)-4-(Dimethylamino)decahydro-2'*H*,5*H*-spiro[furan-2,1'-naphthalen]-5-one (2t)**

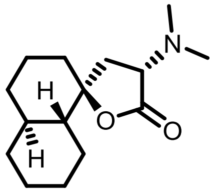

The title compound was prepared using general procedure **B** from alkene **1t**. Purification by column chromatography on silica gel (10% EEA to 80% EEA in heptane) afforded compound **2t** in 80% yield (40.3 mg) as a 5.1:2:1 diastereomeric mixture, as a pale-yellow oil (the fourth diastereomer was not detected). Diastereomers were partially separable.

^{1}H NMR (400 MHz, $CDCl_3$)**: δ** 3.68 (t, *J* = 10.1 Hz, 1H), 2.38 (s, 6H), 2.31 (dd, *J* = 13.6, 10.5 Hz, 1H), 1.80 – 1.68 (m, 6H), 1.66 – 1.54 (m, 2H), 1.36 – 1.15 (m, 5H), 1.15 – 1.06 (m, 1H), 1.06 – 0.91 (m, 2H), 0.85 (qd, *J* = 12.0, 3.5 Hz, 1H); **^{13}C NMR** (101 MHz, $CDCl_3$)**: δ** 175.1, 86.5, 65.0, 50.5, 41.5 (2C), 40.8, 39.2, 33.9, 33.3, 29.3, 26.1, 25.9, 25.6, 21.9; **HRMS** (ESI$^+$): calculated for $C_{15}H_{26}O_2N$ [M+H]$^+$ 280.2277; found 280.2272; **IR** (neat, cm^{-1}): 2928, 2856, 2784, 1765, 1452, 1275, 1261, 1244, 1215, 1178, 1130, 1041, 1008, 911, 750, 732.

Minor diastereomer (d2)

^{1}H NMR (400 MHz, $CDCl_3$)**: δ** 3.73 (dd, *J* = 11.1, 9.4 Hz, 1H), 2.16 (dd, *J* = 12.8, 11.3 Hz, 1H), 2.04 (dd, *J* = 13.0, 9.4 Hz, 1H), 1.85 – 1.73 (m, 3H), 1.73 – 1.54 (m, 6H), 1.51 – 1.36 (m, 2H), 1.34 – 1.13 (m, 3H), 1.13 – 0.93 (m, 3H); **^{13}C NMR** (101 MHz, $CDCl_3$)**: δ** 174.8, 85.8, 63.7, 49.8, 41.8, 38.2, 37.5, 34.3, 33.3, 25.9, 25.3, 25.0, 23.0.

Minor diastereomer**

For the other minor diastereomer only the following signals are unambiguously detectable: **^{1}H NMR** (400 MHz, $CDCl_3$)**: δ** 3.74 (dd, *J* = 11.7, 9.1 Hz, 1H), 2.41 (s, 8H); **^{13}C NMR** (101

MHz, $CDCl_3$): **δ** 175.1, 84.9, 64.5, 51.4, 41.8, 40.9, 37.3, 34.3, 34.0, 33.6, 26.6, 26.0, 25.5, 22.0.

(2*S,4*S**,4a'*R**,8a'*R**)-4-(Dimethylamino)decahydro-2'*H*,5*H*-spiro[furan-2,1'-naphthalen]-5-one (2u)**

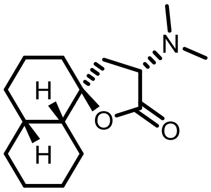

The title compound was prepared using general procedure **B** from alkene **1u**. Purification by column chromatography on silica gel (10% EEA to 80% EEA in heptane) afforded compound **2u** in 76% yield (38.2 mg) as a 4.4:1.6:1.3:1 mixture of partially separable diastereomers, as a pale-yellow oil.

^{1}H NMR (400 MHz, $CDCl_3$): **δ** 3.69 (dd, J = 11.7, 8.8 Hz, 1H), 2.41 (s, 6H), 1.99 (dd, J = 12.5, 8.7 Hz, 1H), 1.90 (t, J = 12.2 Hz, 1H), 1.84 – 1.71 (m, 2H), 1.70 – 1.54 (m, 5H), 1.52 – 1.38 (m, 5H), 1.36 – 1.16 (m, 4H); **^{13}C NMR** (101 MHz, $CDCl_3$): **δ** 174.7, 85.8, 63.7, 47.2, 41.8 (2C), 34.4, 32.2, 31.7, 31.4, 26.7, 24.3, 22.1, 21.7, 21.0; **HRMS** (ESI$^+$): calculated for $C_{15}H_{26}O_2N$ $[M+H]^+$ 252.1964; found 252.1954; **IR** (neat, cm^{-1}): 2927, 2863, 2785, 1766, 1451, 1260, 1245, 1222, 1200, 1152, 1134, 1101, 1078, 1041, 1017, 983, 944, 757.

For the other minor diastereomers only the following signals are unambiguously detectable: **^{1}H NMR** (400 MHz, $CDCl_3$): **δ** 3.69 (dd, J = 10.9, 9.2 Hz, 1H), 3.59 (dd, J = 10.7, 8.9 Hz, 1H), 2.403 (s, 6H), 2.398 (s, 6H), 2.39 (s, 6H); **^{13}C NMR** (101 MHz, $CDCl_3$): **δ** 174.7, 86.7, 86.3, 86.0, 63.63, 63.59, 63.55, 45.3, 44.8, 42.0, 34.6, 33.6, 33.5, 33.4, 33.3, 32.4, 31.7, 31.63, 31.61, 26.8, 26.2, 26.1, 24.1, 23.9, 23.4, 23.2, 22.3, 22.0, 21.6, 21.3, 21.0, 20.7.

(3*S*,5S,6*S*,9*R*)-3-(Dimethylamino)-6-isopropyl-9-methyl-1-oxaspiro[4.5]decan-2-one (2v)

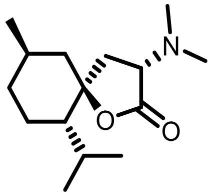

The title compound was prepared using general procedure **B** from alkene **1v**. Purification by multiple column chromatography on silica gel (2% to 30% EEA in heptane) afforded compound **2v** in 55% yield (28.0 mg) as a 3:1 mixture of partially separable diastereomers, as a colorless oil. The other minor diastereomers were not detectable.

^{1}H NMR (700 MHz, $CDCl_3$)**: δ** 3.72 (dd, *J* = 11.5, 9.0 Hz, 1H, d2), 3.69 (t, *J* = 10.2 Hz, 1H, d1), 2.43 (dd, *J* = 13.8, 10.4 Hz, 1H, d1), 2.42 (s, 6H, d2), 2.40 (s, 6H, d1), 2.19 (dd, *J* = 12.9, 11.4 Hz, 1H, d2), 2.09 (dd, *J* = 12.8, 9.0 Hz, 1H, d2), 1.86 – 1.81 (m, 1H, d2), 1.79 (dd, *J* = 13.8, 9.9 Hz, 1H, d1), 1.76 – 1.70 (m, 3H, 4H, d2), 1.70 – 1.67 (m, 1H, d1), 1.60 – 1.52 (m, 1H, d2), 1.50 (dt, *J* = 13.2, 3.5 Hz, 1H, d2), 1.50 – 1.44 (m, 2H, d1), 1.31 – 1.25 (m, 1H, d1), 1.26 (t, *J* = 12.7 Hz, 1H, d2), 1.19 – 1.12 (m, 1H, d1), 1.06 (ddd, *J* = 27.4, 13.8, 3.6 Hz, 1H, d2), 0.96 (d, *J* = 6.9 Hz, 3H, d2), 0.95 – 0.90 (m, 1H, d1), 0.94 (d, *J* = 6.0 Hz, 3H, d1), 0.94 (d, *J* = 6.5 Hz, 3H, d2), 0.93 (d, *J* = 6.8 Hz, 3H, d1), 0.91 (d, *J* = 6.9 Hz, 3H, d2), 0.87 (d, *J* = 6.9 Hz, 3H, d1); **^{13}C NMR** (176 MHz, $CDCl_3$)**: δ** 175.1 (d1), 174.8 (d2), 87.3 (d1), 87.0 (d2), 64.8 (d1), 63.8 (d2), 50.7 (d1), 50.0 (d2), 50.3 (d1), 46.3 (d2), 41.9 (2C, d2), 41.7 (2C, d1), 34.6 (d1), 34.4 (d2), 30.9 (d2), 29.9 (d1), 29.2 (d1), 26.0 (d2), 25.9 (d2), 25.4 (d1), 24.7 (d2), 24.6 (d2), 24.5 (d1), 23.9 (d1), 22.2 (d1), 22.0 (d2), 20.0 (d2), 18.6 (d1); **HRMS** (ESI^+): calculated for $C_{15}H_{28}O_2N$ $[M+H]^+$ 254.2120; found 254.2112; **IR** (neat, cm^{-1}): 2957, 2926, 2870, 2784, 1768, 1454, 1366, 1269, 1231, 1209, 1174, 1148, 1094, 1072, 1046, 1011, 986, 953.

(3*S,4*S**)-3-(Dimethylamino)-4-methyl-1-oxaspiro[4.5]decan-2-one (2w)**

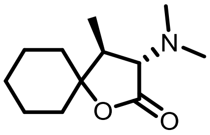

The title compound was prepared using general procedure **B** from alkene **1w**. Purification by column chromatography on silica gel (20% DMA in DCM to 30% DMA) afforded compound **2w** in 65% yield (24.2 mg) in 4:1 d.r. as an opaque oil. Only the major diastereoisomer was characterized.

^{1}H NMR: (600 MHz, $CDCl_3$)**: δ** 3.27 (d, *J* = 12.0 Hz, 1H), 2.50 (s, 6H), 2.16 - 2.05 (m, 1H), 1.76 - 1.68 (m, 2H), 1.67 - 1.54 (m, 6H), 1.50 - 1.39 (m, 1H), 1.36 - 1.13 (m, 2H), 1.13 - 1.06 (m, 4H); **^{13}C NMR** (151 MHz, $CDCl_3$)**: δ** 174.4, 84.9, 69.4, 42.8, 41.6 (2C), 36.4, 31.4, 25.5, 22.3, 21.4, 13.3; **HRMS** (ESI^+): calculated for $C_{12}H_{22}O_2N$ $[M+H]^+$ *m/z*: 212.1651, found: 212.1645; **IR** (neat, cm^{-1}): 2936, 2855, 2792, 1766, 1450, 1161.

(3*S,5*S**,6*S**)-6-Allyl-3-(dimethylamino)-1-oxaspiro[4.5]decan-2-one (2x)**

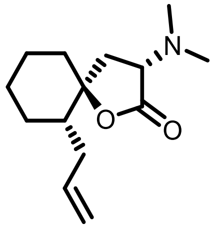

The title compound was prepared using general procedure **B** from alkene **1x**. Purification by column chromatography on silica gel (10% to 80% EEA in heptane) afforded compound **2x** in 66% yield (30.5 mg) as a 6.2:4:1:1 mixture of partially separable diastereomers, as a colorless oil (analytical sample contained diastereomers).

^{1}H NMR (400 MHz, $CDCl_3$)**: δ** 5.79 – 5.63 (m, 1H), 5.02 (d, *J* = 13.3 Hz, 2H), 3.72 (dd, *J* = 11.3, 9.2 Hz, 1H), 2.41 (s, 6H), 2.35 – 2.24 (m, 1H), 2.07 (dd, *J* = 12.8, 9.2 Hz, 1H), 1.90 – 1.57 (m, 7H), 1.50 – 1.35 (m, 1H), 1.35 – 1.19 (m, 2H), 1.04 (ddd, *J* = 13.7, 10.8, 3.5 Hz, 1H); ^{13}C NMR (101 MHz, $CDCl_3$)**: δ** 174.6, 136.6, 116.8, 85.8, 63.6, 44.9, 41.8 (2C), 36.3, 33.5, 27.2, 26.8, 24.0, 23.6; **HRMS** (ESI$^+$): calculated for $C_{14}H_{24}O_2N$ $[M+H]^+$ 238.1807; found 238.1803; **IR** (neat, cm^{-1}): 3076, 2973, 2935, 2863, 2835, 2784, 1767, 1452, 1274, 1211, 1144, 1122, 1095, 1077, 1045, 1012, 977, 963, 941, 912.

Minor diastereomer*

^{1}H NMR (400 MHz, $CDCl_3$)**: δ** 5.75 – 5.62 (m, 1H), 5.03 (dd, *J* = 8.3, 6.6 Hz, 2H), 3.66 (dd, *J* = 10.2, 9.7 Hz, 1H), 2.40 (s, 6H), 2.29 (dd, *J* = 13.8, 10.5 Hz, 1H), 2.23 – 2.12 (m, 1H), 1.92 – 1.84 (m, 1H), 1.79 – 1.63 (m, 6H), 1.37 – 1.16 (m, 3H), 1.07 – 0.95 (m, 1H); **^{13}C NMR** (101 MHz, $CDCl_3$)**: δ** 174.9, 136.2, 116.9, 86.6, 64.8, 45.1, 41.7 (2C), 40.2, 34.0, 28.7, 28.2, 24.8, 23.0.

Minor diastereomers**

For the other minor diastereomers only the following signals are unambiguously detectable: **^{1}H NMR** (400 MHz, $CDCl_3$)**: δ** 3.73 (dd, *J* = 11.5, 9.3 Hz, 1H**), 2.40 (s, 6H**), 2.39 (s, 6H***).; **^{13}C NMR** (101 MHz, $CDCl_3$)**: δ** 137.0, 136.5, 116.5, 85.1, 84.4, 64.2, 63.5, 45.2, 43.9, 41.9, 34.1, 27.3, 26.9, 22.4, 22.2.

(3*S*,5*S*)-3-(Dimethylamino)-5-methyl-5-((*S*)-4-methylcyclohex-3-en-1-yl)dihydrofuran-2(3*H*)-one (diastereomer 1 – 2y)

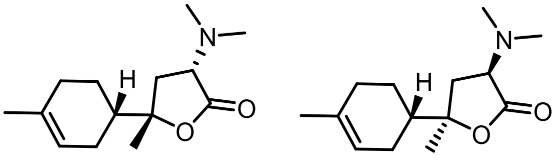

d1 d2

The title compound was prepared using general procedure **B** from (*S*)-(–)-limonene. The diastereomeric ratio was measured using ^{1}H NMR analysis of the crude sample (1.5:1.5:1:1). Purification by column chromatography on silica gel (10% EEA in heptane to 80% EEA) afforded compound **2y** in 86% yield (40.7 mg) as a 1.5:1.5:1:1 mixture of partially separable diastereomers, as a yellow oil. The relative configuration of diastereomers was determined by NOESY experiment.

^{1}H NMR (700 MHz, $CDCl_3$)**: δ** 5.38 (m, 1H), 3.76 (dd, *J* = 11.4, 6.4 Hz, 1H, d1), 3.75 (dd, 11.6, 6.4 Hz, 1H, d2), 2.41 (s, 6H), 2.13 – 1.89 (m, 6H), 1.81 – 1.75 (m, 2H), 1.65 (s, 3H), 1.37 – 1.24 (m, 1H), 1.32 (s, 3H, d1), 1.30 (s, 3H, d2). For the other two minor diastereomers, only the following signals are unambiguously detectable: 3.679 (t, *J* = 9.8 Hz, 1H), 3.673 (t, *J* = 10.0 Hz, 1H), 2.401 (s, 3H), 2.399 (s, 3H), 1.64 (s, 3H), 1.393 (s, 3H), 1.387 (s, 3H). **^{13}C NMR** (176 MHz, $CDCl_3$) **δ** 174.68, 174.64, 134.6, 134.2, 119.8, 119.5, 85.4, 85.3, 63.9, 63.8, 44.4, 44.1, 41.85, 41.83, 32.8, 31.8, 30.48, 30.42, 26.3, 26.1, 23.7, 23.47, 23.44, 23.41, 22.1. For the other two minor diastereomers, only the following signals are unambiguously detectable: 174.8, 134.4, 134.0, 119.7, 119.4, 86.0, 64.5, 64.4, 43.0, 42.0, 41.7, 41.6, 32.8, 31.9, 30.4, 30.3, 26.9, 26.3, 24.4, 24.0, 23.5, 23.2.; **HRMS** (ESI^{+}): calculated for $C_{14}H_{24}ON_2$ $[M+H]^{+}$ 238.1802; found 238.1798; **IR** (neat, cm^{-1}): 2989, 2942, 2852, 1747, 1431, 1301, 1163, 1098, 1055, 922, 910.

4. General procedure C for the three-component formation of aminolactones from alkenes

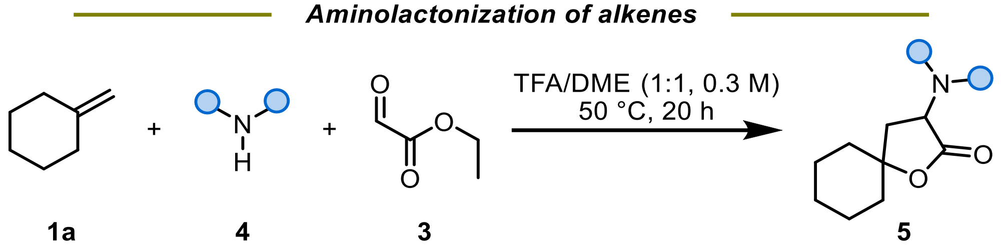

To a flame dried-Schlenk flask charged with ethyl glyoxylate **3** (4.0 eq., 0.8 mmol, in 50% toluene) at 0 °C were added the desired amine **4** (4.0 eq., 0.8 mmol) and trifluoroacetic acid (0.33 mL). After 5 min, methylenecyclohexane **1** (1.0 eq., 0.2 mmol) was added as a solution in anhydrous 1,2-dimethoxyethane (0.33 mL). The reaction mixture was heated to 50 °C and vigorously stirred for 20 h (unless otherwise stated), after which it was cooled to 0 °C. Then, an aqueous solution of NaOH (1.0 M) was added until a pH >7 was reached. The resulting biphasic mixture was separated and the aqueous phase was extracted with DCM (3 × 50 mL/mmol). The combined organic phases were then dried over anhydrous K_2CO_3 and filtered. The filtrate was concentrated under reduced pressure to afford the crude product, which was purified by flash column chromatography on silica gel with DCM/DMA (DMA = solvent mixture of dichloromethane, methanol, aq. NH_4OH in the ratio 9:1:0.15) or heptane/EEA (EEA = solvent mixture of ethyl acetate, ethanol, aq. NH_4OH in the ratio 3:1:0.08) to afford the analytically pure desired products **5**.

4.1 Characterization of the aminolactones formed using procedure C

3-(Dibenzylamino)-1-oxaspiro[4.5]decan-2-one (5a)

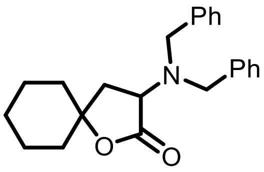

The title compound was prepared from dibenzylamine using general procedure **C**. Purification by column chromatography on silica gel (10% DMA in DCM to 60% DMA) afforded compound **5a** in 63% yield (39.2 mg) as a colourless oil.

^{1}H NMR (600 MHz, $CDCl_3$)**: δ** 7.42 – 7.40 (m, 4H), 7.33 – 7.31 (t, *J* = 7.5 Hz, 4H), 7.24 – 7.21 (m, 2H), 3.92 (d, *J* = 13.8 Hz, 2H), 3.91 – 3.85 (m, 1H), 3.67 (d, *J* = 13.8 Hz, 2H), 2.23 – 2.20 (m, 1H), 1.95 – 1.87 (m, 1H), 1.77 – 1.68 (m, 2H), 1.64 – 1.60 (m, 2H), 1.47 – 1.41 (m, 3H), 1.39 – 1.30 (m, 2H), 1.28 – 1.26 (m, 1H); **^{13}C NMR** (151 MHz, $CDCl_3$)**: δ** 175.5, 139.1 (2C), 129.0, 128.8, 128.6 (2C), 128.4, 128.3 (2C), 128.1, 127.2, 126.9, 82.9, 58.4, 54.6 (2C), 38.5, 36.7, 36.0, 24.9, 22.5, 22.4, **HRMS** (ESI$^+$): calculated for $C_{23}H_{28}O_2N$ [M+H]$^+$ 350.2120; found 350.2108, **IR** (neat, cm^{-1}): 3061, 3027, 2933, 2857, 2810, 1765, 1602, 1452, 1147, 738, 698.

3-Morpholino-1-oxaspiro[4.5]decan-2-one (5b)

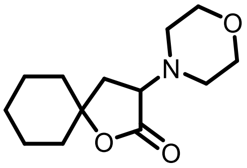

The title compound was prepared from morpholine using general procedure **C**. Purification by column chromatography on silica gel (20% DMA in DCM to 60% DMA) afforded compound **5b** in 68% yield (30.1 mg) as an off-white oil.

^{1}H NMR (600 MHz, $CDCl_3$)**: δ** 3.75 – 3.72 (m, *J* = 4.7 Hz, 4H), 3.66 – 3.61 (m, 1H), 2.88 – 2.82 (m, 2H), 2.55 – 2.50 (m, 2H), 2.22 (dd, *J* = 12.7, 9.0 Hz, 1H), 1.94 (dd, *J* = 12.7, 11.2 Hz, 1H), 1.81 – 1.67 (m, 4H), 1.65 – 1.60 (m, 1H), 1.58 – 1.47 (m, 4H), 1.42 – 1.34 (m, 1H); **^{13}C NMR** (151 MHz, $CDCl_3$)**: δ** 174.3, 83.1, 67.1, 63.5 (2C), 50.1 (2C), 38.5, 36.6, 34.8, 25.0, 22.7, 22.6; **HRMS** (ESI$^+$): calculated for $C_{13}H_{22}O_3N$ [M+H]$^+$ 240.1600; found 240.1599; **IR** (neat, cm^{-1}): 2932, 2855, 1760, 1449, 1146.

3-(Bis(2-hydroxyethyl)amino)-1-oxaspiro[4.5]decan-2-one (5c)

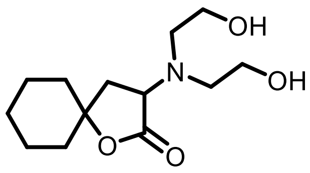

The title compound was prepared from diethanolamine using general procedure. Purification by column chromatography on silica gel (20% DMA in DCM to 100% DMA) afforded compound **5c** in 82% yield (41.6 mg) in as an off-white oil.

^{1}H NMR (600 MHz, $CDCl_3$): **δ** 4.06 (dd, *J* = 11.5, 9.2 Hz, 1H), 3.66 - 3.57 (m, 4H), 3.52 – 3.29 (m, 2H), 2.92 - 2.84 (m, 2H), 2.79 - 2.68 (m, 2H), 2.34 (dd, *J* = 12.8, 9.2 Hz, 1H), 1.89 (t, *J* = 12.2 Hz, 1H), 1.82 - 1.60 (m, 4H), 1.58 - 1.44 (m, 4H), 1.43 - 1.35 (m, 1H), 1.33 - 1.19 (m, 1H); **^{13}C NMR** (151 MHz, $CDCl_3$)**: δ** 177.0, 83.9, 61.7, 59.8 (2C), 53.7 (2C), 38.6, 36.5, 35.8, 25.0, 22.7, 22.5; **HRMS** (ESI$^+$): calculated for $C_{13}H_{24}O_4N$ $[M+H]^+$ *m/z*: 258.1705, found: 258.1701; **IR** (neat, cm^{-1})**:** 3403 (Bs), 2933, 2859, 1750, 1206, 1044.

3-(Diallylamino)-1-oxaspiro[4.5]decan-2-one (5d)

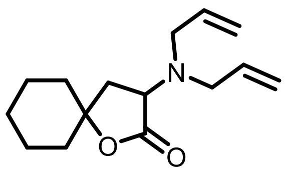

The title compound was prepared from diallylamine using general procedure **C**. Purification by column chromatography on silica gel (20% DMA in DCM to 60% DMA) afforded compound **5d** in 43% yield (20.2 mg) as an off-white oil.

^{1}H NMR (600 MHz, $CDCl_3$)**: δ** 5.88 – 5.78 (m, 2H), 5.24 (dd, *J* = 17.1, 1.6 Hz, 2H), 5.15 (dd, *J* = 10.2, 1.4 Hz, 2H), 4.04 – 3.96 (m, 1H), 3.34 (dd, *J* = 14.1, 6.5 Hz, 2H), 3.16 (dd, *J* = 14.1, 6.1 Hz, 2H), 2.19 (dd, *J* = 12.8, 9.3 Hz, 1H), 1.91 – 1.82 (m, 1H), 1.79 – 1.64 (m, 4H), 1.64 – 1.53 (m, 2H), 1.53 – 1.43 (m, *J* = 17.1, 7.9 Hz, 3H), 1.42 – 1.33 (m, 1H); **^{13}C NMR** (151 MHz, $CDCl_3$)**: δ** 175.6, 135.9 (C2), 117.8 (C2), 82.8, 59.2, 54.1 (C2), 38.5, 36.5, 35.5, 24.9, 22.6, 22.4; **HRMS** (ESI$^+$): calculated for $C_{15}H_{24}O_4N$ $[M+H]^+$ *m/z*: 250.1807, found: 250.1804; **IR** (neat, cm^{-1})**:** 2977, 3932, 2858, 1761, 1447, 1254, 1201, 881.

5. Procedures and characterization: Derivatization of the spiroaminolactones

(5*s,8*s**)-8-Phenyl-1-oxaspiro[4.5]dec-3-en-2-one (6)**

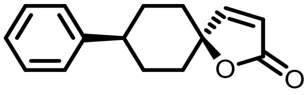

To a stirred solution of spirolactone **2j** (single diastereoisomer, 14 mg, 0.05 mmol, 1 eq.) in DCM (1 mL) was added *m*CPBA (17 mg, 0.075 mmol, 1.50 eq.) in one portion at 0 °C and the reaction mixture was stirred for 4 h at the same temperature. The reaction mixture was then treated with sat. aq. Na_2CO_3 solution (1 mL) and the mixture was stirred for 15 min. The phases were separated and the organic phase was dried over Na_2SO_4, concentrated, and purified by column chromatography on silica gel (heptane/EtOAc neat to 5:1) to afford the product **6** in 65% yield (7.4 mg) as a colorless oil.

^{1}H NMR (400 MHz, $CDCl_3$): **δ** 7.33 (d, *J* = 5.6 Hz, 1H), 7.30 – 7.22 (m, 2H), 7.19 – 7.11 (m, 3H), 5.97 (d, *J* = 5.6 Hz, 1H), 2.55 – 2.46 (m, 1H), 1.98 (dd, *J* = 13.3, 3.3 Hz, 1H), 1.92 (dd, *J* = 13.0, 3.7 Hz, 1H), 1.87 – 1.76 (m, 4H), 1.73 – 1.65 (m, 2H); **^{13}C NMR** (101 MHz, $CDCl_3$)**:** **δ** 172.6, 161.1, 146.2, 128.5, 126.9, 126.4, 120.4, 82.6, 42.9 (2C), 34.7, 29.6; **HRMS** (ESI$^+$): calculated for $C_{15}H_{16}O_2NNa$ [M+Na]$^+$ 251.1048; found 251.1038; **IR** (neat, cm^{-1}): 3081, 3063, 3028, 2929, 2861, 1757, 1493, 1444, 1275, 1214, 1189, 1123, 1108, 974, 921, 819, 701.

(5*s,8*s**)-3-(Dimethylamino)-8-phenyl-1-oxaspiro[4.5]dec-3-en-2-one (7)**

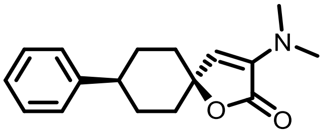

To a stirred solution of spirolactone **2j** (single diastereosiomer,14 mg, 0.05 mmol, 1 eq.) in DCM (1 mL) was added *m*CPBA (17 mg, 0.075 mmol, 1.50 eq.) in one portion and the reaction mixture was heated at 60 °C for 12 h. The reaction mixture was then treated with sat. aq. Na_2CO_3 solution (1 mL) and the mixture was stirred for 15 min. The phases were separated and the organic phase was dried over Na_2SO_4, concentrated, and purified by column chromatography on silica gel (heptane/EtOAc neat to 5:1) to afford the product **7** in 47% yield (6.4 mg) as a colorless oil.

^{1}H NMR (400 MHz, $CDCl_3$)**:** **δ** 7.34 – 7.27 (m, 3H), 7.25 – 7.17 (m, 2H), 5.74 (s, 1H), 2.86 (s, 6H), 2.53 (tt, *J* = 12.3, 3.4 Hz, 1H), 2.04 – 1.94 (m, 2H), 1.86 – 1.80 (m, 4H), 1.78 – 1.68 (m,

2H); **^{13}C NMR** (151 MHz, $CDCl_3$)**: δ** 169.2, 146.7, 138.9, 128.6, 127.0, 126.3, 124.1, 82.9, 43.1 (2C), 40.4, 36.9, 30.2; **HRMS** (ESI$^+$): calculated for $C_{17}H_{21}O_2NNa$ [M+Na]$^+$ 294.1470; found 294.1461; **IR** (neat, cm^{-1}): 3030, 2996, 2933, 2872, 2856, 2798, 1763, 1455, 1440, 1276, 1222, 1147, 1103, 1083, 977, 763, 702.

Proposed mechanism for formation of product 7

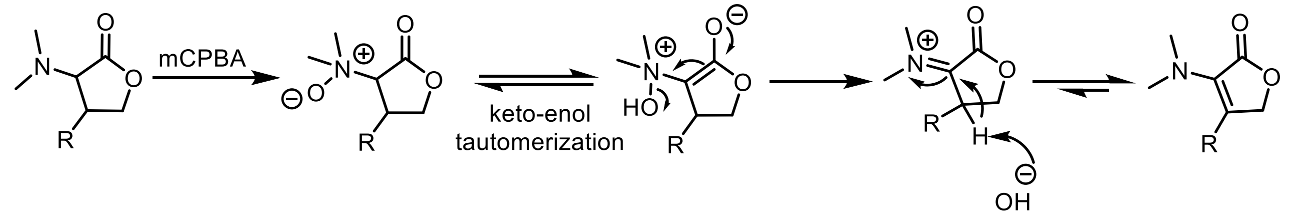

(3*S,5s*,8*R**)-3-Allyl-3-(dimethylamino)-8-phenyl-1-oxaspiro[4.5]decan-2-one (8)**

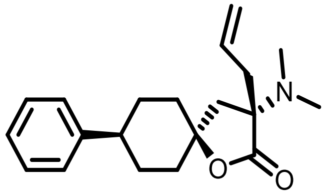

To a stirred solution of spirolactone **2j** (single diastereoisomer, 14 mg, 0.05 mmol, 1 eq.) in MeOH (0.1 mL) was added allyl bromide (4.5 µL, 0.053 mmol, 1.05 eq.) in one portion and the reaction mixture was stirred at 23 °C for 15 h. The reaction mixture was subsequently concentrated under reduced pressure, providing the quaternary amine salt as an off-white solid. The solid was redissolved in anhydrous THF (0.5 mL) under an argon atmosphere, cooled to –78 °C, and *t*BuOK (8.7 mg, 0.075 mmol, 1.50 eq.) was added. The reaction mixture was stirred for 4 h at the same temperature, before being treated with a sat. aq. NH_4Cl solution (1 mL), and extracted by Et_2O (3 × 10 mL). The combined organic layers were dried over Na_2SO_4, filtered, and evaporated under reduced pressure. The crude product was purified by column chromatography on silica gel (heptane/EtOAc, neat to 5:1) to yield spirolactone **8** quantitatively (15.3 mg) as an off-white solid.

^{1}H NMR (400 MHz, $CDCl_3$)**: δ** 7.33 – 7.26 (m, 2H), 7.25 – 7.17 (m, 3H), 5.89 (ddt, *J* = 17.2, 10.3, 7.1 Hz, 1H), 5.24 – 5.15 (m, 2H), 2.61 – 2.49 (m, 3H), 2.34 (s, 6H), 2.22 (d, *J* = 14.1 Hz, 1H), 2.21 – 2.14 (m, 1H), 2.06 – 1.88 (m, 3H), 1.94 (d, *J* = 14.1 Hz, 1H), 1.82 – 1.74 (m, 2H), 1.70 – 1.59 (m, 2H); **^{13}C NMR** (101 MHz, $CDCl_3$)**: δ** 176.7, 146.5, 133.0, 128.5, 127.0, 126.3, 119.6, 81.3, 68.7, 43.3, 40.2, 39.6, 39.3, 38.6, 29.9 (2C); **HRMS** (ESI$^+$): calculated for $C_{20}H_{28}O_2N$ [M+Na]$^+$ 314.2120; found 314.2115; **IR** (neat, cm^{-1}): 3077, 3061, 3026, 2979, 2930, 2863, 2833, 2788, 1759, 1493, 1458, 1448, 1273, 1214, 1192, 1158, 1130, 1093, 1044, 968, 950, 758, 700.

(3*R,5*s**,8*S**)-3-(Dimethylamino)-3-(2-methylbut-3-en-2-yl)-8-phenyl-1-oxaspiro[4.5]decan-2-one (9)**

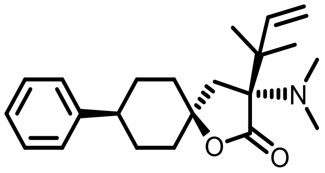

To a stirred solution of spirolactone **2j** (single diastereoisomer, 14 mg, 0.05 mmol, 1 eq.) in MeOH (0.1 mL) was added prenyl bromide (6.7 µL, 0.053 mmol, 1.05 eq.) in one portion and the reaction mixture was stirred at 23 °C for 15 h. The reaction mixture was concentrated under reduced pressure, providing the quaternary amine salt as an off-white solid. The solid was redissolved in anhydrous THF (0.5 mL) under an argon atmosphere, cooled to –78 °C, and *t*BuOK (8.7 mg, 0.075 mmol, 1.5 eq.) was added. The reaction mixture was stirred for 1 h at the same temperature, before being treated with a sat. aq. NH_4Cl solution (1 mL), and extracted with Et_2O (3 × 10 mL). The combined organic layers were dried over Na_2SO_4, filtered, and evaporated under reduced pressure. The crude product was purified by column chromatography on silica gel (heptane/EtOAc, neat to 5:1) to yield spirolactone **9** in 72% (12.3 mg) as an off-white solid.

^{1}H NMR (400 MHz, $CDCl_3$)**: δ** 7.32 – 7.27 (m, 2H), 7.25 – 7.16 (m, 3H), 6.15 (dd, *J* = 17.4, 10.9 Hz, 1H), 5.07 (dd, *J* = 17.4, 1.1 Hz, 1H), 5.05 (dd, *J* = 10.9, 1.2 Hz, 1H), 2.58 – 2.48 (m, 1H), 2.47 (s, 6H), 2.28 – 2.23 (m, 1H), 2.20 (d, *J* = 14.3 Hz, 1H), 2.05 (d, *J* = 14.3 Hz, 1H), 2.01 – 1.85 (m, 3H), 1.84 – 1.72 (m, 2H), 1.69 – 1.60 (m, 1H), 1.53 (td, *J* = 13.7, 4.1 Hz, 1H), 1.30 (s, 3H), 1.19 (s, 3H); **^{13}C NMR** (101 MHz, $CDCl_3$)**: δ** 175.8, 146.7, 146.0, 128.5, 127.0, 126.3, 113.1, 80.5, 73.8, 45.4, 43.5, 41.9 (2C), 38.8, 38.7, 37.9, 30.4, 29.8, 25.4, 23.2; **HRMS** (ESI$^+$): calculated for $C_{22}H_{32}O_2N$ [M+H]$^+$ 342.2433; found 342.2419; **IR** (neat, cm^{-1}): 3082, 3060, 3027, 2980, 2862, 2784, 1755, 1453, 1276, 1213, 1190, 1131, 1030, 967, 924, 757, 700.

(3*R,5*s**,8*S**)-3-(Dimethylamino)-8-phenyl-3-(propa-1,2-dien-1-yl)-1-oxaspiro[4.5]decan-2-one (10)**

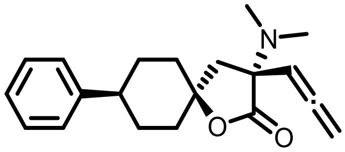

To a stirred solution of spirolactone **2j** (single diastereoisomer, 14 mg, 0.05 mmol, 1 eq.) in MeOH (0.1 mL) was added propargyl bromide (4 µL, 0.053 mmol, 1.05 eq.) in one portion and the reaction mixture was stirred at 23 °C for 15 h. The reaction mixture was concentrated under reduced pressure, providing the quaternary amine salt as an off-white solid. The solid was redissolved in anhydrous THF (0.5 mL) under an argon atmosphere and *t*BuOK (8.7 mg, 0.075 mmol, 1.5 eq.) was added rapidly at 23 °C. The reaction mixture was stirred for 15 h at the same temperature, before being treated with a sat. aq. NH_4Cl solution (1 mL), and extracted with Et_2O (3 × 10 mL). The combined organic layers were dried over Na_2SO_4, filtered, and evaporated under reduced pressure. The crude product was purified by column chromatography on silica gel (heptane/EtOAc, neat to 5:1) to yield spirolactone **10** in 66% (10.3 mg) as an off-white solid.

^{1}H NMR (400 MHz, $CDCl_3$)**: δ** 7.33 – 7.27 (m, 2H), 7.24 – 7.17 (m, 3H), 5.44 (t, *J* = 6.6 Hz, 1H), 5.00 (d, *J* = 6.6 Hz, 2H), 2.54 (tt, *J* = 12.2, 3.5 Hz, 1H), 2.29 (s, 6H), 2.20 (d, *J* = 13.7 Hz, 1H), 2.15 (d, *J* = 13.8 Hz, 1H), 2.09 – 2.02 (m, 2H), 1.99 – 1.84 (m, 2H), 1.83 – 1.65 (m, 3H), 1.63 – 1.54 (m, 1H); **^{13}C NMR** (101 MHz, $CDCl_3$)**: δ** 208.6, 175.7, 146.5, 128.6, 127.0, 126.3, 93.5, 81.7, 79.7, 68.9, 43.4 (2C), 39.6, 38.8, 38.3, 36.3, 30.1, 29.7; **HRMS** (ESI$^+$): calculated for $C_{20}H_{25}O_2N$ $[M+H]^+$ 312.1964; found 312.1957; **IR** (neat, cm^{-1}): 3026, 2986, 2945, 2927, 2862, 2826, 2784, 1954, 1763, 1494, 1458, 1275, 1218, 1192, 1129, 1045, 969, 949, 759, 701.

(3*S,5*s**,8*R**)-3-Benzyl-3-(dimethylamino)-8-phenyl-1-oxaspiro[4.5]decan-2-one (11)**

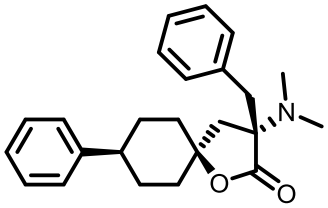

To a stirred solution of spirolactone **2j** (single diastereoisomer, 14 mg, 0.05 mmol, 1.0 eq.) in MeOH (0.1 mL) was added benzyl bromide (6.3 µL, 0.053 mmol, 1.05 eq.) in one portion and the reaction mixture was stirred at 23 °C for 15 h. The reaction mixture was concentrated under reduced pressure, providing the quaternary amine salt as an off-white solid. The solid was redissolved in anhydrous THF (0.5 mL) under an argon atmosphere and *t*BuOK (8.7 mg, 0.075 mmol, 1.5 eq.) was added at 23 °C. The reaction mixture was stirred for 15 h at the same temperature, before being treated with a sat. aq. NH_4Cl solution (1 mL), and extracted by Et_2O (3 × 10 mL). The combined organic layers were dried over Na_2SO_4, filtered, and evaporated under reduced pressure. The crude product was purified by column chromatography on silica gel (heptane/EtOAc, neat to 5:1) to yield spirolactone **11** in 76% (13.8 mg) as an off-white solid.

^{1}H NMR (400 MHz, $CDCl_3$)**: δ** 7.31 – 7.19 (m, 7H), 7.17– 7.10 (m, 3H), 3.33 (d, *J* = 13.0 Hz, 1H), 2.90 (d, *J* = 13.1 Hz, 1H), 2.43 (s, 6H), 2.41 – 2.32 (m, 1H), 2.26 – 2.12 (m, 1H), 2.12 (d, *J* = 14.1 Hz, 1H), 1.97 (d, *J* = 14.2 Hz, 1H), 1.89 – 1.75 (m, 1H), 1.75 – 1.65 (m, 1H), 1.65 – 1.39 (m, 3H), 1.07 (td, *J* = 13.7, 4.2 Hz, 1H), 0.34 (ddd, *J* = 14.0, 5.9, 2.9 Hz, 1H); **^{13}C NMR** (101 MHz, $CDCl_3$)**: δ** 177.1, 146.6, 136.3, 131.1, 128.7, 128.4, 127.3, 126.9, 126.2, 81.5, 70.8, 43.2, 40.0, 39.9, 38.3, 37.7, 37.6, 29.8, 29.7; **HRMS** (ESI$^+$): calculated for $C_{24}H_{30}O_2N$ $[M+H]^+$ 364.2277; found 364.2270; **IR** (neat, cm^{-1}): 3083, 3061, 3027, 3000, 2930, 2863, 2833, 2789, 1759, 1494, 1455, 1274, 1236, 1216, 1188, 1156, 1128, 1097, 1040, 968, 758, 735, 701.

6. Assessment of the reactivity of different classes of alkenes

As can be inferred from the scope presented within the manuscript, during the course of our investigations, we found 1,1-disubstituted alkenes to be privileged motifs for this aminolactonization reaction.

Below, we aim to showcase the less selective reactivity of other classes of alkenes and comment on the regioselectivity that was observed for substrates presented in the reaction scope (Scheme 1 of the manuscript).

As previously reported, monosubstituted terminal alkenes tend to favor formation of products derived from hydroaminoalkylation.[1] This preference, however, does not always preclude the formation of aminolactones, as 4-phenyl-1-butene was found to provide **S1** in 23% NMR yield. A similarly non-selective reaction was observed for olefins embedded within a carbocycle, such as cycloheptene (giving product **S2** in 42% yield alongside varying amounts of hydroaminoalkylation products) and 1-methylcyclohexene (**S3** formed in 20% NMR yield).

Alkenes bearing electron-withdrawing substituents—such as methyl acrylate—were not found to undergo any sort of productive reaction under the reported conditions, while dienes—such as *trans*-1-phenyl-1,3-butadiene—underwent non-specific decomposition with no detectable product formation.

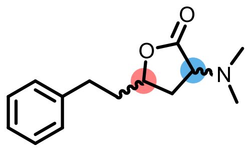

S1
23% NMR yield

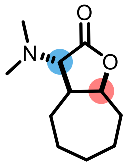

S2
42% isolated yield

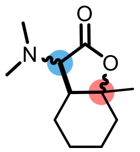

S3
20% NMR yield

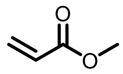

methyl acrylate
No product formed

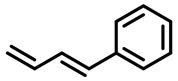

***trans*-1-phenyl-1,3-butadiene**
Decomposition

With regard to the chemoselectivity observed for products bearing multiple olefins, several factors might be at play.

2x: Throughout our investigations of the reactions of iminium ions with electron-rich double bonds, we have observed significant reductions of reactivity for species bearing protonated heteroatoms. Product **2x**, which exists in a protonated state under the reaction conditions,

thus also avoids overreaction at the terminal monosubstituted olefin (and other olefins present in other substrates or products, such as **2y** and **5d**). Despite having no evidence of this effect, we are led to believe that this must be the result of inductive effects.

2y: The preference for reaction at the exocyclic methylene (terminal, 1,1-disubstituted) can be related to the result observed for 1-methylcyclohexene shown above, where the 1,1,2-trisubstituted double bond was found to react sluggishly. Moreover, it can be assumed that the 1,1-disubstituted double bond of **1y** is more sterically accessible than the endocyclic trisubstituted double bond.

The result obtained for **2y** can also be contrasted with **2w**, which does result from the reaction of a trisubstituted double bond. However, in comparison with **1y**, the olefin found in **1w** is i) likely more sterically accessible and ii) its reactivity is not attenuated by the inductive effects invoked above.

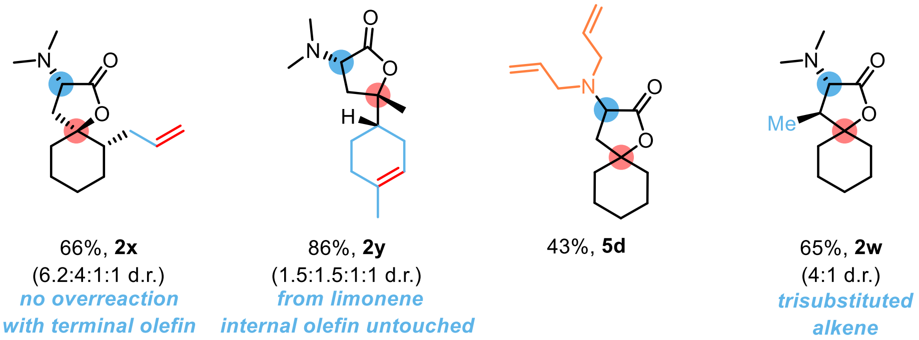

7. Comparison with the Huang & Li procedure[14]

In order to establish reproducibility of the previously reported transformation, a test reaction was performed, aiming to obtain the product of Run 6 (Table 2). As shown below, the reaction was found to be reproducible in our hands.

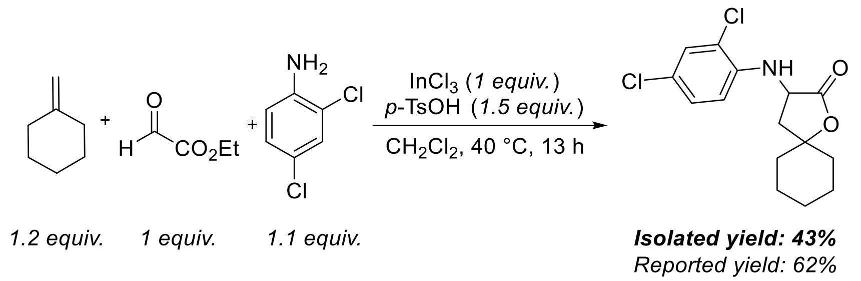

For this reason, we set out to survey amines not bearing electron-depleting substituents. As can be seen below, the reaction of methylenecyclohexane, ethyl glyoxylate and dibenzylamine did not lead to the formation of quantifiable amounts of product.

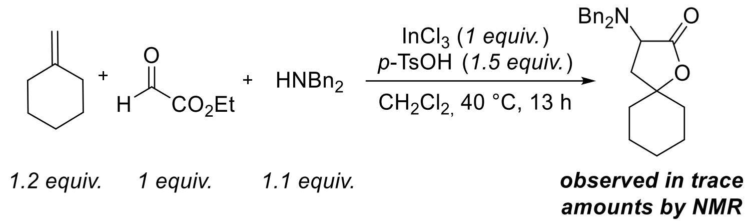

We further wanted to probe the ability of the previously reported conditions to tolerate potentially sensitive functional groups. For this reason, 8-methylene-1,4-dioxaspiro[4.5]decane (**1n**) was subjected to the reaction conditions using a suitable, electron-deficient amine.

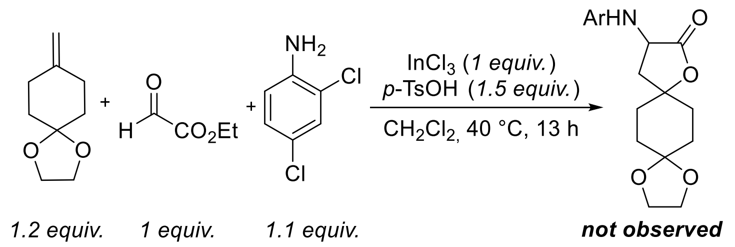

Notably, this transformation also did not yield any of the anticipated product, leading only to non-specific decomposition on the acetal-containing starting material, which could not be recovered. This result further highlights the contrasts between the related previously reported procedure and the transformation shown herein.

8. Stereochemical assignments

An explanation of pseudosymmetry on lactone **2j** is depicted in the following scheme.

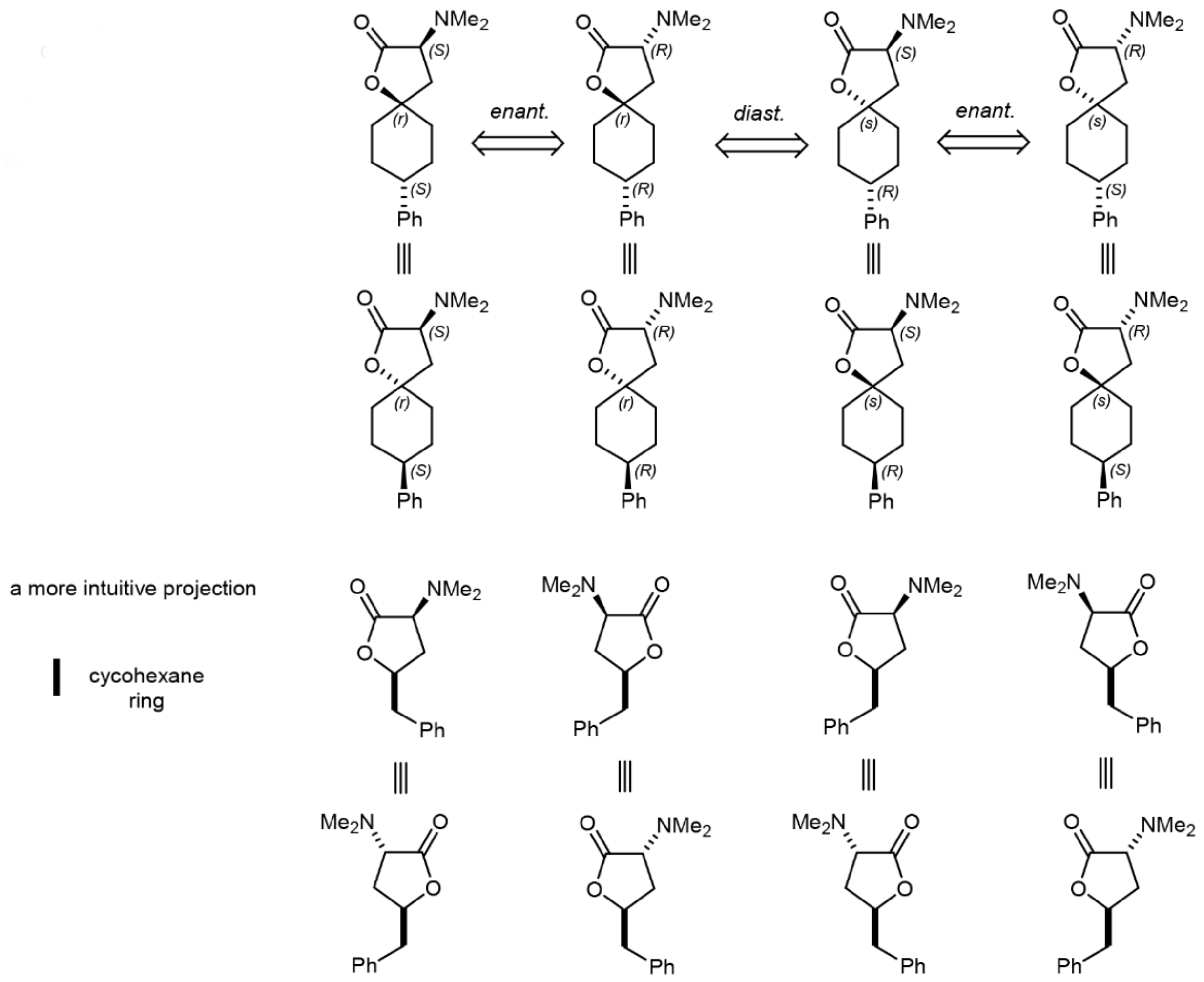

The major diastereoisomer of **2j** was crystallized from heptane/EtOAc and analyzed by X-ray crystallography showing a *syn* relationship between Cx and Ox.

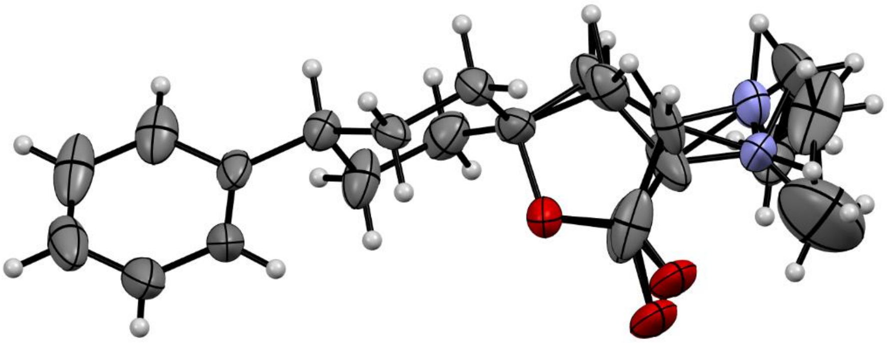

The five-membered lactone ring is disordered, likely due to pseudosymmetry on the spiro-quaternary carbon center.

The ^{1}H NMR assignment of protons on the CH_2 atom at the five-membered lactone ring showed similar behavior common for all compounds **2h**, **2i**, **2k**, **2o**, **2q**, and **2r** having the same 1,4-substitution pattern on the cyclohexane ring. While protons of the major diastereomer connected to the carbon atom in the equatorial position exhibit 2.03 – 2.15 ppm values, one of the protons of the minor diastereomer connected to the carbon atom in the axial position was shifted down-field, up to 2.46 ppm. This phenomenon is attributed to the clash of the proton with axial protons on the six-membered ring.

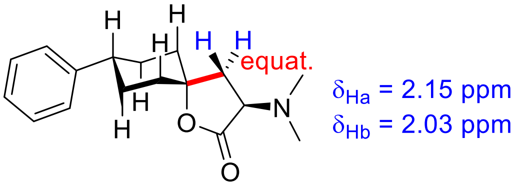

major diastereomer **2j**

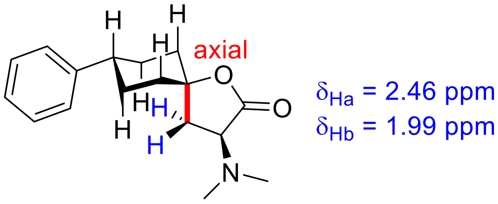

minor diastereomer **2j**

Therefore, the relative configurations of compounds **2h**, **2i**, **2k**, **2o**, **2q**, and **2r** were assigned in analogy to have *syn* configuration at Cx-Ox of the major diastereoisomer.

The configuration of compounds **2l**, **2m**, **2p**, **2s**, **2v**, and **2x** having 1,2 substitution patterns on the cyclohexane ring and exhibiting no pseudochirality could not be analyzed by X-Ray crystallography. The NOESY experiment of the major diastereomer of **2p** showed strong NOE cross-peaks between protons of the NMe_2 group with the CH_2 group neighboring the quaternary spiro center. Moreover, the proton adjacent to the NMe_2 group showed cross-

peaks of medium to weak intensity with all of the protons of the EtO group, suggesting that the NMe_2 group has a syn relationship with the spirocyclic oxygen atom. On the other hand, the chemical shifts of CH_2 protons on the lactone ring are shifted up to 2.49 ppm, resulting from this proton's axial location. Additionally, the acidic proton adjacent to the NMe_2 group is shifted significantly downfield, up to 3.95 ppm, which is likely a deshielding result of the quasi-hydrogen bond with the EtO group located in an equatorial position.

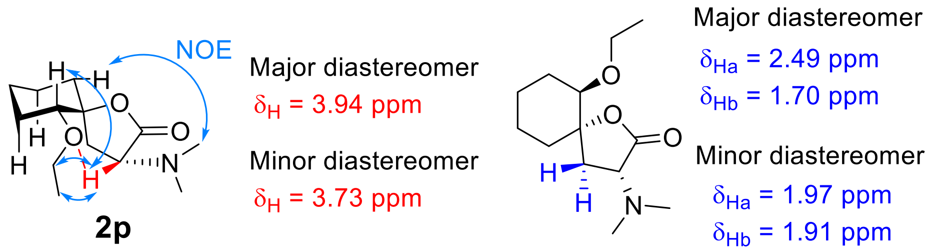

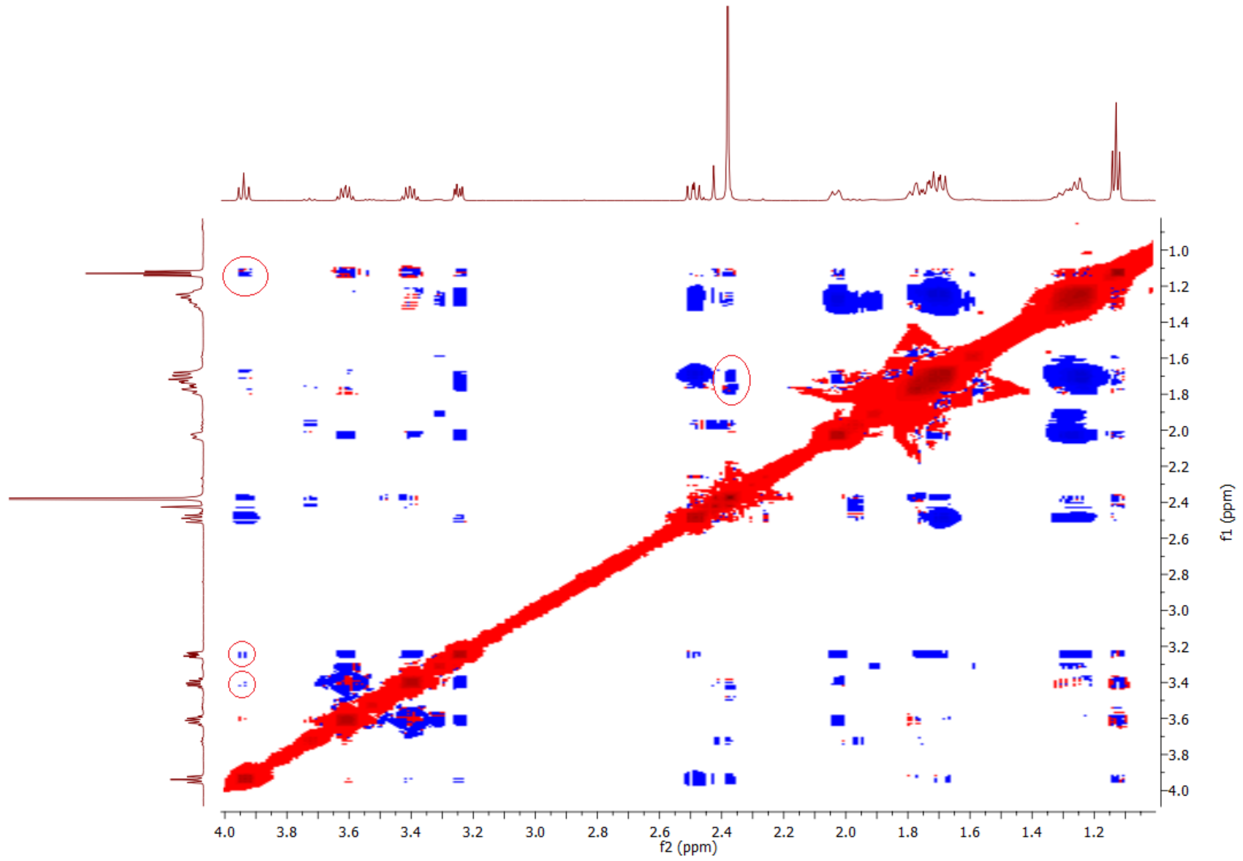

Therefore, the relative configuration of compounds **2l**, **2m**, **2s**, **2v**, and **2x** were assigned in analogy.

9. X-Ray crystallographic data of 2j

checkCIF/PLATON report

Structure factors have been supplied for datablock(s) daju196maj

THIS REPORT IS FOR GUIDANCE ONLY. IF USED AS PART OF A REVIEW PROCEDURE FOR PUBLICATION, IT SHOULD NOT REPLACE THE EXPERTISE OF AN EXPERIENCED CRYSTALLOGRAPHIC REFEREE.

No syntax errors found. CIF dictionary Interpreting this report

Datablock: daju196maj

Bond precision:	C-C = 0.0066 A		Wavelength=0.71073
Cell:	a=14.635(2)	b=10.097(2)	c=20.471(3)
	alpha=90	beta=103.824(11)	gamma=90
Temperature:	100 K		

	Calculated	Reported
Volume Space	2937.4(8)	2937.6(8)
groupHall	I 2/a	I 1 2/a 1
group	-I 2ya C17	-I 2ya C17
Moiety formula	H23 N O2	H23 N O2
Sum formula	C17 H23 N O2	C17 H23 N O2
Mr	273.36	273.36
Dx,g cm-3	1.236	1.236
Z	8	8
Mu (mm-1)	0.080	0.080

F000	1184.0	1184.0
F000'	1184.50	
h,k,lmax	17,12,24	17,12,24
Nref	2779	2744
Tmin,Tmax	0.955,0.972	0.693,0.993
Tmin'	0.953	

Correction method= # Reported T Limits: Tmin=0.693 Tmax=0.993AbsCorr = MULTI-SCAN

Data completeness= 0.987 Theta(max)= 25.680

R(reflections)= 0.0991(1290)

wR2(reflections)= 0.3023(2744)

S = 1.043 Npar= 232

The following ALERTS were generated. Each ALERT has the format

test-name_ALERT_alert-type_alert-level.

Click on the hyperlinks for more details of the test.

Alert level C

PLAT026_ALERT_3_C Ratio Observed / Unique Reflections (too) Low .. 47% Check

PLAT031_ALERT_4_C Refined Extinction Parameter Within Range of ... 2.737 Sigma

PLAT084_ALERT_3_C High wR2 Value (i.e. > 0.25) .... 0.30 Report

PLAT213_ALERT_2_C Atom C13A has ADP max/min Ratio ..... 3.6 prolat

PLAT213_ALERT_2_C Atom C17A has ADP max/min Ratio ..... 4.0 prolat

PLAT213_ALERT_2_C Atom C13B has ADP max/min Ratio ..... 3.6 prolat

PLAT220_ALERT_2_C NonSolvent Resd 1 C Ueq(max)/Ueq(min) Range 5.1 Ratio

PLAT222_ALERT_3_C NonSolvent Resd 1 H Uiso(max)/Uiso(min) Range 6.3 Ratio

PLAT230_ALERT_2_C Hirshfeld Test Diff for C4 --C5 . 6.0 s.u.

PLAT230_ALERT_2_C Hirshfeld Test Diff for C10 --C11 . 5.3 s.u.

PLAT234_ALERT_4_C Large Hirshfeld Difference O1A --C13A . 0.21 Ang.

PLAT234_ALERT_4_C Large Hirshfeld Difference N1A --C16A . 0.20 Ang.

PLAT234_ALERT_4_C Large Hirshfeld Difference N1A --C17A . 0.18 Ang.

PLAT234_ALERT_4_C Large Hirshfeld Difference C13A --C14A . 0.23 Ang.

PLAT309_ALERT_2_C Single Bonded Oxygen (C-O > 1.3 Ang) .... O1B Check

PLAT340_ALERT_3_C Low Bond Precision on C-C Bonds .... 0.00662 Ang.

PLAT906_ALERT_3_C Large K Value in the Analysis of Variance ...... 21.312 Check

PLAT906_ALERT_3_C Large K Value in the Analysis of Variance ...... 3.599 Check

PLAT911_ALERT_3_C Missing FCF Refl Between Thmin & STh/L= 0.600 31 Report

---

Alert level G

PLAT002_ALERT_2_G Number of Distance or Angle Restraints on AtSite 14 Note
PLAT003_ALERT_2_G Number of Uiso or Uij Restrained non-H Atoms ... 4 Report
PLAT072_ALERT_2_G SHELXL First Parameter in WGHT Unusually Large 0.17 Report
PLAT171_ALERT_4_G The CIF-Embedded .res File Contains EADP Records 3 Report
PLAT176_ALERT_4_G The CIF-Embedded .res File Contains SADI Records 3 Report
PLAT186_ALERT_4_G The CIF-Embedded .res File Contains ISOR Records 2 Report
PLAT301_ALERT_3_G Main Residue Disorder ....(Resd 1) 35% Note
PLAT413_ALERT_2_G Short Inter XH3 .. XHn H4 ..H17D . 2.14 Ang.
-x,1/2+y,1/2-z = 4_455 Check
PLAT793_ALERT_4_G Model has Chirality at C14A (Centro SPGR) S Verify
PLAT793_ALERT_4_G Model has Chirality at C14B (Centro SPGR) R Verify
PLAT811_ALERT_5_G No ADDSYM Analysis: Too Many Excluded Atoms .... ! Info
PLAT860_ALERT_3_G Number of Least-Squares Restraints .... 46 Note
PLAT912_ALERT_4_G Missing # of FCF Reflections Above STh/L= 0.600 5 Note
PLAT933_ALERT_2_G Number of HKL-OMIT Records in Embedded .res File 1 Note
PLAT941_ALERT_3_G Average HKL Measurement Multiplicity .... 4.8 Low
PLAT978_ALERT_2_G Number C-C Bonds with Positive Residual Density. 0 Info

---

0 **ALERT level A** = Most likely a serious problem - resolve or explain
0 **ALERT level B** = A potentially serious problem, consider carefully
19 **ALERT level C** = Check. Ensure it is not caused by an omission or oversight
16 **ALERT level G** = General information/check it is not something unexpected

0 ALERT type 1 CIF construction/syntax error, inconsistent or missing data

13 ALERT type 2 Indicator that the structure model may be wrong or deficient

10 ALERT type 3 Indicator that the structure quality may be low

11 ALERT type 4 Improvement, methodology, query or suggestion

1 ALERT type 5 Informative message, check

It is advisable to attempt to resolve as many as possible of the alerts in all categories. Often the minor alerts point to easily fixed oversights, errors and omissions in your CIF or refinement strategy, so attention to these fine details can be worthwhile. In order to resolve some of the more serious problems it may be necessary to carry out additional measurements or structure refinements. However, the purpose of your study may justify the reported deviations and the more serious of these should normally be commented upon in the discussion or experimental section of a paper or in the "special_details" fields of the CIF. checkCIF was carefully designed to identify outliers and unusual parameters, but every test has its limitations and alerts that are not important in a particular case may appear. Conversely, the absence of alerts does not guarantee there are no aspects of the results needing attention. It is up to the individual to critically assess their own results and, if necessary, seek expert advice.

Publication of your CIF in IUCr journals

A basic structural check has been run on your CIF. These basic checks will be run on all CIFs submitted for publication in IUCr journals (*Acta Crystallographica*, *Journal of Applied Crystallography*, *Journal of Synchrotron Radiation*); however, if you intend to submit to *Acta Crystallographica Section C* or *E* or *IUCrData*, you should make sure that full publication checks are run on the final version of your CIF prior to submission.

Publication of your CIF in other journals

Please refer to the *Notes for Authors* of the relevant journal for any special instructions relating to CIF submission.

PLATON version of 18/11/2022; check.def file version of 18/11/2022

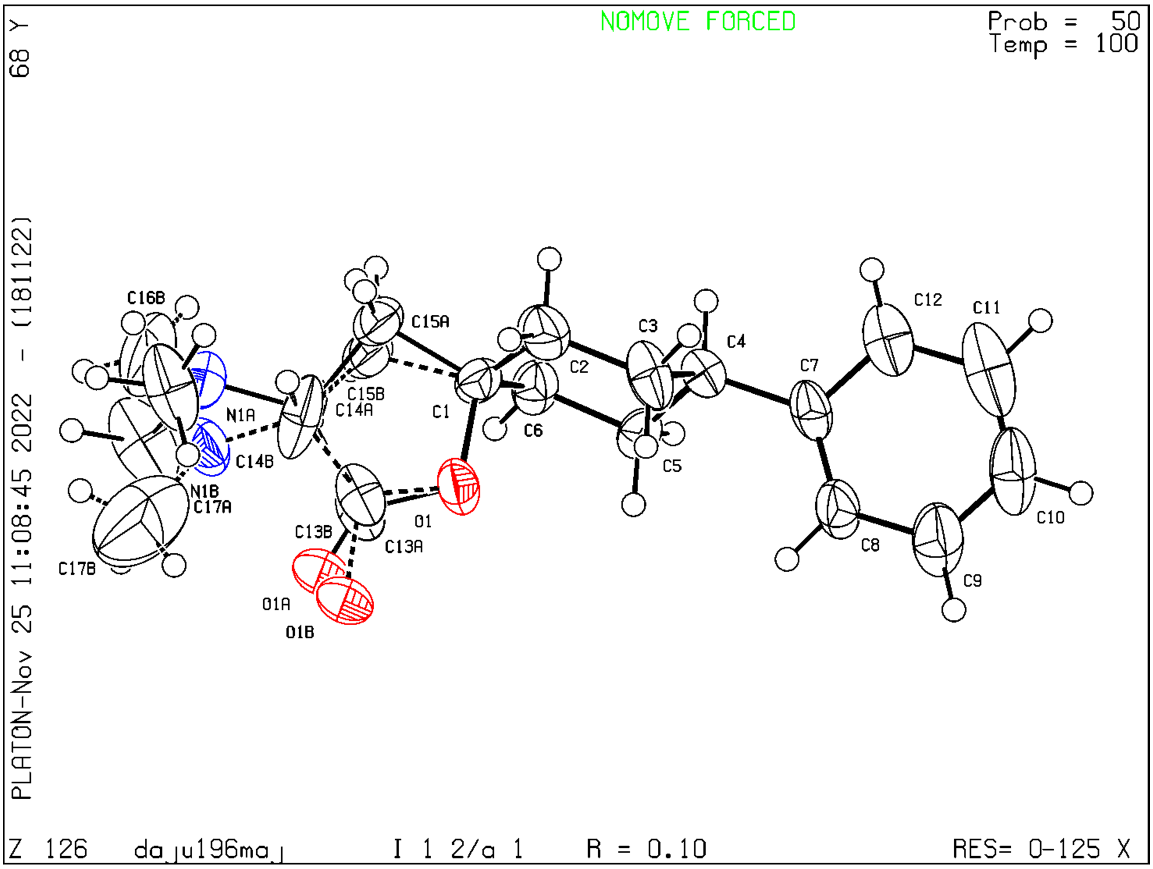

10. Spectral data

3-(Dimethylamino)-1-oxaspiro[4.5]decan-2-one (2a)

^{1}H NMR (600 MHz, $CDCl_3$)

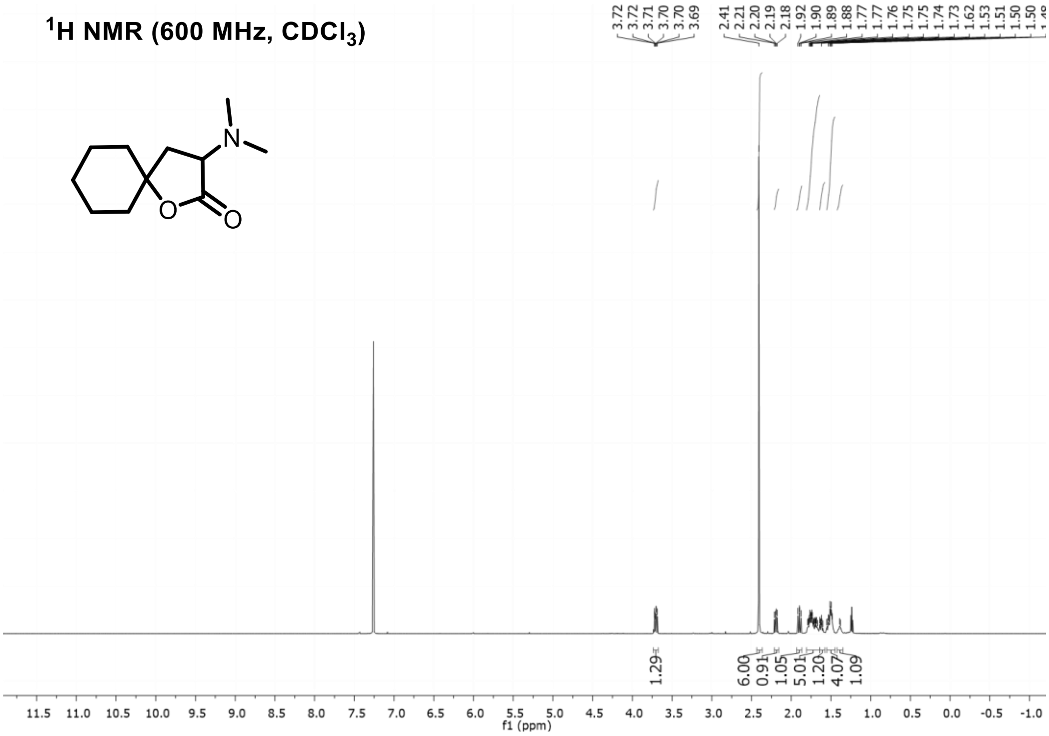

^{13}C NMR (151 MHz, $CDCl_3$)

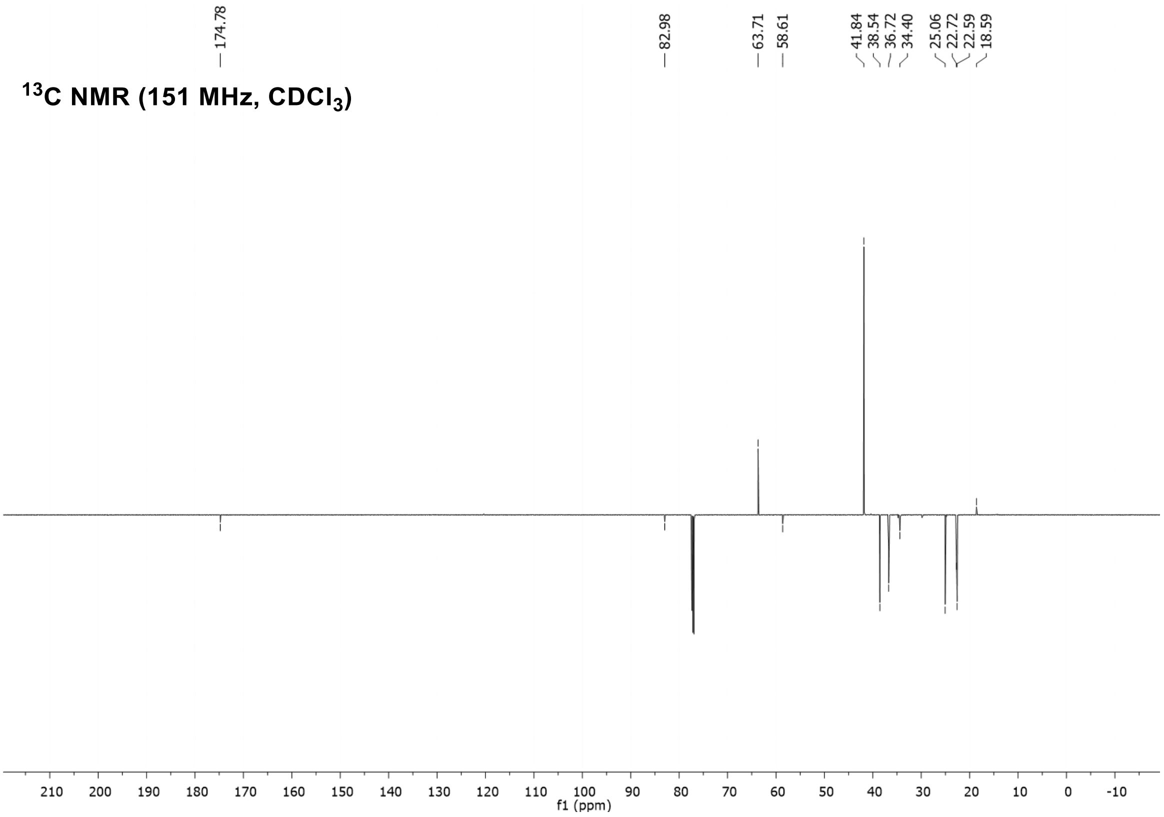

3-(Dimethylamino)-1-oxaspiro[4.6]undecan-2-one (2b)

^{1}H NMR (600 MHz, $CDCl_3$)

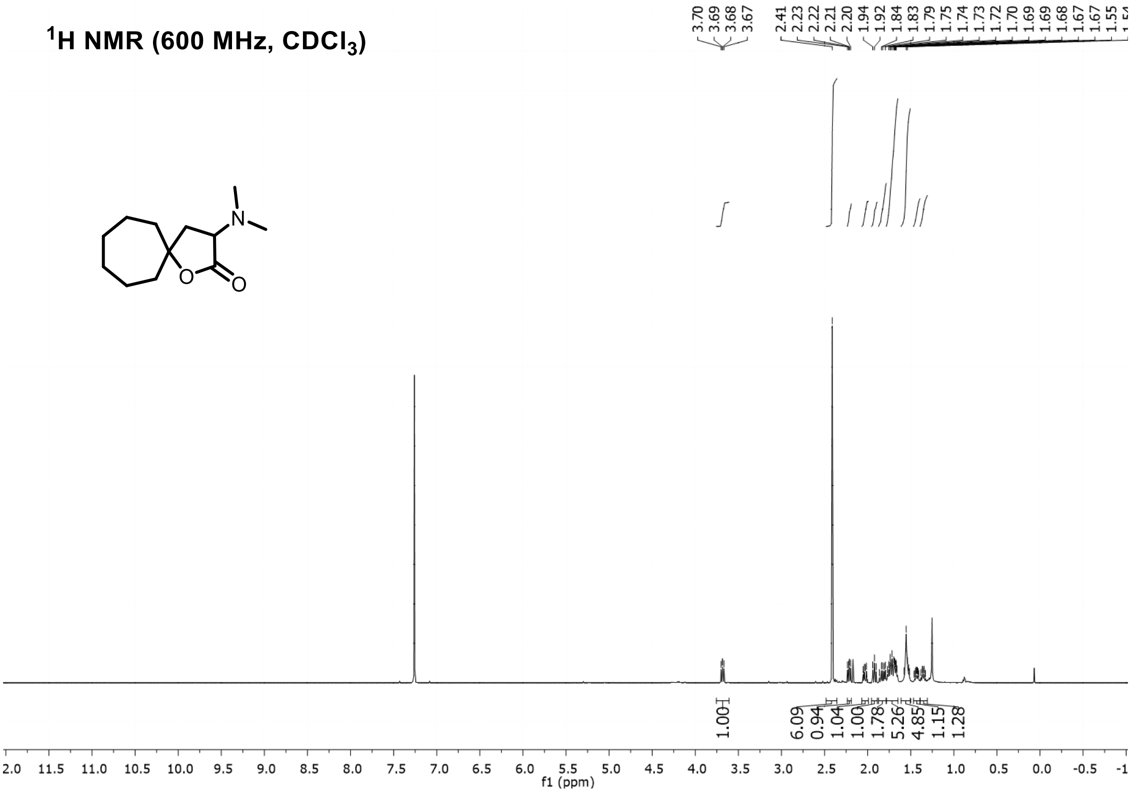

^{13}C NMR (151 MHz, $CDCl_3$)

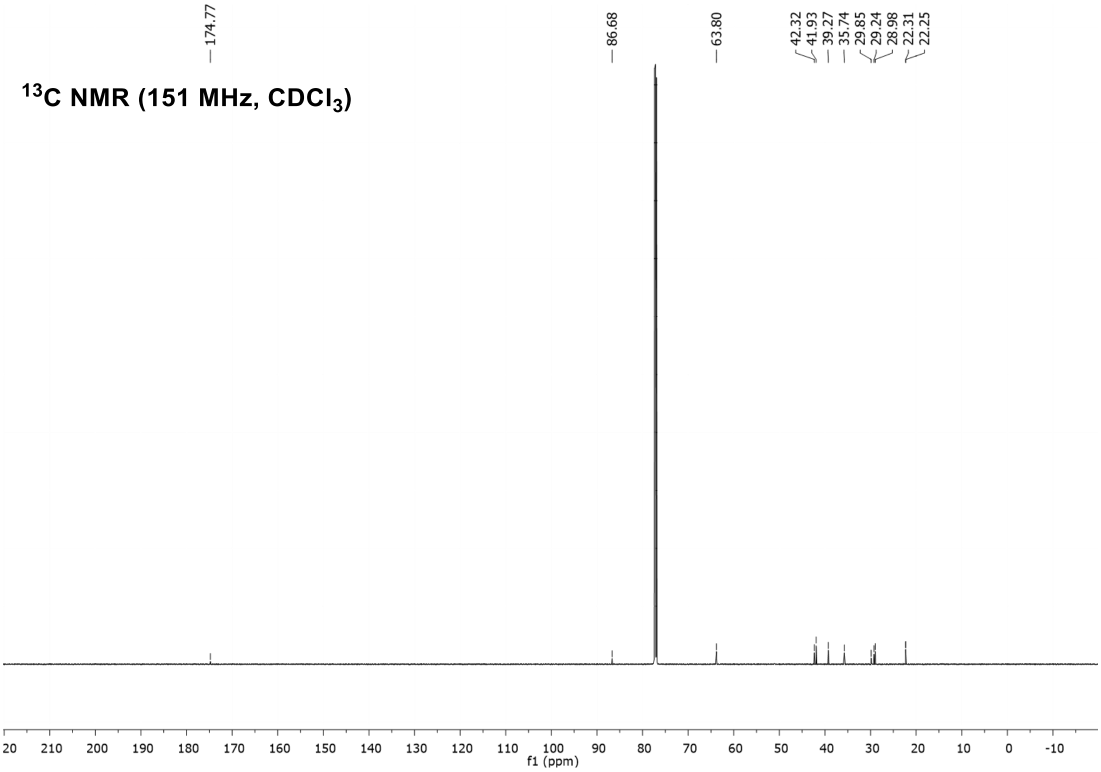

3-(Dimethylamino)-1-oxaspiro[4.4]nonan-2-one (2c)

^{1}H NMR (400 MHz, $CDCl_3$)

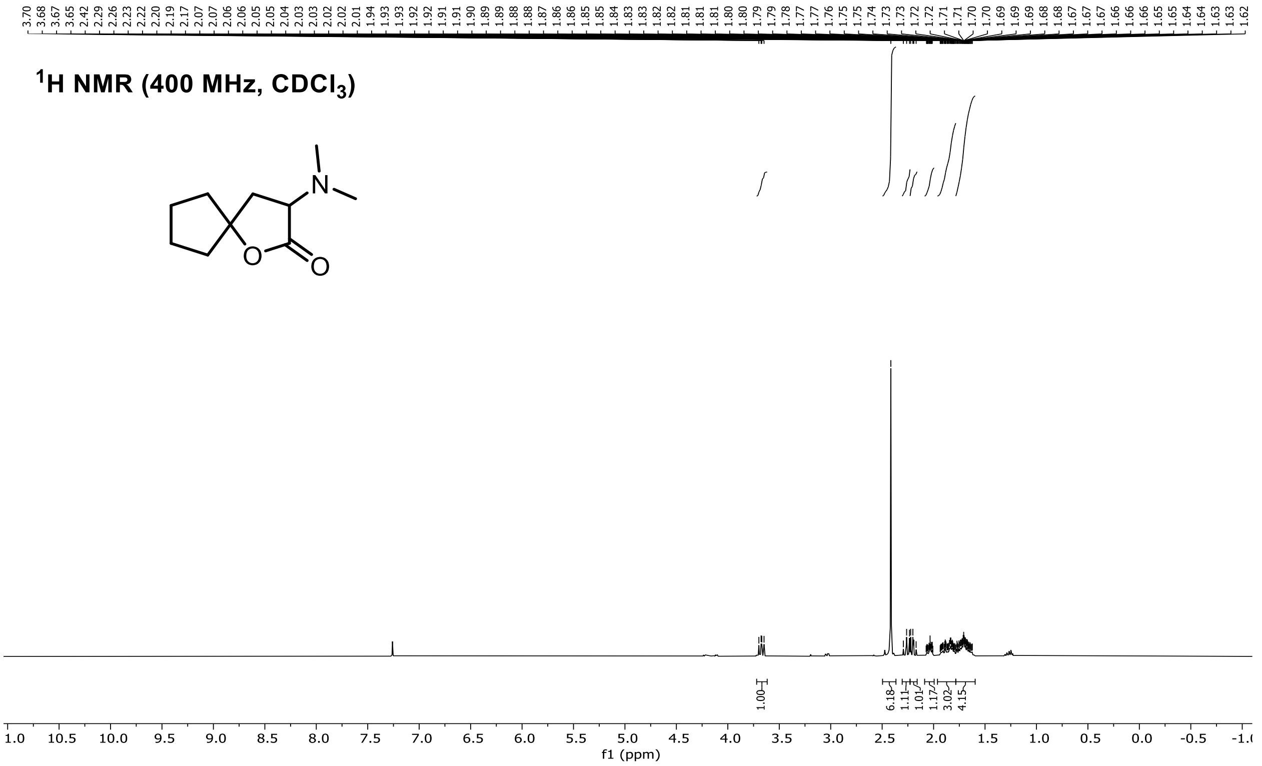

^{13}C NMR (101 MHz, $CDCl_3$)

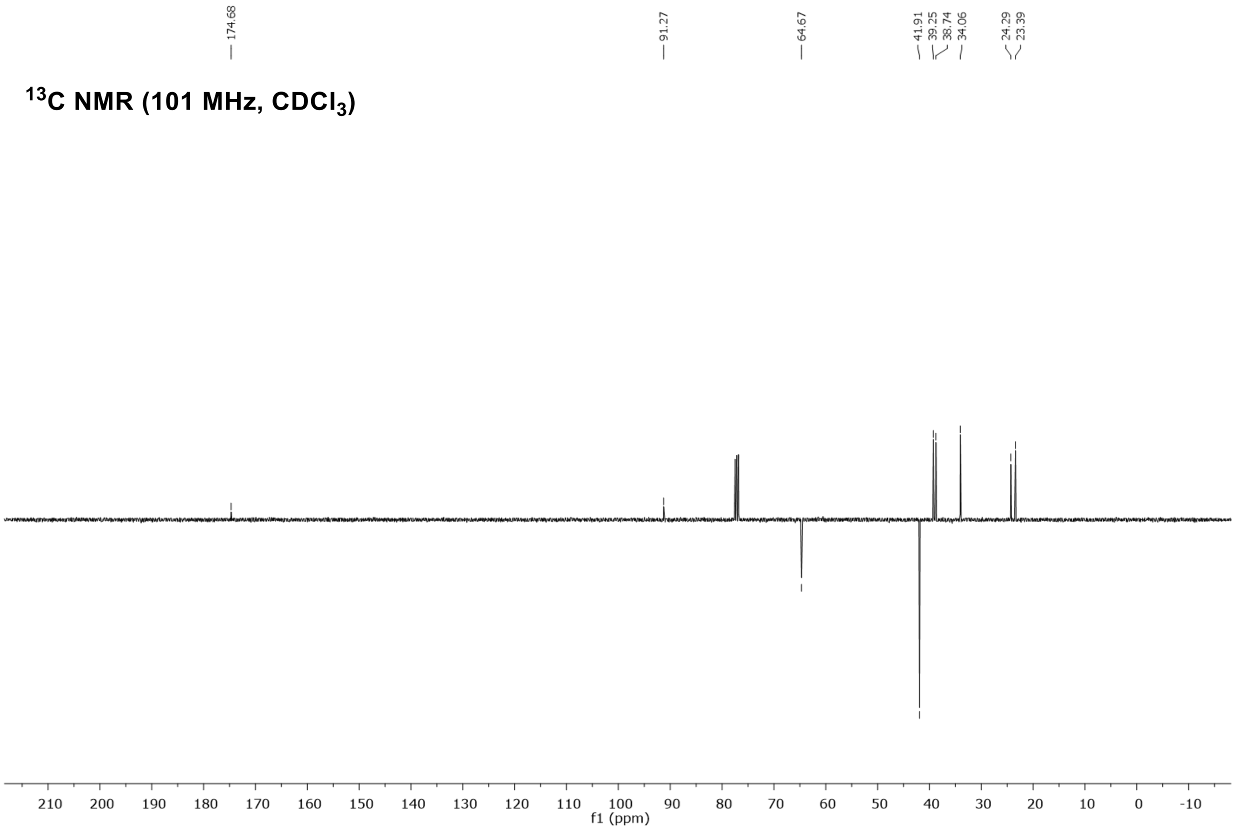

3-(Dimethylamino)-1-oxaspiro[4.4]dodecan-2-one (2d)

^{1}H NMR (400 MHz, $CDCl_3$)

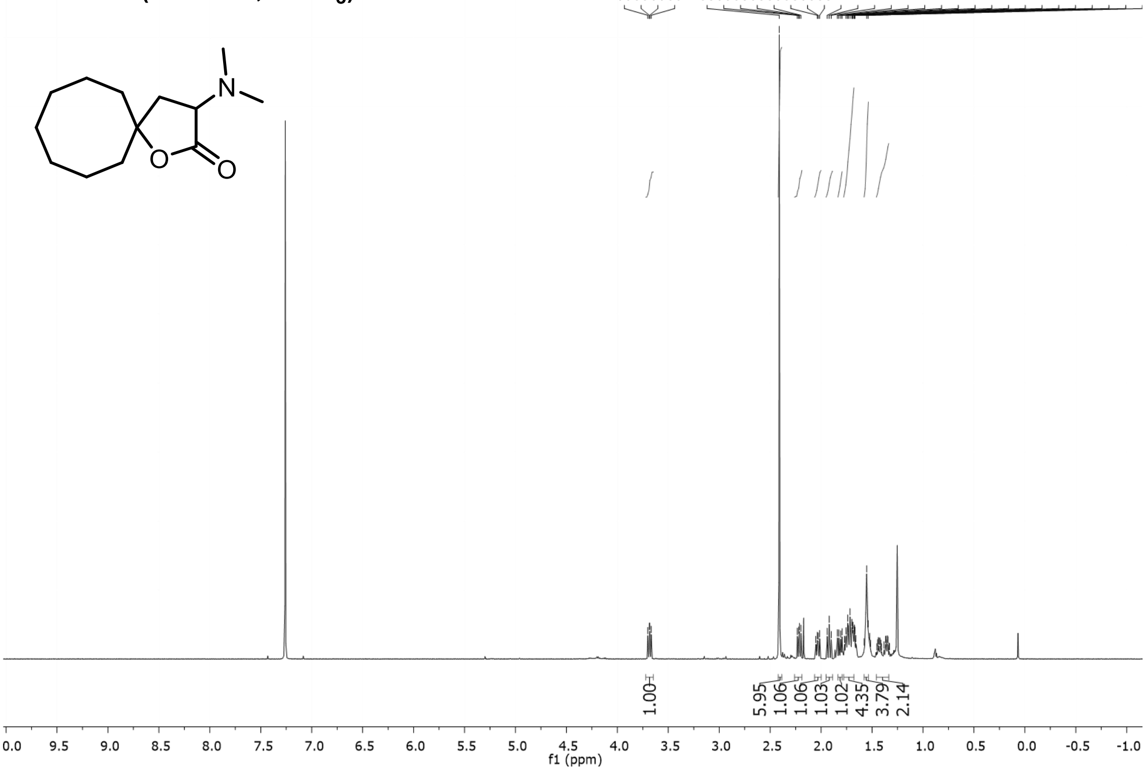

^{13}C NMR (101 MHz, $CDCl_3$)

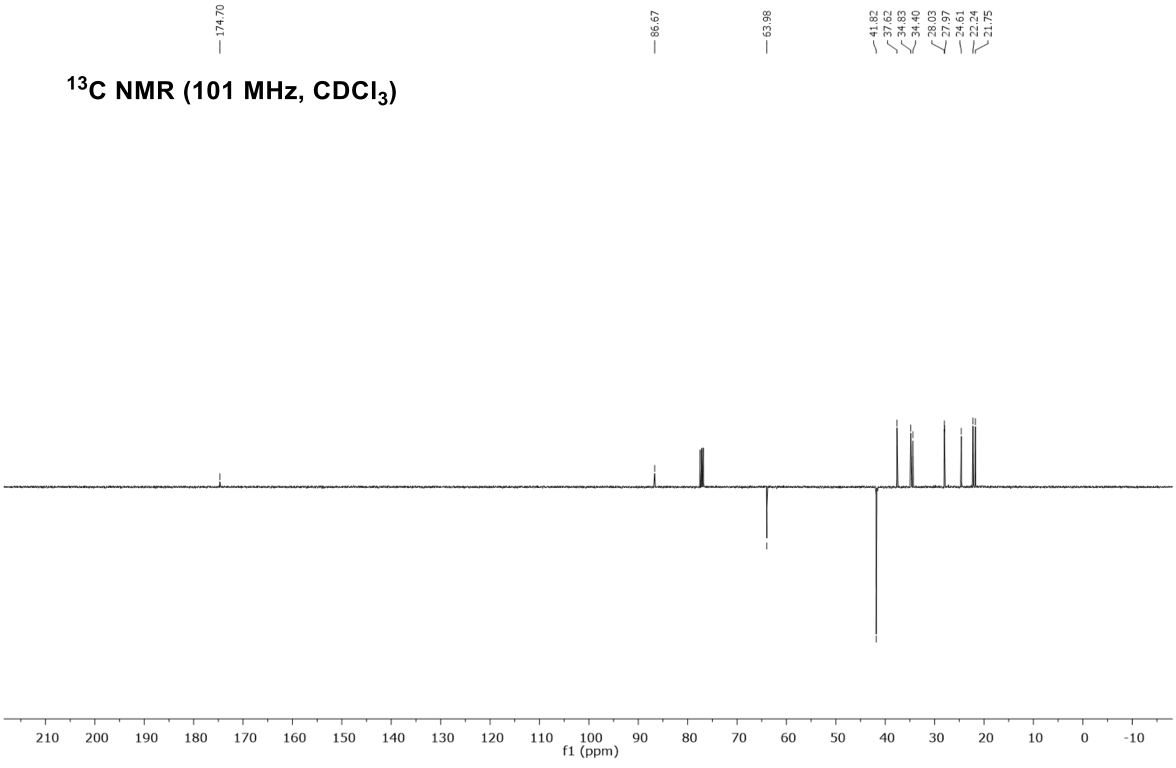

3-(Dimethylamino)-1-oxaspiro[4.4]nonadecan-2-one (2e)

¹H NMR (400 MHz, CDCl$_3$)

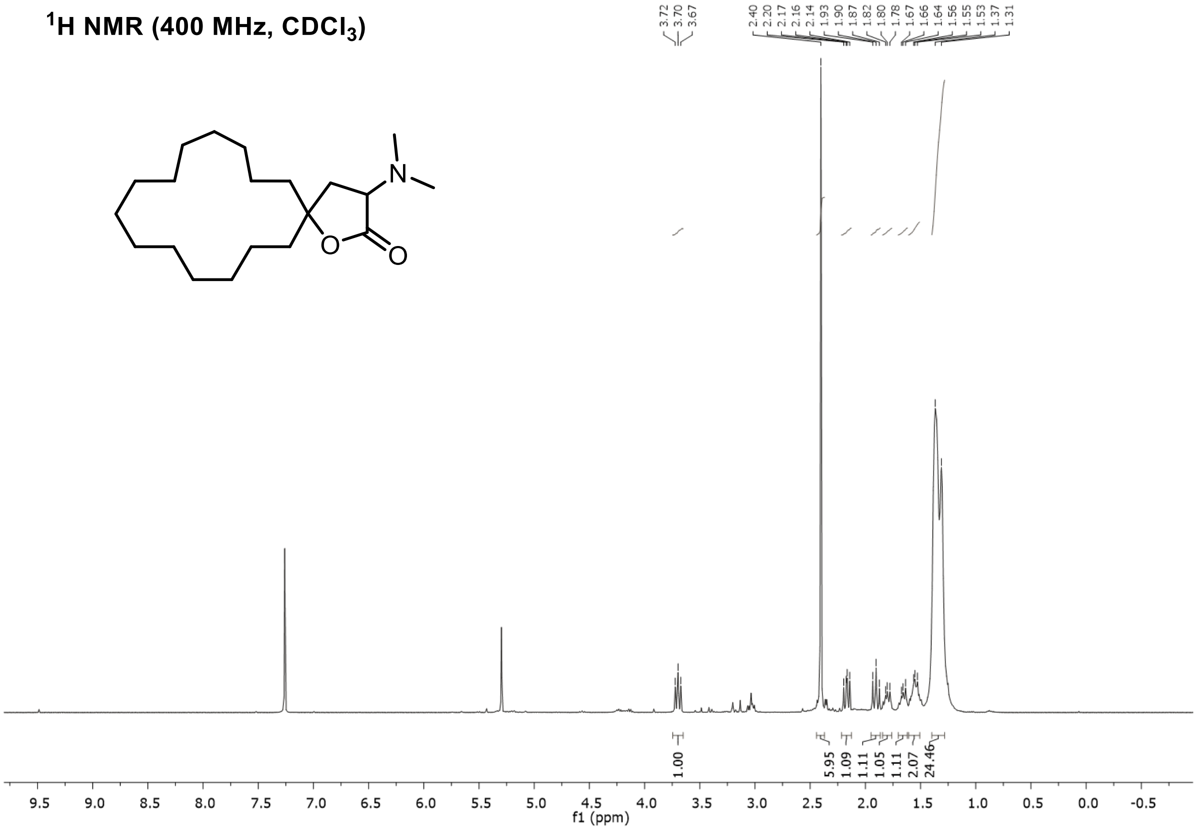

¹³C NMR (101 MHz, CDCl$_3$)

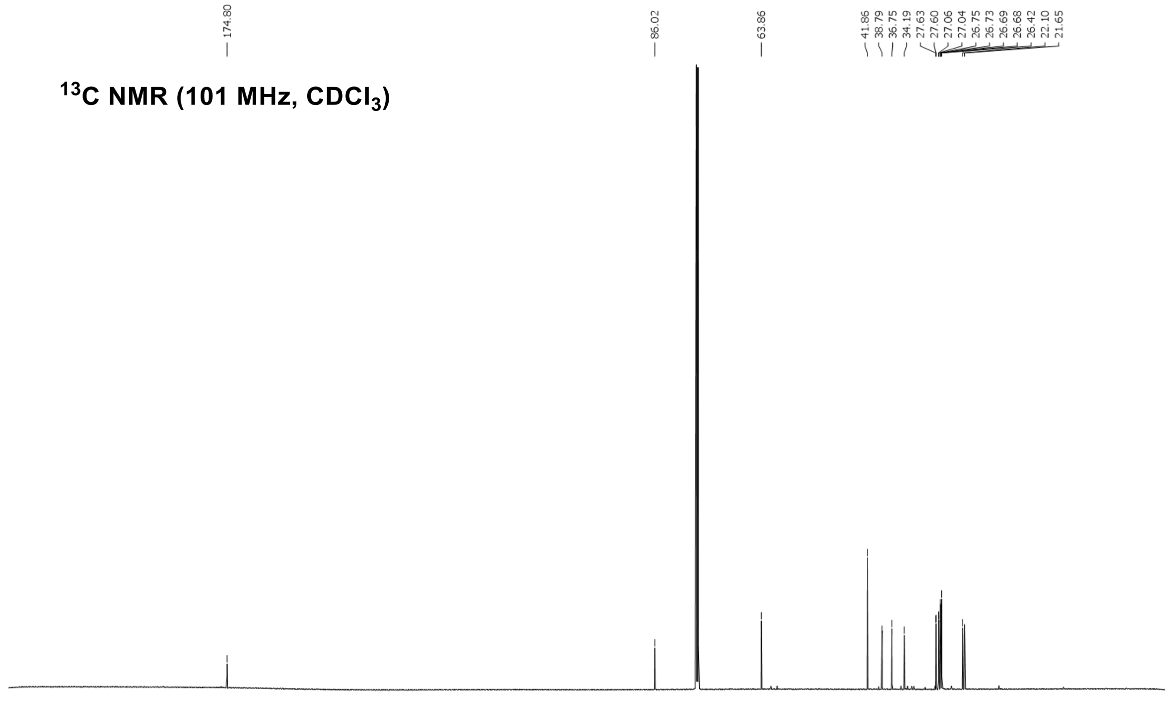

(3*S**,5*S**)-5-(*tert*-Butyl)-3-(dimethylamino)-5-methyldihydrofuran-2(3*H*)-one (2f)

^{1}H NMR (600 MHz, $CDCl_3$)

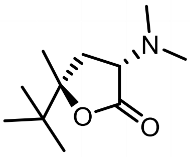

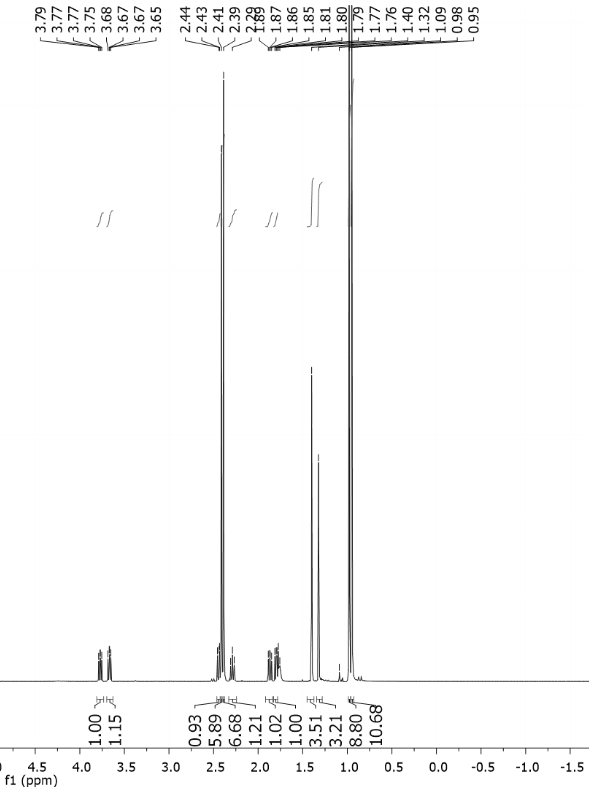

^{13}C NMR (151 MHz, $CDCl_3$)

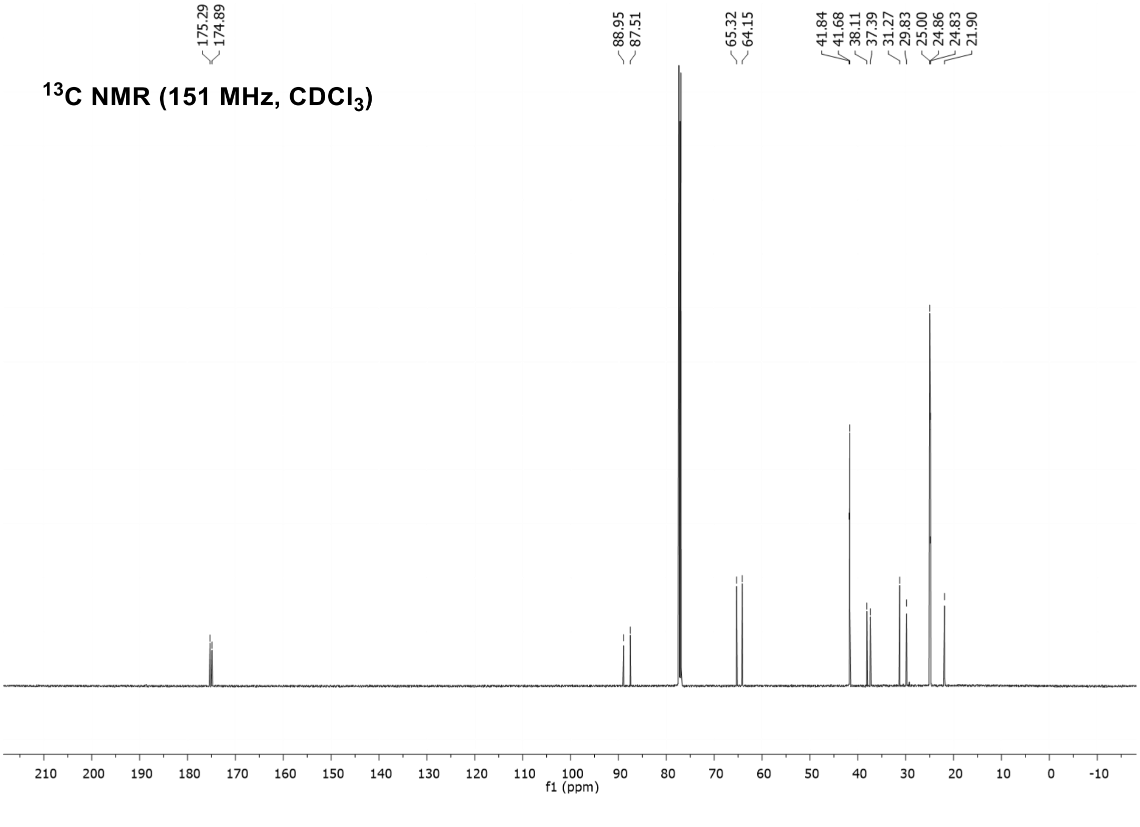

(3*S,5*R**)-3-(Dimethylamino)-5-phenyldihydrofuran-2(3*H*)-one**

(major diastereomer 2g)

^{1}H NMR (400 MHz, $CDCl_3$)

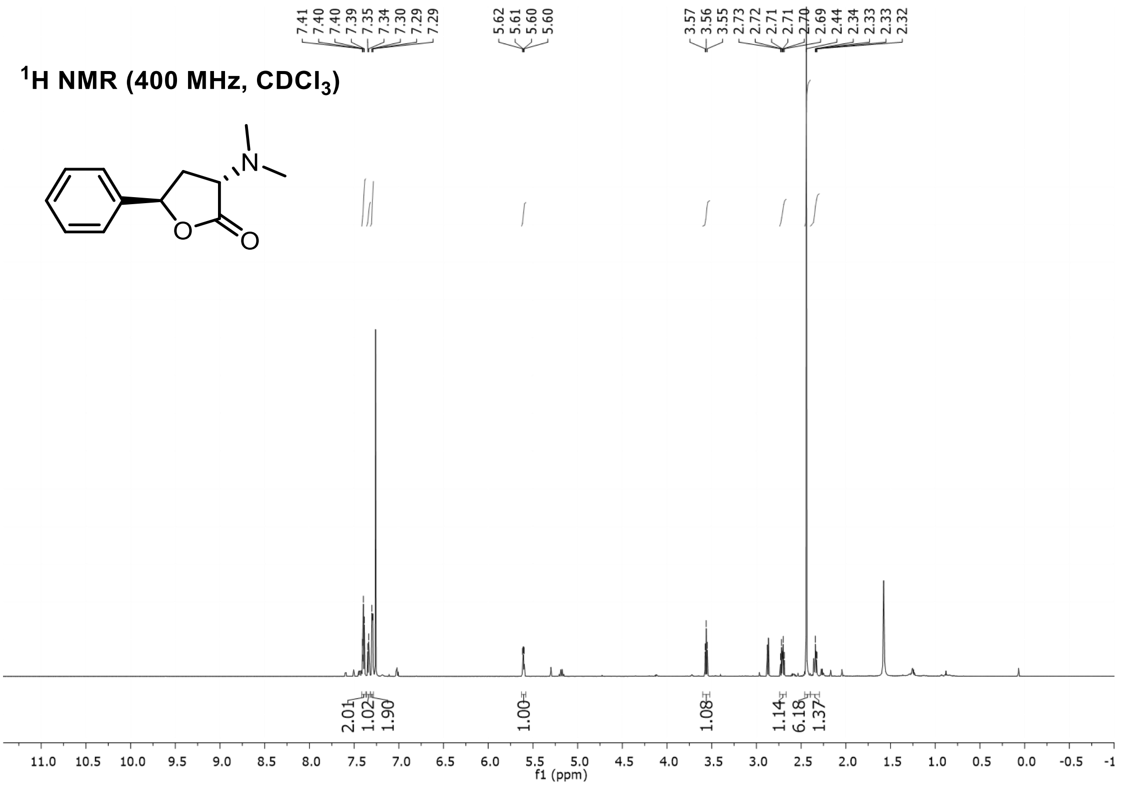

^{13}C NMR (101 MHz, $CDCl_3$)

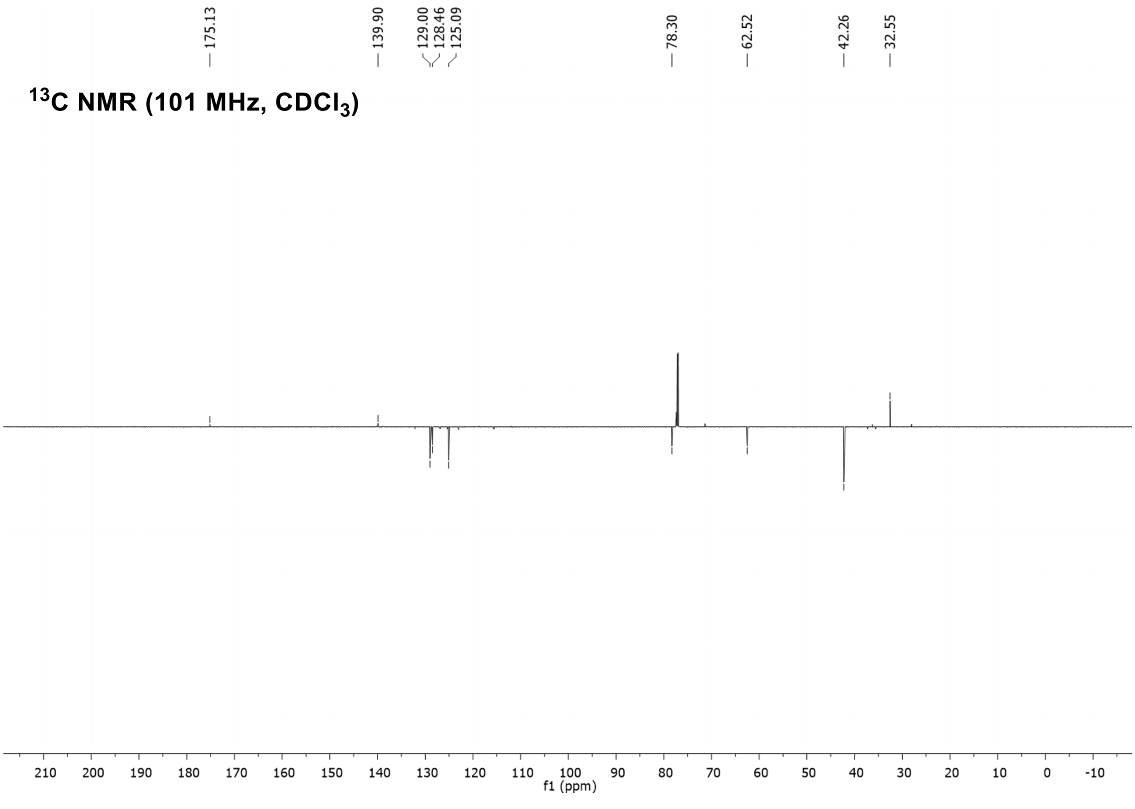

^{1}H NMR (400 MHz, CDCl$_3$)

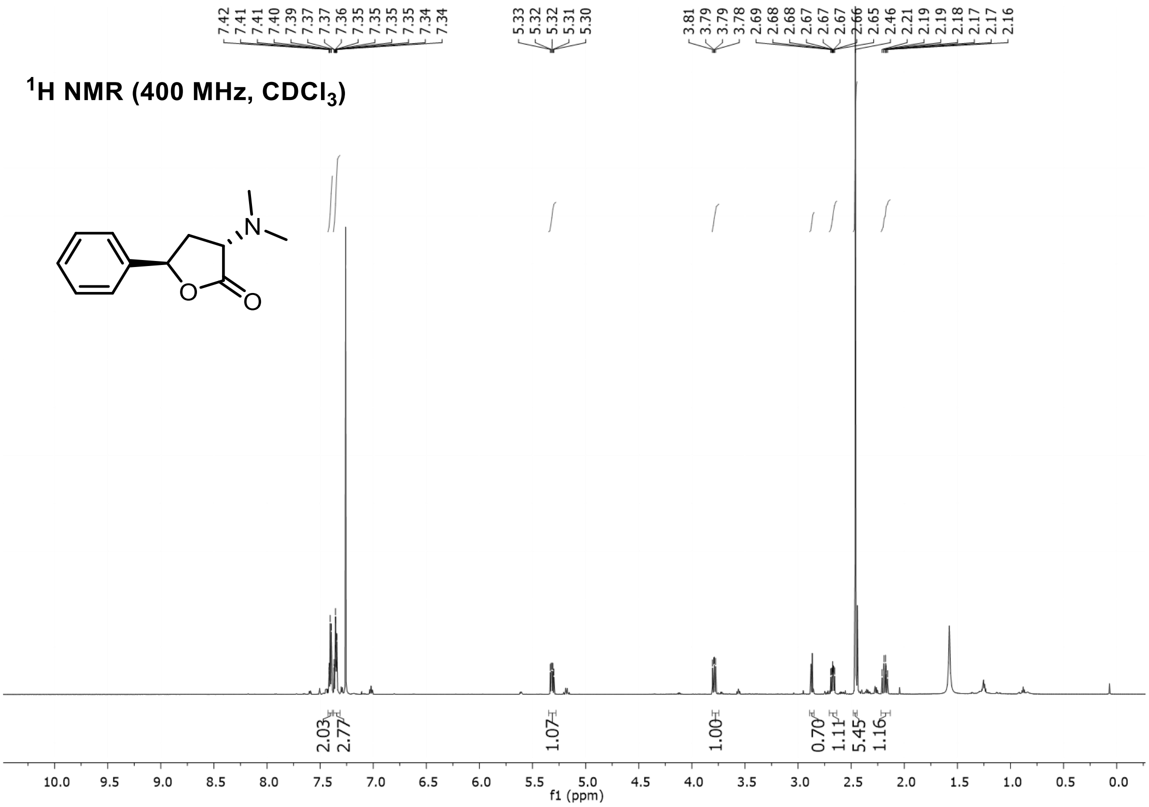

^{13}C NMR (101 MHz, CDCl$_3$)

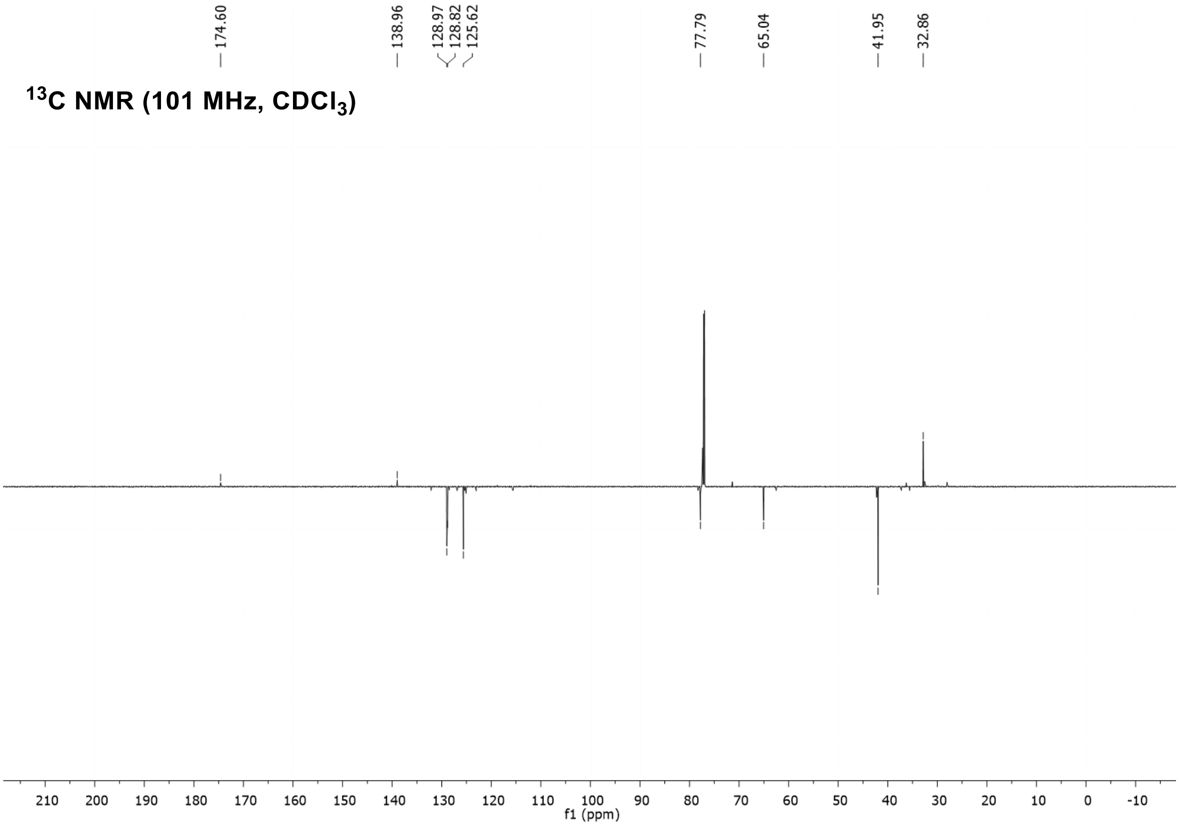

(3*S**,5*s**,8*R**)-3-(Dimethylamino)-8-methyl-1-oxaspiro[4.5]decan-2-one

(major diastereomer 2h)

^{1}H NMR (400 MHz, $CDCl_3$)

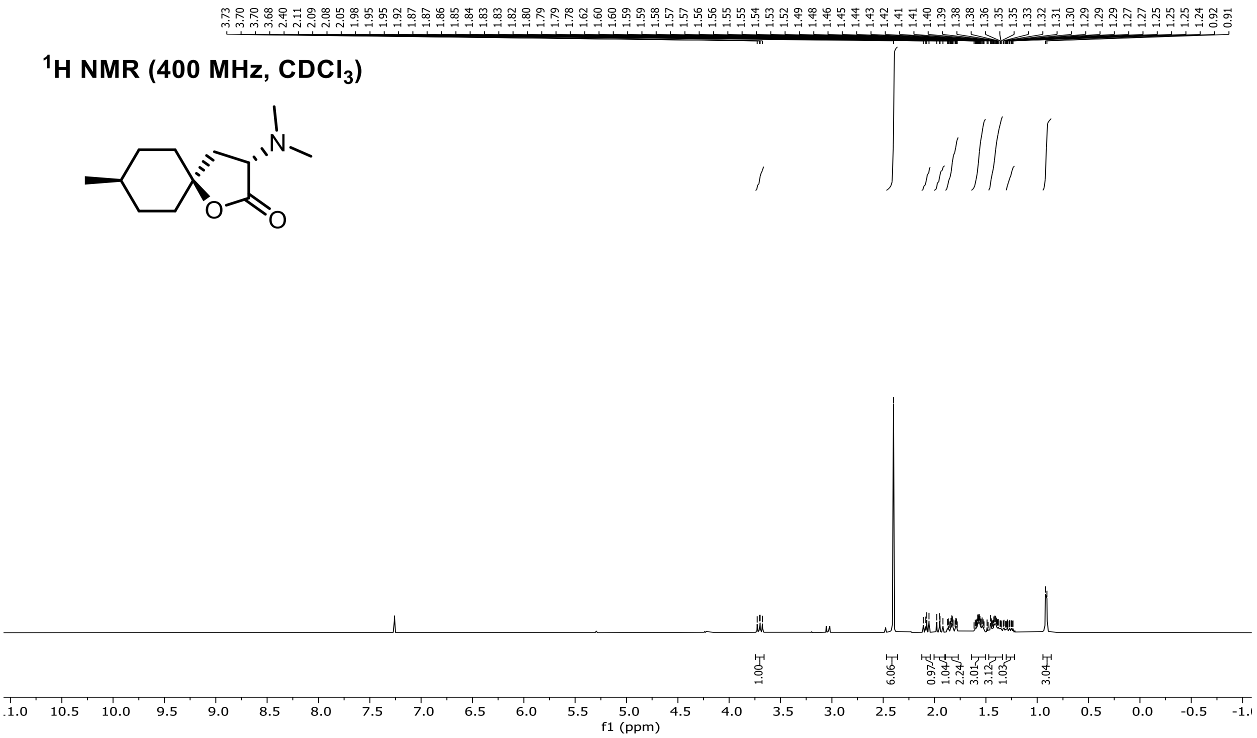

^{13}C NMR (101 MHz, $CDCl_3$)

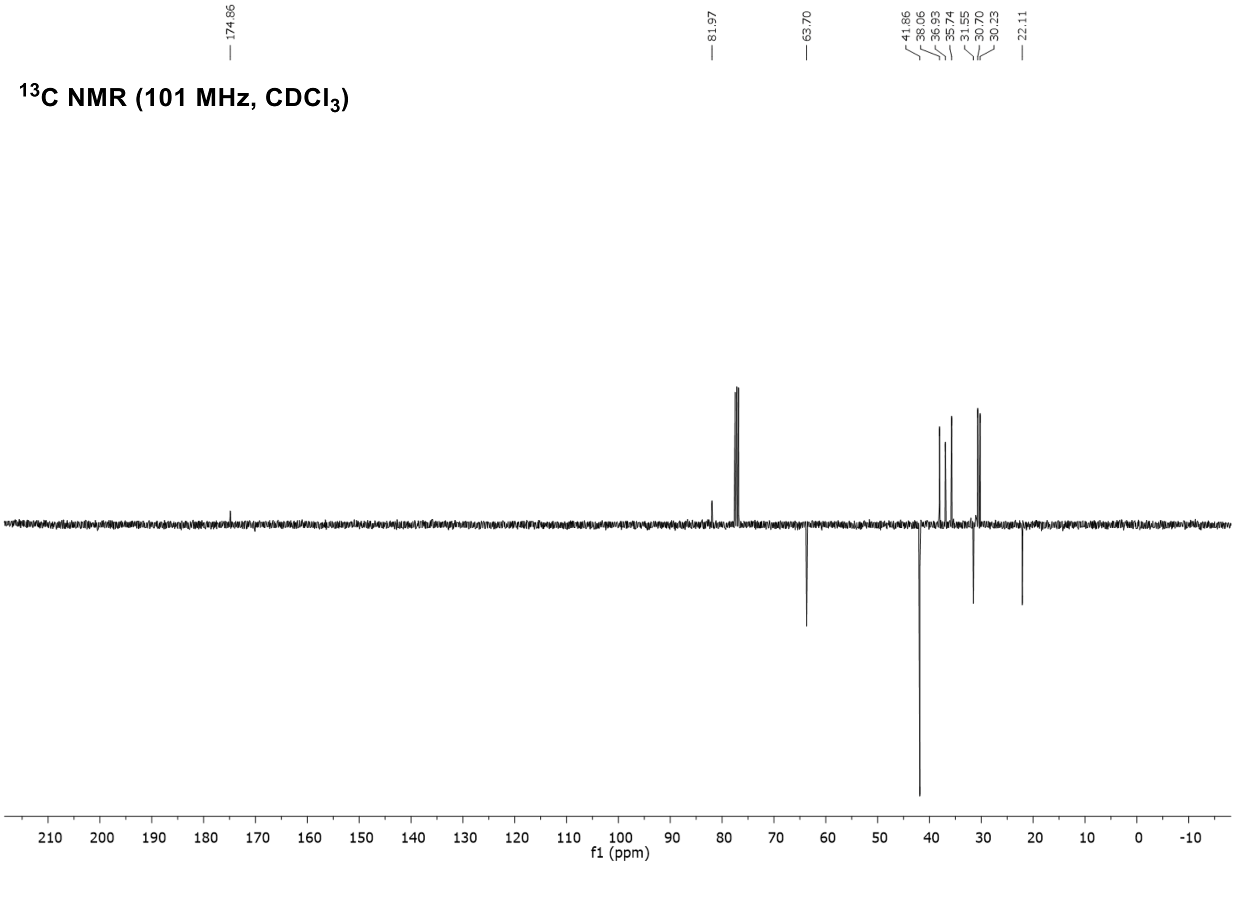

^{1}H NMR (400 MHz, CDCl$_3$)

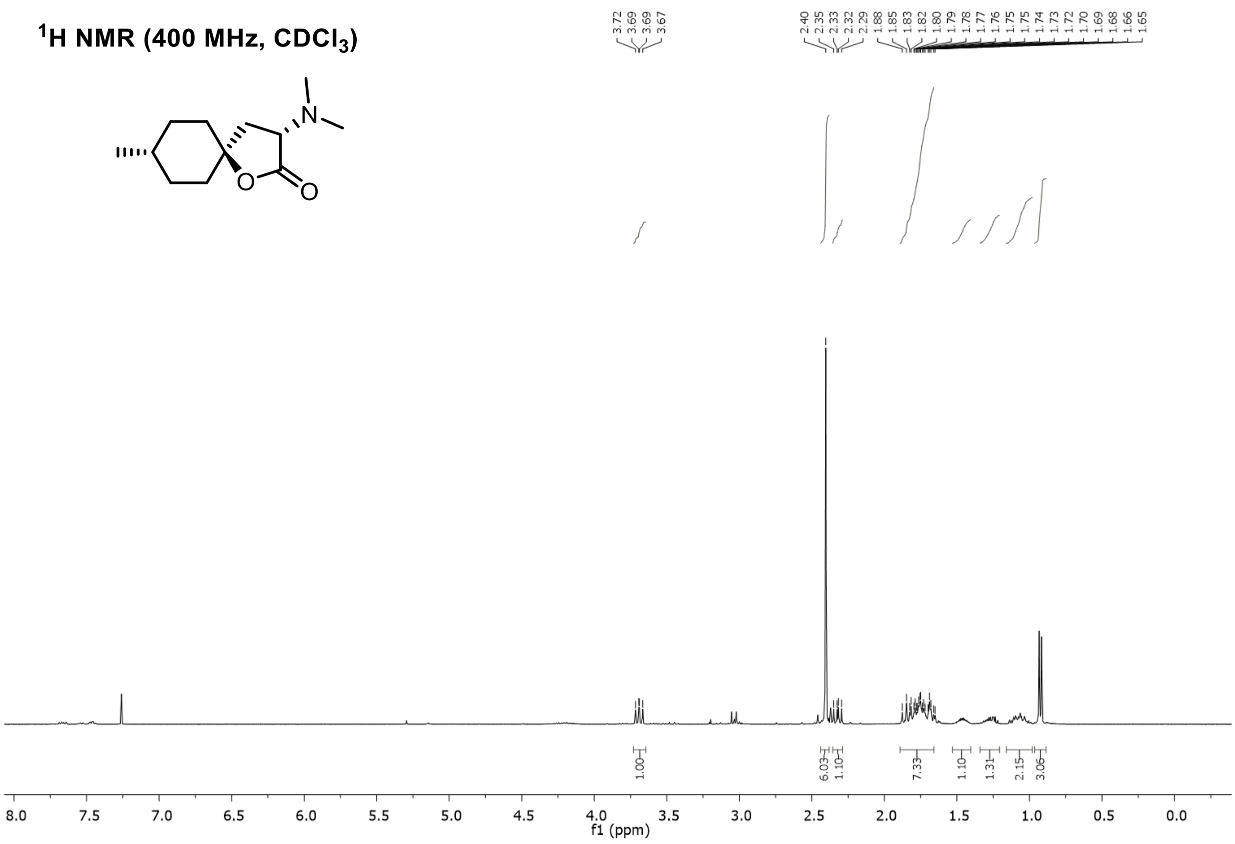

^{13}C NMR (101 MHz, CDCl$_3$)

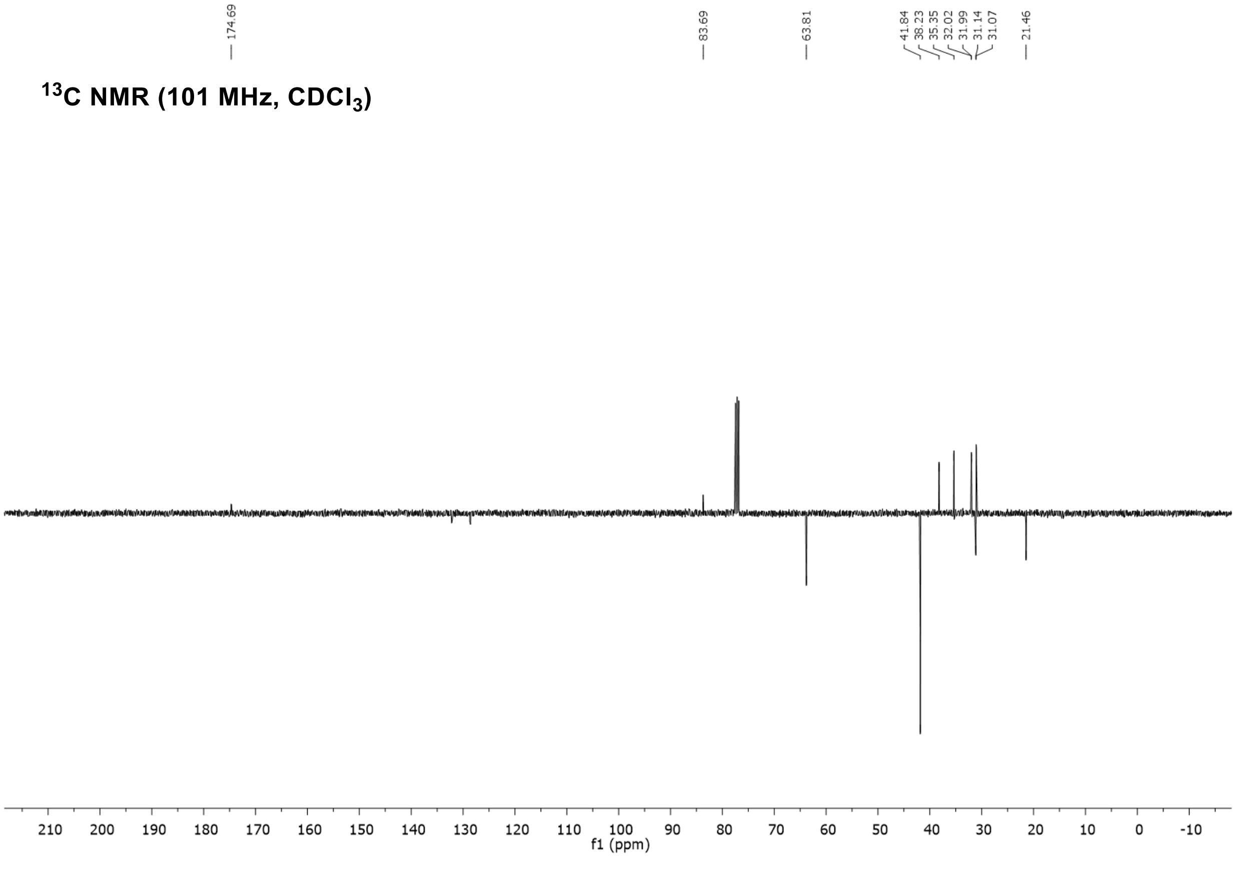

(3*S**,5*s**,8*R**)-8-(*tert*-Butyl)-3-(dimethylamino)-1-oxaspiro[4.5]decan-2-one (2i)

^{1}H NMR (600 MHz, $CDCl_3$)

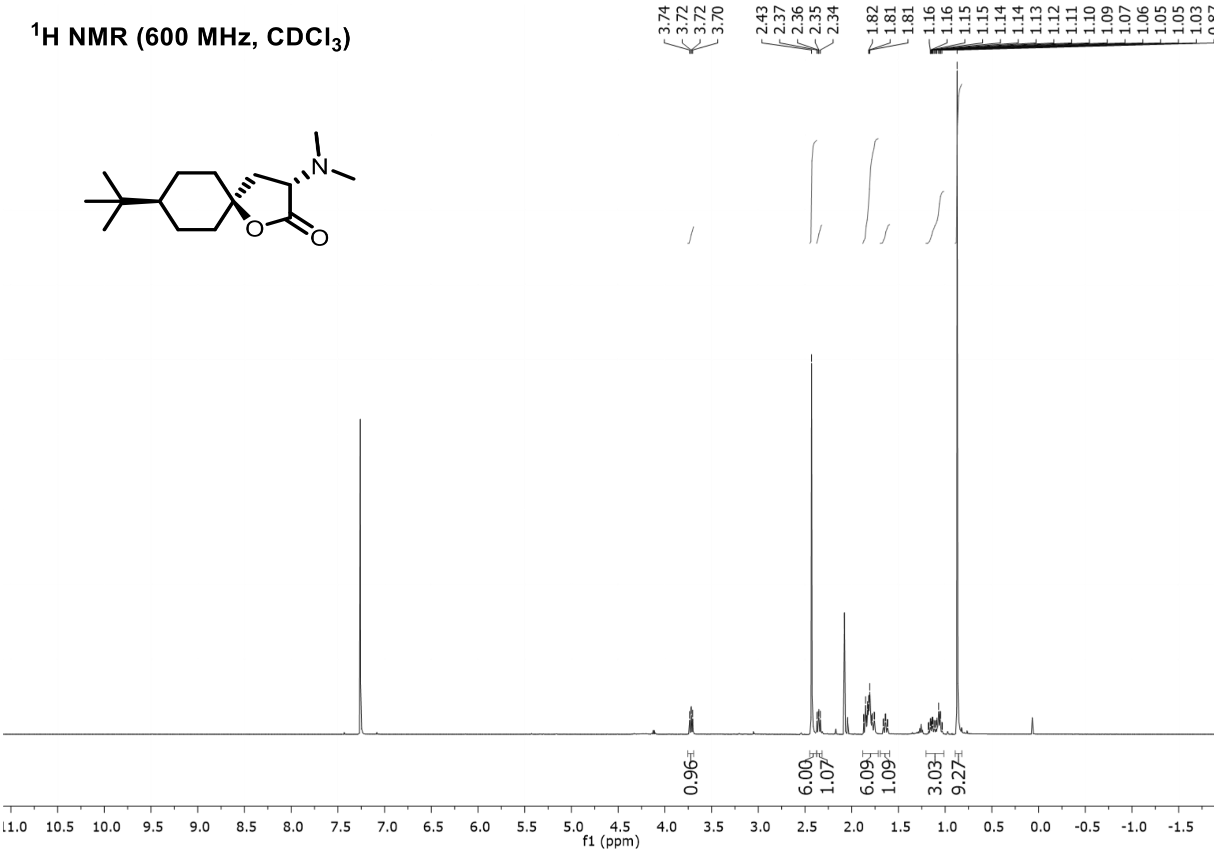

^{13}C NMR (151 MHz, $CDCl_3$)

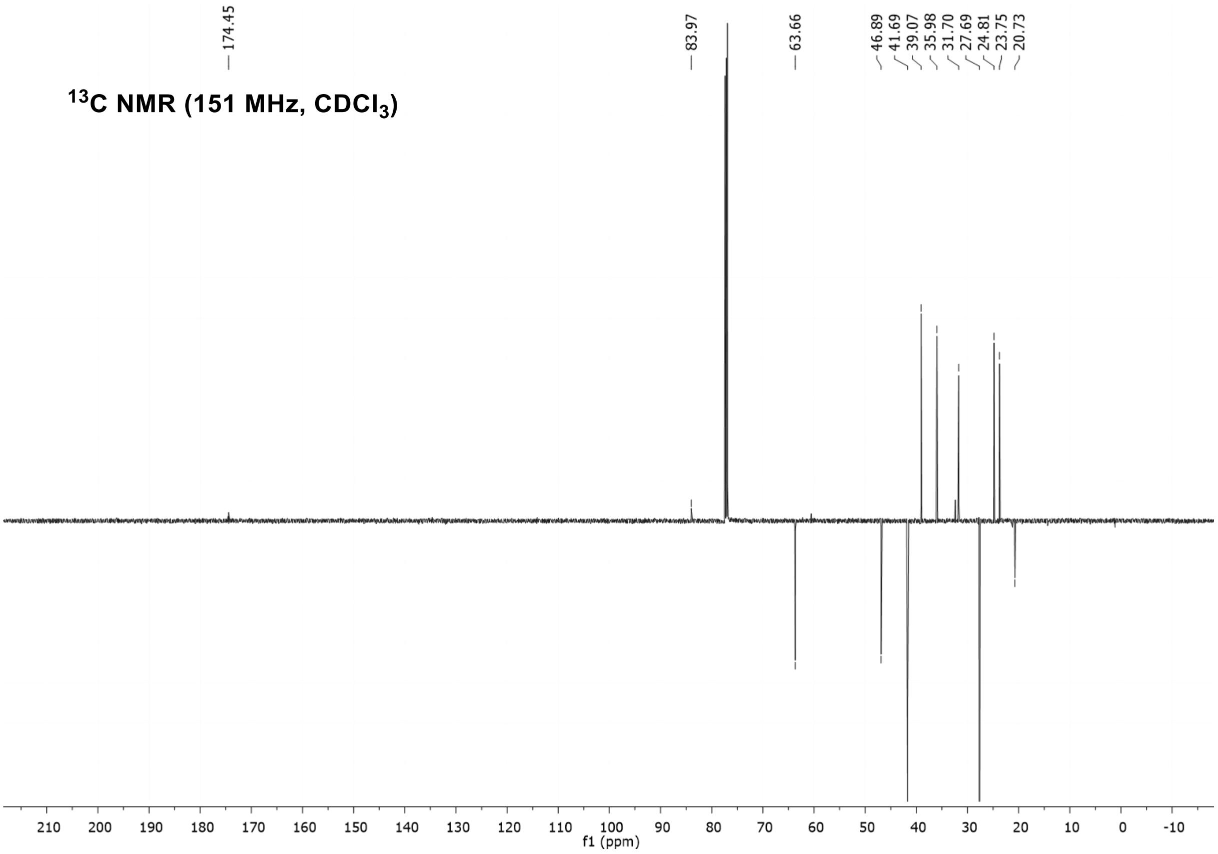

(3*S**,5*s**,8*R**)-3-(Dimethylamino)-8-phenyl-1-oxaspiro[4.5]decan-2-one

(major diastereomer 2j)

^{1}H NMR (400 MHz, $CDCl_3$)

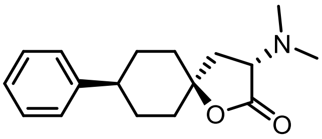

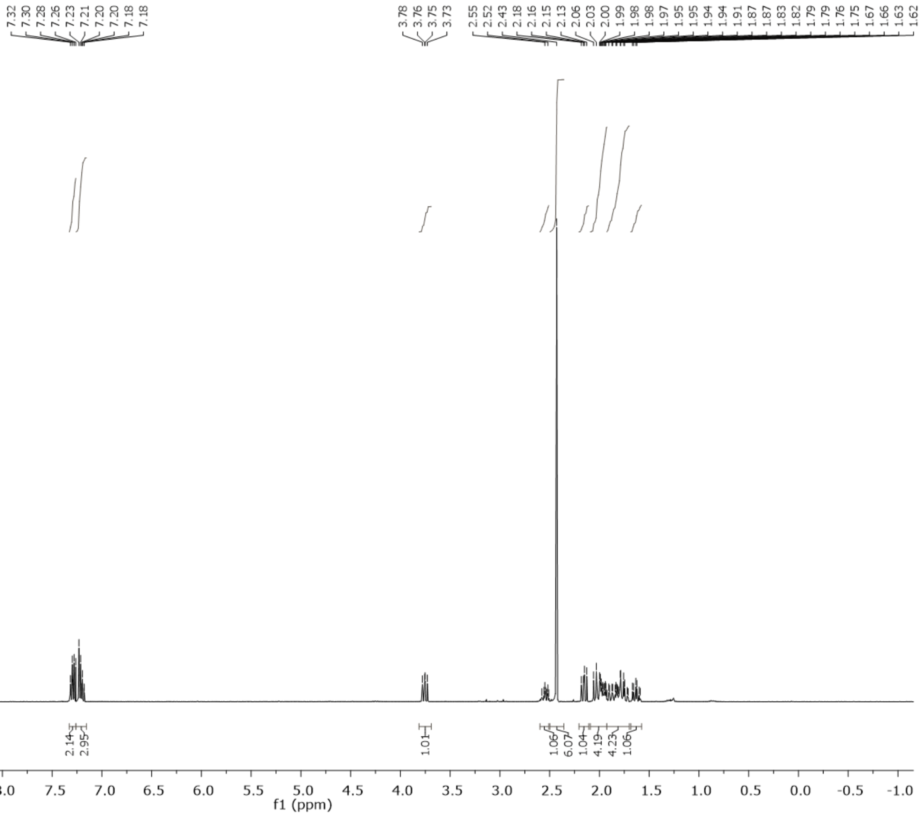

^{13}C NMR (101 MHz, $CDCl_3$)

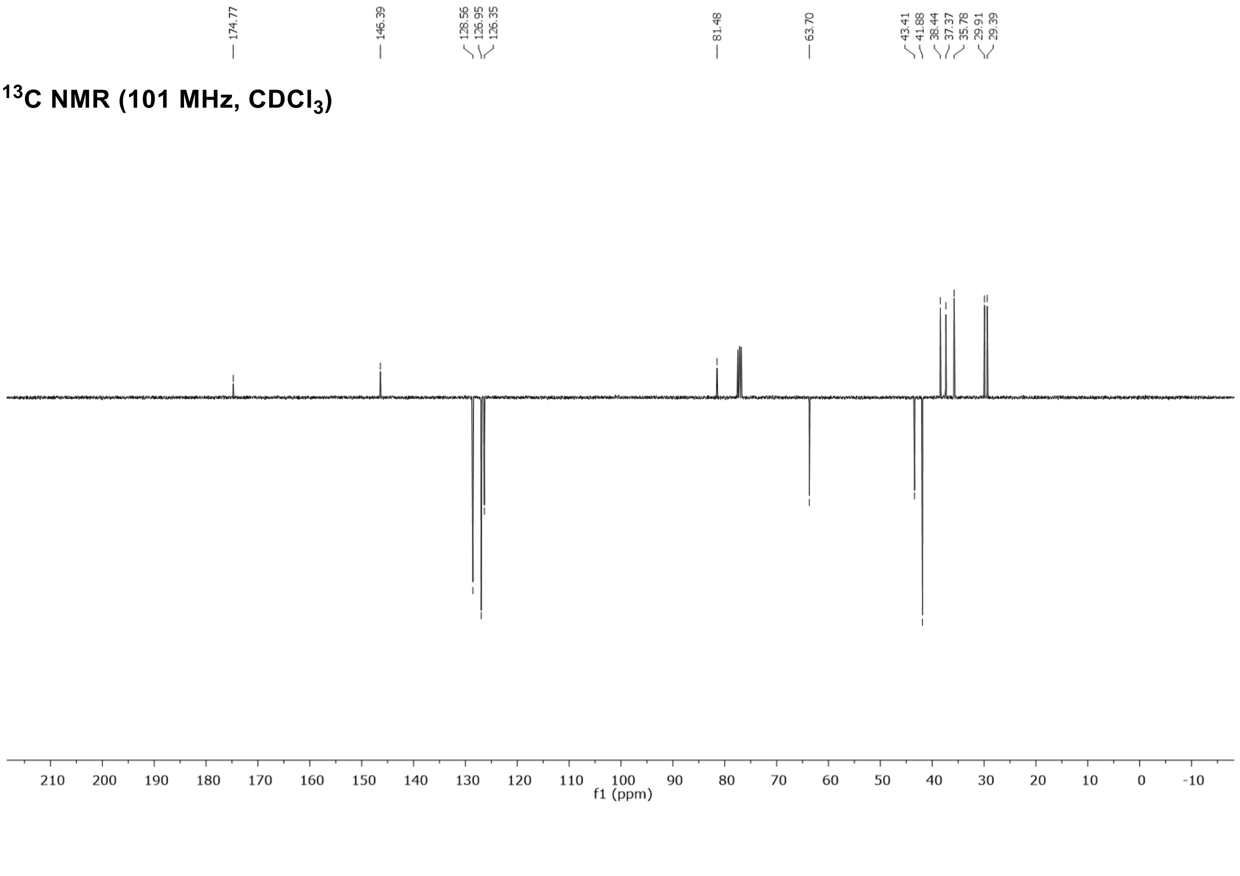

^{1}H NMR (400 MHz, CDCl$_3$)

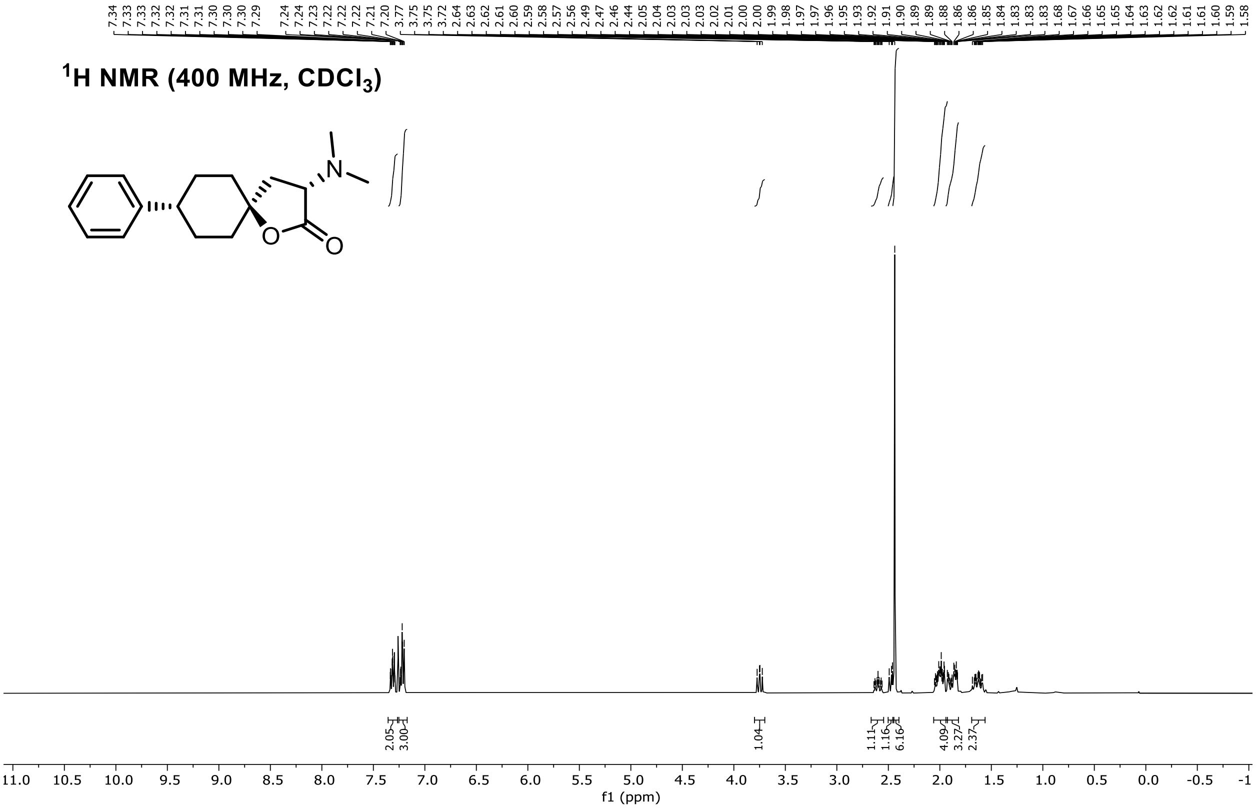

^{13}C NMR (101 MHz, CDCl$_3$)

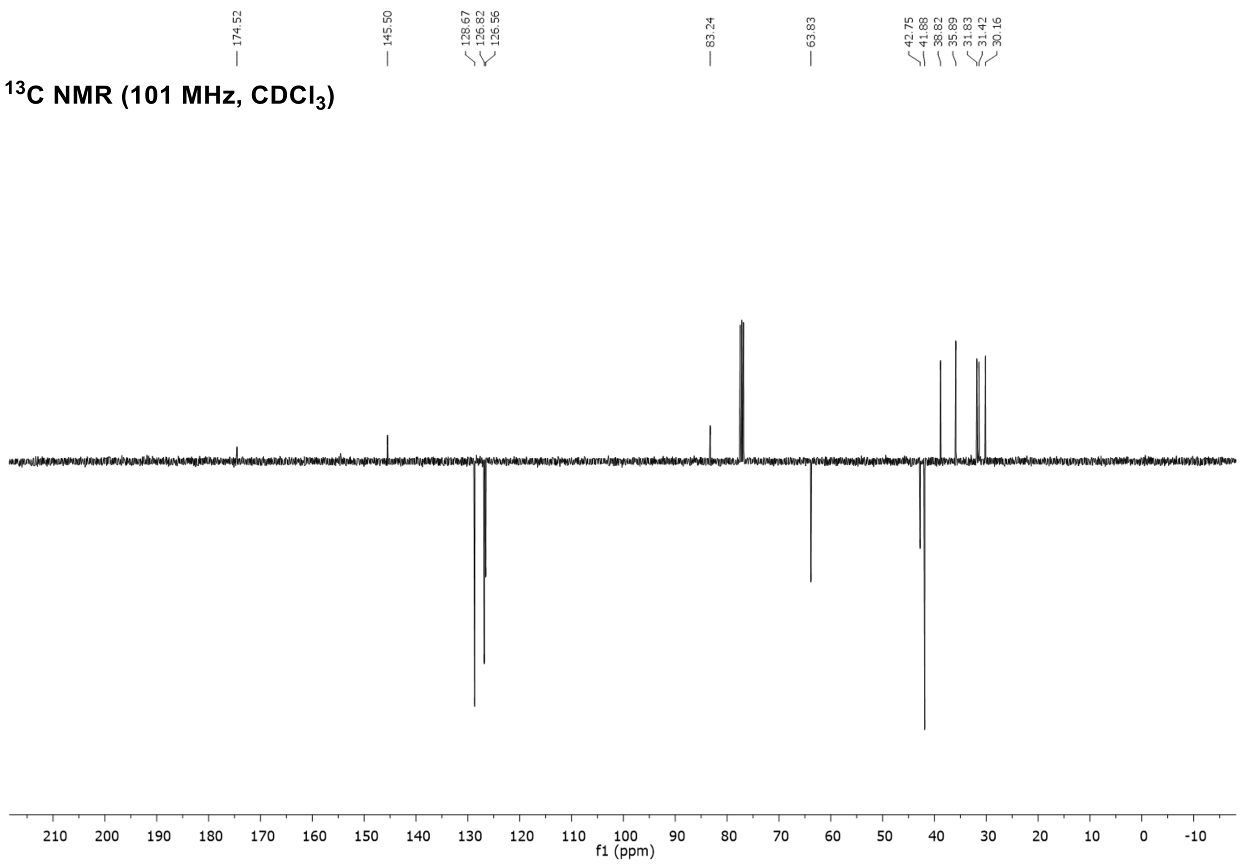

(3*S**,5*s**,8*R**)-3-(Dimethylamino)-8-(trifluoromethyl)-1-oxaspiro[4.5]decan-2-one (major diastereomer 2k)

^{1}H NMR (600 MHz, $CDCl_3$)

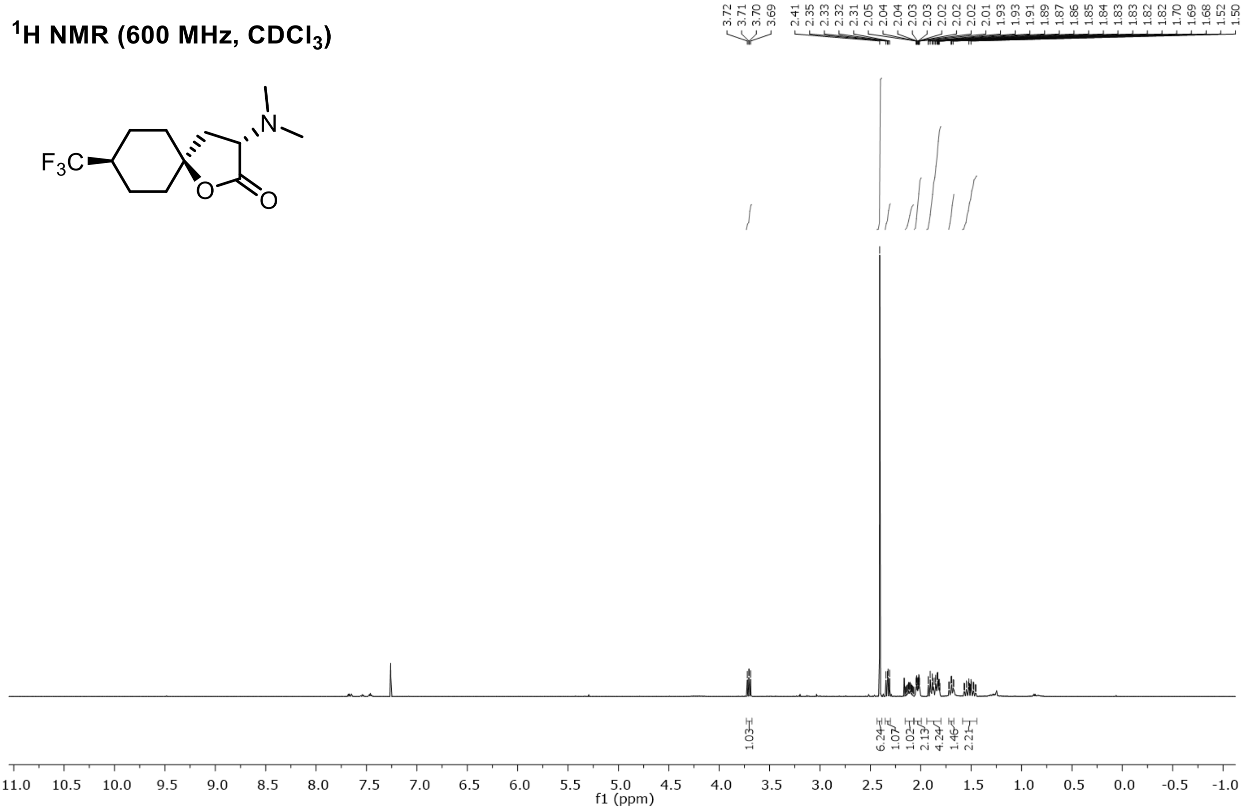

^{13}C NMR (151 MHz, $CDCl_3$)

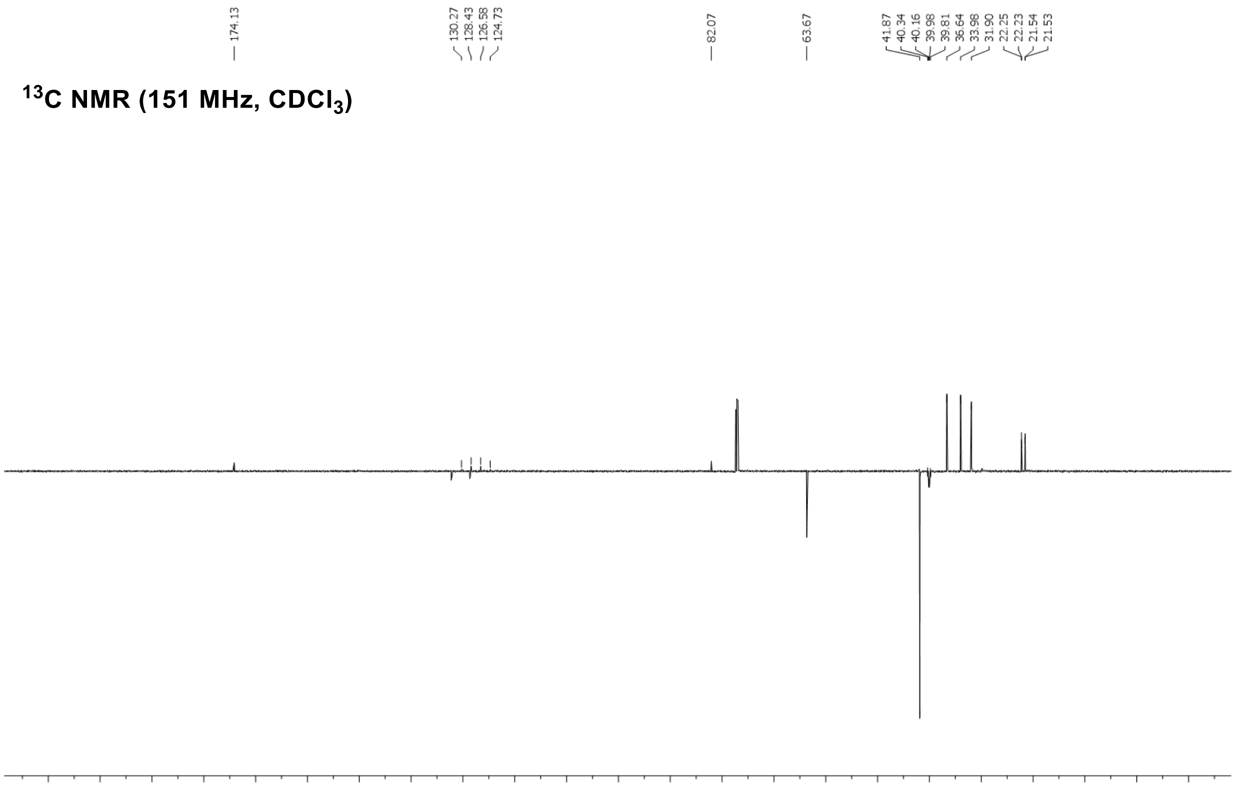

^{19}F NMR (376 MHz, $CDCl_3$)

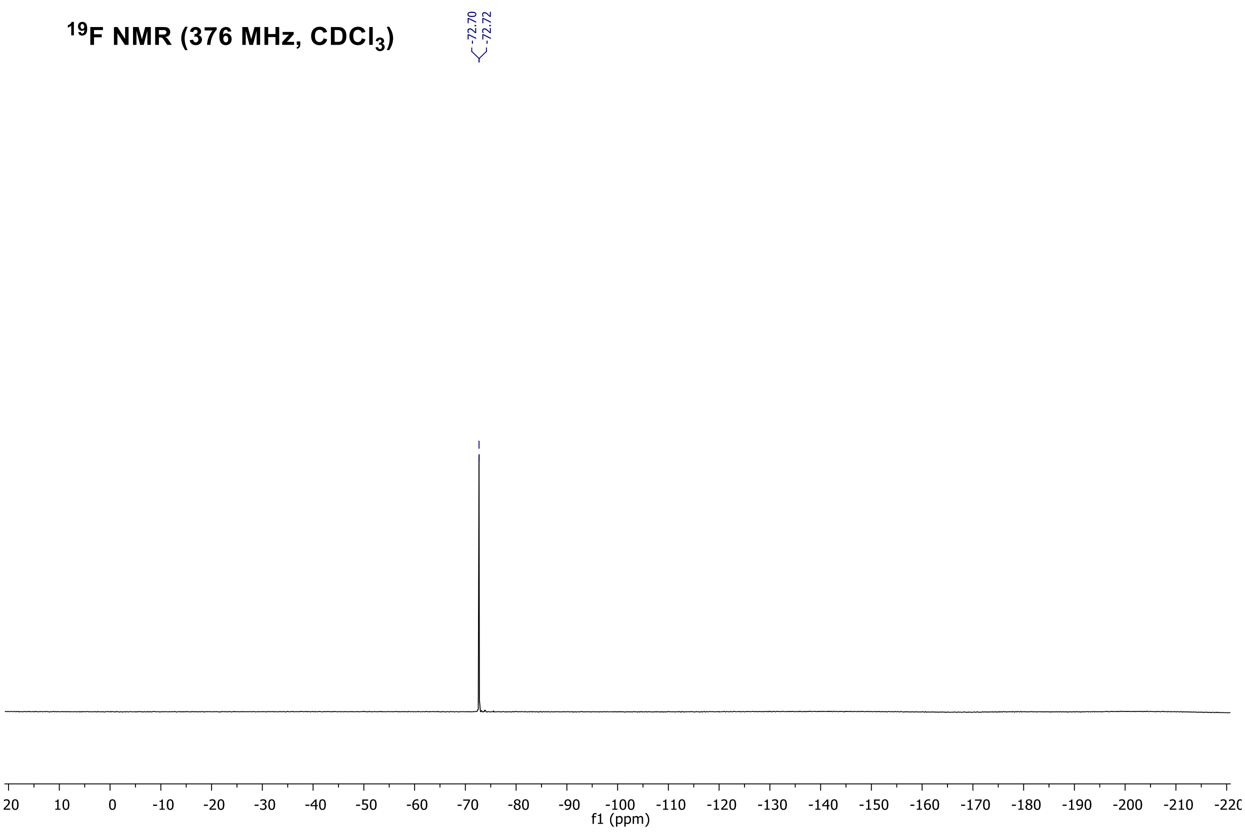

^{1}H NMR (400 MHz, $CDCl_3$)

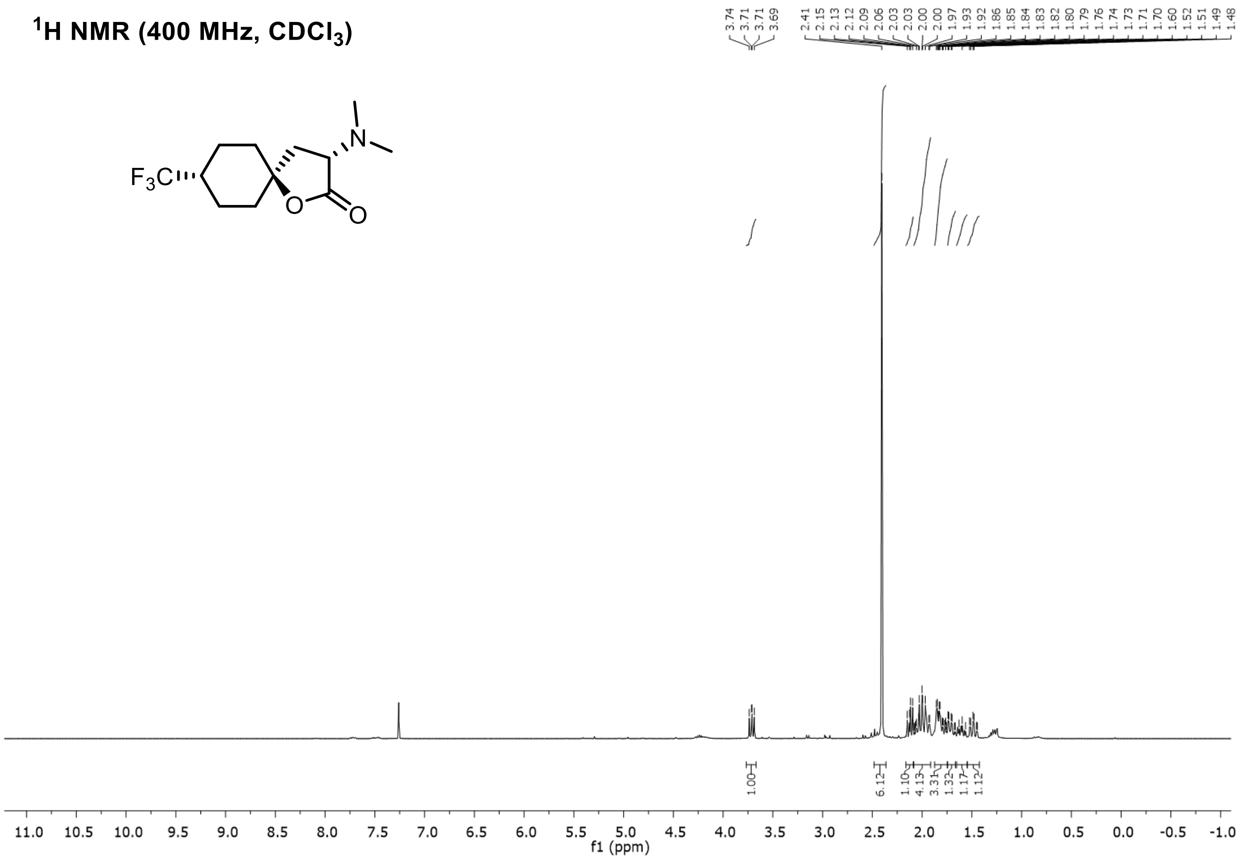

^{13}C NMR (101 MHz, $CDCl_3$)

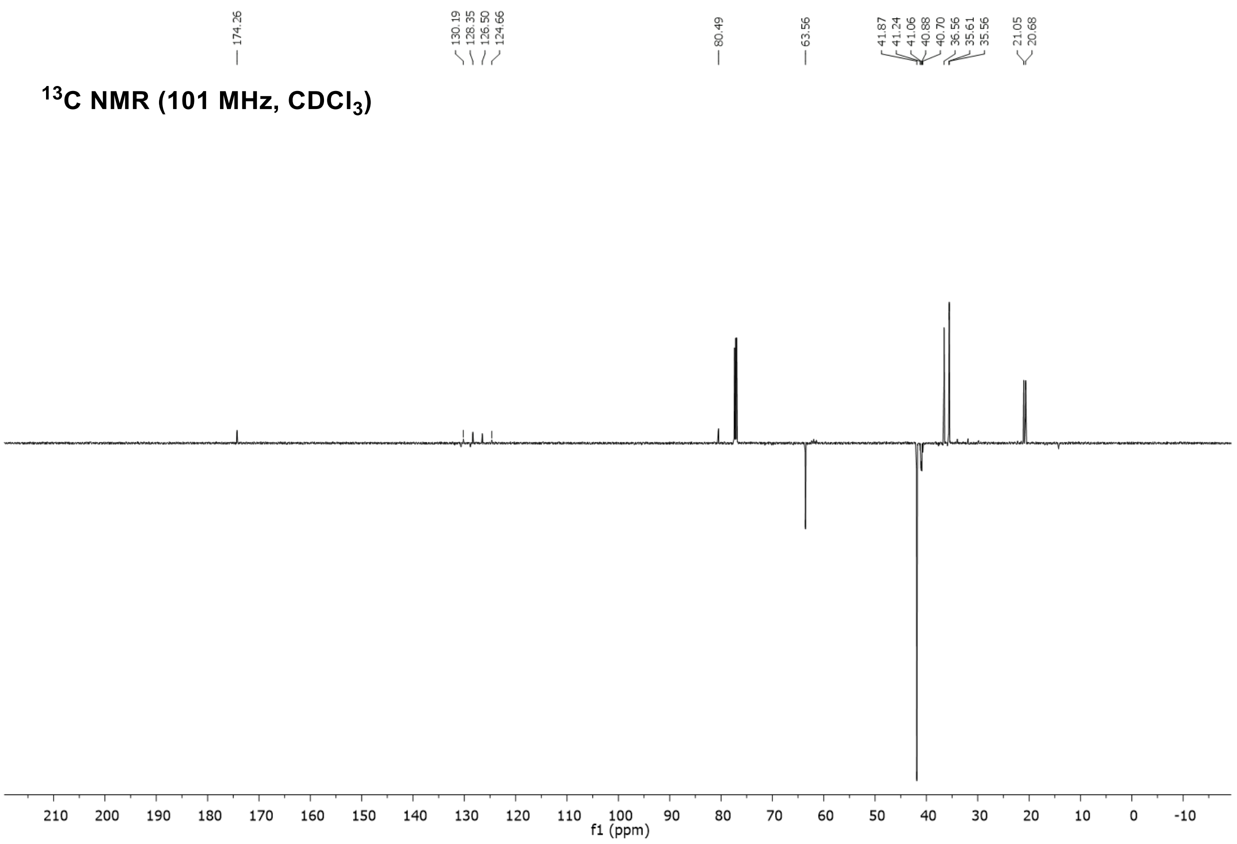

^{19}F NMR (376 MHz, $CDCl_3$)

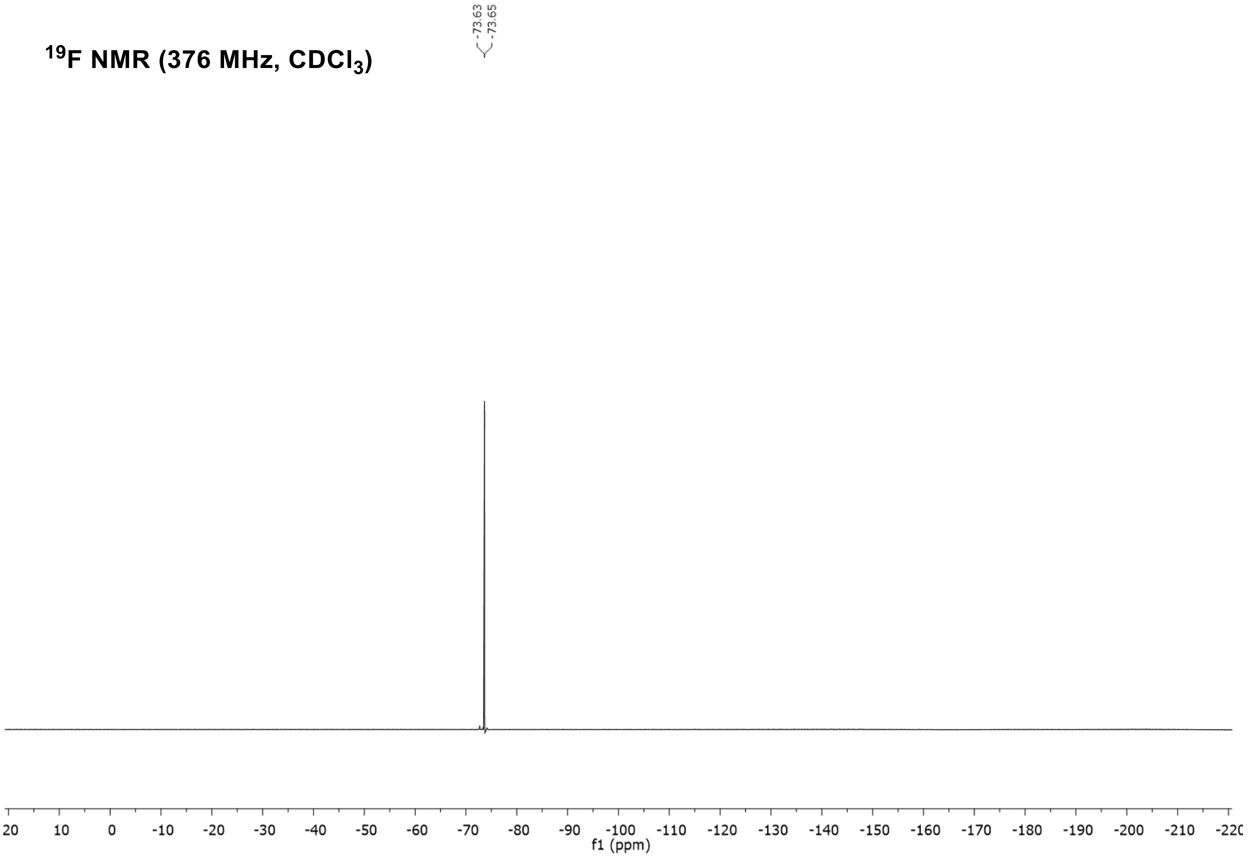

(3*S**,5*S**,6*S**)-3-(Dimethylamino)-6-phenyl-1-oxaspiro[4.5]decan-2-one (2l)

^{1}H NMR (600 MHz, CDCl$_3$)

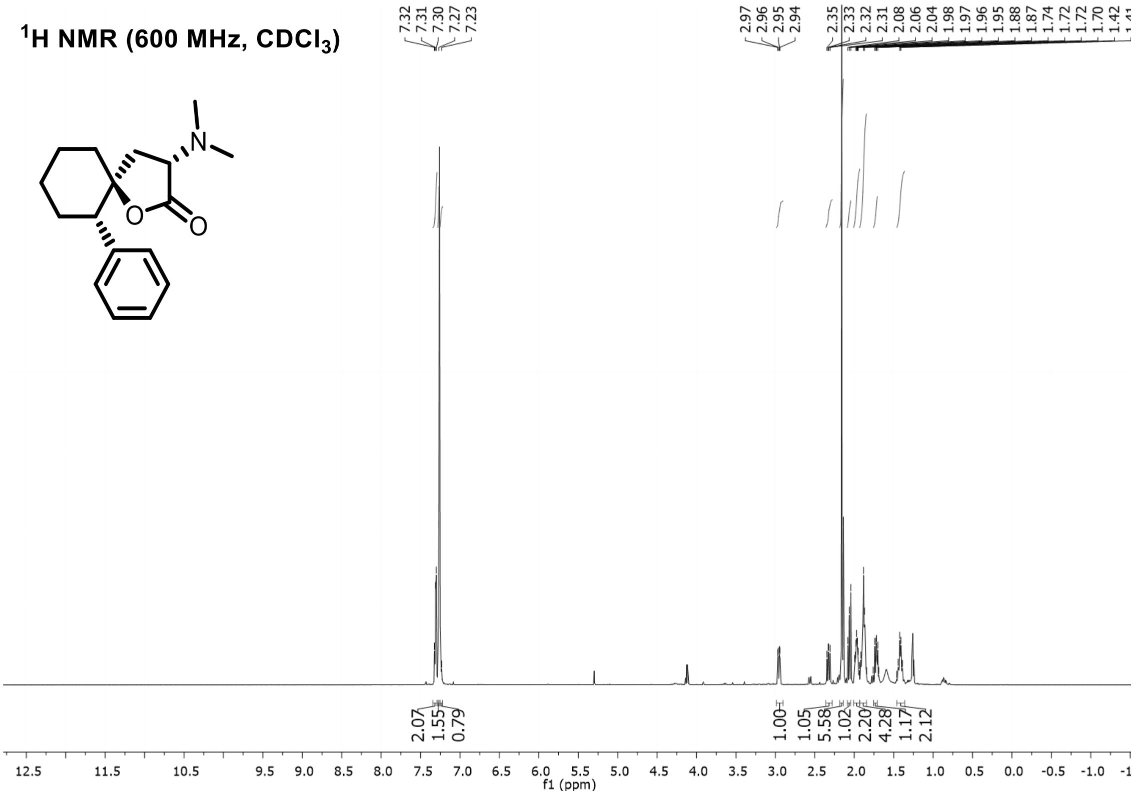

^{13}C NMR (151 MHz, CDCl$_3$)

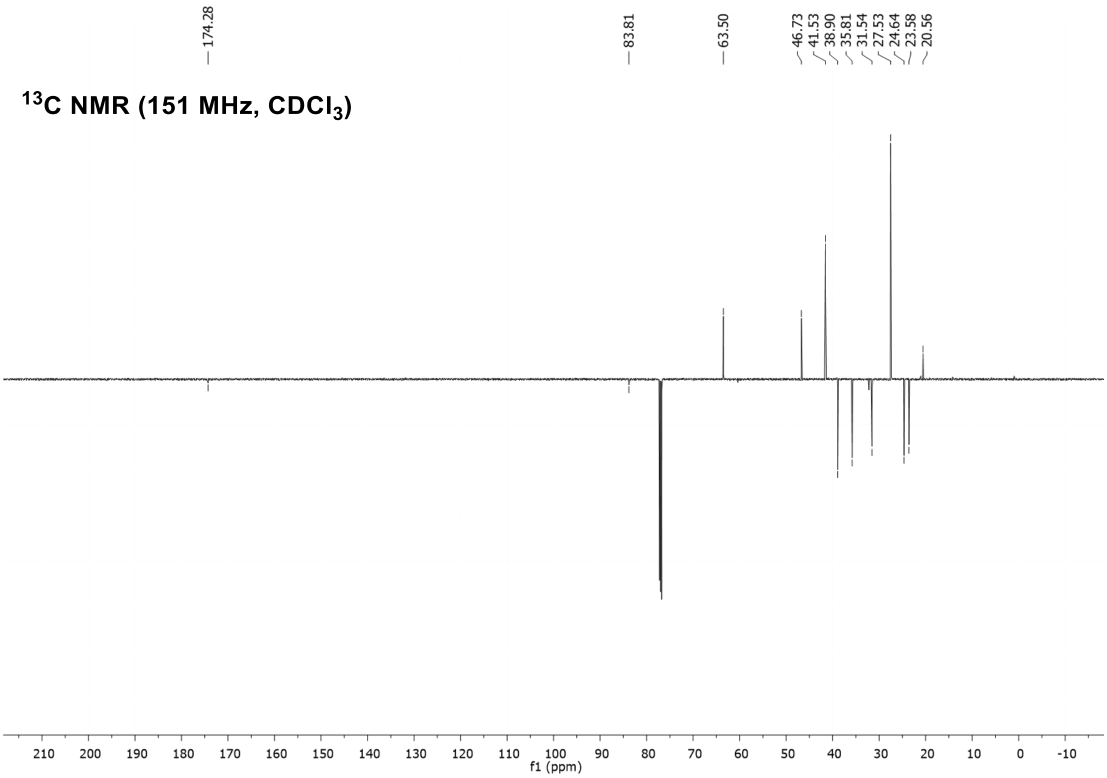

(3*S**,5S*,6*S**)-6-Cyclohexyl-3-(dimethylamino)-1-oxaspiro[4.5]decan-2-one (2m)

^{1}H NMR (400 MHz, $CDCl_3$)

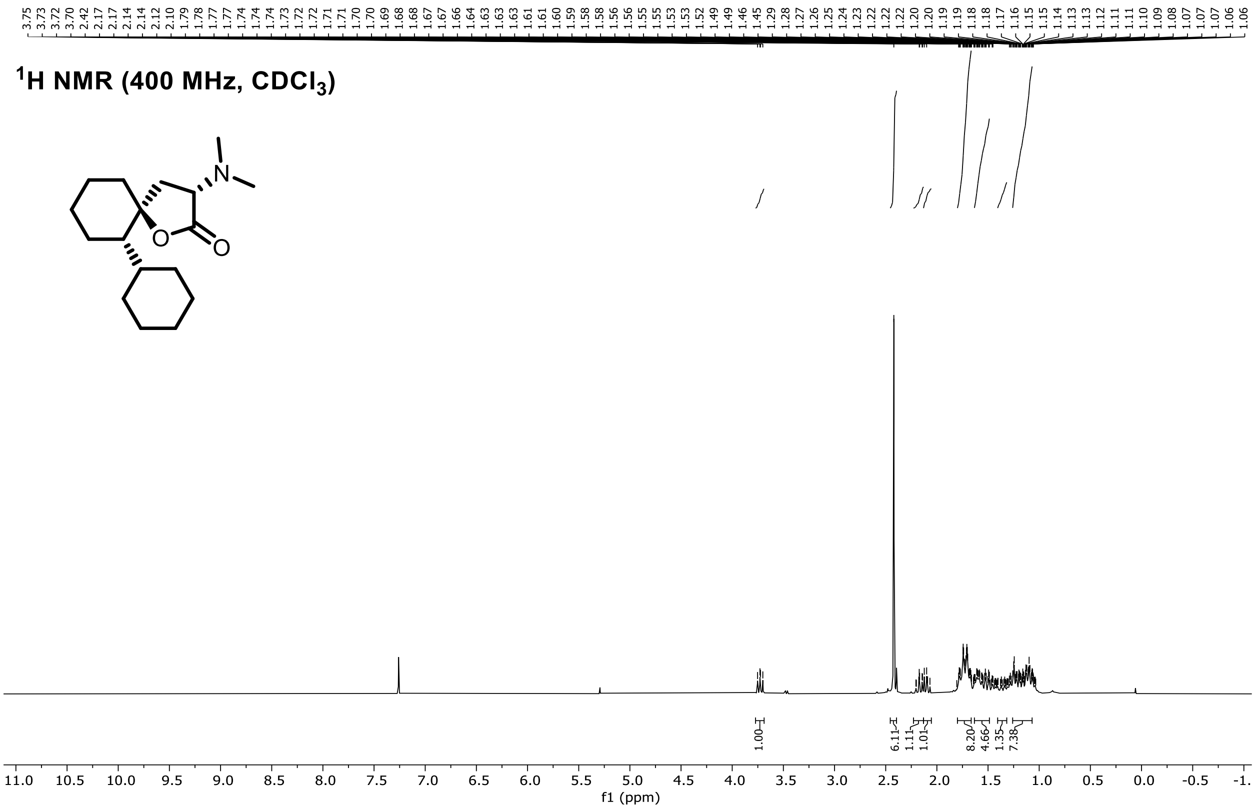

^{13}C NMR (101 MHz, $CDCl_3$)

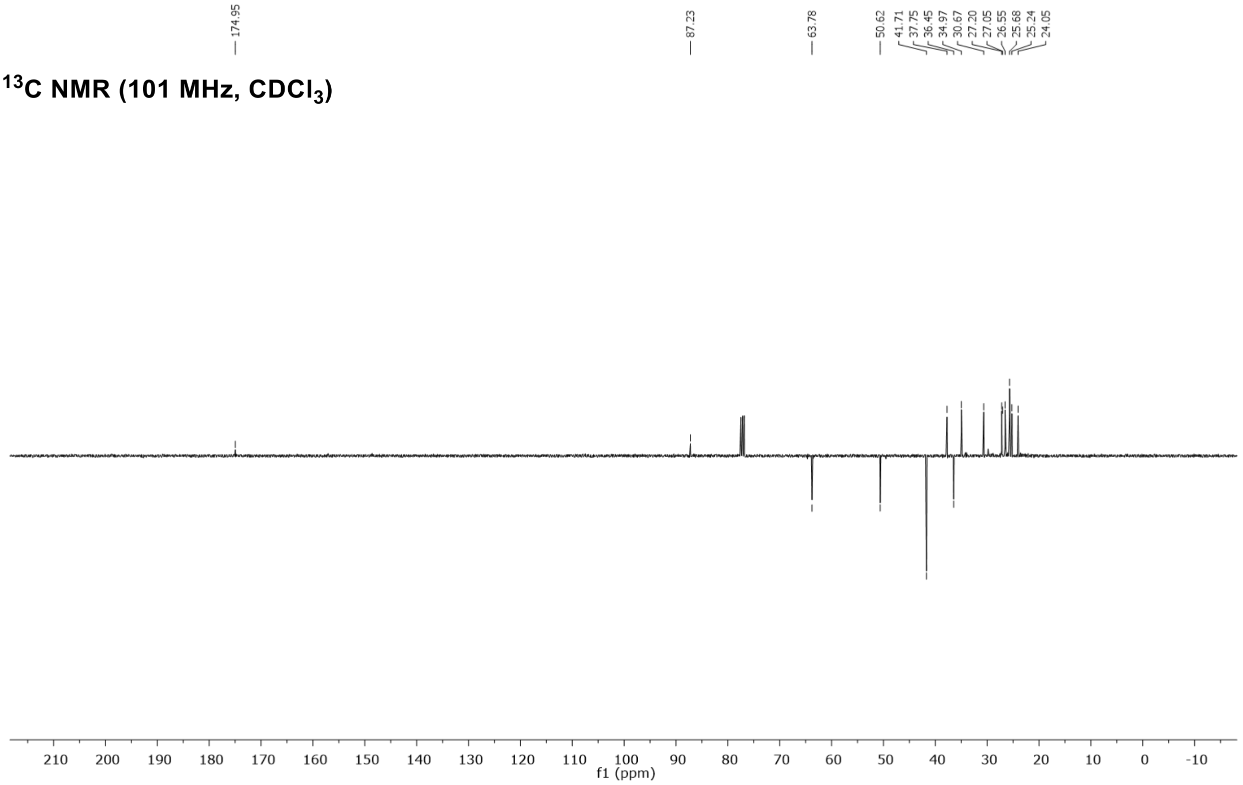

^{1}H NMR (400 MHz, $CDCl_3$)

minor diastereomer

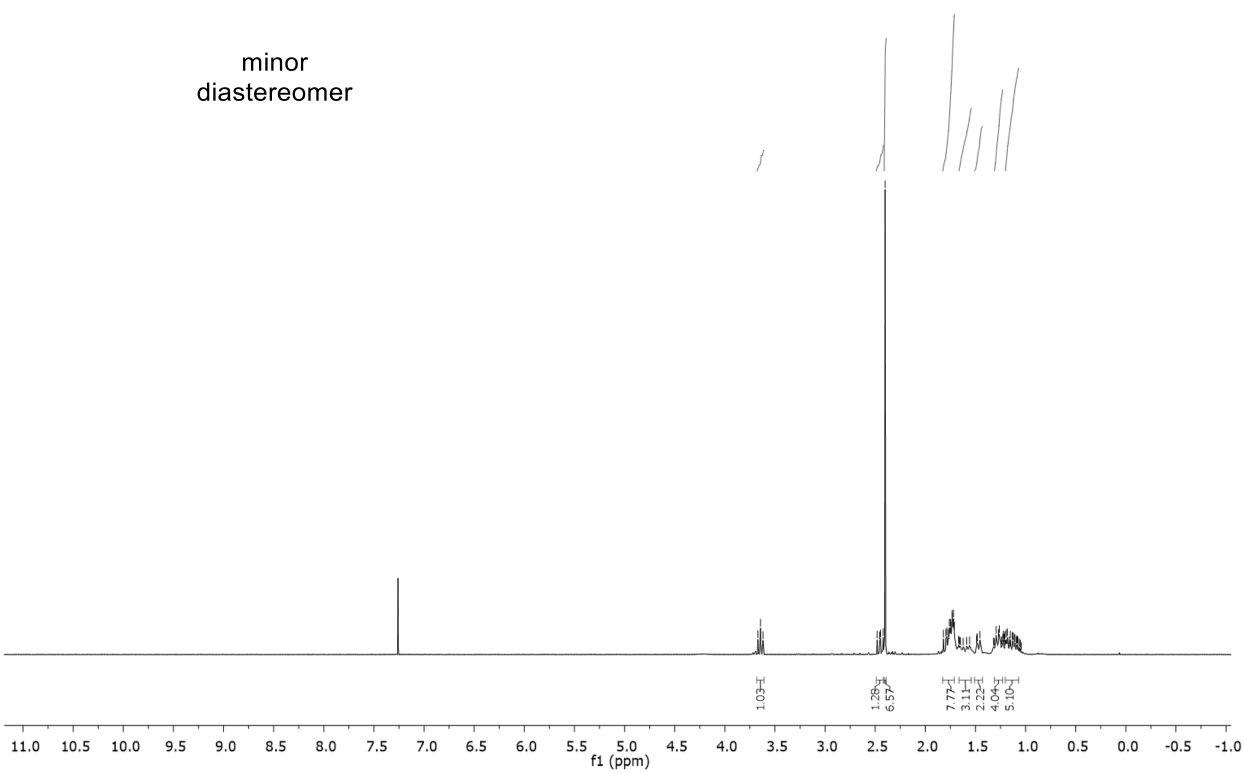

^{13}C NMR (101 MHz, $CDCl_3$)

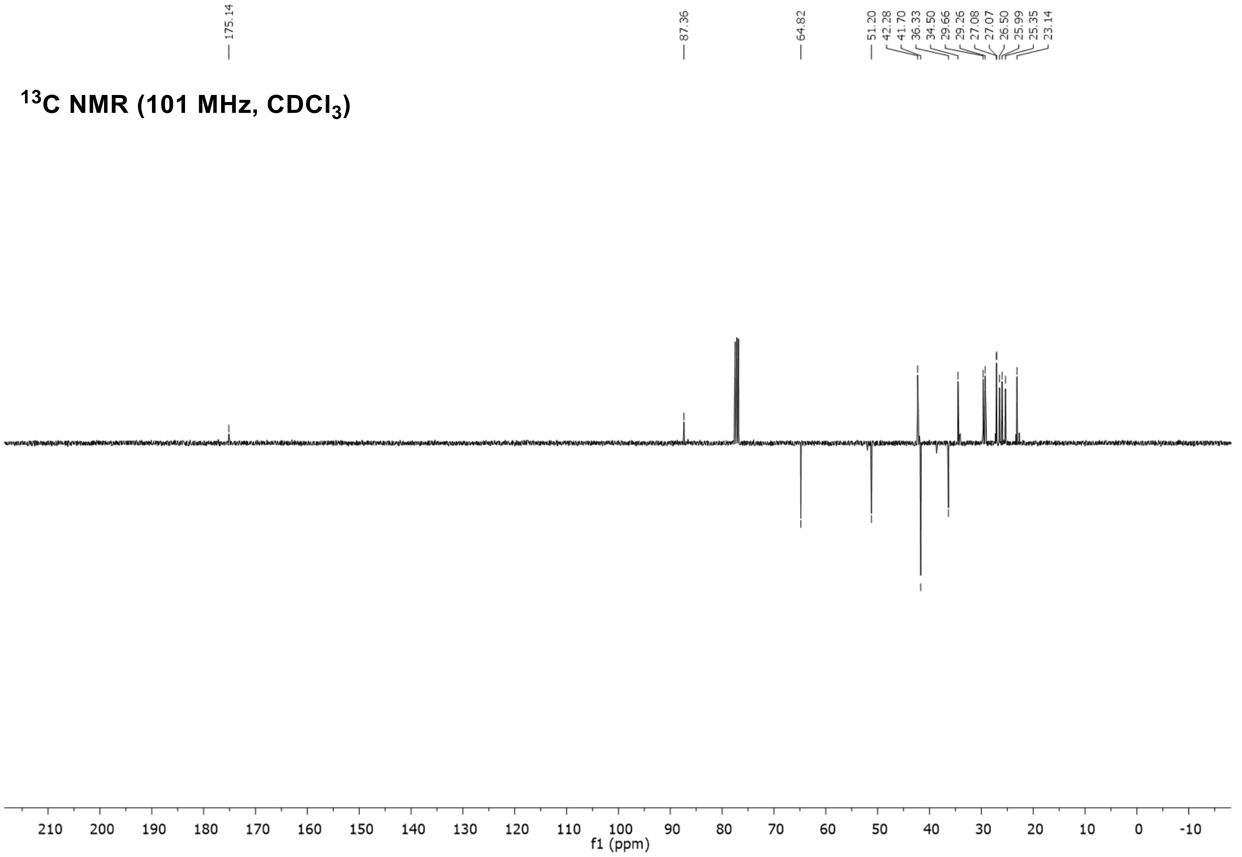

3-(Dimethylamino)-1,9,13-trioxadispiro[4.2.5⁸.2⁵]pentadecan-2-one (2n)

^{1}H NMR (600 MHz, $CDCl_3$)

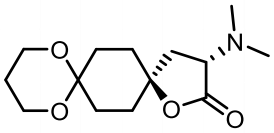

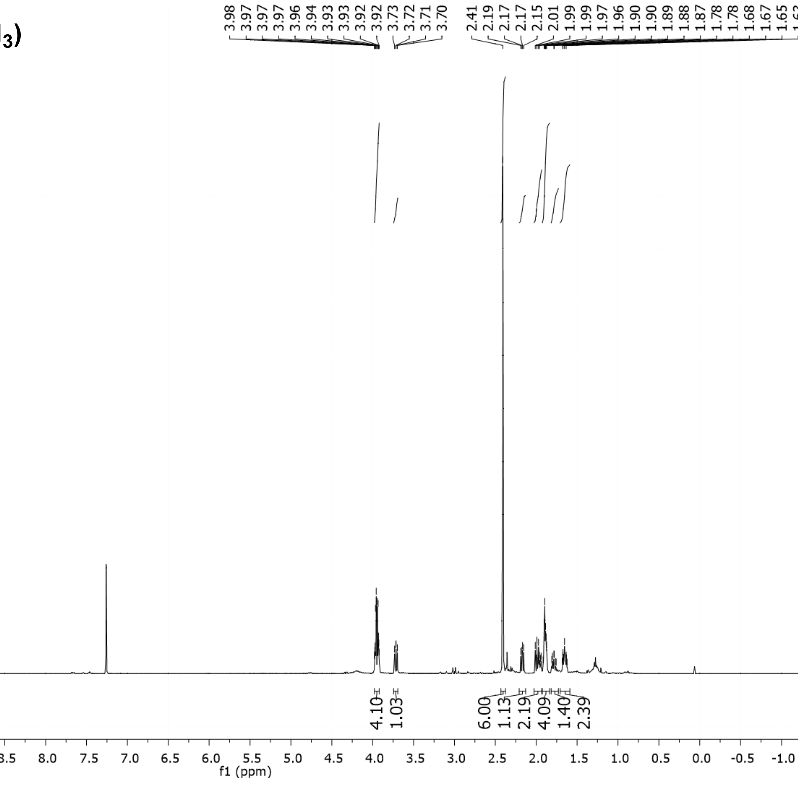

^{13}C NMR (151 MHz, $CDCl_3$)

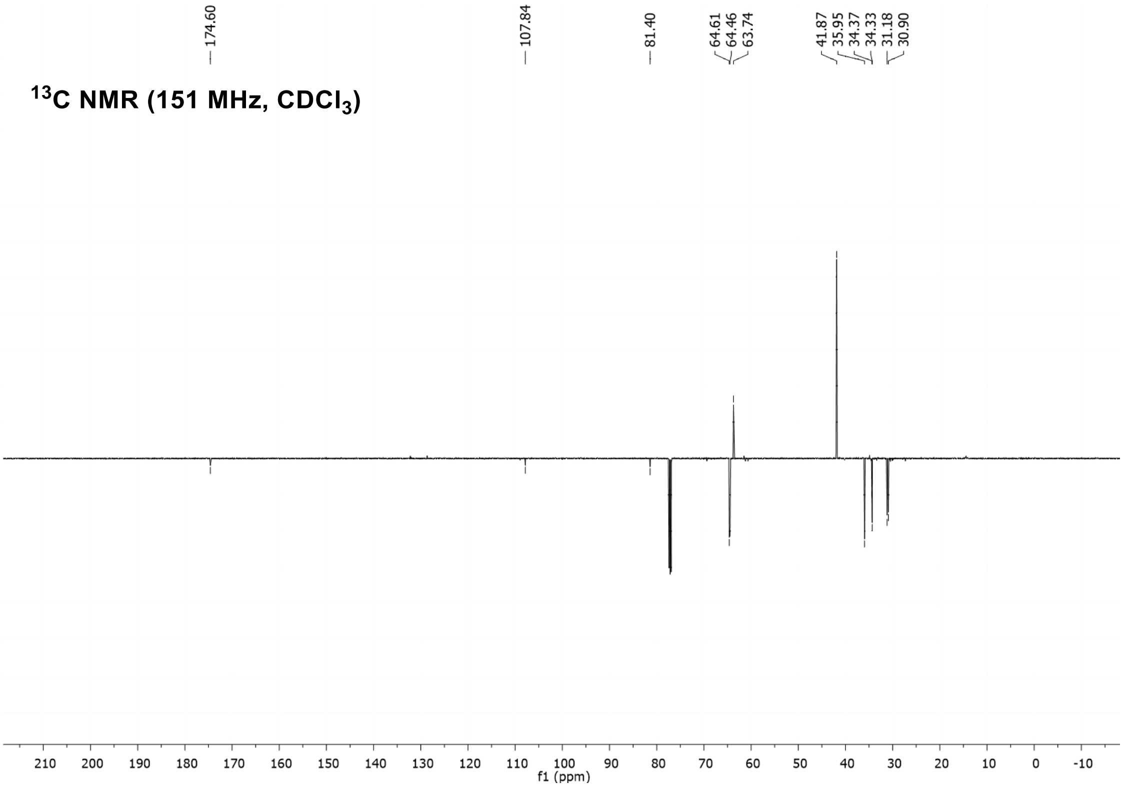

(3*S,5*s**,8*R**)-3-(Dimethylamino)-8-hydroxy-1-oxaspiro[4.5]decan-2-one (2o)**

^{1}H NMR (600 MHz, $CDCl_3$)

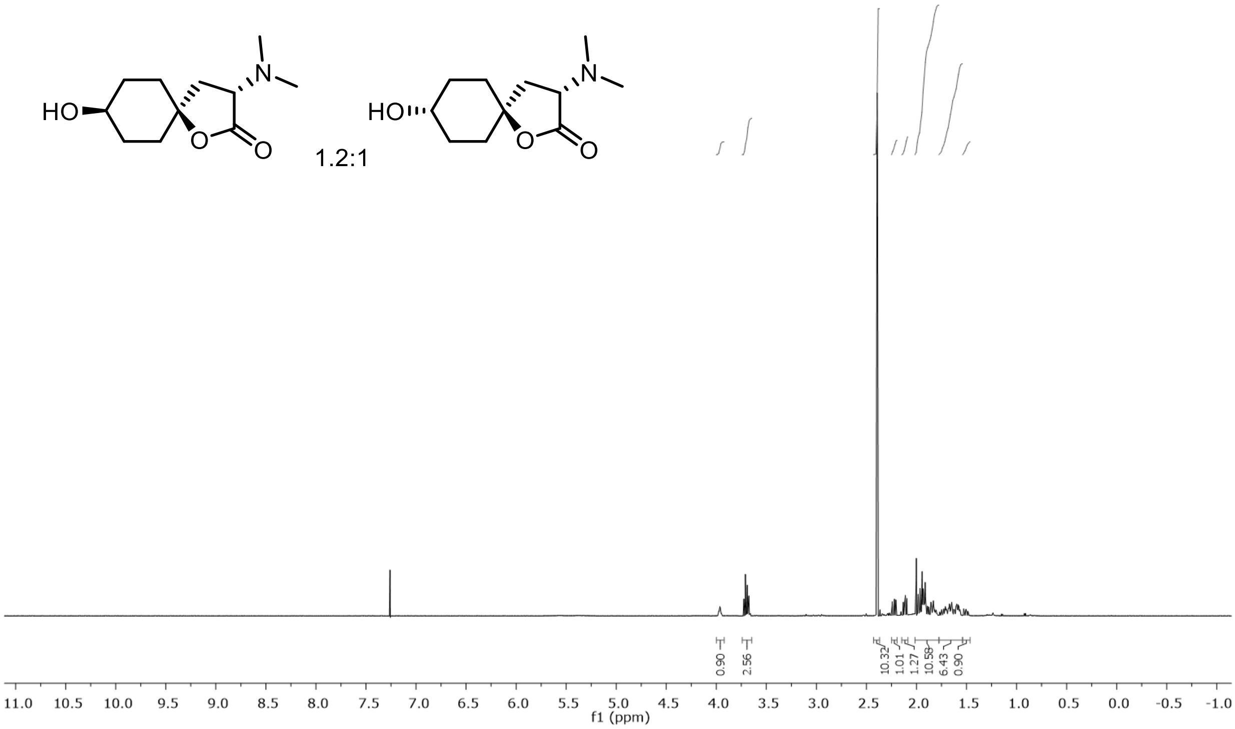

^{13}C NMR (151 MHz, $CDCl_3$)

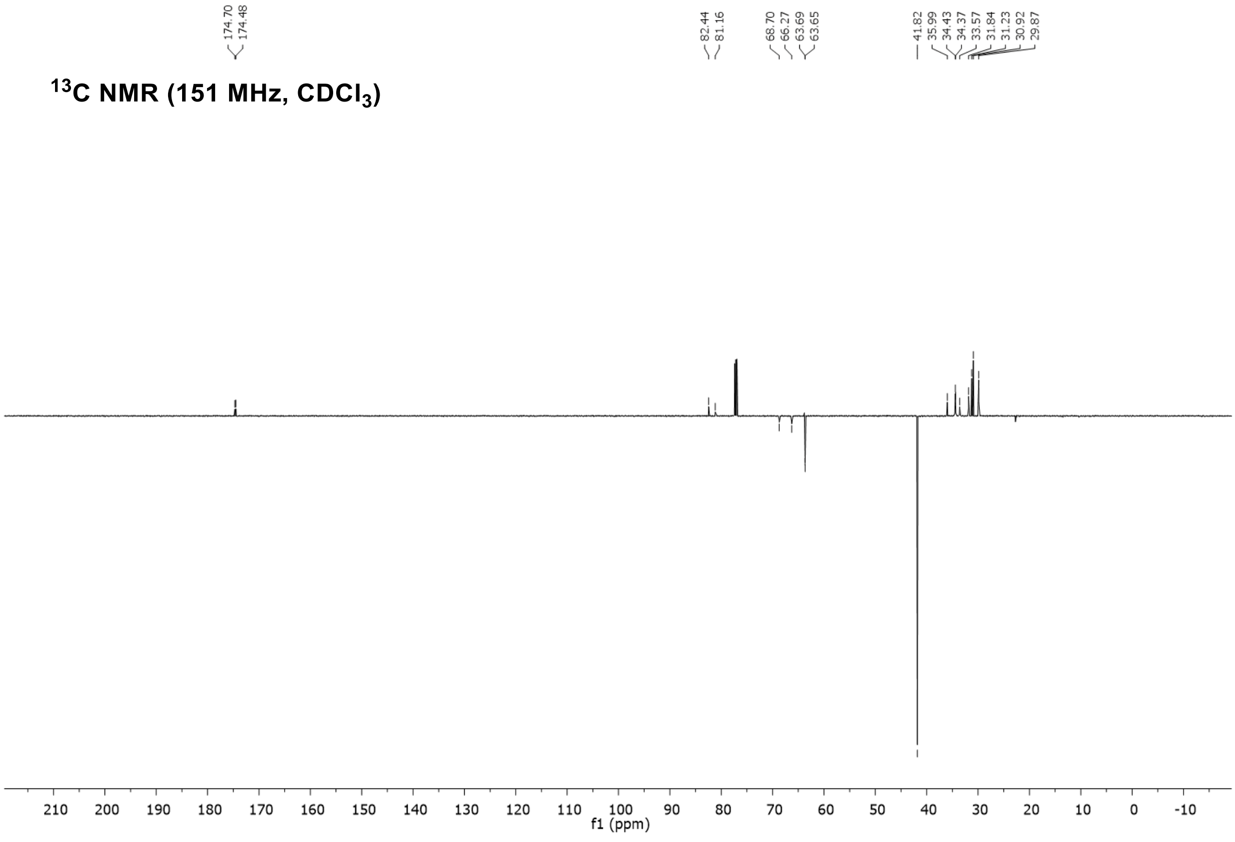

(3*S,5S*,6*R**)-3-(Dimethylamino)-6-ethoxy-1-oxaspiro[4.5]decan-2-one**

(major diastereomer 2p)

^{1}H NMR (400 MHz, $CDCl_3$)

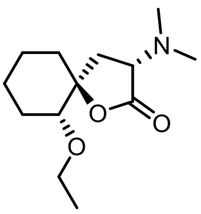

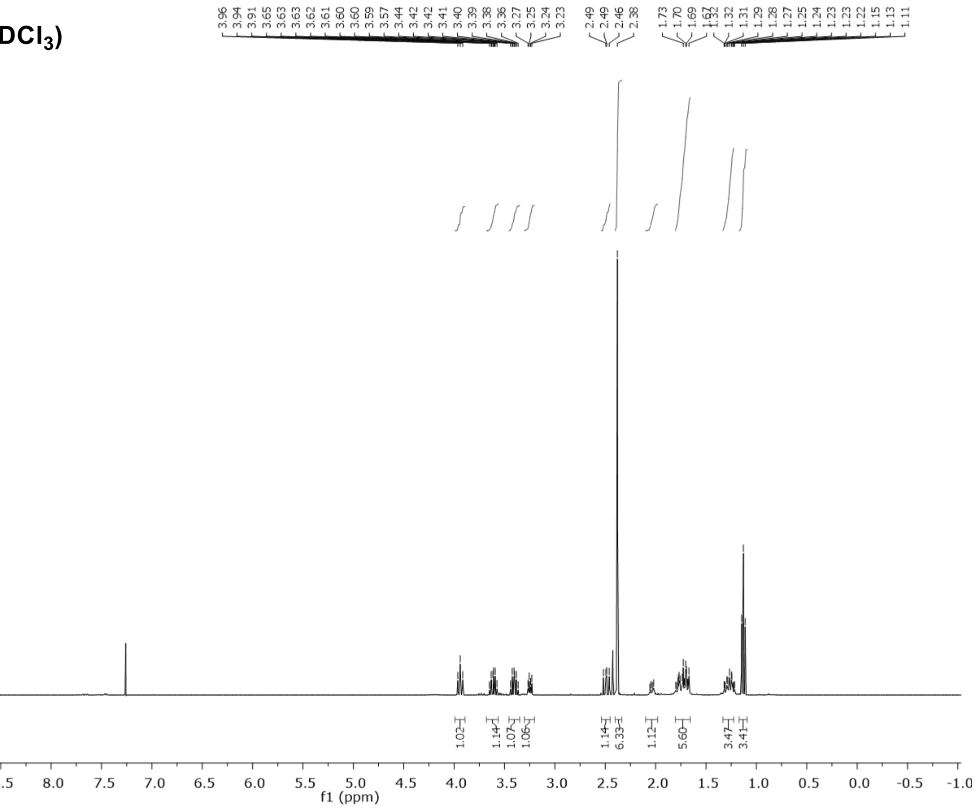

^{13}C NMR (101 MHz, $CDCl_3$)

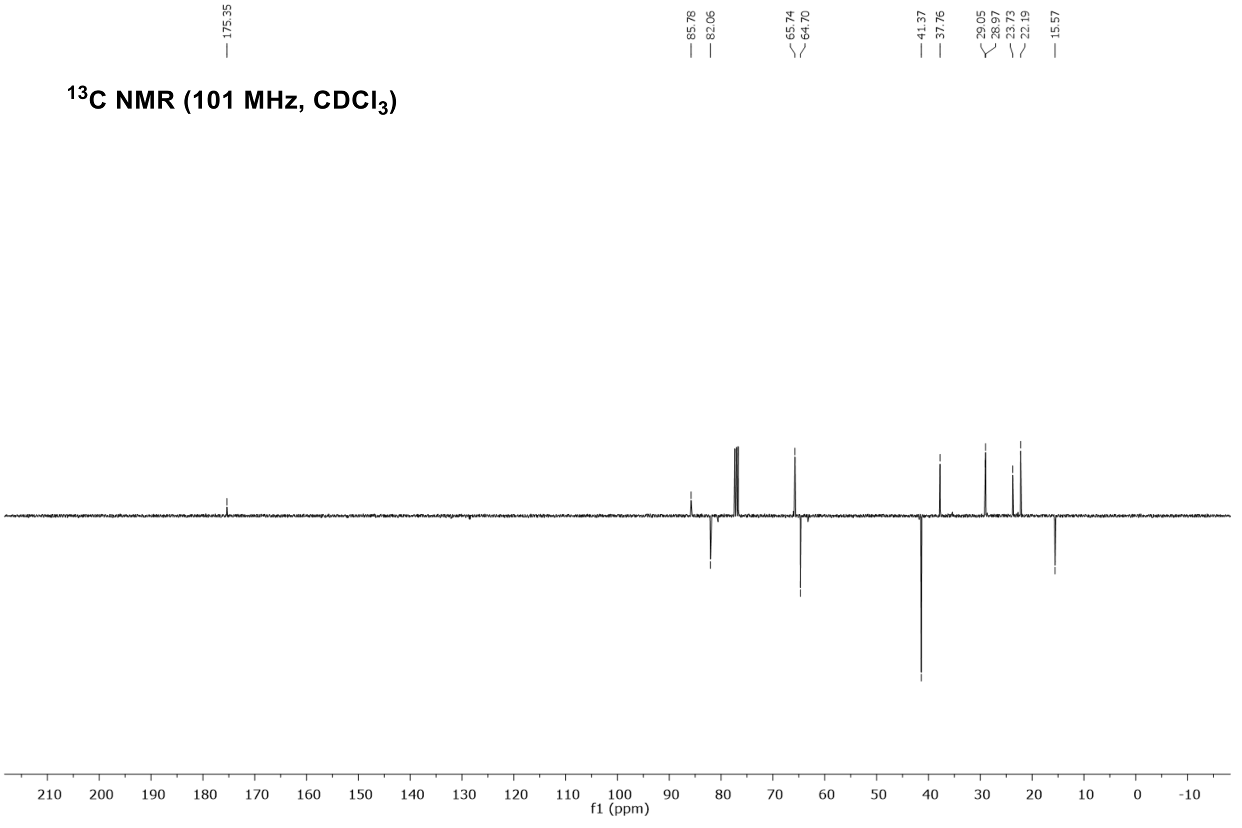

Ethyl (3*S,5*s**,8*R**)-3-(dimethylamino)-2-oxo-1-oxaspiro[4.5]decane-8-carboxylate (major diastereomer 2q)**

^{1}H NMR (400 MHz, $CDCl_3$)

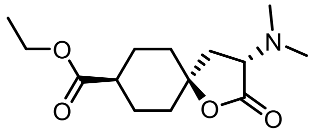

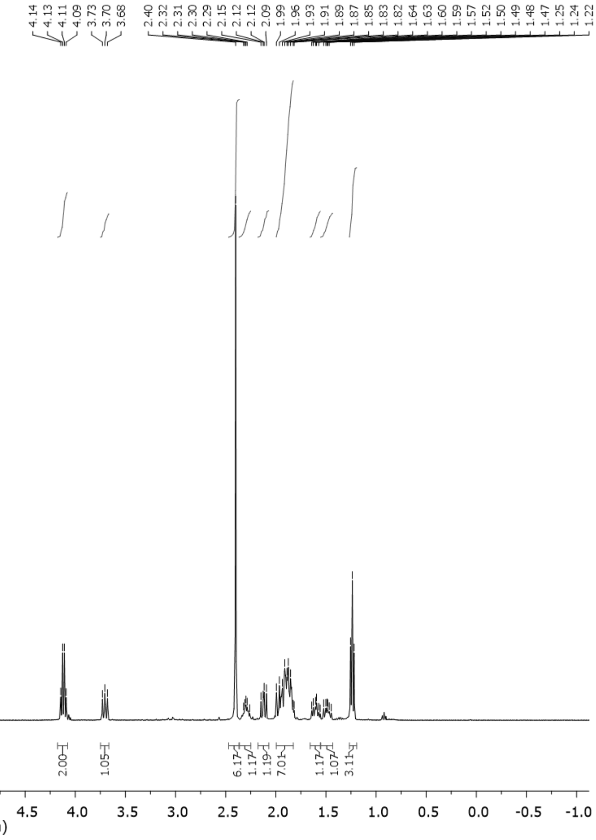

^{13}C NMR (101 MHz, $CDCl_3$)

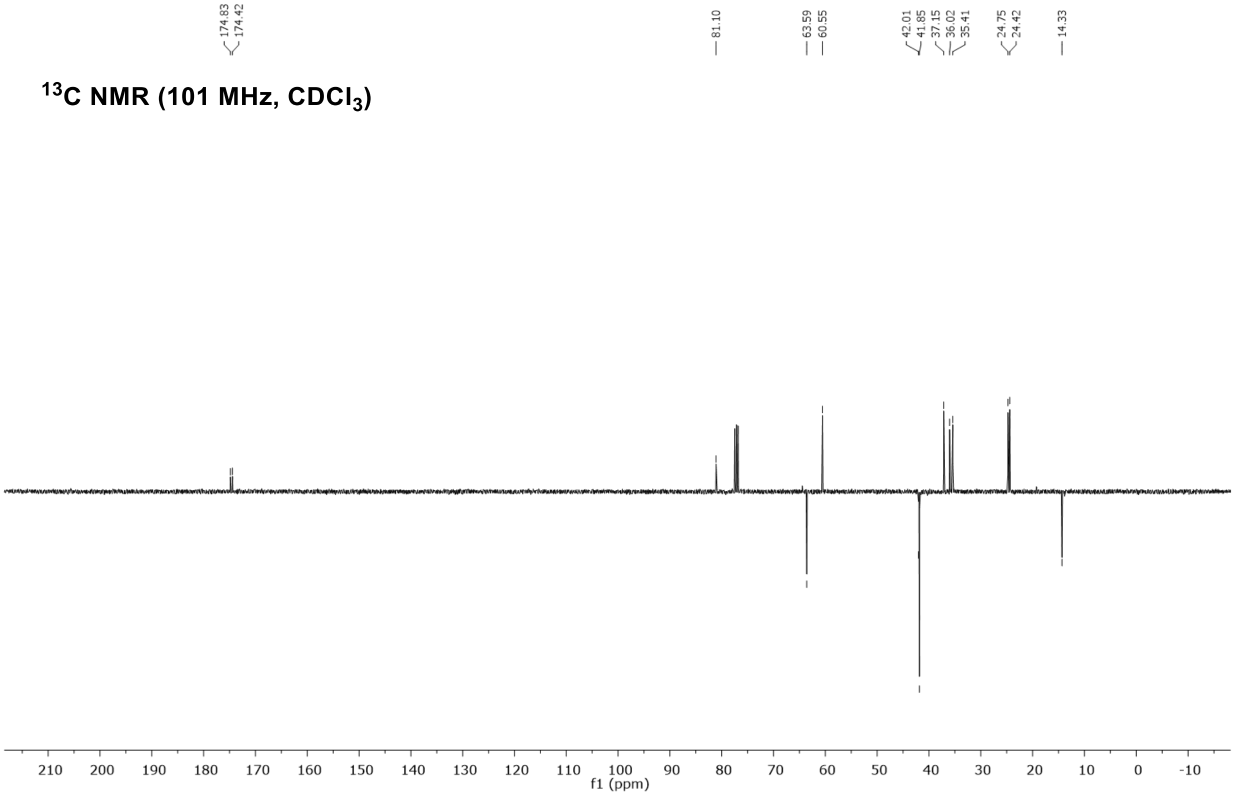

^{1}H NMR (400 MHz, $CDCl_3$)

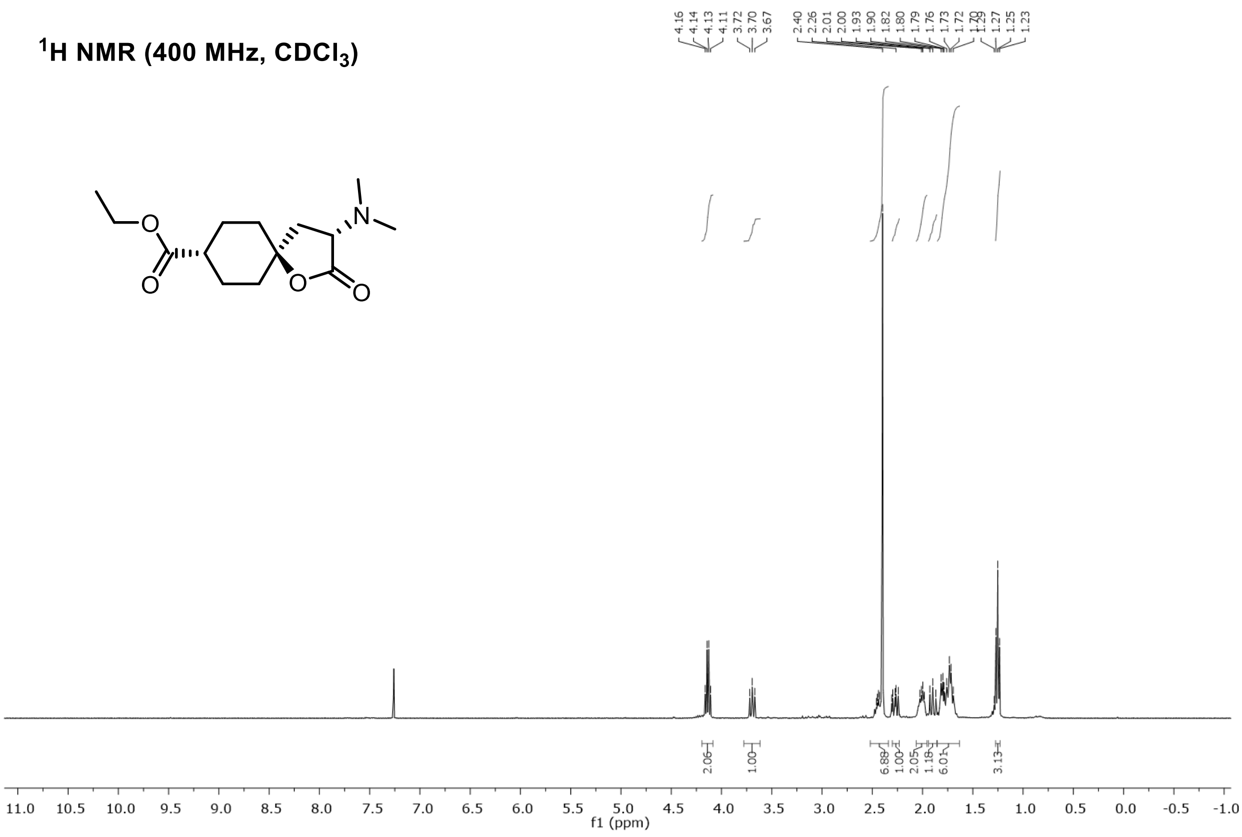

^{13}C NMR (101 MHz, $CDCl_3$)

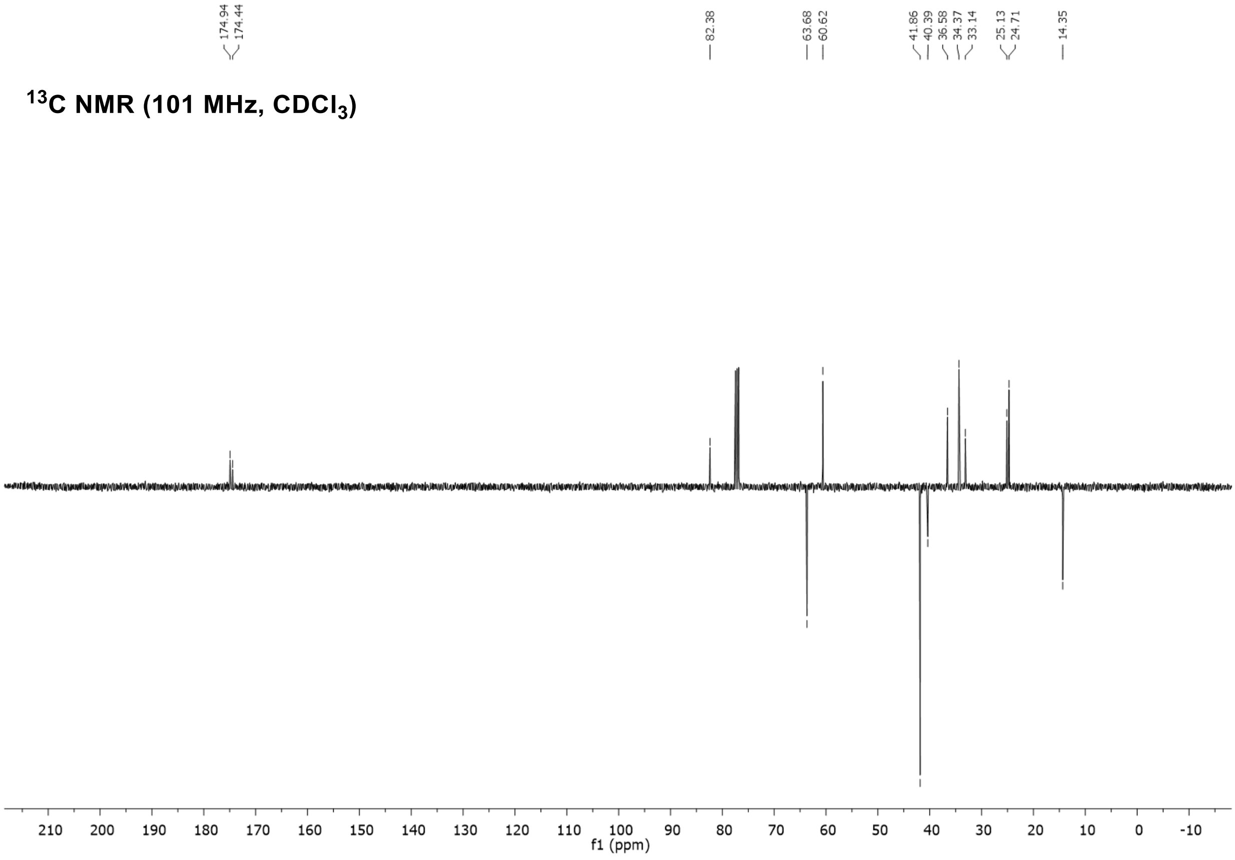

N-((3*S**,5*s**,8*R**)-3-(Dimethylamino)-2-oxo-1-oxaspiro[4.5]decan-8-yl)benzamide (2r)

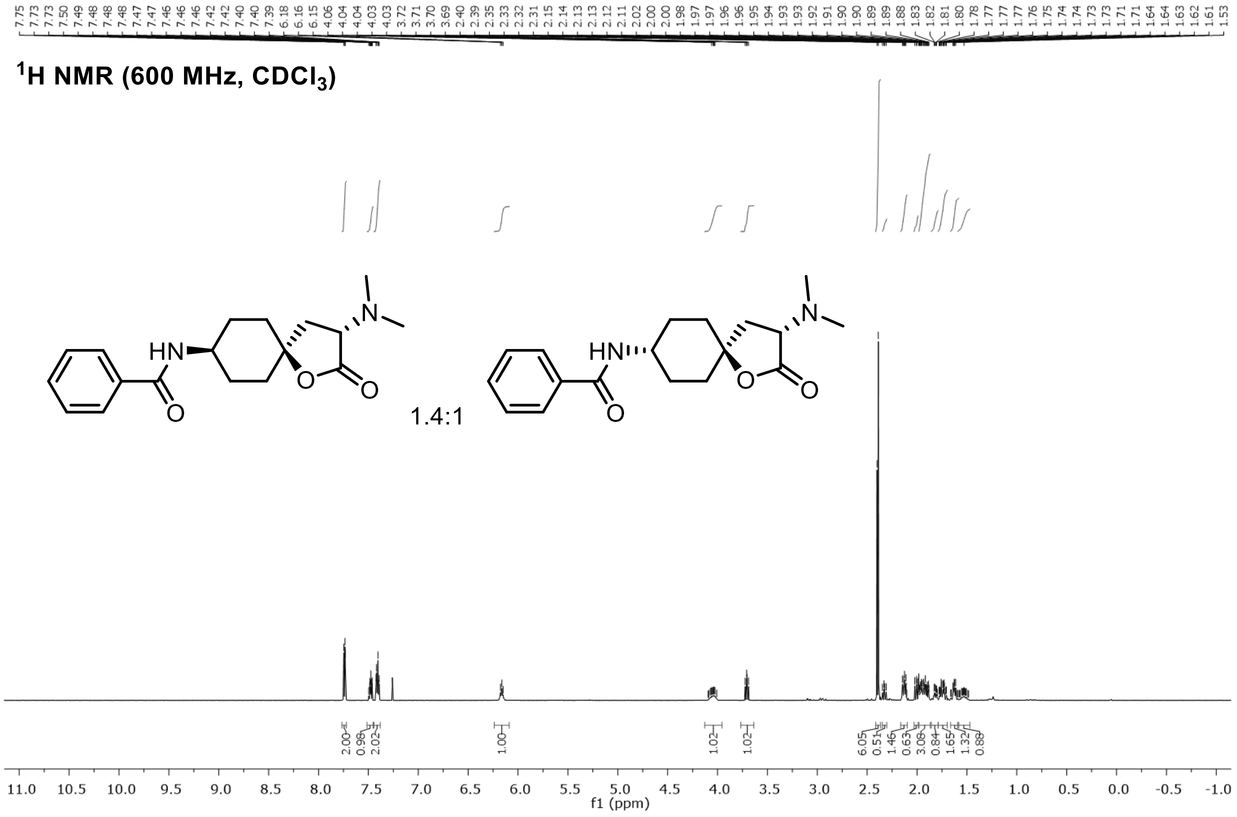

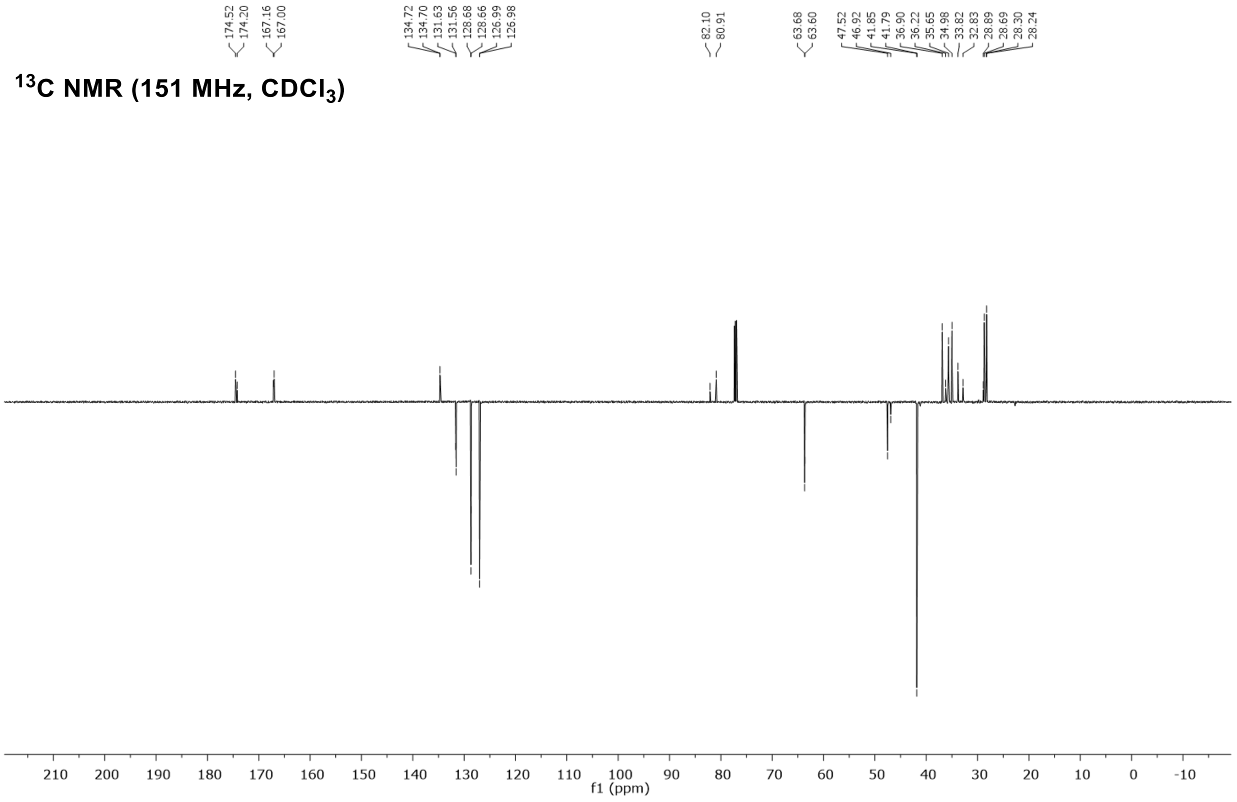

3-((3*S,5*S**,6*S**)-3-(Dimethylamino)-2-oxo-1-oxaspiro[4.5]decan-6-yl)propanenitrile (2s)**

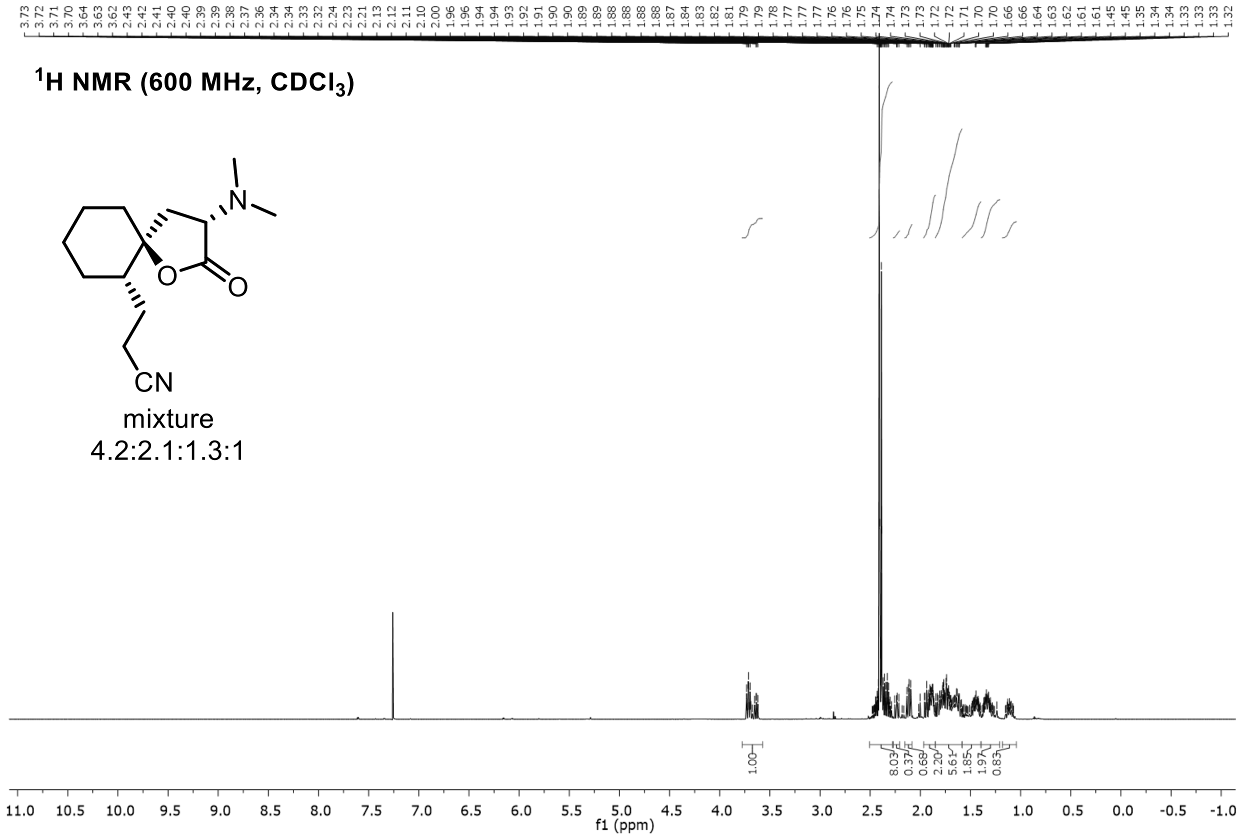

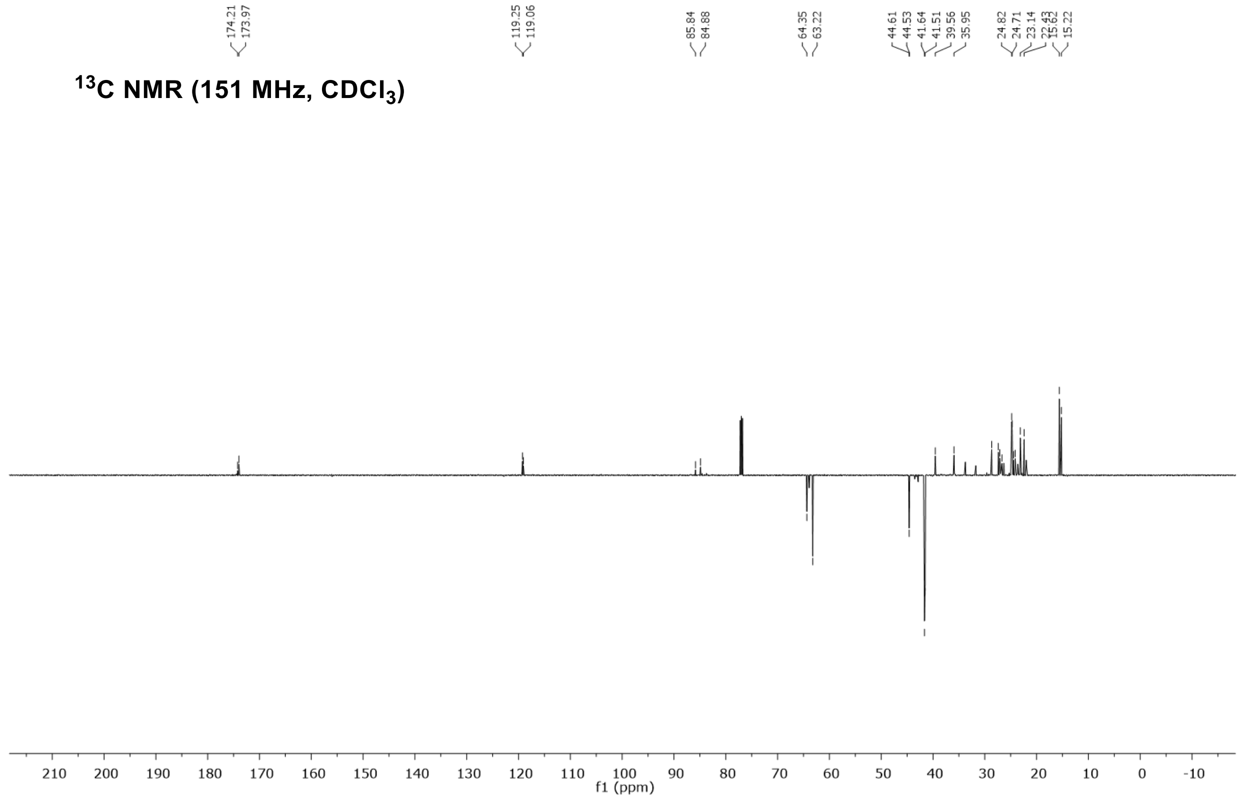

(2*S**,4*S**,4a'*S**,8a'*R**)-4-(Dimethylamino)decahydro-2'*H*,5*H*-spiro[furan-2,1'-naphthalen]-5-one (2t)

^{1}H NMR (400 MHz, $CDCl_3$)

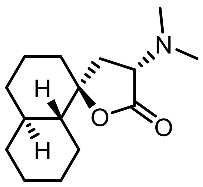

major diastereomer

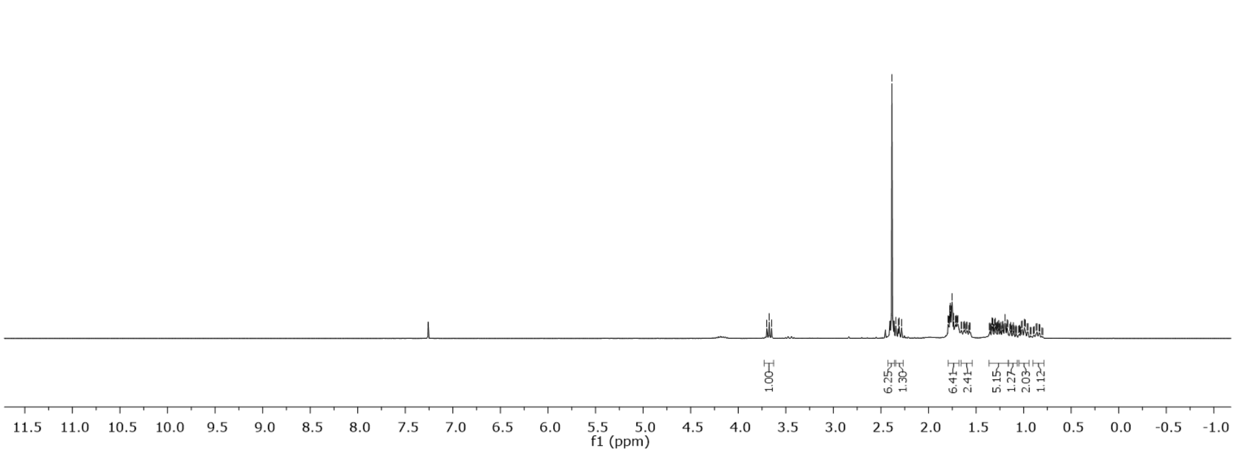

^{13}C NMR (101 MHz, $CDCl_3$)

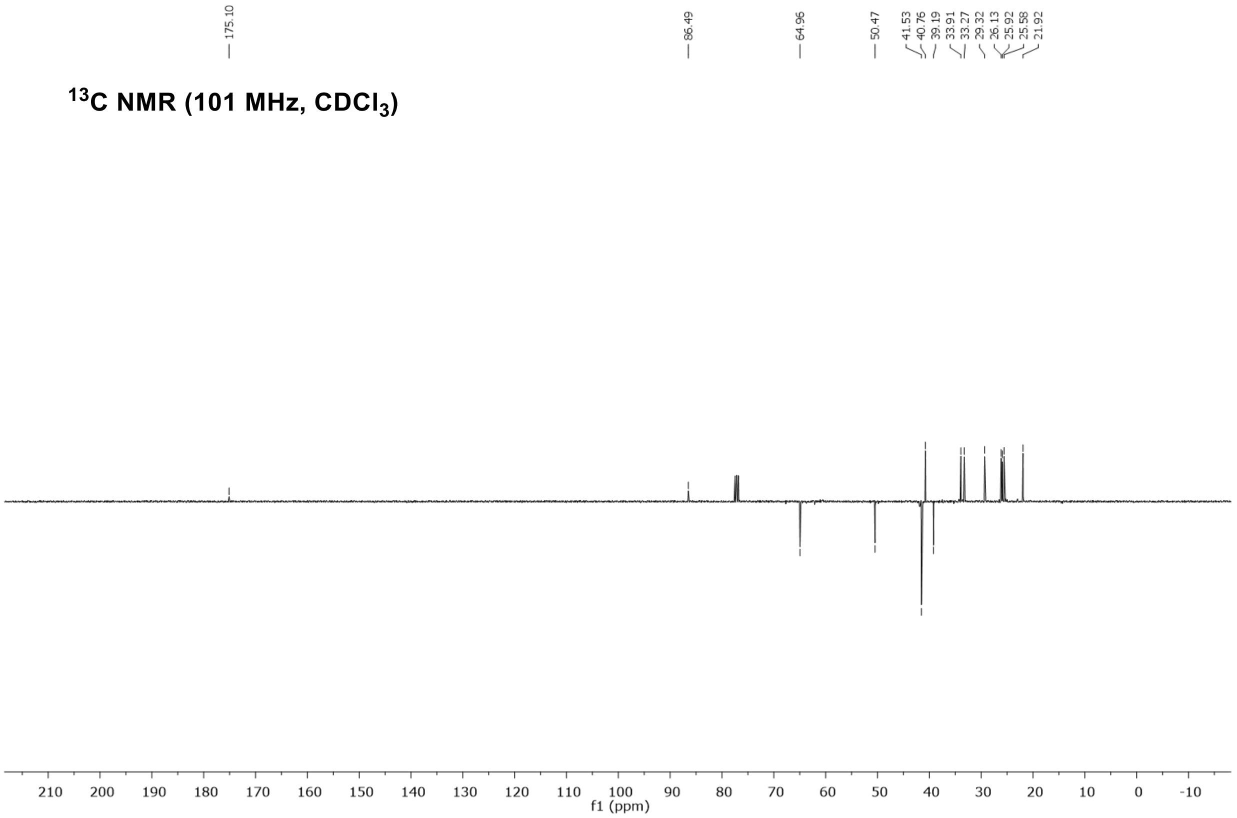

(2*S,4*S**,4a'*R**,8a'*R**)-4-(Dimethylamino)decahydro-2'*H*,5*H*-spiro[furan-2,1'-naphthalen]-5-one (2u)**

^{1}H NMR (400 MHz, CDCl$_3$)

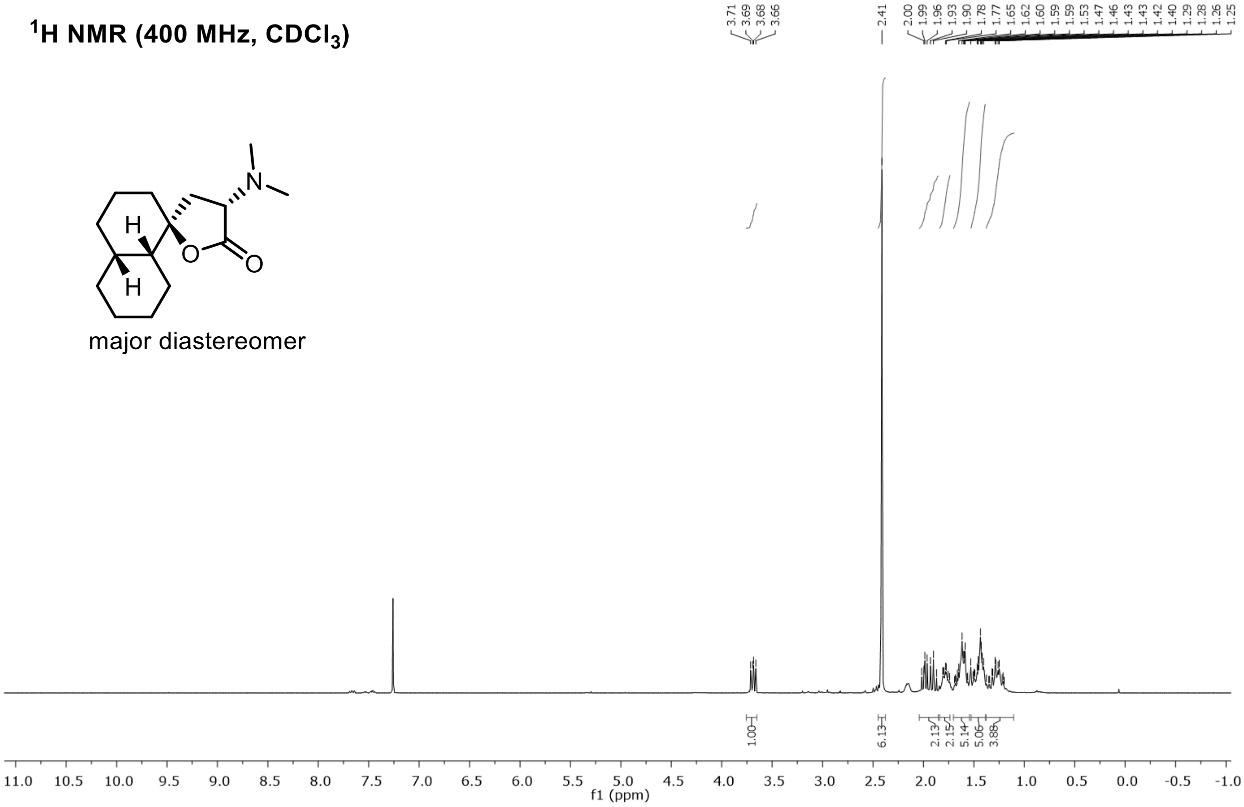

major diastereomer

^{13}C NMR (101 MHz, CDCl$_3$)

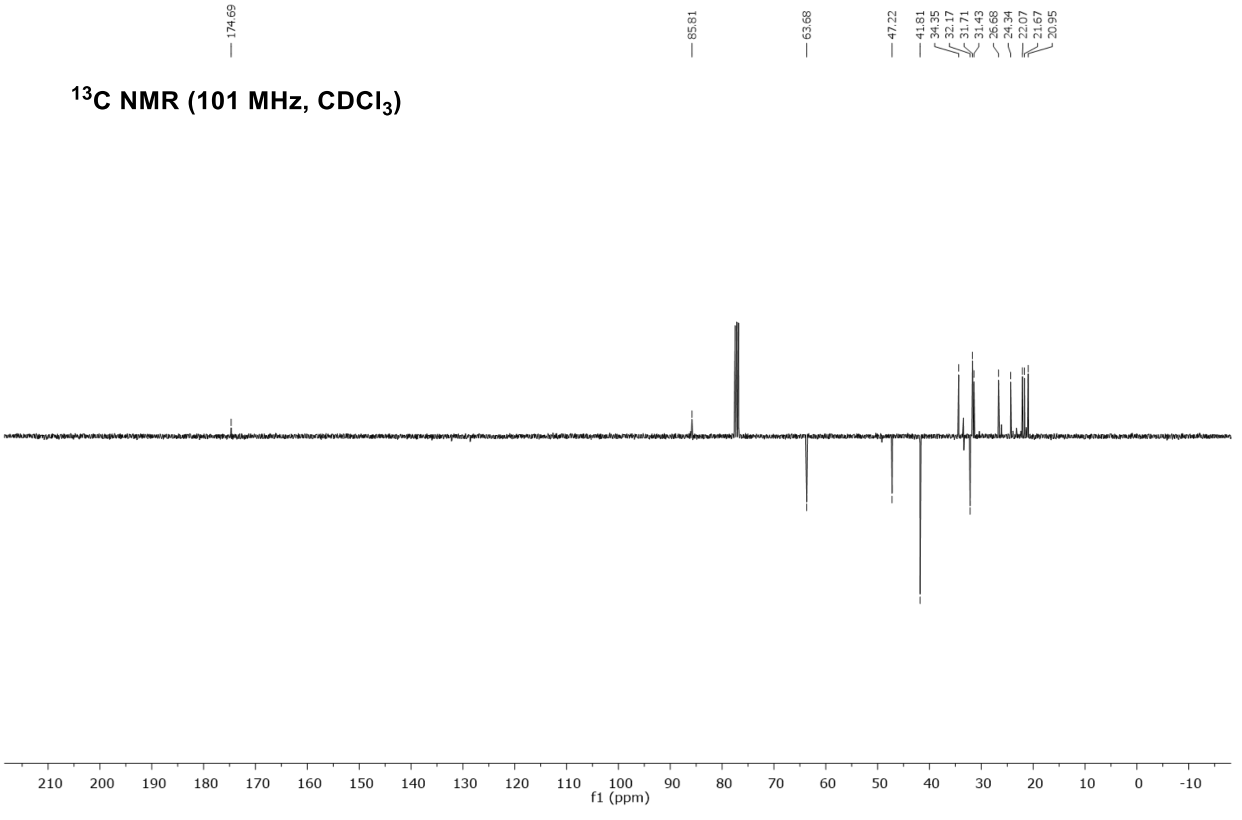

(3*S*,5S,6*S*,9*R*)-3-(Dimethylamino)-6-isopropyl-9-methyl-1-oxaspiro[4.5]decan-2-one (2v)

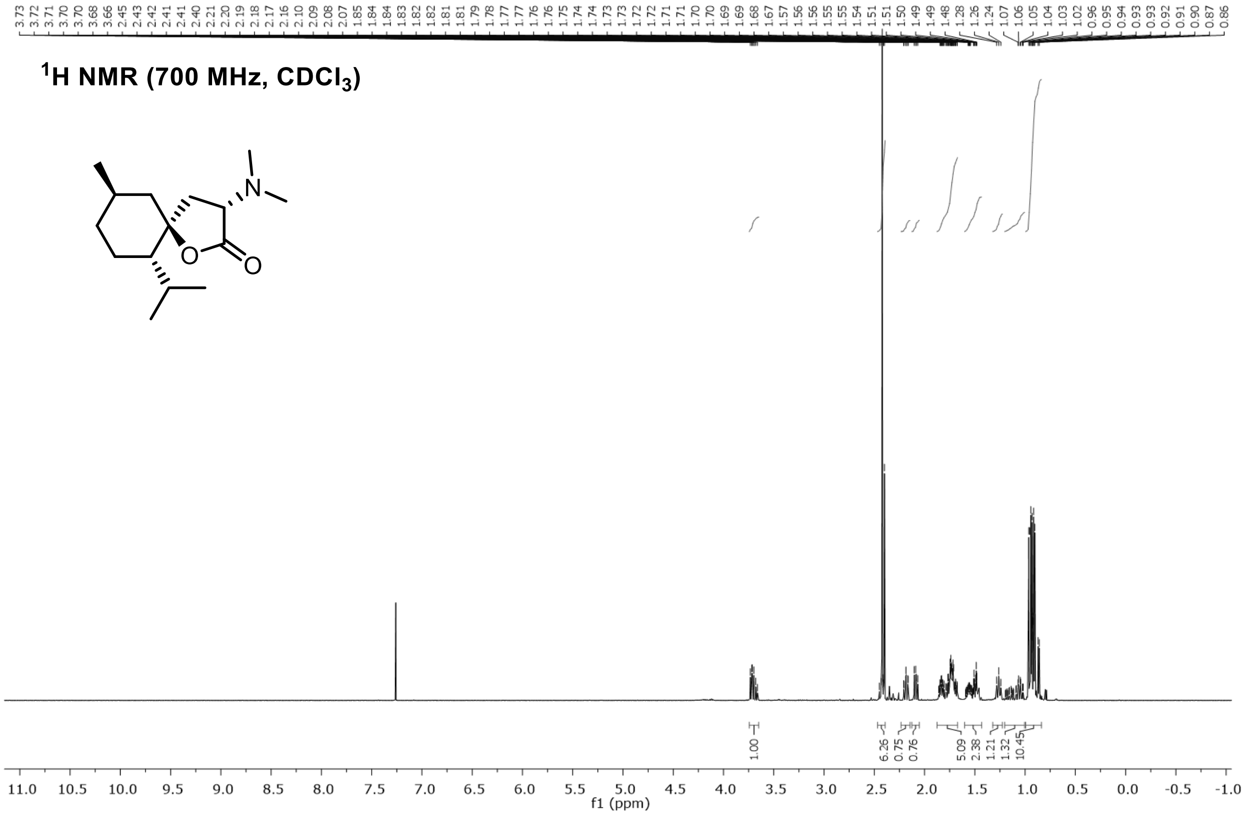

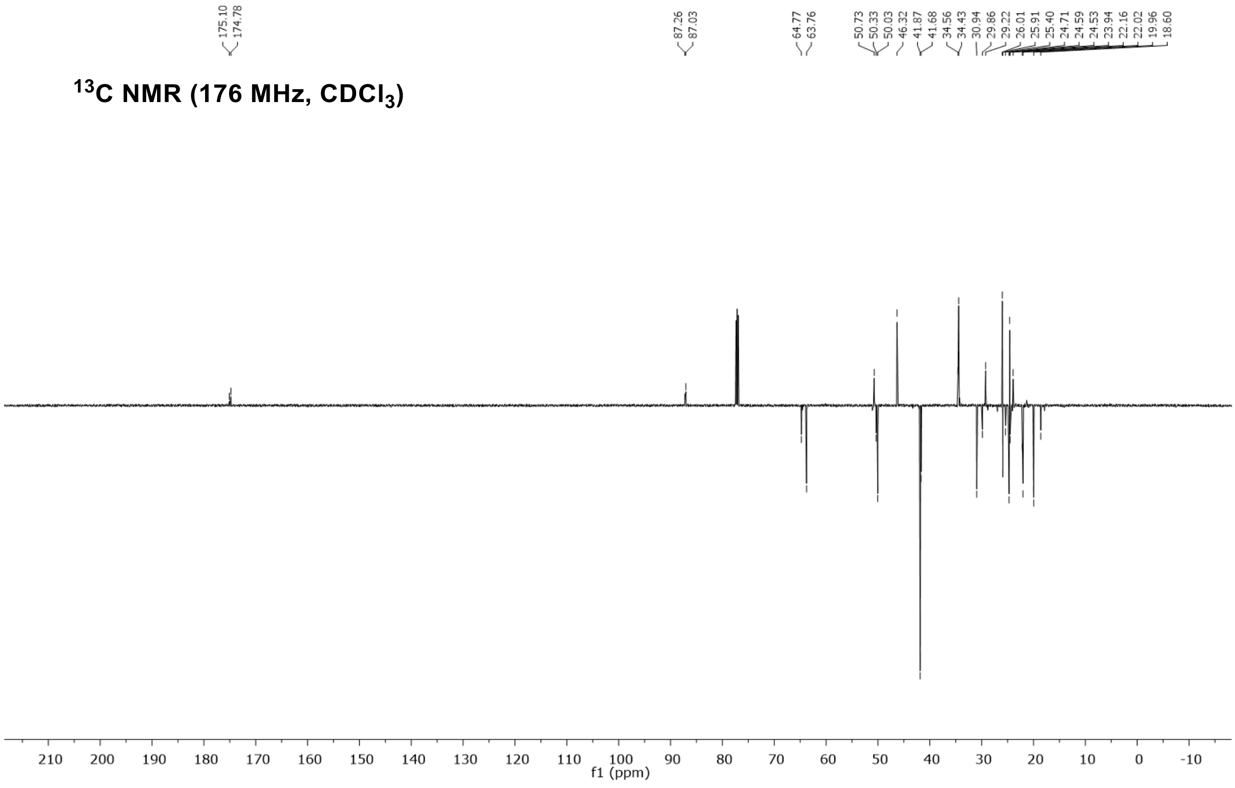

(3*S**,4*S**)-3-(Dimethylamino)-4-methyl-1-oxaspiro[4.5]decan-2-one (2w)

^{1}H NMR (600 MHz, $CDCl_3$)

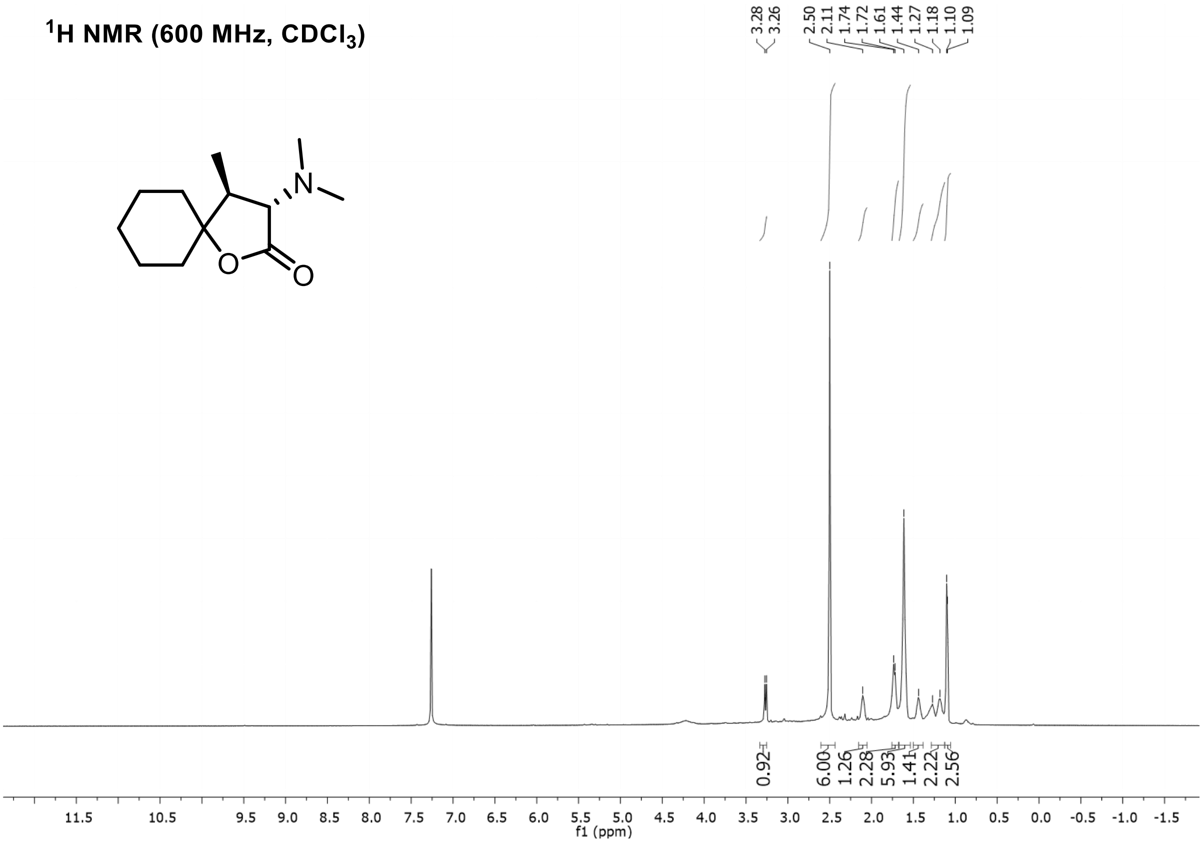

^{13}C NMR (151 MHz, $CDCl_3$)

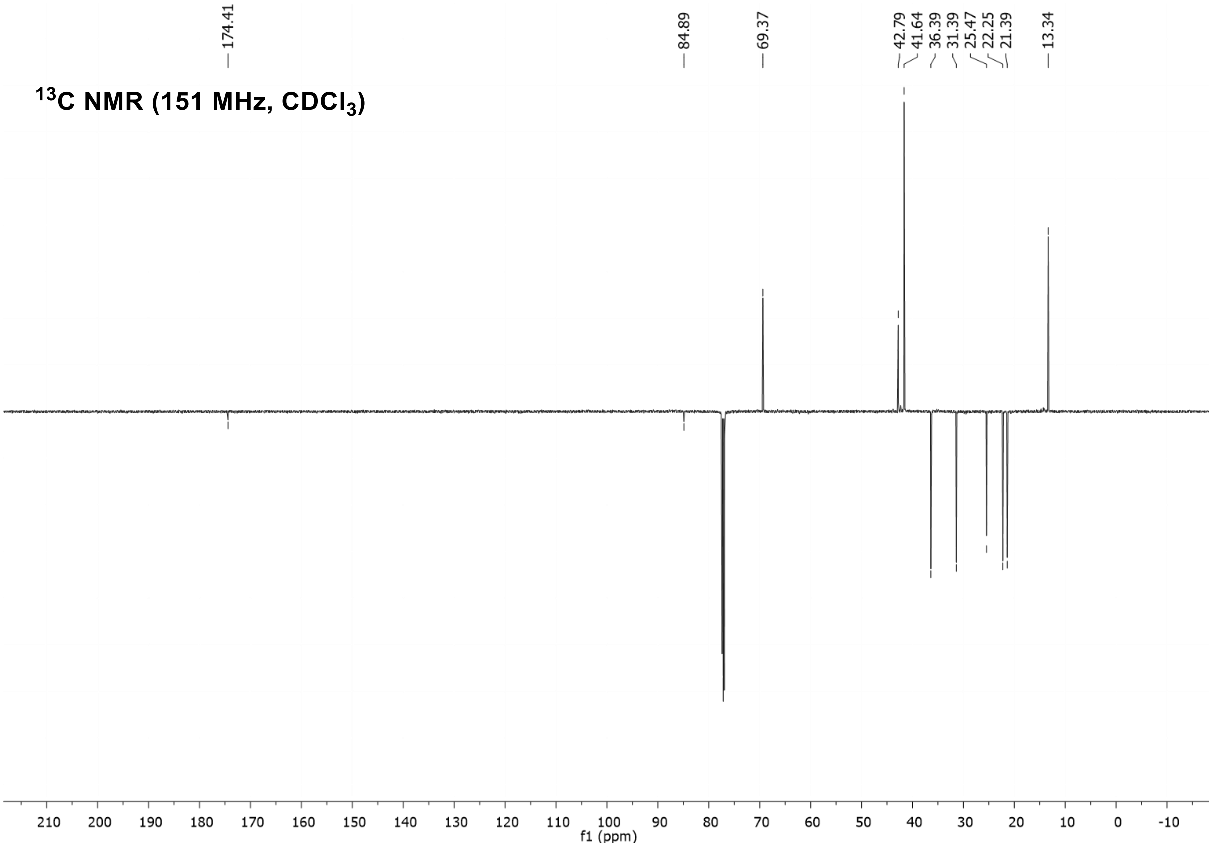

(3*S**,5*S**,6*S**)-6-Allyl-3-(dimethylamino)-1-oxaspiro[4.5]decan-2-one (2x)

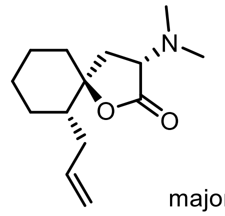

major

plus traces of one of the
minor diastereomers

^{1}H NMR (400 MHz, $CDCl_3$)

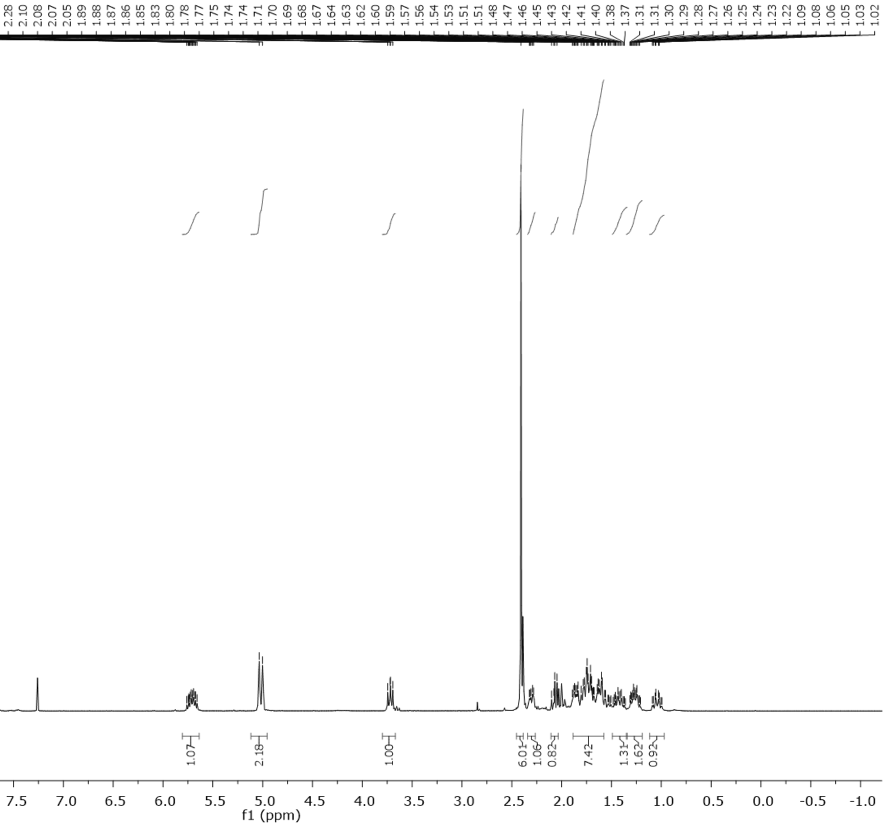

^{13}C NMR (101 MHz, $CDCl_3$)

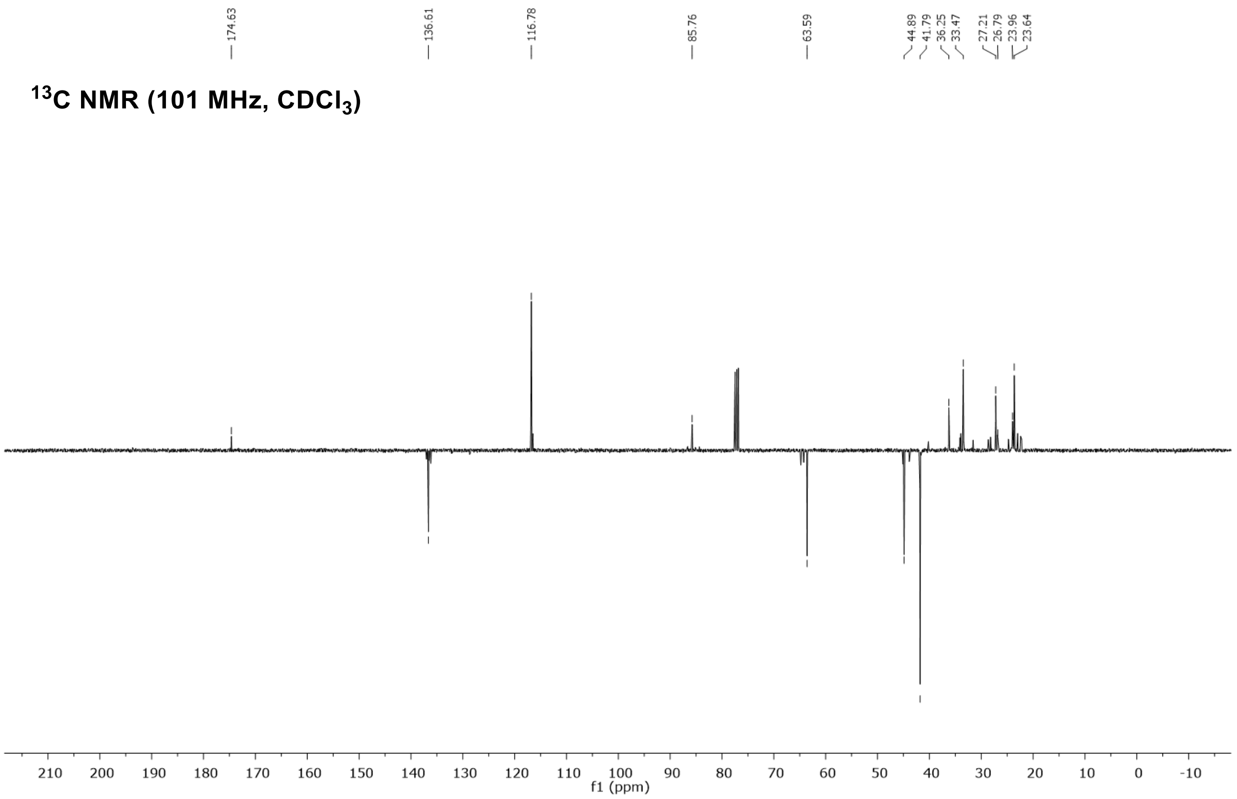

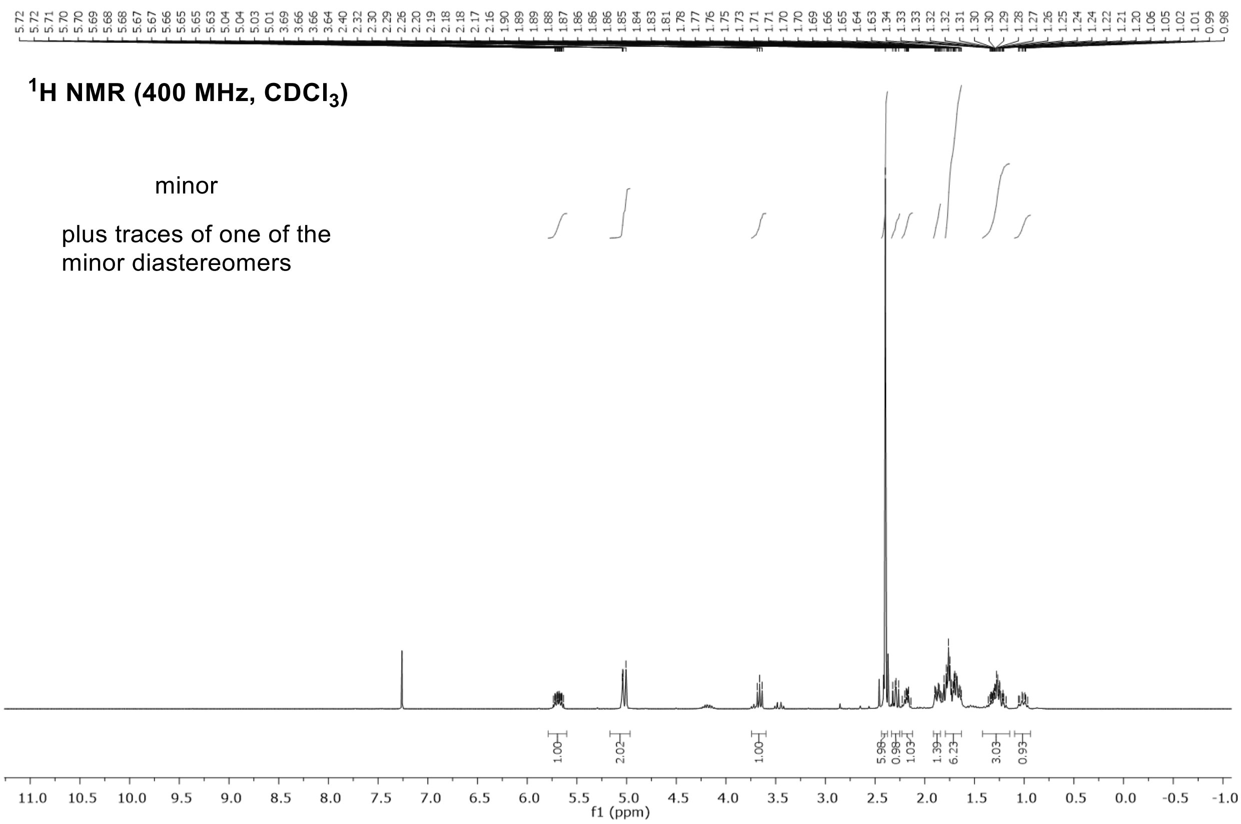

1H NMR (400 MHz, CDCl3)
minor
plus traces of one of the minor diastereomers
f1 (ppm)

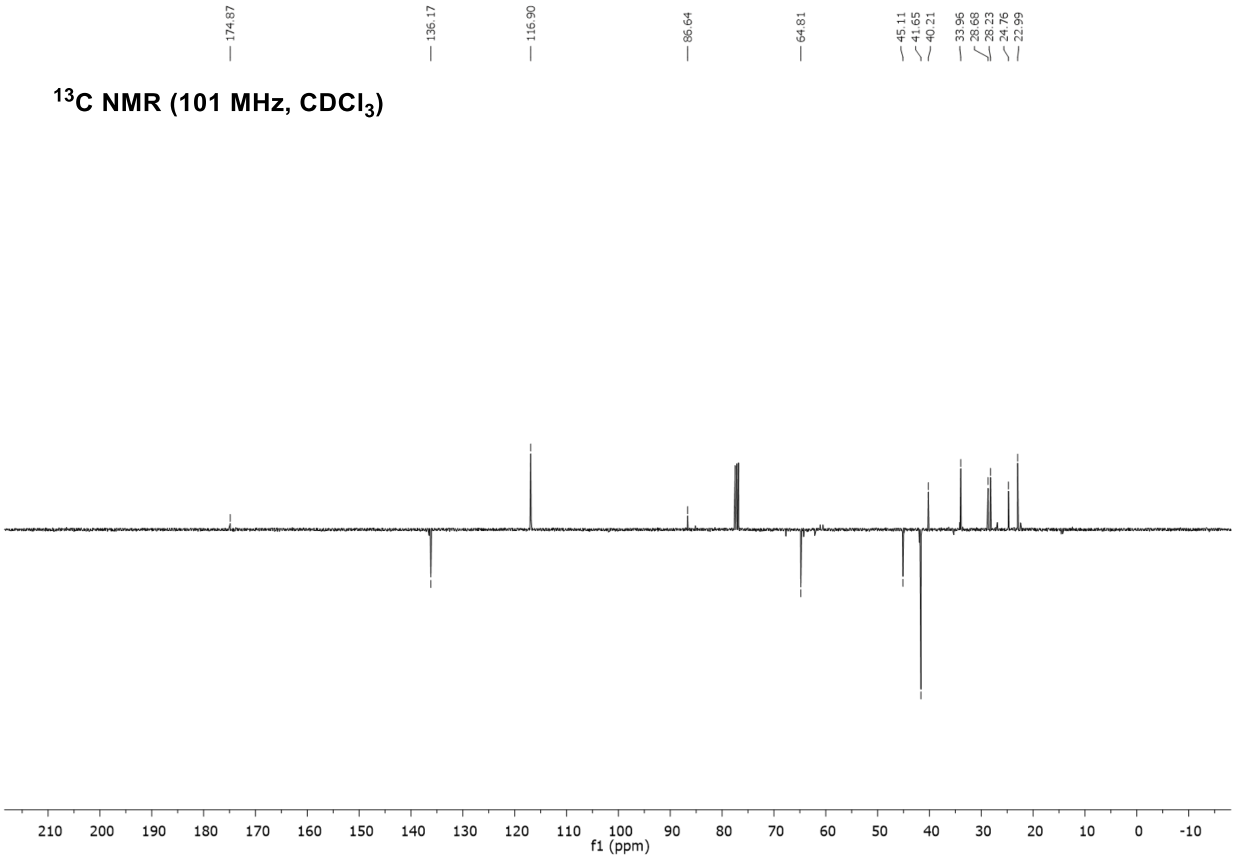

174.87
136.17
116.90
86.64
64.81
45.11
41.65
40.21
33.96
28.68
28.23
24.76
22.99
13C NMR (101 MHz, CDCl3)
f1 (ppm)

(3*S*,5*S*)-3-(Dimethylamino)-5-methyl-5-((*S*)-4-methylcyclohex-3-en-1-yl)dihydrofuran-2(3*H*)-one (diastereomer 1 – 2y)

^{1}H NMR (700 MHz, $CDCl_3$)

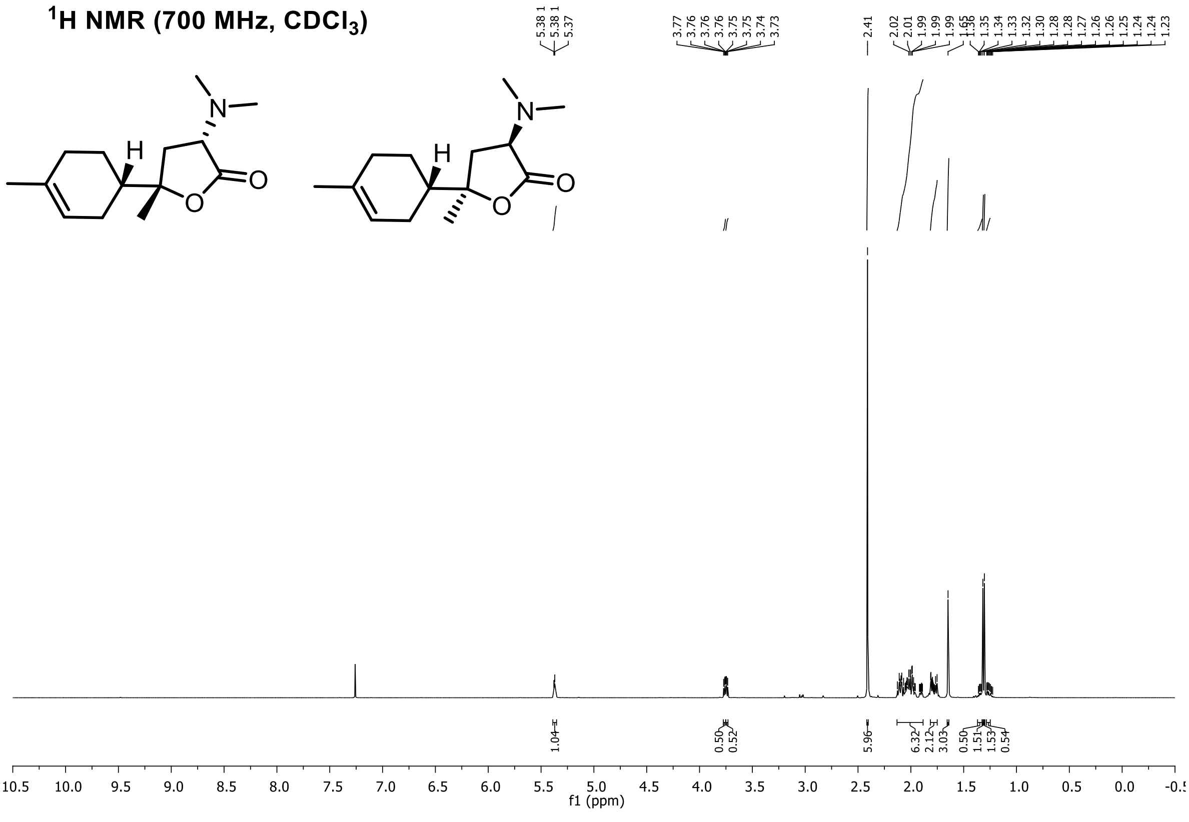

^{13}C NMR (176 MHz, $CDCl_3$)

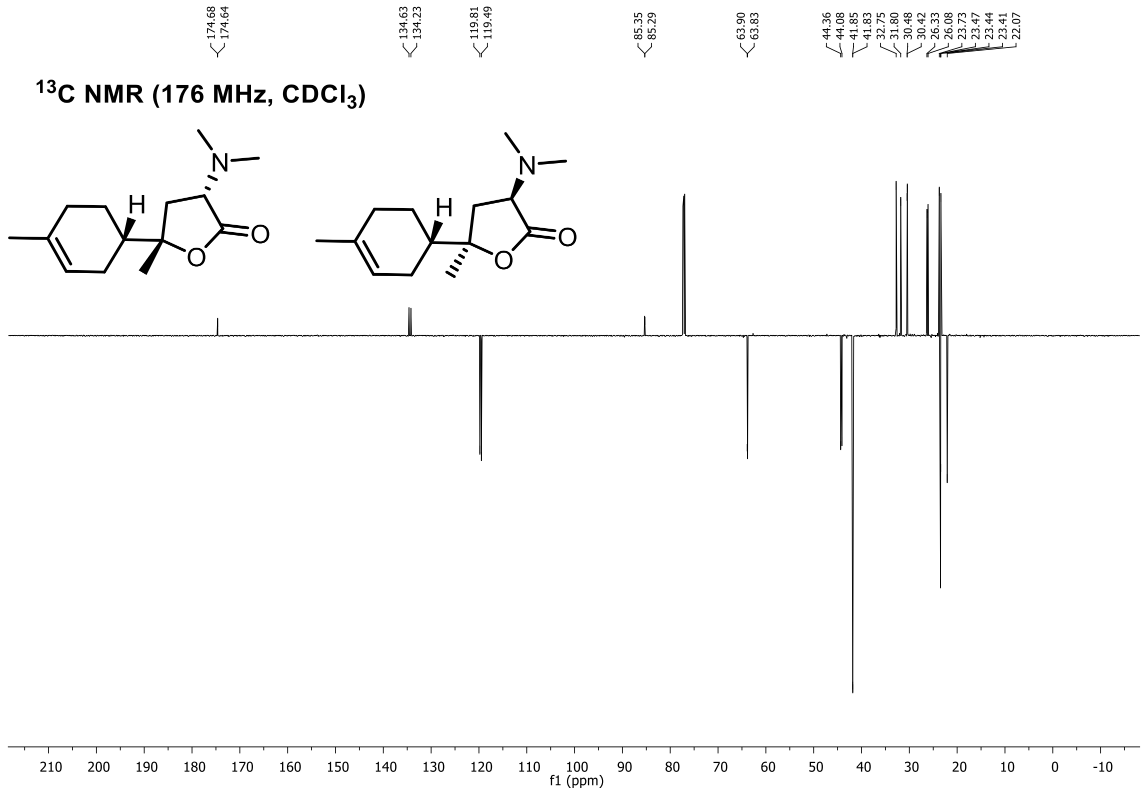

3-(Dibenzylamino)-1-oxaspiro[4.5]decan-2-one (5a)

^{1}H NMR (600 MHz, $CDCl_3$)

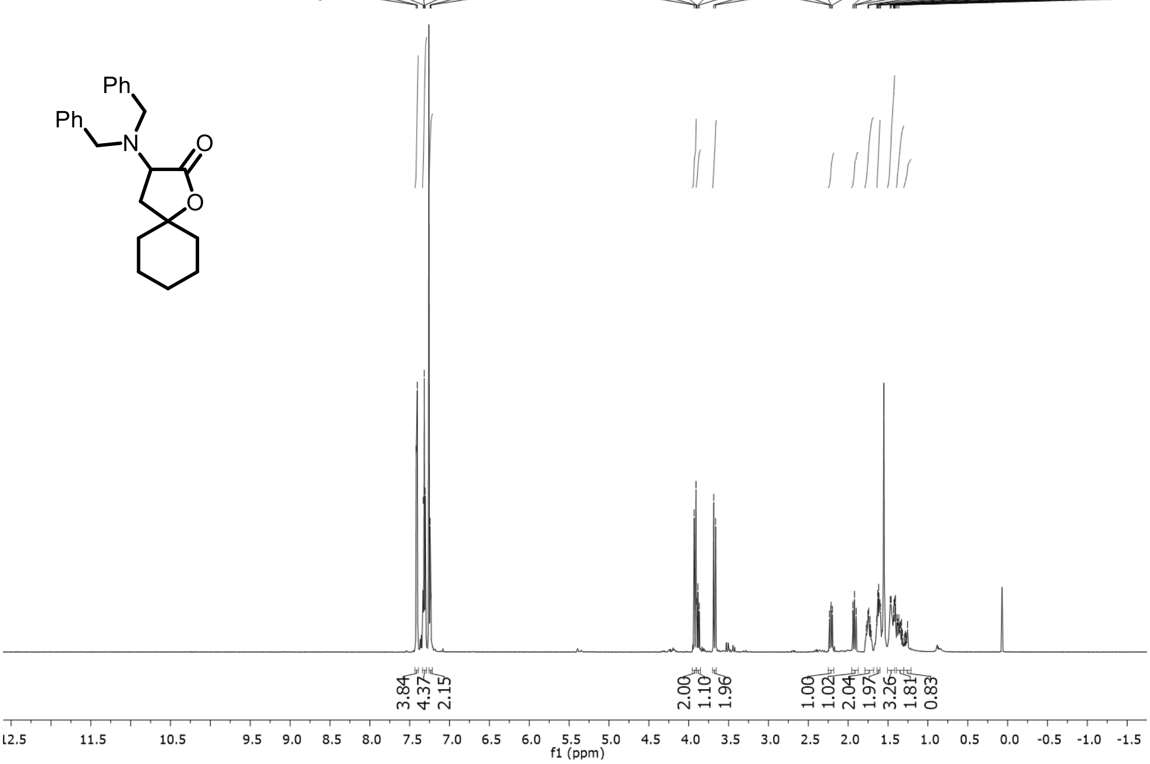

^{13}C NMR (151 MHz, $CDCl_3$)

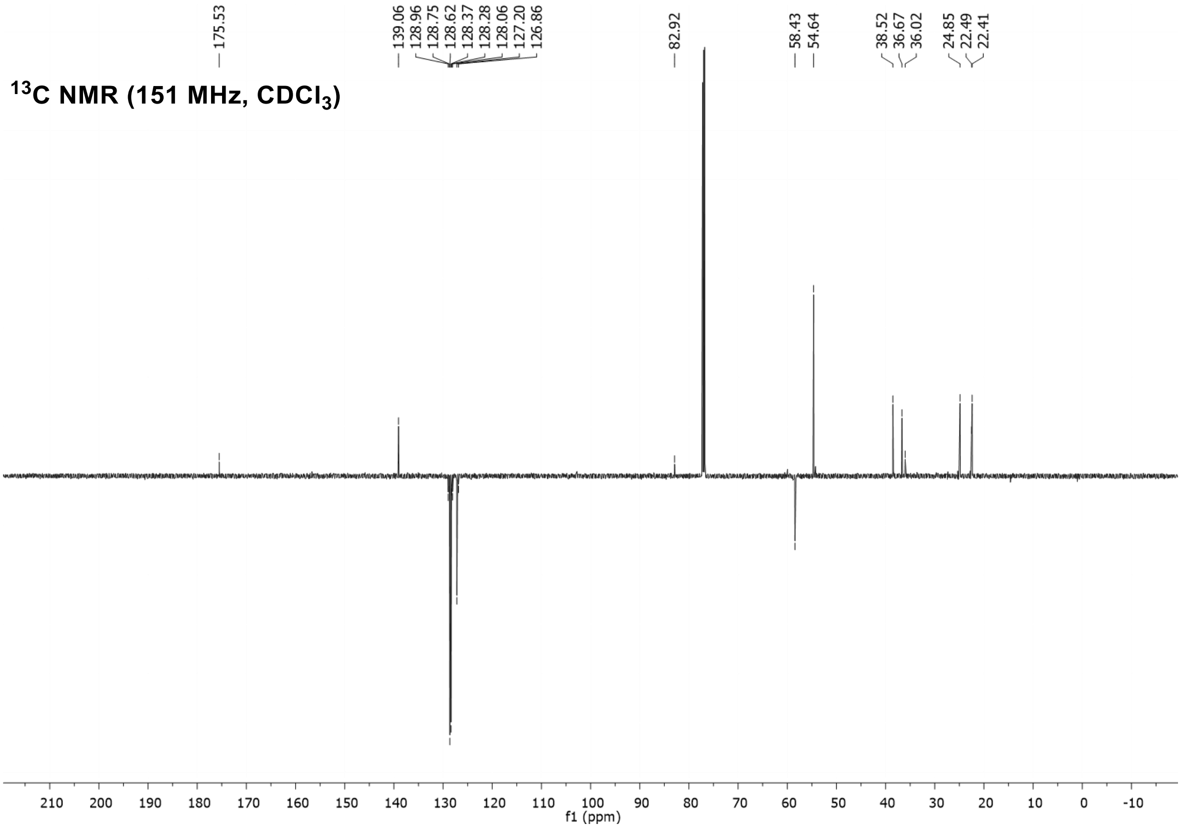

3-Morpholino-1-oxaspiro[4.5]decan-2-one (5b)

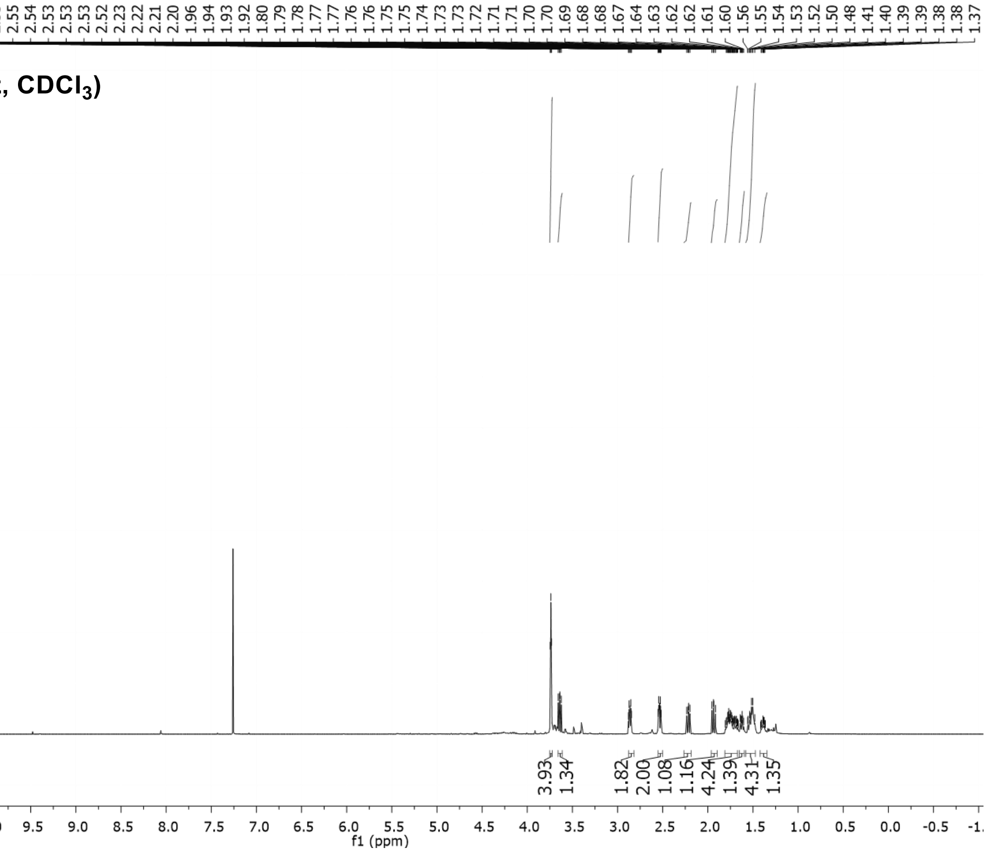

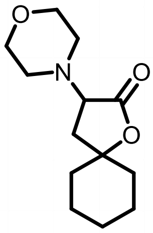

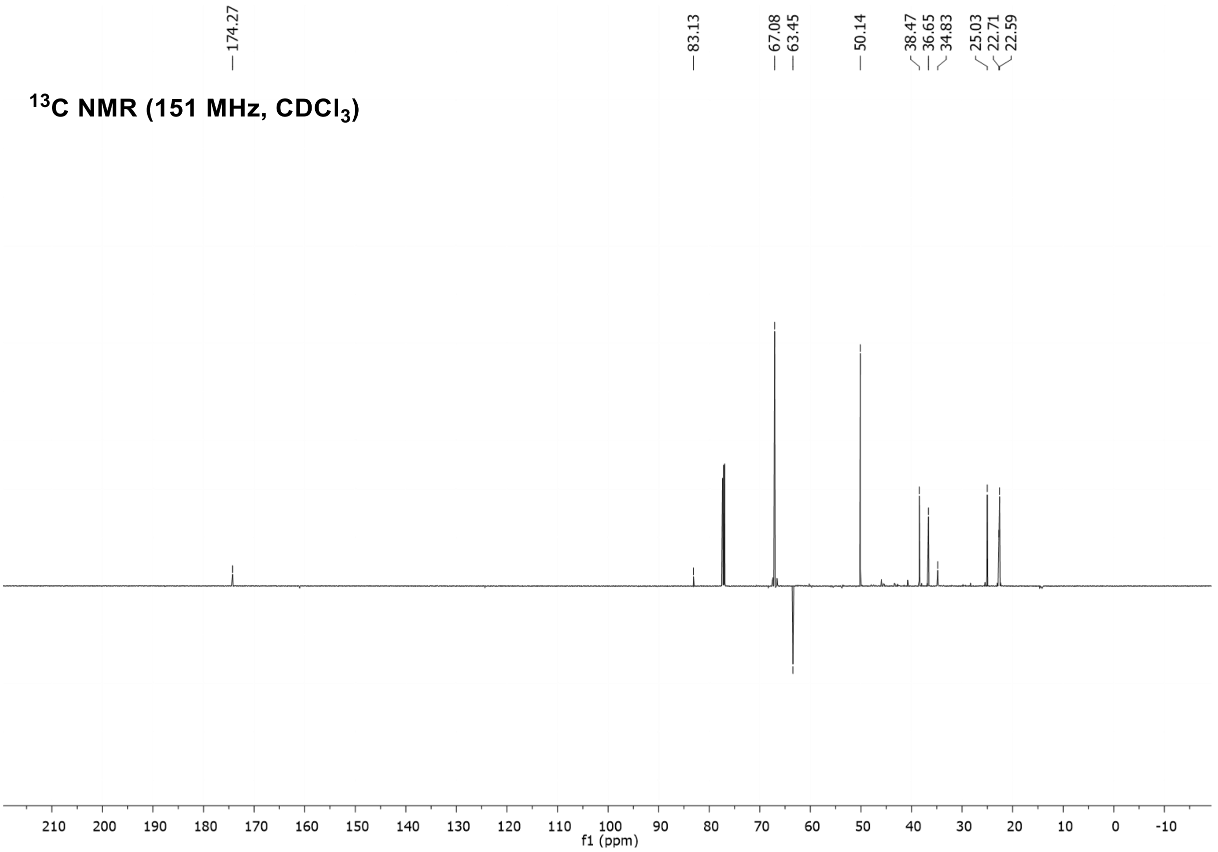

3-(Bis(2-hydroxyethyl)amino)-1-oxaspiro[4.5]decan-2-one (5c)

^{1}H NMR (600 MHz, $CDCl_3$)

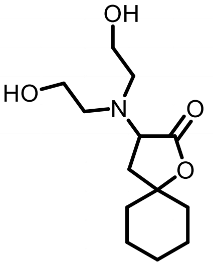

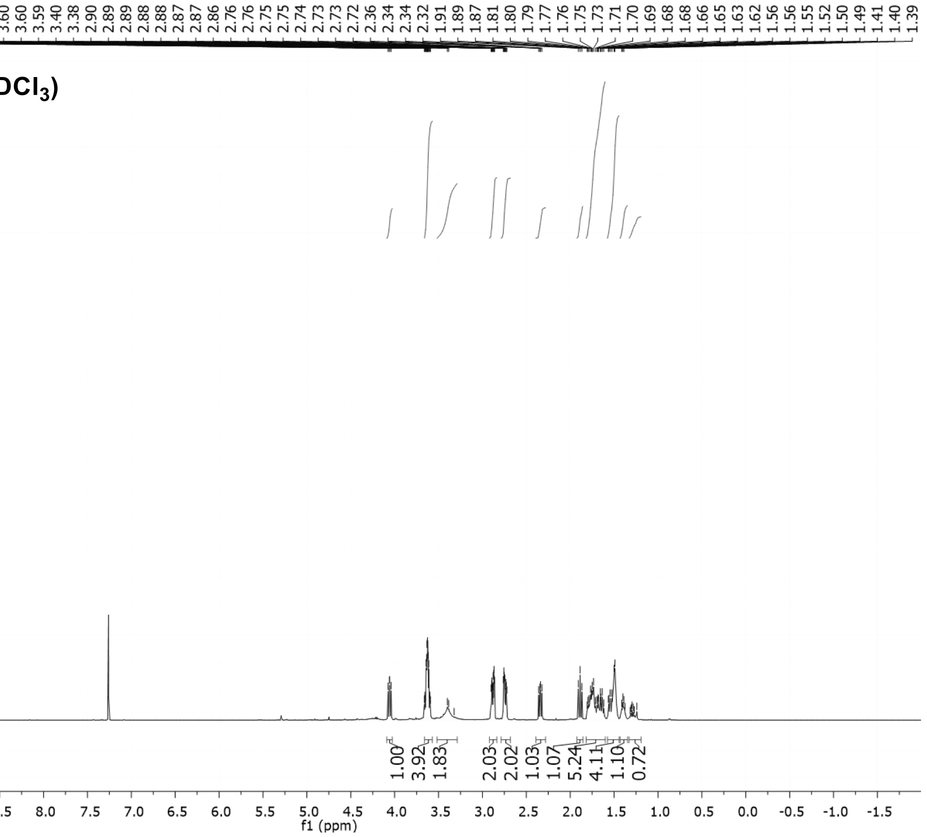

^{13}C NMR (151 MHz, $CDCl_3$)

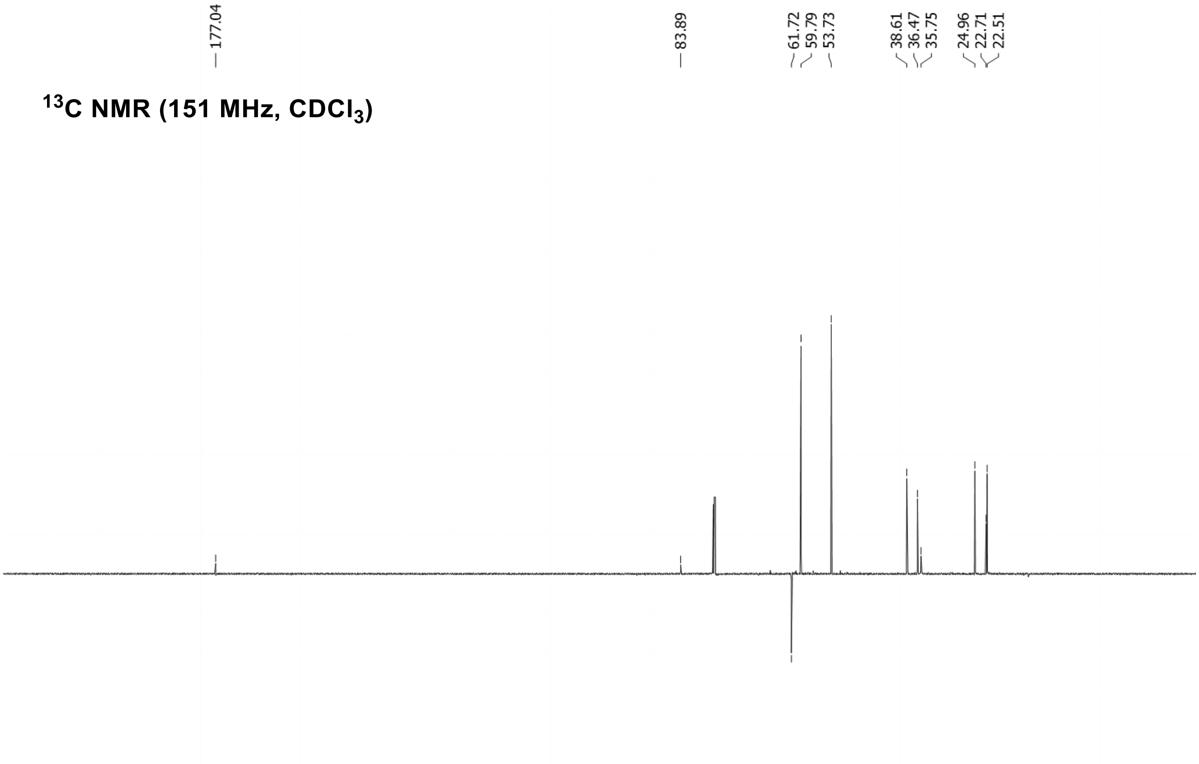

3-(Diallylamino)-1-oxaspiro[4.5]decan-2-one (5d)

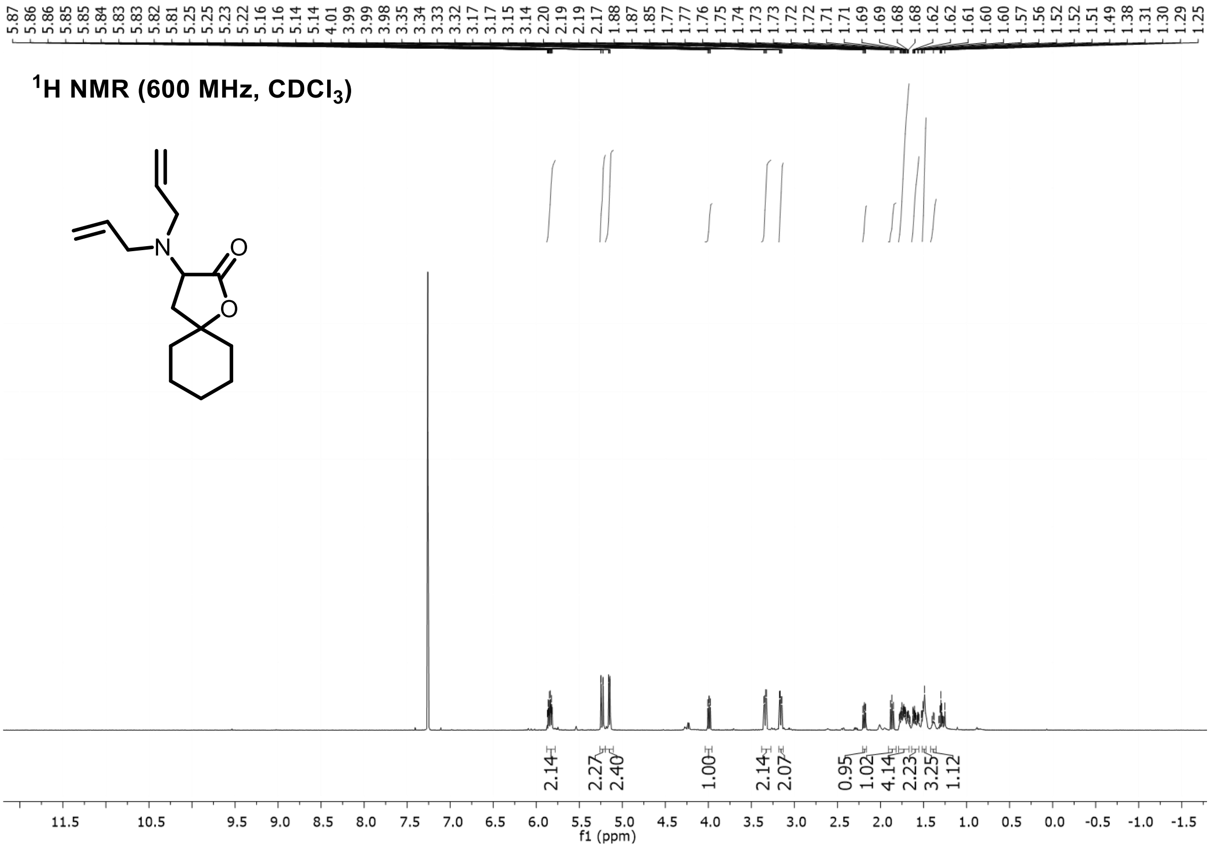

^{1}H NMR (600 MHz, $CDCl_3$)

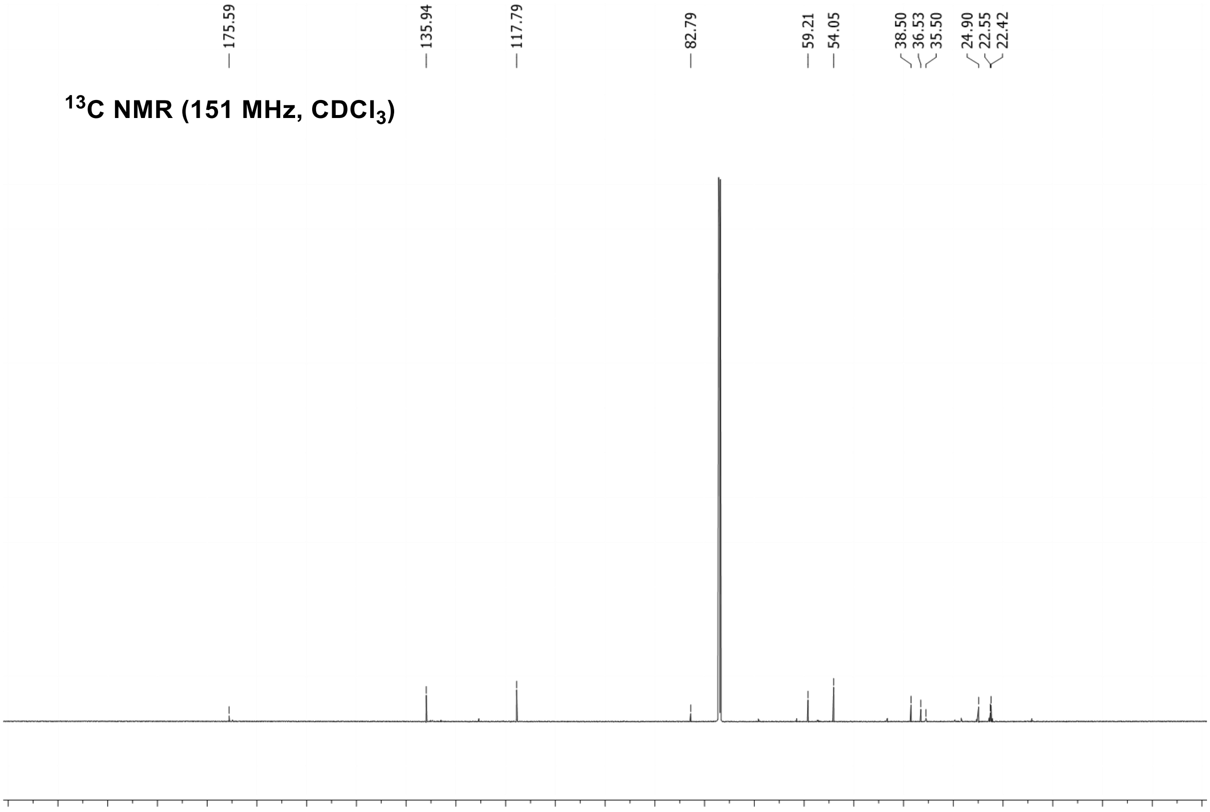

^{13}C NMR (151 MHz, $CDCl_3$)

(5s*,8s*)-8-Phenyl-1-oxaspiro[4.5]dec-3-en-2-one (6)

^{1}H NMR (400 MHz, $CDCl_3$)

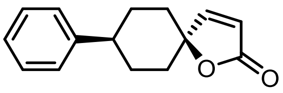

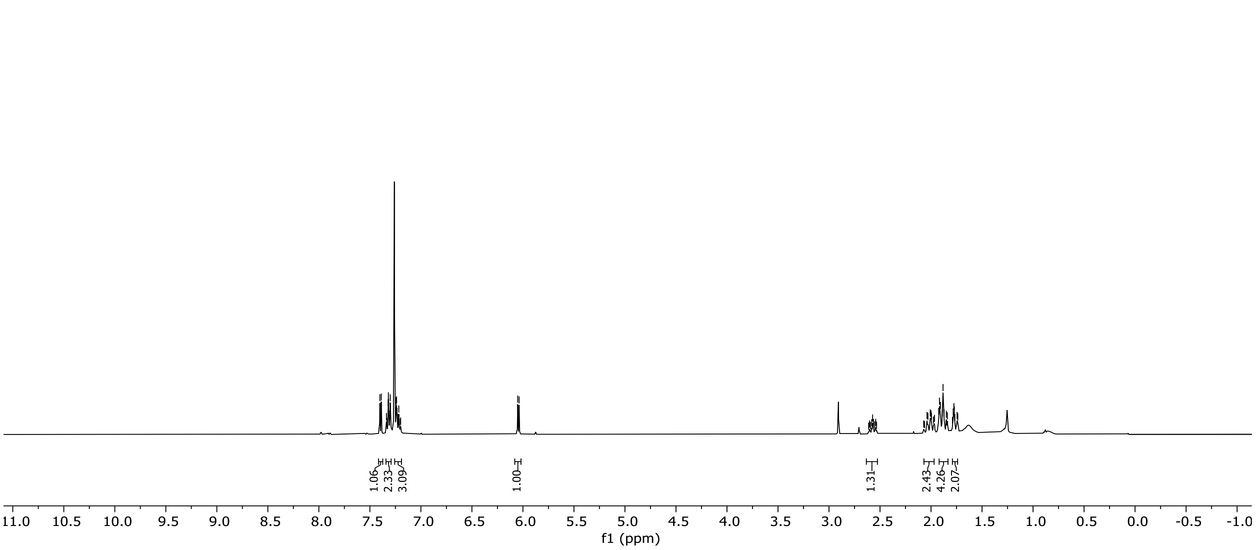

^{13}C NMR (101 MHz, $CDCl_3$)

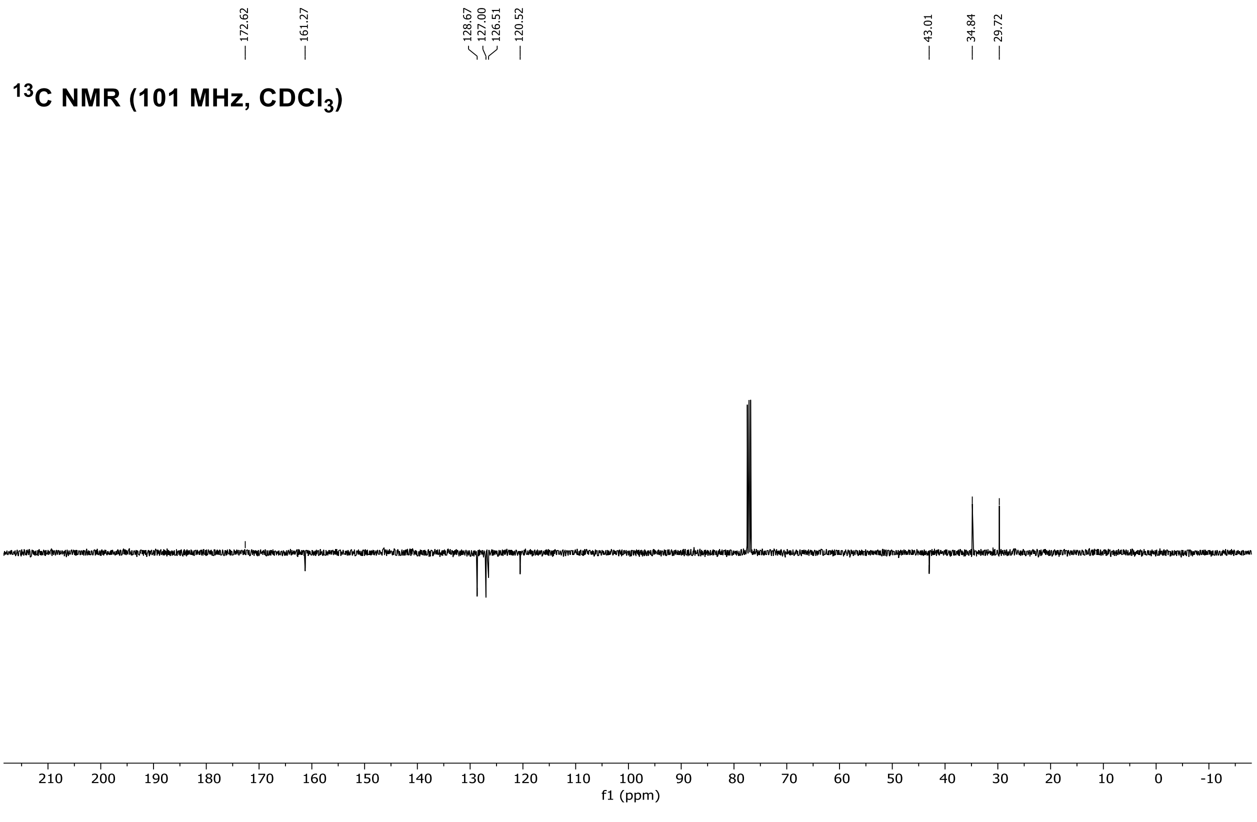

(5*s**,8*s**)-3-(Dimethylamino)-8-phenyl-1-oxaspiro[4.5]dec-3-en-2-one (7)

^{1}H NMR (400 MHz, CDCl$_3$)

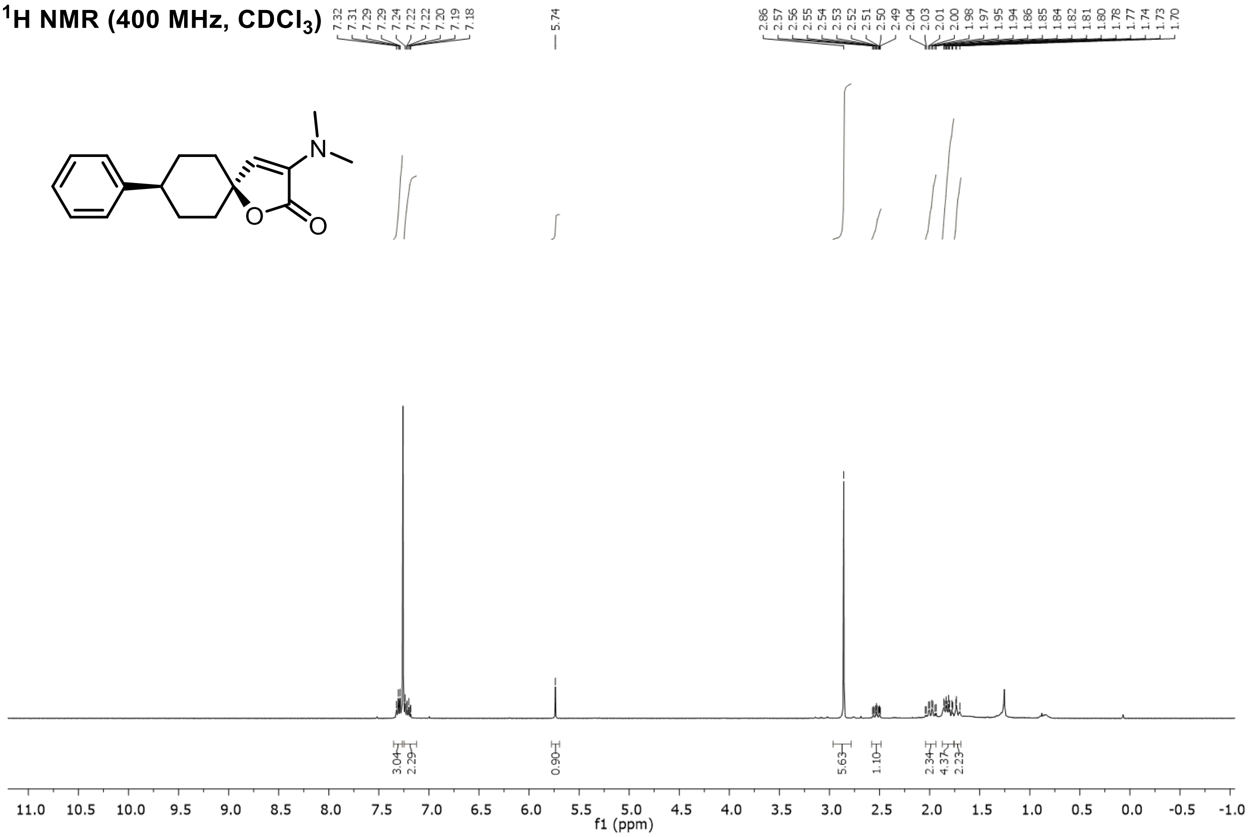

^{13}C NMR (101 MHz, CDCl$_3$)

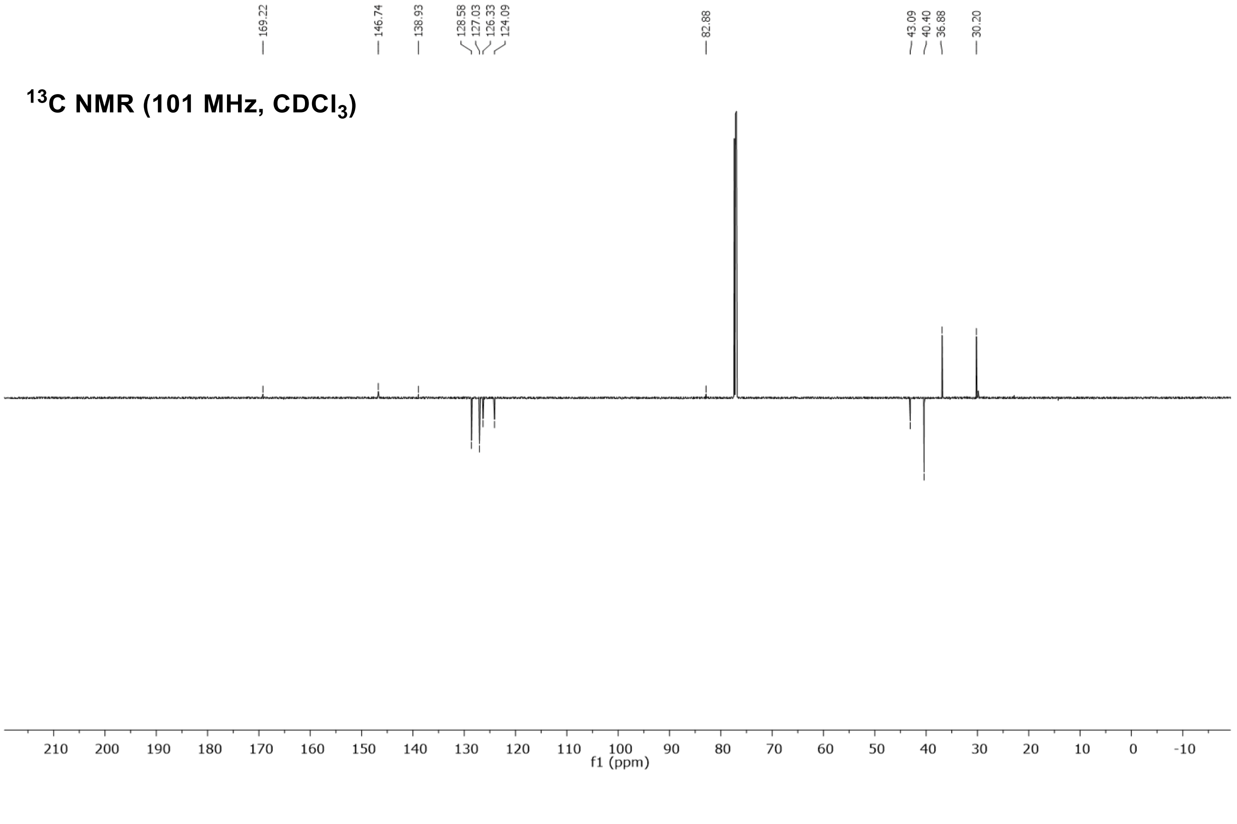

(3*S**,5s*,8*R**)-3-Allyl-3-(dimethylamino)-8-phenyl-1-oxaspiro[4.5]decan-2-one (8)

^{1}H NMR (400 MHz, CDCl$_3$)

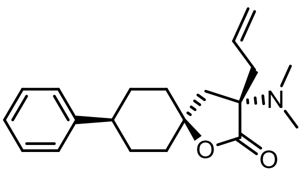

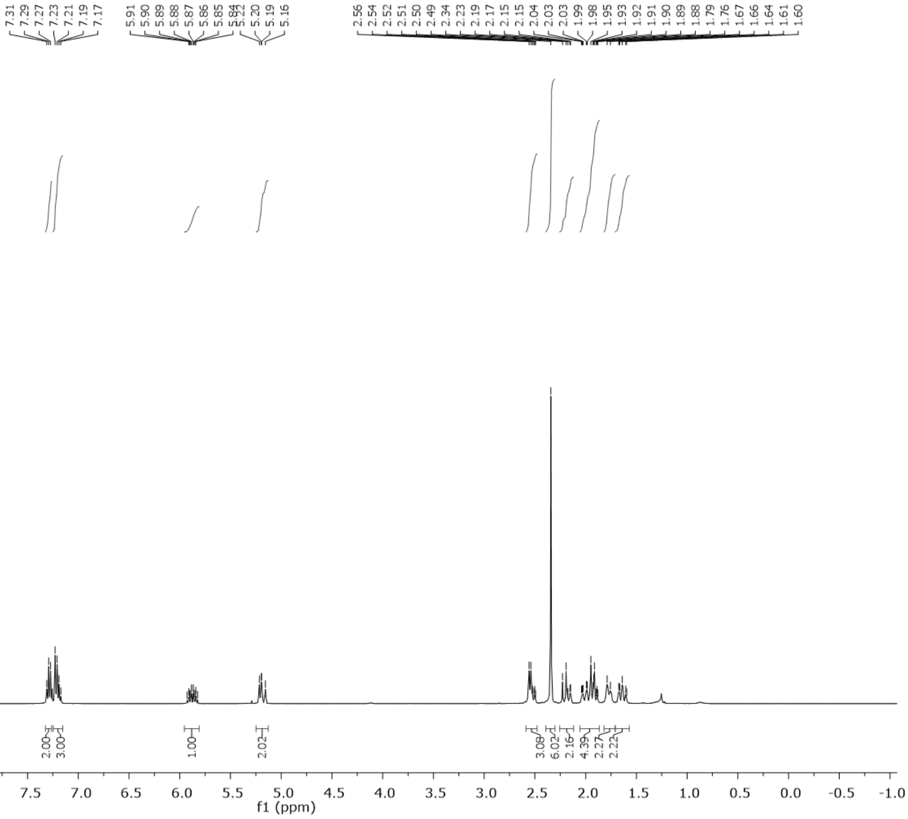

^{13}C NMR (101 MHz, CDCl$_3$)

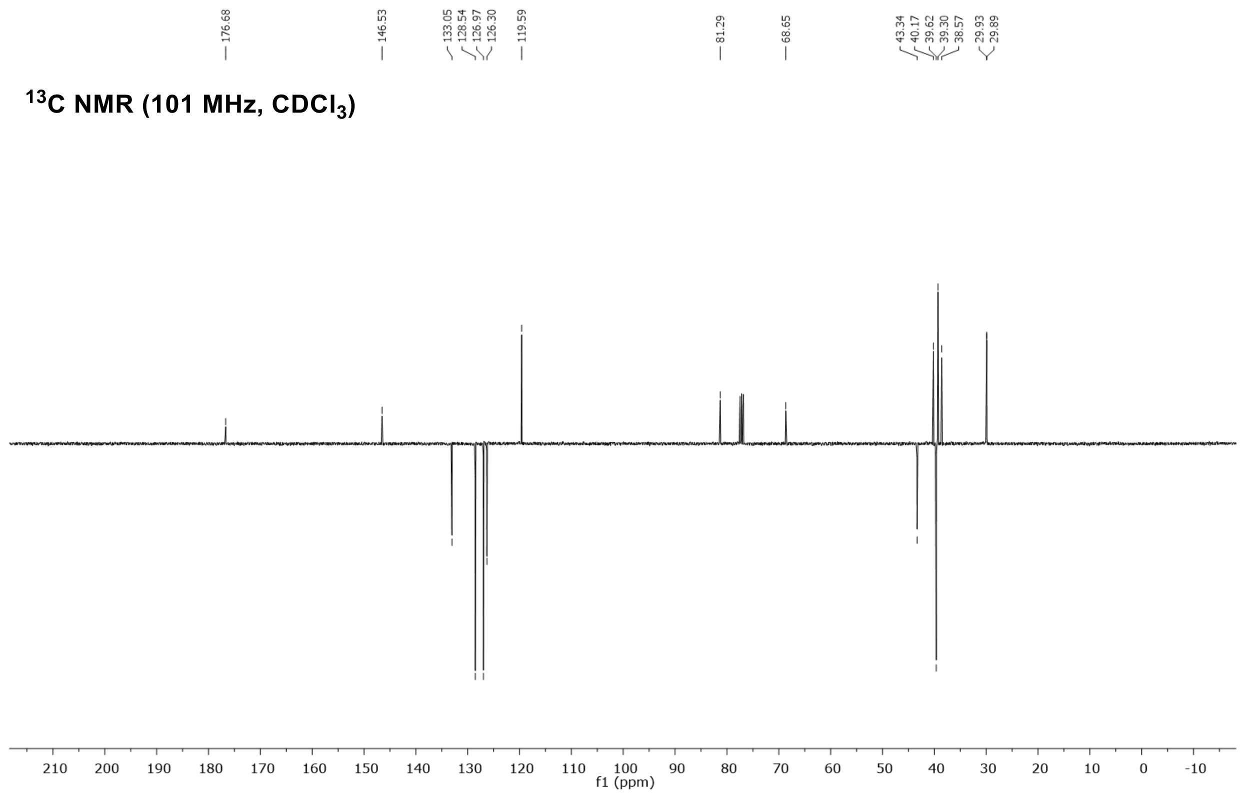

(3*R,5*s**,8*S**)-3-(Dimethylamino)-3-(2-methylbut-3-en-2-yl)-8-phenyl-1-oxaspiro[4.5]decan-2-one (9)**

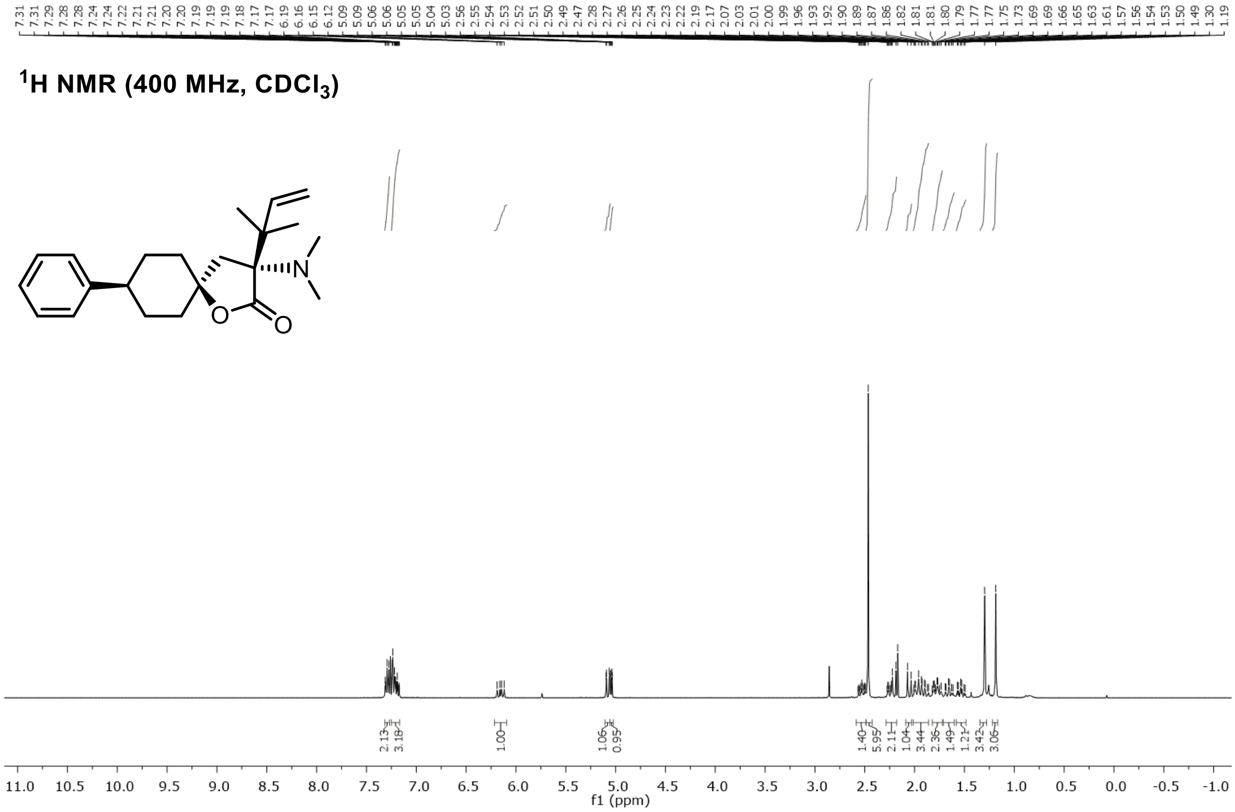

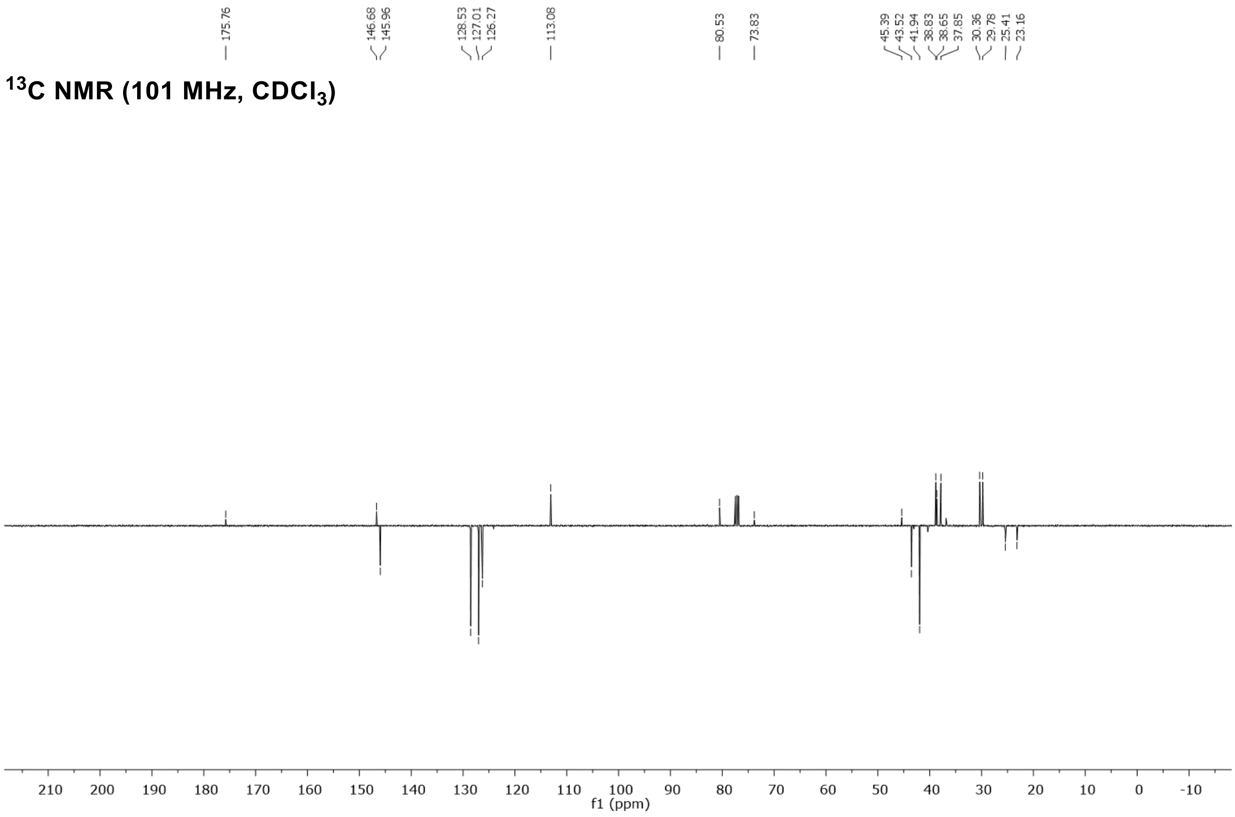

(3*R**,5*s**,8*S**)-3-(Dimethylamino)-8-phenyl-3-(propa-1,2-dien-1-yl)-1-oxaspiro[4.5]decan-2-one (10)

^{1}H NMR (400 MHz, $CDCl_3$)

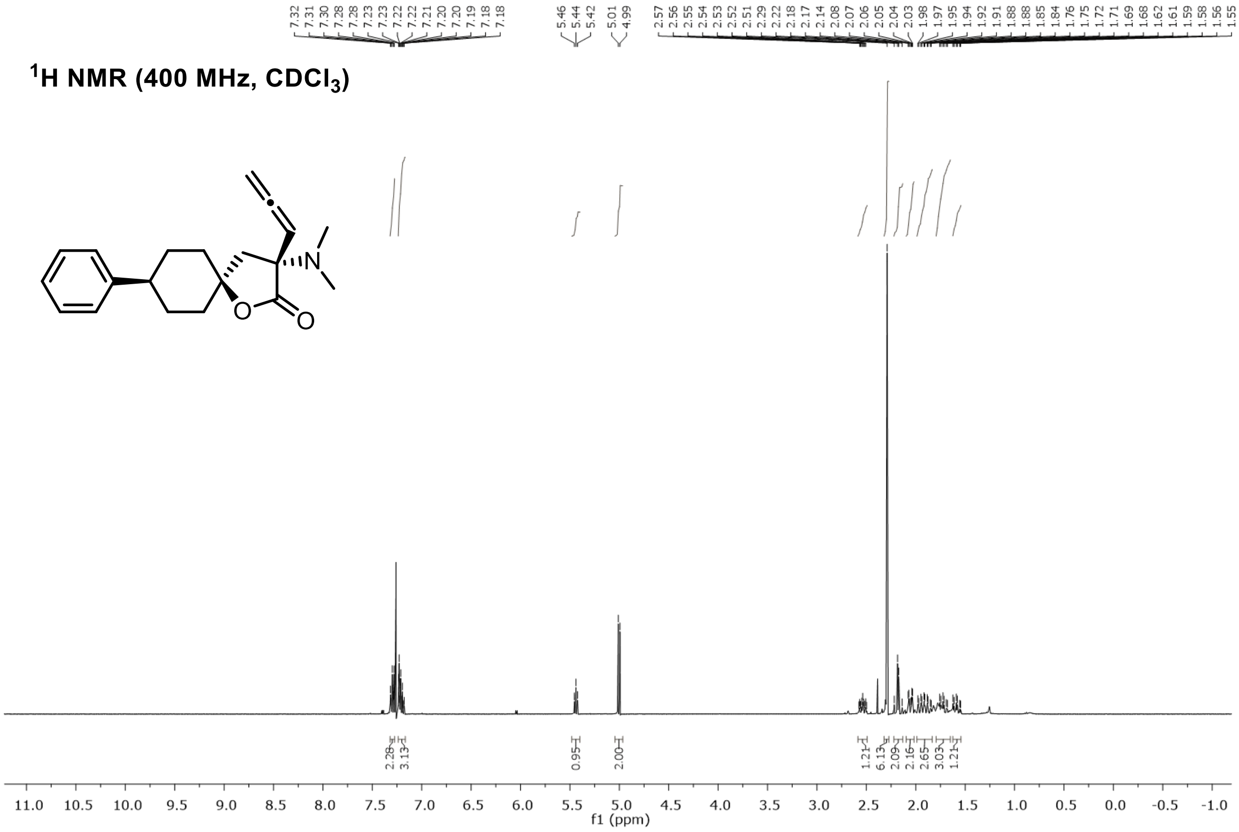

^{13}C NMR (101 MHz, $CDCl_3$)

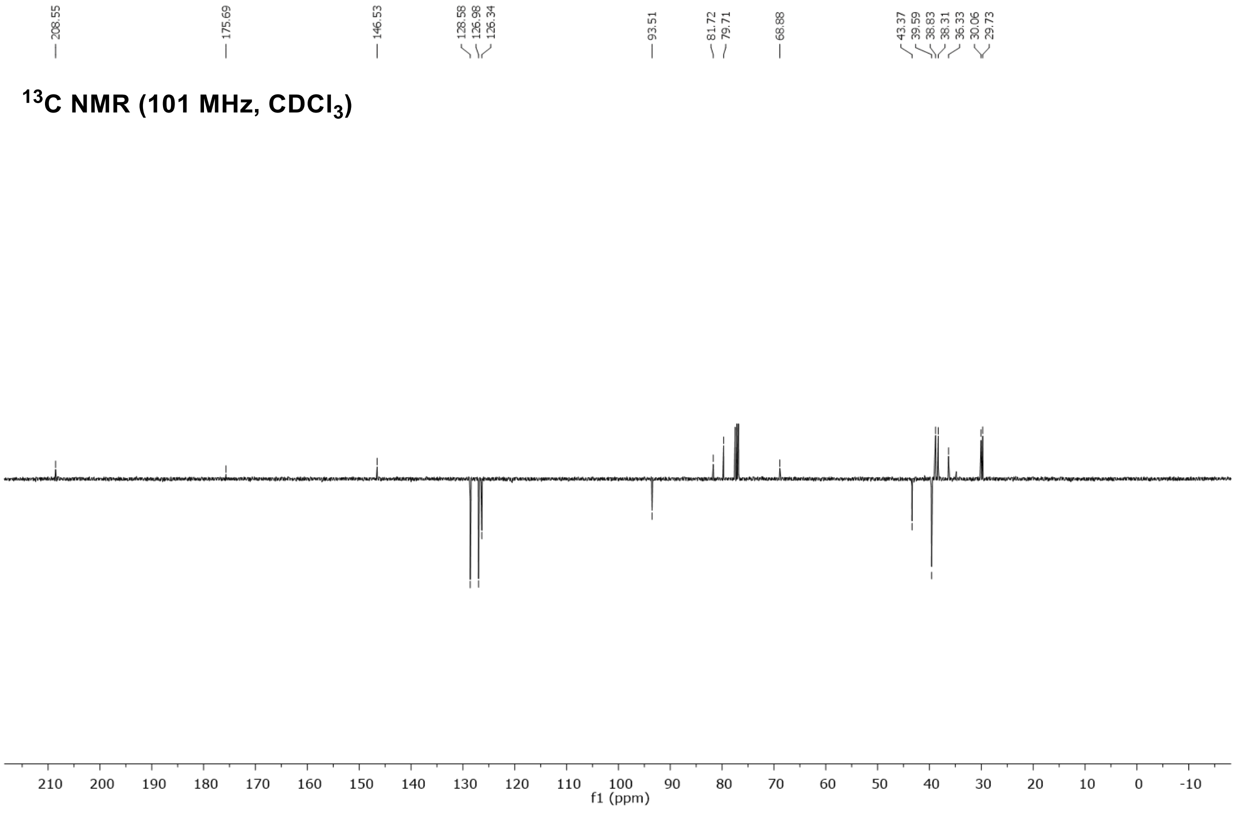

(3*S**,5*s**,8*R**)-3-Benzyl-3-(dimethylamino)-8-phenyl-1-oxaspiro[4.5]decan-2-one (11)

^{1}H NMR (400 MHz, $CDCl_3$)

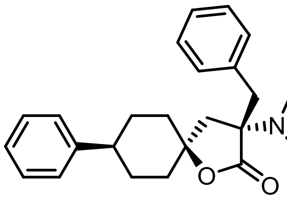

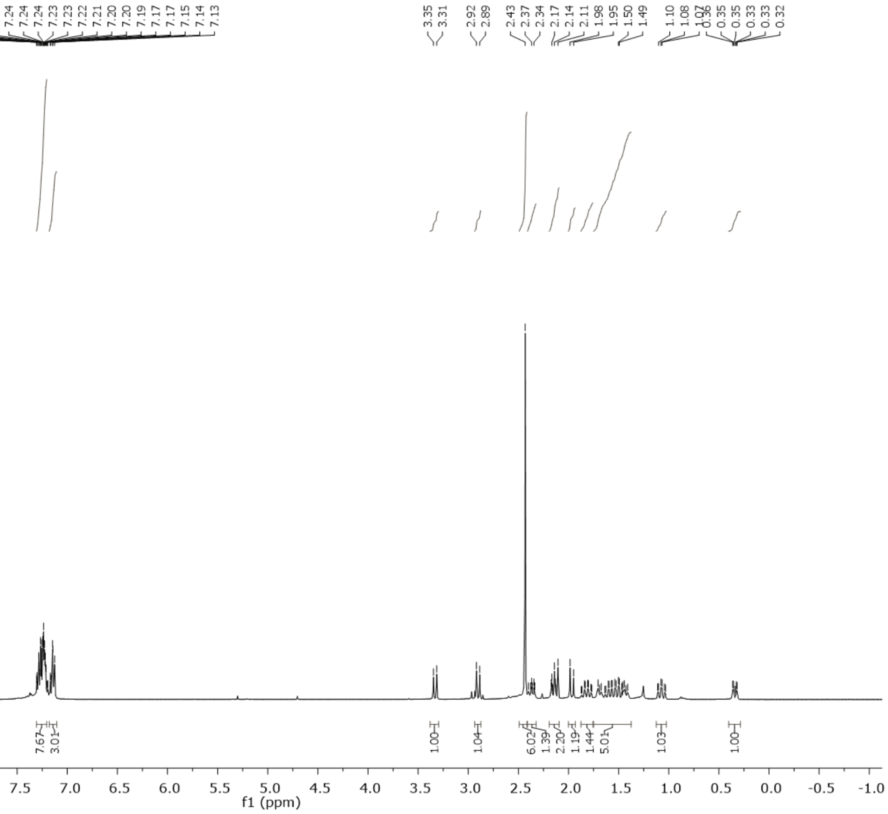

^{13}C NMR (101 MHz, $CDCl_3$)

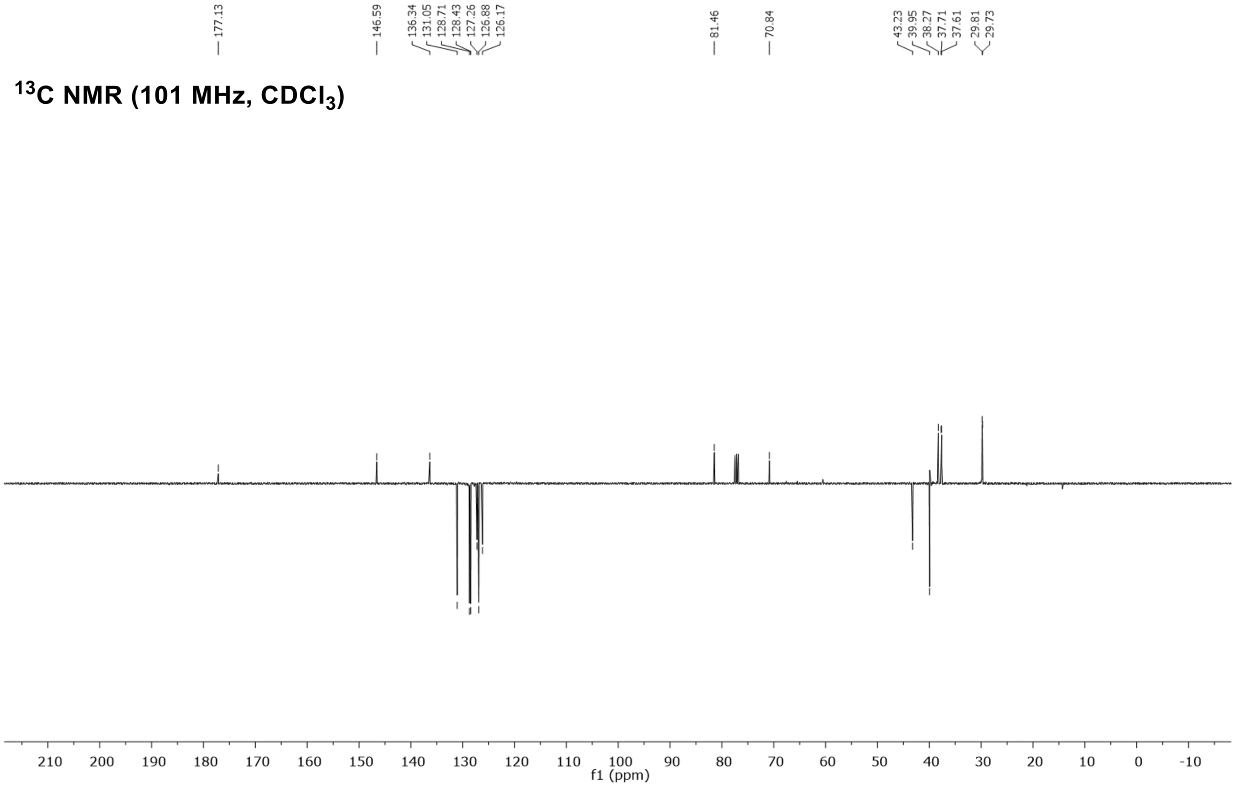

11. References

[1] C.-S. Hsu, C. R. Gonçalves, V. Tona, A. Pons, M. Kaiser, N. Maulide, *Angew. Chem. Int. Ed.* **2022**, *61*, e202115435.

[2] R. Gurbanov, A. Sokolov, S. Golovach, K. Melnykov, A. V. Dobrydnev, O. O. Grygorenko, *Synthesis* **2020**, *53*, 1771-1784.

[3] J. Barluenga, J. L. Fernández-Simón, J. M. Concellón, M. Yus, *J. Chem. Soc., Perkin Trans. 1* **1988**, 3339-3343.

[4] W. Luo, L. Lin, Y. Zhang, X. Liu, X. Feng, *Org. Lett.* **2017**, *19*, 3374-3377.

[5] X. Liu, X. Rong, S. Liu, Y. Lan, Q. Liu, *J. Am. Chem. Soc.* **2021**, *143*, 20633-20639.

[6] I. Ojima, M. Yatabe, T. Fuchikami, *J. Org. Chem.* **1982**, *47*, 2051-2055.

[7] J.-i. Hibino, T. Okazoe, K. Takai, H. Nozaki, *Tetrahedron Lett.* **1985**, *26*, 5579-5580.

[8] Y.-N. Yin, R.-Q. Ding, D.-C. Ouyang, Q. Zhang, R. Zhu, *Nat. Commun.* **2021**, *12*, 2552.

[9] I. Ryu, T. Aya, S. Otani, S. Murai, N. Sonoda, *J. Organomet. Chem.* **1987**, *321*, 279-290.

[10] S. Cren, P. Schär, P. Renaud, K. Schenk, *J. Org. Chem.* **2009**, *74*, 2942-2946.

[11] P. Metzger, E. Casadevall, M. J. Pouet, *Org. Mag. Reson.* **1982**, *19*, 229-234.

[12] J. Duran, D. Oliver, A. Polo, J. Real, J. Benet-Buchholz, X. Fontrodona, *Tetrahedron: Asymmetry* **2003**, *14*, 2529-2538.

[13] H. A. Kerchner, J. Montgomery, *Org. Lett.* **2016**, *18*, 5760-5763.

[14] T. Huang, C.-J. Li, *Tetrahedron Lett.* **2000**, *41*, 9747–9751.
